# Enhanced Reactivities of Iron(IV)‐Oxo Porphyrin Species in Oxidation Reactions Promoted by Intramolecular Hydrogen‐Bonding

**DOI:** 10.1002/advs.202310333

**Published:** 2024-03-13

**Authors:** Zhe Gong, Liwei Wang, Yiran Xu, Duanfeng Xie, Xiaotian Qi, Wonwoo Nam, Mian Guo

**Affiliations:** ^1^ College of Chemistry and Molecular Sciences Wuhan University Wuhan Hubei 430072 P. R. China; ^2^ Department of Chemistry and Nano Science Ewha Womans University Seoul 03760 South Korea

**Keywords:** biomimetic system, H‐bonding, high‐valent iron‐oxo species, kinetic study, mechanism

## Abstract

High‐valent iron‐oxo species are one of the common intermediates in both biological and biomimetic catalytic oxidation reactions. Recently, hydrogen‐bonding (H‐bonding) has been proved to be critical in determining the selectivity and reactivity. However, few examples have been established for mechanistic insights into the H‐bonding effect. Moreover, intramolecular H‐bonding effect on both C‐H activation and oxygen atom transfer (OAT) reactions in synthetic porphyrin model system has not been investigated yet. In this study, a series of heme‐containing iron(IV)‐oxo porphyrin species with or without intramolecular H‐bonding are synthesized and characterized. Kinetic studies revealed that intramolecular H‐bonding can significantly enhance the reactivity of iron(IV)‐oxo species in OAT, C‐H activation, and electron‐transfer reactions. This unprecedented unified H‐bonding effect is elucidated by theoretical calculations, which showed that intramolecular H‐bonding interactions lower the energy of the anti‐bonding orbital of iron(IV)‐oxo porphyrin species, resulting in the enhanced reactivities in oxidation reactions irrespective of the reaction type. To the best of the knowledge, this is the first extensive investigation on the intramolecular H‐bonding effect in heme system. The results show that H‐bonding interactions have a unified effect with iron(IV)‐oxo porphyrin species in all three investigated reactions.

## Graphical abstract

Kinetic studies of heme containing iron(IV)‐oxo species with intramolecular hydrogen bonding exhibited unprecedented unified enhanced reactivities in oxygen‐atom transfer, C‐H bond activation and electron transfer reactions. Theoretical calculations demonstrated that hydrogen bonding is capable of lowering the energy of anti‐bonding orbitals of iron(IV)‐oxo porphyrin species, making it a more powerful oxidant.

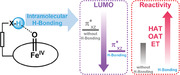

## 1 Introduction

High‐valent iron‐oxo complexes are key intermediates in catalytic oxidation processes in both enzymatic and biomimetic systems.^[^ ^1^, ^2^, ^3^, ^4^ ^]^ For example, iron(IV)‐oxo porphyrin *π*‐cation radical species (compound I, Cpd I) has been considered to be the reactive intermediate in C‐H activation and oxygen atom transfer (OAT) in cytochrome P450 and synthetic metalloporphyrin catalysis.^[^ ^2^, ^5^ ^]^ Its one‐electron reduced form, iron(IV)‐oxo porphyrin complex (compound II, Cpd II)^[^ ^2^, ^6^ ^]^ and the related non‐heme iron(IV)‐oxo analogues^[^ ^3^, ^4^, ^7^ ^]^ have also been proved to be responsible for various oxidation reactions. Due to their intriguing oxidation capability, many studies have investigated the effect of ligands on the structure and reactivity of high‐valent iron‐oxo complexes, such as the electronic effect,^[^ ^4^, ^5^, ^8^ ^]^ ring size of the equatorial ligand,^[^ ^9^ ^]^ ligand topology^[^ ^2^, ^10^ ^]^ and axial ligand effect.^[^ ^5^, ^11^ ^]^ Inspired by the comprehensive ligand effects, a variety of powerful catalysts for oxidation reactions or oxidative functionalization reactions have been developed that exhibit remarkable potential uses for synthetic applications.^[^ ^12^ ^]^ Despite this success, many challenges still remain, especially with the low selectivity and catalytic efficiency in biomimetic systems compared with natural enzymatic systems.^[^ ^1b^ ^]^

The well‐studied ligand effects, either the equatorial ligand or the axial ligand, which are directly binding to the metal center through covalent bonding are regarded as the “first‐coordination sphere”.^[^ ^13^ ^]^ However, in enzymatic systems, the protein active sites have also evolved to establish a complex network of weak non‐covalent interactions, defined as the “second‐coordination sphere”, such as H‐bonding.^[^ ^14^ ^]^ Although H‐bonding is weak compared with the “first‐coordination sphere” interactions, it is still able to influence the electronic configuration and molecular orbital of the reactive high‐valent iron‐oxo species. For examples, the redox potential, p*K*_a_ values and other thermochemical properties of the oxidants which determine the reactivity of oxidative reactions are regulated by the electronic perturbation through H‐bonding effect.^[^ ^14^ ^]^ For instances, in heme proteins, the Cpd II intermediate is stabilized by H‐bonding interactions with nearby amino acid residues to prevent side oxidative damage to the enzymes;^[^ ^11^, ^15^ ^]^ H‐bonding interactions with the active site of P450 Cpd I were found to change the redox potential and the spin density distribution of the active oxidant.^[^ ^16^ ^]^ Therefore, extensive studies of the H‐bonding effect on the high‐valent iron‐oxo species are essential for the deeper understanding of the mechanism in enzymatic systems and development of novel biomimetic systems.

Although it is believed that the oxygen‐atom of iron‐oxo species interacts with nearby H‐bonding donors, such as P450cam Cpd I interacting with hydroxy group of Thr252,^[^ ^17^ ^]^ the mechanism for the influence of H‐bonding on the iron‐oxo species during the oxidation process is not understood.^[^ ^18^ ^]^ Because of the instability of the highly reactive iron‐oxo species and the complexity of the enzyme structures, it is difficult to investigate the detailed contribution of the H‐bonding effect on the reactive intermediate. One approach is the use of synthetic iron‐oxo models mimicking the structure and function of enzymatic active sites, in which the structural features are tunable and easier for spectroscopic characterization and reactivity investigation.^[^ ^1^, ^2^, ^4^, ^14^ ^]^ Therefore, synthetic iron‐oxo models installed with H‐bonding interactions may allow us to gain a detailed knowledge of the H‐bonding effect on iron‐oxo species in both biological and biomimetic systems. However, the design and synthesis of biomimetic iron‐oxo or other metal‐oxo models with unambiguous H‐bonding interactions is still challenging because the weak non‐covalent interaction is difficult to characterize. Moreover, introduction of hydrogen donor to form the H‐bonding with high‐valent iron‐oxo species favors the formation of related Fe‐OH species, because of the strong basicity of iron‐oxo moiety.^[^ ^2^, ^21^ ^]^ To date, there have been limited examples. Nocera and co‐workers had developed “Hangman” porphyrin complexes with a carboxylic acid group positioned at the active site to form an intramolecular H‐bonding, but the details of the H‐bonding effect on the iron‐oxo species were not well established.^[^ ^19^ ^]^ Borovik and co‐workers had developed a tripodal ligand [H_3_buea]^3‐^ with urea arms that could potentially form H‐bonding with metal‐oxo moiety. Further studies have shown that the oxidative capability of the related iron‐oxo or manganese‐oxo species will be weakened by the H‐bonding interaction.^[^ ^20^ ^]^ However, Karlin and co‐workers demonstrated that the intermolecular H‐bonding could enhance the reactivity of the iron(IV)‐oxo porphyrin complex in C‐H activation reactions.^[^ ^21^ ^]^ Very recently, Kojima and co‐workers reported that intramolecular H‐bonding resulted in enhancement of OAT reactivity of Ru(IV)‐oxo species but decreased its reactivity in phenol oxidation.^[^ ^22^ ^]^ Apparently, the H‐bonding effect may result in either a positive or negative influence on the oxidation properties of metal‐oxo species, depending on the ligand topology, metal center and reaction type. Therefore, a distinct H‐bonding effect on heme‐containing iron‐oxo species compared with non‐heme systems could be expected in various oxidation reactions. However, extensive studies of the intramolecular H‐bonding effect on high‐valent iron‐oxo porphyrin species have not been explored yet. Moreover, the H‐bonding effect of high‐valent metal‐oxo species on both C‐H activation and OAT reactions in identical system has not been explored yet.

**Scheme 1:**
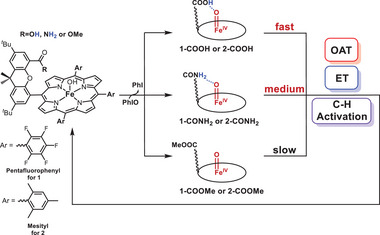
Synthesis of iron(IV)‐oxo porphyrin species and reactivity studies in oxygen atom transfer (OAT), C‐H activation and electron‐transfer (ET) reactions.

**Figure 1:**
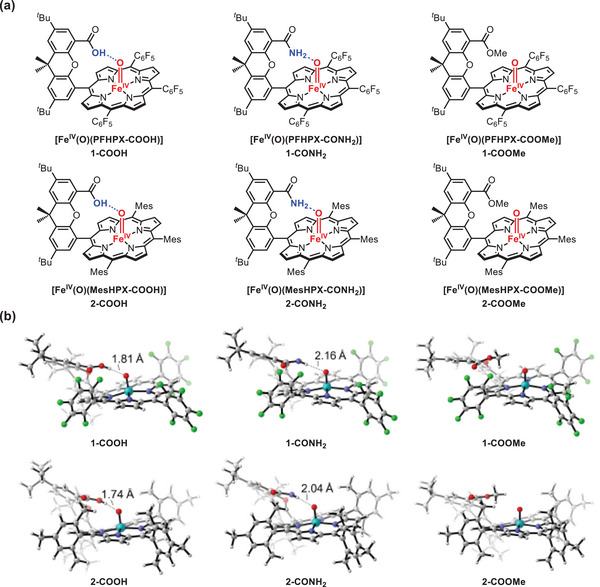
a) Structures of investigated iron(IV)‐oxo porphyrin species. b) Theoretical simulation of the structures of triplet iron(IV)‐oxo porphyrin species. See Section 1 for details.

Herein, we synthesized a series of iron(III) porphyrin complexes based on the “Hangman” porphyrin ligand. The ferric porphyrin complexes were constructed with a carboxylic acid group, an amide group, or an ester group attached to the xanthene scaffold. The former two complexes could form intramolecular H‐bonds with the in‐situ generated iron(IV)‐oxo Cpd II species (**Scheme** **1** and **Figure** **1**). The well‐known Cpd II analogue [Fe^IV^(O)(TMP)] bearing symmetrical porphyrin ligand was also synthesized for comparison. All the Cpd II species were fully characterized by various spectroscopies and structurally optimized using density functional theory (DFT) calculations. Reactivity and kinetic studies in OAT, C‐H activation, and electron‐transfer reactions were performed to investigate the intramolecular H‐bonding effect on Cpd II. An unprecedented unified H‐bonding effect that enhanced the oxidizing power of iron(IV)‐oxo species in all three kinds of reactions was demonstrated. DFT calculations were also used to elucidate the electronic configuration and molecular orbital energy changes caused by the H‐bonding interactions, which eventually promoted the oxidation reactions by Cpd II. Taken together, the experimental and computational results provide deep insight into the mechanism of the H‐bonding effect on iron‐oxo species. This is helpful to understand the “second‐coordination sphere” in enzymes and will aid development of efficient bioinspired catalysis.

## 2 Results and Discussion

### 2.1 Synthesis and Characterization of Iron(IV)‐Oxo Porphyrin Complexes (Cpd II)

**Figure 2:**
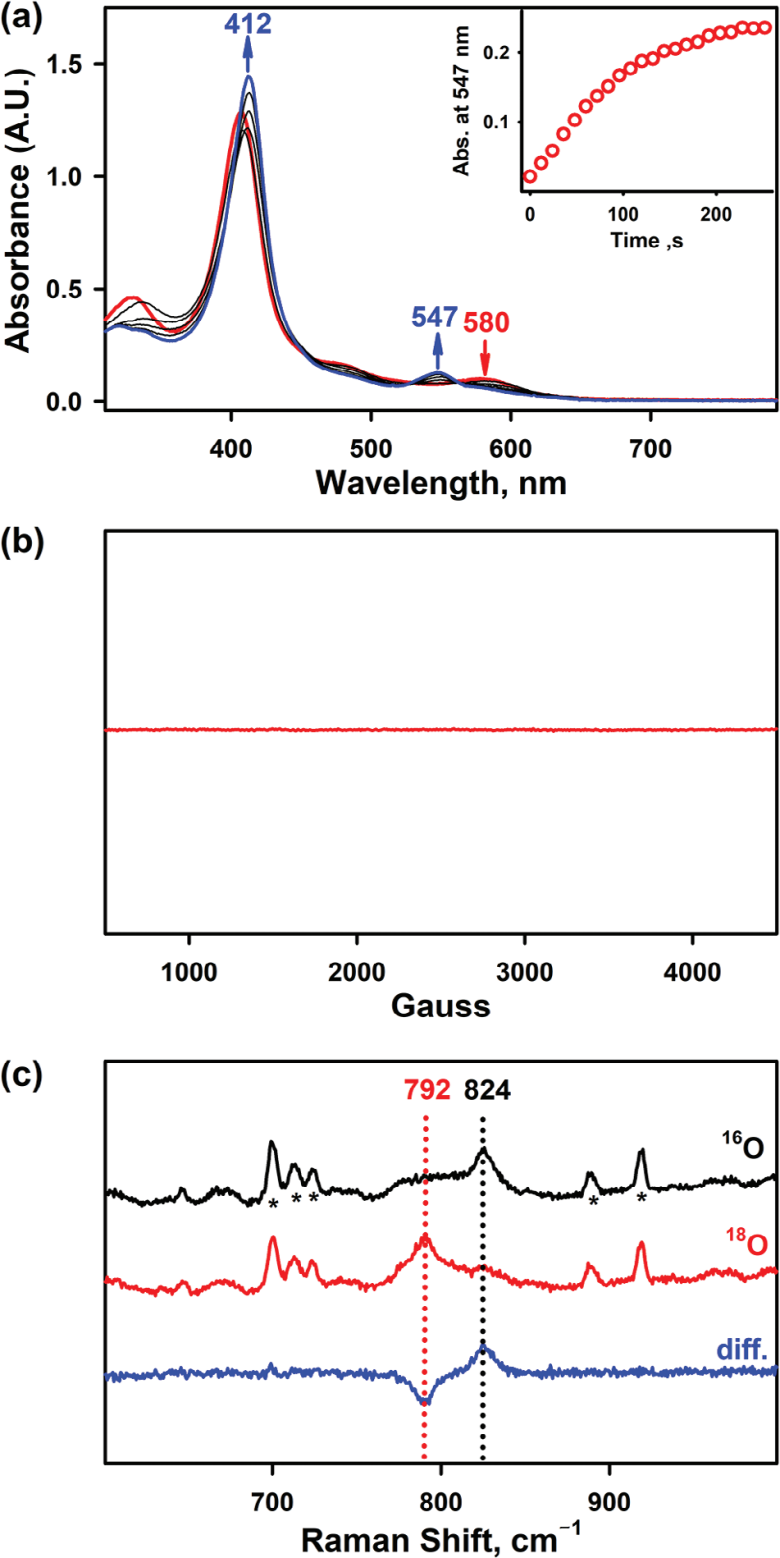
a) UV−vis spectral changes showing the formation of **1‐COOH** (blue line) in the reaction of [Fe^III^(PFHPX‐COOH)(OH)] (1.0 × 10^−2^ mm, red line) and PhIO (2.5 × 10^−2^ mm) in CH_3_CN/CH_3_OH (v/v 100:1) at 258 K. The inset shows the time trace monitored at 547 nm due to the formation of **1‐COOH**. b) X‐band EPR spectrum of **1‐COOH**. The spectrum was recorded at 90 K. c) rRaman spectra of **1‐COOH‐^16^O** (1.0 mm, black line) and **1‐COOH‐^18^O** (1.0 mm, red line). The blue line shows the difference spectrum of **1‐COOH‐^16^O** and **1‐COOH‐^18^O**. The peaks marked with asterisks (*) are from the solvent. The rRaman spectra were recorded with 442 nm excitation in acetone‐*d*_6_: CH_3_CN (v/v, 1:1) at 77 K.

The ferric Hangman porphyrin hydroxo complexes were synthesized according to the literature procedures (see the Supporting Information (SI) for synthesis details).^[^ ^19^ ^]^ X‐band electron paramagnetic resonance (EPR) spectroscopy of [Fe^III^(PFHPX‐COOH)(OH)] exhibited a peak at *g* = 6.2, indicating a high‐spin ferric complex (Figure S1a, Supporting Information). The corresponding iron(IV)‐oxo Hangman porphyrin species [Fe^IV^(O)(PFHPX‐COOH)], denoted as **1‐COOH**, was prepared by reacting [Fe^III^(PFHPX‐COOH)(OH)] with 2.5 equiv of iodosylbenzene (PhIO) in the solvent mixture of acetonitrile/CH_3_OH (v/v, 100:1) at 258 K.^[^ ^23^ ^]^ The color of the reaction solution changed from brown to red within 3 min, accompanied by the shift of Soret band from 410 to 412 nm and Q‐band from 580 to 547 nm with clean isosbestic points (**Figure** **2**a). Formation of **1‐COOH** may be from the comproportionation or decay of the initially generated Cpd I species, as reported by Nam, van Eldik and their co‐workers.^[^ ^1^, ^6^ ^]^ **1‐COOH** was metastable (*t*_1/2_ ≈30 min, Table S3, Supporting Information) at 258 K, which allowed us to characterize it with various spectroscopic techniques. The EPR spectrum of **1‐COOH** was silent, suggesting an Fe(IV) oxidation state (Figure 2b).^[^ ^6^, ^21^ ^]^ The electron spray ionization mass spectrometry (ESI‐MS) of **1‐COOH** exhibited a prominent ion peak at a mass‐to‐charge ratio (*m*/*z*) of 1242.2 and an isotopic distribution pattern corresponding to the mass of [Fe(O)(PFHPX‐COOH)] (calcd. *m*/*z* = 1242.2) (Figure S2, Supporting Information). When the reaction was carried out by using ^18^O‐labeled PhI^18^O, the ESI‐MS exhibited a peak at *m*/*z* = 1244.2, which corresponded to [Fe(^18^O)(PFHPX‐COOH)] (calcd. *m*/*z* = 1244.2). The two‐mass unit shift in the ^18^O‐labeled experiment suggests that **1‐COOH** is an iron‐oxo porphyrin complex containing one oxygen atom derived from the oxidant PhIO. The resonance Raman (rRaman) spectroscopy of **1‐COOH**, measured upon 442 nm excitation at 77 K, displayed one isotopically sensitive band at 824 cm^–1^, which shifted to 792 cm^–1^ upon ^18^O‐substitution (Figure 2c). The observed isotopic shift of –32 cm^–1^ with ^18^O‐substitution is in good agreement with the calculated value for a diatomic Fe–O oscillator (–37 cm^–1^). Therefore, the rRaman data indicates that **1‐COOH** possesses an Fe = O unit, which is consistent with the reported iron(IV)‐oxo porphyrin species (*ν*(Fe = O) in the range of 780 to 850 cm^−1^).^[^ ^24^ ^]^

When other ferric Hangman porphyrin complexes bearing electron‐deficient or electron‐rich substituents, such as carboxylic acid, amide, or ester group, were treated under identical conditions, similar results were obtained. As indicated by the ultraviolet–visible (UV‐vis) spectral changes, corresponding iron(IV)‐oxo porphyrin species were formed. These species were further characterized by EPR, ESI‐MS, and rRaman spectroscopies as well (Figures S3−S12, Supporting Information). It is noteworthy that the rRaman spectrum of **1‐COOMe**, in which the intramolecular H‐bonding was absent, was very close to that of **1‐COOH** (Figure 2c; Figure S5c, Supporting Information). Moreover, the Fe‐O single bond of Fe(IV)‐OH species exhibited a band at 574 cm^−1^ in rRaman spectroscopy, which was much smaller than that of Fe = O double bond of Cpd II species.^[^ ^6^, ^24^ ^]^ These results suggest that the hydrogen atom of carboxylic acid in **1‐COOH** is not transferred to the iron‐oxo moiety to form the Fe(IV)‐OH analogue, and a weak non‐covalent interaction between the H atom and oxygen atom is probably retained.^[^ ^20a^ ^]^

As reported by Nocera and co‐workers, the single X‐ray crystal structure of the ferric Hangman porphyrin precursor showed that the distal functional group, such as carboxylic acid attached on the xanthene scaffold was positioned to the metal center. This group has the potential to form an intramolecular H‐bonding interaction with the oxygen atom of the iron‐oxo species.^[^ ^19c^ ^]^ Unfortunately, we were unable to obtain the single crystal structure of reactive iron(IV)‐oxo species. To further confirm the presence of intramolecular H‐bonding, DFT calculations were used to optimize the structure of the Hangman iron(IV)‐oxo porphyrin species (Figure 1b). DFT calculations clearly showed that an intramolecular H‐bond with a length of 1.74 Å formed between the H atom of the carboxylic acid in **2‐COOH** and the oxygen atom of the iron(IV)‐oxo moiety. Alternatively, an intramolecular H‐bond with a length of 2.04 Å formed between the H atom of the amide group in **2‐CONH_2_** and the oxygen atom of the iron(IV)‐oxo moiety. In the case of **2‐COOMe**, no intramolecular H‐bonding formed. It is worth noting that the length of the H‐bond formed in **2‐COOH** was much shorter than that in **2‐CONH_2_**, indicating a much stronger intramolecular H‐bonding interaction in **2‐COOH**. This result is consistent with the stronger acidity of the carboxylic acid group compared with the amide group.^[^ ^14b^ ^]^ Moreover, the other N‐H moiety in **2‐CONH_2_** interacted with the oxygen of the xanthene scaffold on the porphyrin ligand to form another intramolecular H‐bonding, which pulled the amide group away from the Fe = O moiety and further elongated the Fe = O—H bond (Figure S36, Supporting Information). Similar results were also obtained for the electron‐deficient Hangman iron(IV)‐oxo porphyrin species (1.81 Å of **1‐COOH** versus 2.16 Å of **1‐CONH_2_**, Figure 1b). Based on the results of spectroscopic characterization and DFT calculations, we can assign **1‐COOH**, **1‐CONH_2_**, **2‐COOH**, and **2‐CONH_2_** as [Fe^IV^(O)(PFHPX‐COOH)], [Fe^IV^(O)(PFHPX‐CONH_2_)], [Fe^IV^(O)(MesHPX‐COOH)] and [Fe^IV^(O)(MesHPX‐CONH_2_)] respectively, in which the intramolecular H‐bonding is present, while **1‐COOMe** and **2‐COOMe** are assigned as [Fe^IV^(O)(PFHPX‐COOMe)] and [Fe^IV^(O)(MesHPX‐COOMe)] in which the intramolecular H‐bonding is absent.

### 2.2 Reactivity and Kinetic Studies of Iron(IV)‐Oxo Porphyrin Species in Various Oxidation Reactions

#### 2.2.1 Olefin Epoxidation Reactions

**Figure 3:**
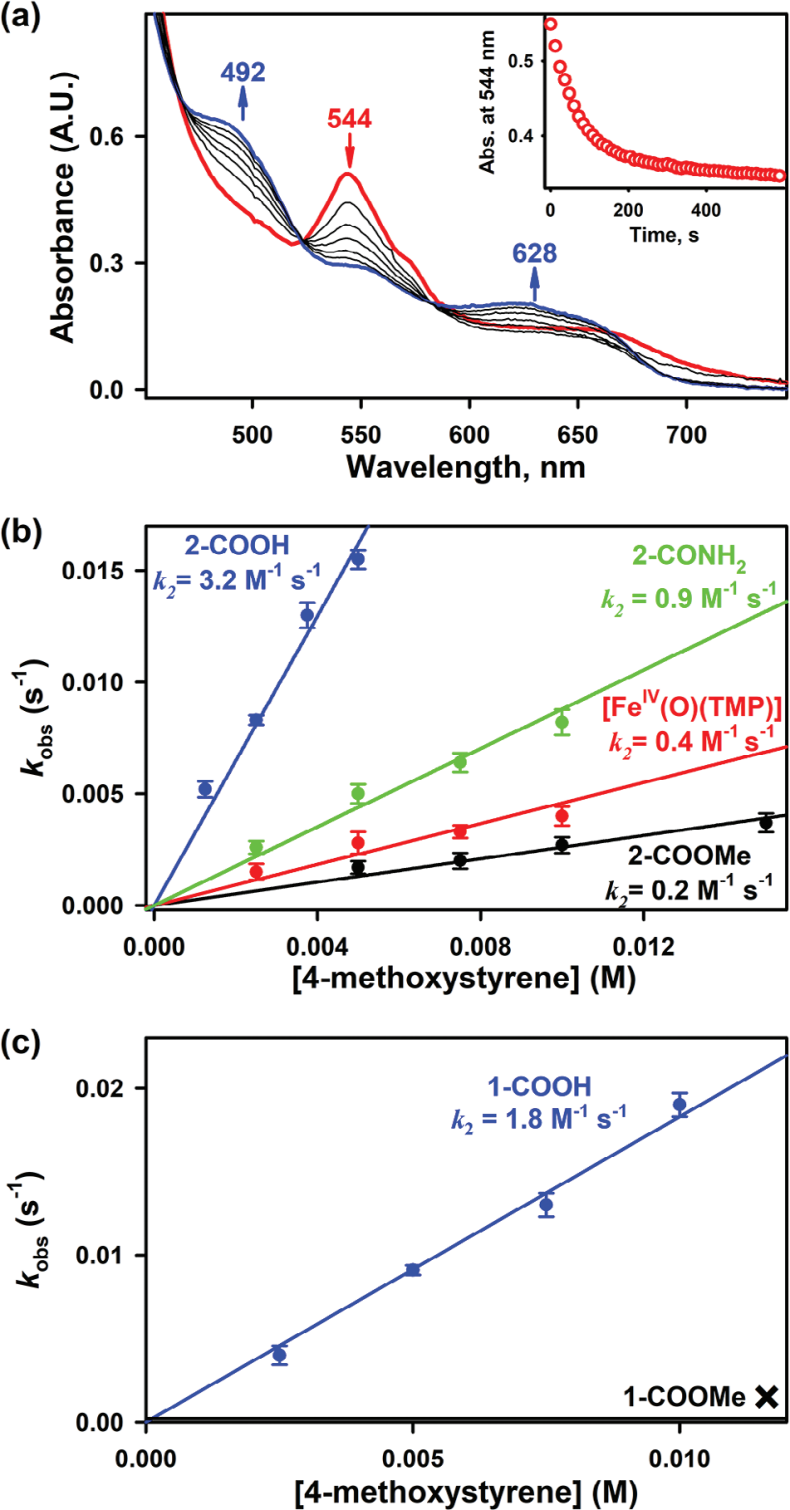
a) UV−vis spectral changes showing the reaction of **2‐COOH** (5.0 × 10^−2^ mm, red line) and 4‐methoxystyrene (5.0 mm) in 2.5 mL of CH_3_CN/CH_3_OH (v/v 100:1) at 258 K. The inset shows the time course monitored at 544 nm due to the decay of **2‐COOH**. b) Plots of the *k*_obs_ against the concentration of 4‐methoxystyrene to determine the second‐order rate constants in epoxidation by **2‐COOH** (5.0 × 10^−2^ mm, blue line), **2‐CONH_2_** (5.0 × 10^−2^ mm, green line), **2‐COOMe** (5.0 × 10^−2^ mm, black line) and [Fe^IV^(O)(TMP)] (5.0 × 10^−2^ mm, red line) at 258 K. (c) Plots of the *k*_obs_ against the concentration of 4‐methoxystyrene to determine the second‐order rate constants in epoxidation by **1‐COOH** (5.0 × 10^−2^ mm, blue line) and **1‐COOMe** (5.0 × 10^−2 ^mm, black line) at 258 K.

High‐valent iron‐oxo species are known to undergo OAT reactions.^[^ ^1^, ^2^, ^12^ ^]^ However, the H‐bonding effect on the reactivity of heme‐containing high‐valent iron‐oxo species in OAT reactions has not been performed yet. Thus, we investigated the reactivities of the Hangman iron(IV)‐oxo species in olefin epoxidation reactions. Because large spectral changes occurred in the Q‐band in UV‐vis spectroscopy, the investigated concentration of iron(IV)‐oxo porphyrin species was increased to 5.0 × 10^−2^ mm in the reactivity and kinetic studies to focus on the Q‐band spectral changes. Addition of 4‐methoxystyrene to the in‐situ generated **2‐COOH** at 258 K resulted in clean spectral changes back to the characteristic peaks of the starting iron(III) porphyrin complex within 10 min (**Figure** **3**a). The ESI‐MS of the final product exhibited a prominent ion peak at a mass‐to‐charge ratio (*m*/*z*) of 1082.5 and an isotopic distribution pattern corresponding to the mass of [Fe^III^(MesHPX‐COOH)]^+^. The EPR analysis of the final product showed the characteristic peaks of high‐spin iron (III) species at *g* = 6.1 and 2.2 respectively (SI, Figure S13, Supporting Information). Both of ESI‐MS and EPR results were identical with the spectra of [Fe^III^(MesHPX‐COOH)OH], indicating the final iron porphyrin product was the iron(III) porphyrin precursor. The formation of ferric porphyrin complexes as the final product was due to the oxidation of the initially formed ferrous porphyrin complexes by the remaining PhIO present in reaction solution. Similar results were also found in other Cpd II and Mn(IV)‐oxo porphyrin systems, in which the iron(III) and manganese(III) porphyrin complexes were the final products in OAT reactions.^[^ ^6^, ^26^ ^]^ Product analysis by gas chromatography showed that 2‐(4‐methoxyphenyl)oxirane was the sole organic product with the yield of 99% (Table S1, Supporting Information). Similarly, when other Hangman iron(IV)‐oxo species bearing electron‐rich substituents, such as **2‐CONH_2_** and **2‐COOMe**, were used, conversion of iron(IV)‐oxo to iron(III) species were observed in the reaction of 4‐methoxystyrene with different reaction rates (vide infra), yielding epoxide product (Table S1, Supporting Information).

In the case of the electron‐deficient Hangman iron(IV)‐oxo species, however, **1‐COOH** surprisingly reacted facilely with the olefin substrate. By contrast, **1‐COOMe**, which had no intramolecular H‐bonding, was not reactive under the same conditions (Figure 3c). These results clearly indicate that intramolecular H‐bonding will enhance the reactivity of iron(IV)‐oxo species in OAT reactions.

**Figure 4:**
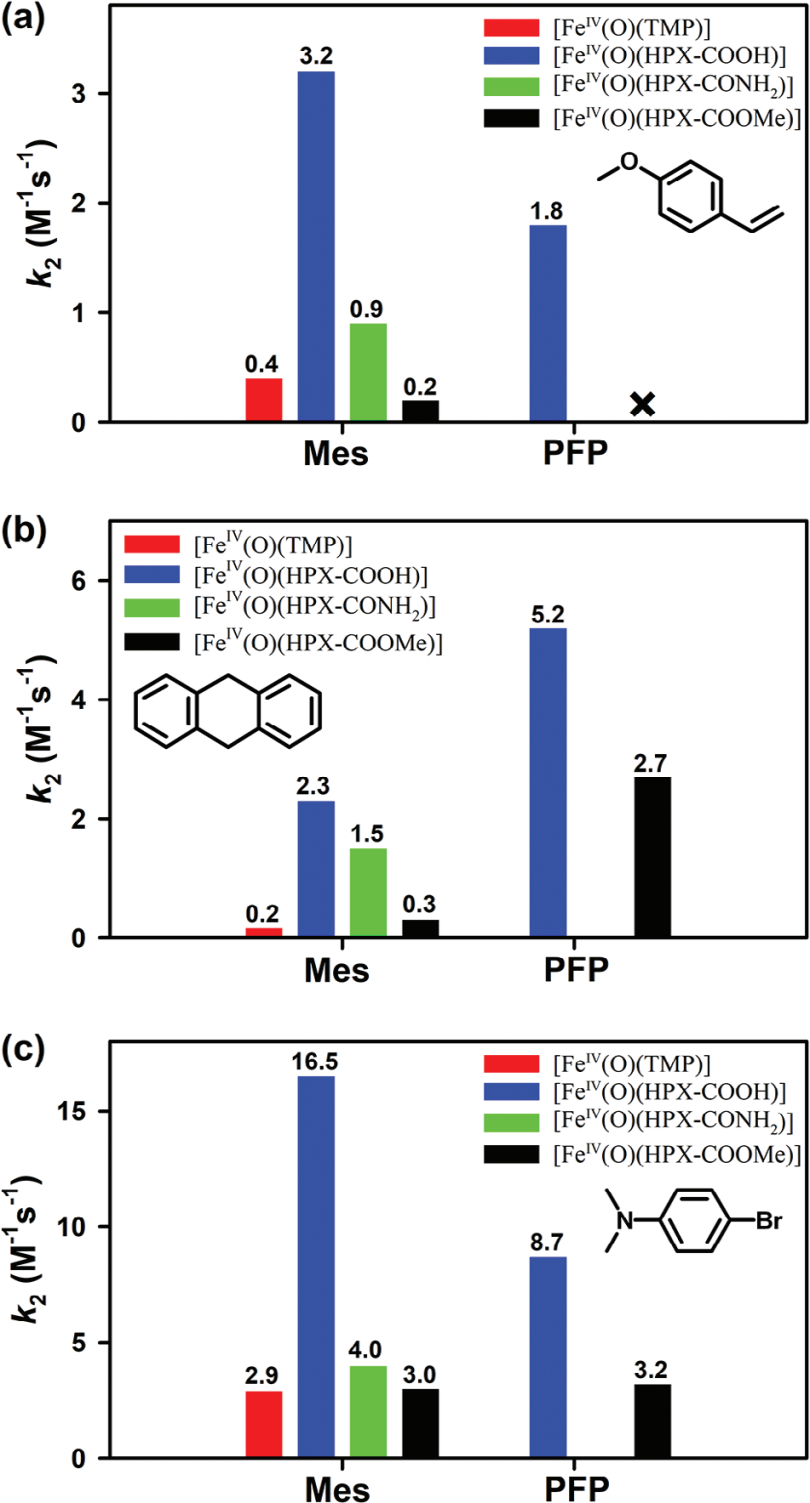
Column diagram of the second‐order rate constants *k*_2_ obtained in the reaction of iron(IV)‐oxo porphyrin species and a) 4‐methoxystyrene, b) 9,10‐dihydroanthracene, and c) 4‐bromo‐*N*,*N*‐dimethylaniline at 258 K. Mes and PFP refer to the iron(IV)‐oxo complexes bearing mesityl groups or pentafluorophenyl group respectively.

To quantify the H‐bonding effect on the reactivities of iron(IV)‐oxo species, kinetic studies were also performed. The first‐order rate constants, determined by pseudo‐first‐order fitting for the decay of iron(IV)‐oxo porphyrin species, increased linearly with increasing 4‐methoxystyrene concentration, giving second‐order rate constants (*k*_2_) of 3.2 m ^−1^ s^−1^ for **2‐COOH**, 0.9 m ^−1^ s^−1^ for **2‐CONH_2_** and 0.2 m ^−1^ s^−1^ for **2‐COOMe** (**Figure** 3b and **4**a). The obtained *k*_2_ values of iron(IV)‐oxo species with intramolecular H‐bonding were much larger than those without H‐bonding. Moreover, **2‐COOH**, which had stronger H‐bonding than **2‐CONH_2_**, also exhibited higher reactivity in OAT reactions. Further, for comparison, the well‐studied iron(IV)‐oxo species bearing a symmetrical porphyrin ligand, [Fe^IV^(O)(TMP)], was also used under identical conditions. The *k*_2_ value of [Fe^IV^(O)(TMP)] was close to the *k*_2_ of **2‐COOMe** (Figures 3b and 4a). For the electron‐deficient iron(IV)‐oxo species, introduction of H‐bonding interactions promoted the OAT reaction, affording *k*_2_ value of 1.8 M^−1^ s^−1^ for **1‐COOH**, which was comparable to that for **2‐COOH** (Figures 3c and 4a). No reaction occurred between **1‐COOMe** and 4‐methoxystyrene. The above results suggest that the different reactivities can be mainly attributed to intramolecular H‐bonding interaction under the reaction conditions.

When other olefin substrates, such as *cis*‐stilbene and 1,1‐diphenylethylene were used in the OAT reactions (Figures S14 and S15, Supporting Information), similar results were obtained. For example, the iron(IV)‐oxo species with intramolecular H‐bonding exhibited enhanced reactivities in OAT reactions. The stronger the H‐bonding, the higher the reactivity; Furthermore, the electron‐deficient iron(IV)‐oxo species **1‐COOMe** without H‐bonding was almost inactive toward olefin substrates. Introduction of H‐bonding could promote the OAT reaction. Taken together, the reactivity and kinetic studies demonstrated unambiguously that intramolecular H‐bonding is able to enhance the reactivity of heme‐containing iron(IV)‐oxo species in OAT reactions.

#### 2.2.2 C‐H Activation Reactions

Although previous studies have shown that intramolecular H‐bonding can influence the reactivity of tripodal non‐heme metal‐oxo species in C‐H activation reactions,^[^ ^20^, ^25^ ^]^ the effect of intramolecular H‐bonding on heme iron‐oxo species in C‐H activation reactions has not been studied. Therefore, we investigated the intramolecular H‐bonding interaction of the Hangman iron(IV)‐oxo porphyrin species in C‐H activation reactions. Similar to the OAT reactions, addition of hydrocarbons, such as 9,10‐dihydroanthracene (DHA), to the solution of **2‐COOH** resulted in the spectral conversion from the iron(IV)‐oxo species to its ferric precursor [Fe^III^(MesHPX)]^+^, accompanied by the formation of anthracene in the yield of 68% (Table S2, Supporting Information). For other Hangman iron(IV)‐oxo porphyrin species, both electron‐rich and ‐deficient, similar spectral changes and product yields were obtained.

Subsequent kinetic studies revealed that intramolecular H‐bonding with iron(IV)‐oxo porphyrin species could enhance the reactivity in C‐H activation reactions, as observed in OAT reactions. For example, the *k*_2_ values of **2‐COOH**, **2‐CONH_2_,** and **2‐COOMe** with DHA were determined to be 2.3 m ^–1^ s^–1^, 1.5 m ^–1^ s^–1^, and 0.3 M^–1^ s^–1^ at 258 K, respectively (**Table** **1**; Figure S16, Supporting Information). The iron(IV)‐oxo species installed with stronger H‐bonding showed higher reactivity in C‐H activation reactions. When other hydrocarbon substrates, such as xanthene, 1,4‐cylclohexadiene (CHD) and tetralin (THN), were used, the same trend was obtained (Figures S17–S19, Supporting Information). However, it should be noted that the intramolecular H‐bonding effect in C‐H activation reactions was not as remarkable as that found in OAT reactions. Nevertheless, it was clear that intramolecular H‐bonding could enhance the reactivity of iron(IV)‐oxo porphyrin species in C‐H activation reactions. This enhancement is consistent with the effect of intermolecular H‐bonding in heme systems,^[^ ^21a^ ^]^ but opposite to tripodal non‐heme metal‐oxo systems. In the latter case, the reactivities of metal‐oxo species are inhibited by intramolecular H‐bonding.^[^ ^20^, ^25^ ^]^ When comparing the tripodal non‐heme systems with the heme systems, differences in coordination strength and symmetry will result in completely different electronic configurations and frontier molecular orbitals (FMOs). Take $d_{x^{2}-y^{2}}$ orbital for example, this orbital is usually an anti‐bonding orbital in heme system, while in the tripodal systems, it is usually a non‐bonding orbital.^[^ ^4f^ ^]^ H‐bonding may interact with different FMOs in these two systems, resulting in an opposite effect.

**Table 1:** Second‐order rate constants *k*_2_ for OAT, C‐H activation and ET reactions by iron(IV)‐oxo porphyrin species.

Sub.^a)^	*k*_2_ [M^−1^ s^−1^]				
	1‐COOH	1‐COOMe	2‐COOH	2‐CONH_2_	2‐COOMe
1,1‐diphenylethylene	1.6	0.2	18.2	12.0	7.1
4‐methoxystyrene	1.8	N.R.^b)^	3.2	0.9	0.2
*cis*‐stilbene	0.4	N.R.^b)^	2.3	1.0	0.2
xanthene	25.9	9.0	7.1	3.5	1.5
DHA	5.2	2.7	2.3	1.5	0.3
CHD	2.9	1.7	1.6	1.4	0.8
THN	0.1	N.R.^b)^	23.7	1.0	0.5
DMA	22.5	3.6	162.1	8.9	6.5
4‐Br‐DMA	8.7	3.2	16.5	4.0	3.0

^a)^ The iron(IV)‐oxo porphyrin species (5.0 × 10^−2^ mm) was used in various oxidation reactions in CH_3_CN/CH_3_OH (v/v 100:1) at 258 K;

^b)^ N.R.: No reaction.

When deuterated substrate, such as *d*_2_‐xanthene was used in the reaction of **2‐COOH**, *k*_2_ value was determined to be 1.4 mol^−1^ s^−1^, affording the kinetic isotop effect (KIE) of 5.1 (Figure S22, Supporting Information). Similarly, the KIE values was determined to be 7.4 for **2‐COOMe** in the reaction of xanthene/*d*_2_‐xanthene (Figure S22, Supporting Information). These results indicated that the KIE was not significantly affected by the intramolecular H‐bonding in C‐H activation reactions.

#### 2.2.3 Electron Transfer Reactions

Electron transfer reaction is commonly used to probe the oxidative capability of high‐valent metal‐oxo species.^[^ ^26^ ^]^ By the addition of 4‐bromo‐*N*,*N*‐dimethylaniline (4‐Br‐DMA), iron(IV)‐oxo porphyrin species was one electron reduced to the relevant ferric porphyrin complex with much faster reaction rate compared with OAT and C‐H activation reactions, as indicated by UV‐vis spectroscopy (Figure S20, Supporting Information). We also found that intramolecular H‐bonding promoted the electron transfer reaction. As shown in Figure 4c and Table 1, determined *k*_2_ values of **2‐COOH** and **1‐COOH** in the reaction of 4‐Br‐DMA were much larger than those of the other species. This difference suggested that iron(IV)‐oxo porphyrin species with intramolecular H‐bonding were stronger oxidants. When other substrates such as *N*,*N*‐dimethylaniline (DMA) was used, the same trend was obtained (Figure S21, Supporting Information).

The *k*_2_ values obtained in the reactions of Hangman iron(IV)‐oxo porphyrin species and various substrates are summarized in Table 1. In conclusion, we demonstrated unambiguously that intramolecular H‐bonding with iron(IV)‐oxo porphyrin species enhanced its oxidative reactivity in OAT, C‐H activation and electron transfer reactions. This unified enhanced reactivities of high‐valent metal‐oxo complexes promoted by H‐bonding interaction in various oxidation reactions is first discovered in biomimetic system.

### 2.3 Mechanistic Studies of the Intramolecular H‐bonding Effect on Iron(IV)‐Oxo Porphyrin Species in Oxidation Reactions

#### 2.3.1 Determination of the Activation Parameters

**Figure 5:**
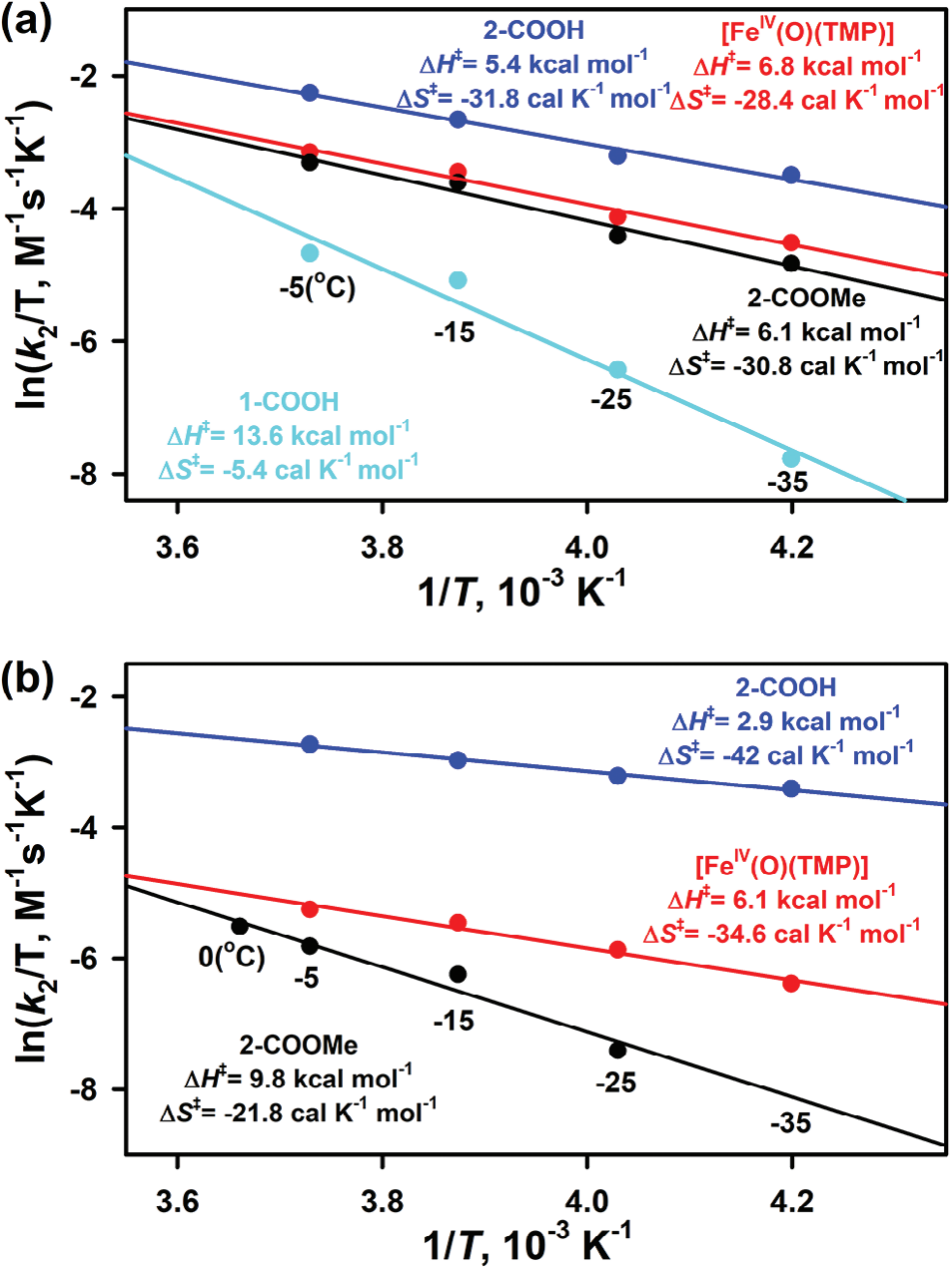
Eyring plots for a) the OAT reaction of 1,1‐diphenylethylene and b) the C−H activation of xanthene by **2‐COOH** (blue line), **2‐COOMe** (black line), [Fe^IV^(O)(TMP)] (red line) and **1‐COOH** (cyan line).

To elucidate the H‐bonding effect on iron(IV)‐oxo porphyrin species, kinetic studies were performed at different temperatures to determine the activation enthalpy (Δ*H* ^⧧^) and activation entropy (Δ*S* ^⧧^) of oxidation reactions from Eyring plots. As shown in **Figure** **5**, the Δ*H* ^⧧^ value for **2‐COOH** was the smallest one, compared with the Δ*H* ^⧧^ values for **2‐COOMe** or [Fe^IV^(O)(TMP)] in both the OAT and C‐H activation reactions. The lower activation enthalpy of **2‐COOH** was expected because of the higher oxidizing power as observed in electron transfer reactions, and further confirmed that intramolecular H‐bonding could enhance the reactivity of iron(IV)‐oxo porphyrin species in oxidation reactions.^[^ ^26a^ ^]^

For the activation entropy, the Δ*S* ^⧧^ value of **2‐COOH** was the most negative in both OAT and C‐H activation reactions. This result can be explained by the “enthalpy‐entropy compensation effect” (EECE), which was recently reported in biomimetic systems.^[^ ^26a^ ^]^ In general, changes of Δ*H* ^⧧^ and Δ*S* ^⧧^ are often linearly related.^[^ ^27^ ^]^ Consequently, the larger Δ*H* ^⧧^ will afford less negative Δ*S* ^⧧^ to “compensate” the lager Δ*H* ^⧧^, where the overall Δ*G* ^⧧^ can be small enough for the reaction taking place, according to the Gibbs free energy Equation (1).

(1) $$\Delta G^{‡}=\Delta H^{‡}-T\Delta S^{‡}$$

For example, the Δ*H* ^⧧^ of **2‐COOMe** in the reaction of xanthene was 9.8 kcal mol^−1^, which was much larger than that of **2‐COOH** (2.9 kcal mol^−1^). However, the difference of Δ*G* ^‡^ between **2‐COOMe** and **2‐COOH** in the reaction of xanthene was only 1.7 kcal mol^−1^ at 258 K (15.4 vs 13.7 kcal mol^−1^), which was smaller than the difference of Δ*H* ^⧧^, because the less negative Δ*S* ^⧧^ of **2‐COOMe** compensated the larger Δ*H* ^⧧^. The larger Δ*G* ^‡^ of **2‐COOMe** compared with **2‐COOH** was consistent with the smaller *k*_2_ values obtained in kinetic studies (Table 1).

In OAT reactions, **2‐COOH** was more reactive than **1‐COOH** (Table 1). For example, *k*_2_ of **2‐COOH** in the reaction of 1,1‐diphenylethylene was 18.6 mol^−1^ s^−1^, which was much larger than that of **1‐COOH** (1.6 mol^−1^ s^−1^). This result was similar to Mn(IV)‐oxo porphyrin system but opposed to Cpd I system.^[^ ^2^, ^26^ ^]^ The higher reactivity of **2‐COOH** bearing electron‐rich porphyrin ligand could also be ascribed to EECE. Eyring plot of **2‐COOH** and **1‐COOH** in the reaction of 1,1‐diphenylethylene will cross at 307K (Figure 5a), which is the compensation temperature (*T*_comp_). Below the *T*_comp_, Δ*H* ^⧧^ makes the major contribution of Δ*G* ^⧧^. Since Δ*H* ^⧧^ of **2‐COOH** was much smaller (5.4 vs 13.6 kcal mol^−1^), the overall Δ*G* ^⧧^ of **2‐COOH** was smaller, resulting in higher reactivity. Above the *T*_comp_, *T*Δ*S* ^⧧^ makes the major contribution of Δ*G* ^⧧^. Since Δ*S* ^⧧^ of **2‐COOH** was much more negative (−31.8 vs −5.4 cal K^−1^ mol^−1^), the overall Δ*G* ^⧧^ of **2‐COOH** was higher, leading to lower reactivity. However, the *T*_comp_ of 307 K is too high for Cpd II species for kinetic studies, which will be decomposed very quickly. Therefore, **2‐COOH** bearing electron‐rich porphyrin ligand exhibited higher reactivity in OAT reactions than **1‐COOH** bearing electron‐deficient porphyrin ligand at investigated temperatures.

As stated by Starikov, such as “still a lot of difficulties, in particular, those with conclusively elucidating the entropy notion's exact meaning”,^[^ ^28^ ^]^ the inherent character of the activation entropy is difficult to elucidate, because Δ*S* ^⧧^ cannot be directly measured. Nevertheless, to some extent, the activation entropy reflects the structural changes from the ground state to the transition state during the reaction.^[^ ^29^ ^]^ The most negative Δ*S* ^⧧^ of **2‐COOH** indicates that formation of the transition state requires more severe changes in the geometry and configuration of **2‐COOH** in oxidation reactions. We propose that the unique intramolecular H‐bonding interaction with the Fe = O moiety in **2‐COOH** may cause more significant structural changes to achieve the transition state in H‐atom abstraction by iron(IV)‐oxo species in C‐H activation reactions, or the C‐O bond formation in OAT reactions.

#### 2.3.2 Theoretical Calculations

As mentioned above, we have confirmed through kinetic studies that heme compounds with stronger intramolecular H‐bonding exhibited higher reactivity. Further Eyring plots showed that compounds with stronger intramolecular H‐bonding had lower enthalpy changes, which agreed with the results of the kinetic studies. Borovik, Que, and others have reported that H‐bonding greatly influences the reactivity of high‐valent iron‐oxo species,^[^ ^30^ ^]^ especially for the C‐H activation reaction. In these studies, theoretical calculations revealed the existence of a significant effect of H‐bonding on the structure and reactivity of the iron‐oxo complexes, and a rational mechanism was proposed on the basis of this discovery. However, few theoretical calculations have been performed to study the H‐bonding effect on the OAT reaction. Furthermore, integration with experimental results is required. Moreover, the unified enhanced reactivities promoted by H‐bonding, which has been revealed for the first time in this work, need to be elucidated in more details. Hence, to clarify the exact effect of intramolecular H‐bonding on the properties of the Cpd II intermediate, DFT calculations were carried out to investigate the OAT and C‐H activation reactions. All calculations were carried out using the Gaussian 16 soft‐ware package at B3LYP‐D3(BJ)/6‐31G(d)‐LANL2DZ level of theory.^[^ ^31^ ^]^

**Figure 6:**
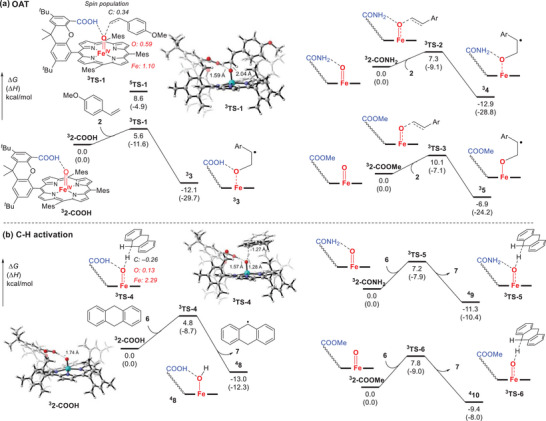
Free energy profiles of different iron (IV)‐oxo porphyrin species in the a) OAT reactions and b) C‐H activation reactions. ∆*G*, change in Gibbs free energy; ∆*H*, change in enthalpy.

As shown in **Figure** **6**a, the OAT reaction between **2‐COOH** and substrate 4‐methoxystyrene can occur easily through a triplet transition state **^3^TS‐1** (Δ*G* ^‡^ = 5.6 kcal mol^−1^), leading to the formation of the epoxide coordinated iron(II) complex **3**.^[^ ^5a^ ^]^ The corresponding quintet transition state **^5^TS‐1** has a higher activation free energy (Δ*G* ^‡^ = 8.6 kcal mol^−1^), indicating that the OAT preferentially occurs on the triplet potential energy surface. The OAT process proceeds without the formation of a typical benzyl radical intermediate. Instead, it forms an intermediate with spin distributed on both the benzyl carbon and oxygen atom (Figure S38, Supporting Information). This conclusion also applies to the analogue **2‐CONH_2_** species in OAT reactions. **2‐CONH_2_** went through **^3^TS‐2** (Δ*G* ^‡^ = 7.3 kcal mol^−1^) directly to accomplish the epoxidation. The energy barrier is higher due to the weaker intramolecular H‐bonding in **2‐CONH_2_**. For **2‐COOMe**, the highest energy barrier is observed for OAT transition state **^3^TS‐3** (Δ*G* ^‡^ = 10.1 kcal mol^−1^) when intramolecular H‐bonding is absent (Figure S37, Supporting Information). A stable benzyl radical intermediate **^3^5** is also located in this case. The potential energy surface shows that **2‐COOH** undergoes a one‐step two‐electron OAT reaction to directly generate the epoxidation product. This is similar to the mechanism of epoxidation reactions catalyzed by high‐valent metal‐oxo species reported by Fujii and co‐workers.^[^ ^5a^ ^]^

The computational results reveal that intramolecular H‐bonding can greatly decrease the energy barrier of the OAT reactions, affording faster reaction rate as observed in the kinetic studies. Similarly, the intramolecular H‐bonding interaction decreases the C‐H activation energy barrier. As shown in Figure 6b, 2**‐COOH** and the substrate DHA **6** go through a triplet transition state **^3^TS‐4** (Δ*G* ^‡^ = 4.8 kcal mol^−1^), which is kinetically favored over the quintet **^5^TS‐4** (SI, Figure S37, Supporting Information) This generates the radical product **7** and Fe‐OH intermediate **8**. **2‐CONH_2_** and **2‐COOMe** go through similar triplet transition states **^3^TS‐5** (Δ*G* ^‡^ = 7.2 kcal mol^−1^) and **^3^TS‐6** (Δ*G* ^‡^ = 7.8 kcal mol^−1^) to form an Fe‐OH intermediate. **2‐COOMe** in C‐H activation reaction requires the highest activation free energy, where the intramolecular H‐bonding is absent. It should be noted that the difference in the energy barrier in the OAT reactions is larger than that in the C‐H activation reactions, implying that intramolecular H‐bonding has a greater effect on the OAT reaction. As a result, larger energy barrier difference in the OAT reaction leads to larger difference of *k*_2_ values in the kinetic studies, compared with the C‐H activation reactions. For example, the energy barrier difference between **2‐COOH** and **2‐COOMe** in the reaction of 4‐methoxystyrene is 4.5 kcal mol^−1^ (5.6 vs 10.1 kcal mol^−1^ in Figure 6a), while the energy difference in the reaction of DHA is 3.0 kcal mol^−1^ (4.8 vs 7.8 kcal mol^−1^ in Figure 6b). In accordance, the difference of *k*_2_ values in the reaction of 4‐methoxystyrene is 16 times (3.2 vs 0.2 m ^–1^ s^–1^, Table 1), while the difference of *k*_2_ values in the reaction of DHA is about 7 times (2.3 vs 0.3 m ^–1^ s^–1^, Table 1). We also investigated the bond dissociation free energy (BDFE) of O‐H bonds in the intermediates **8**, **9**, and **10** by theoretical calculations, which were determined to be 85.5 kcal mol^−1^, 83.8 kcal mol^−1^ and 81.9 kcal mol^−1^, respectively (Table S4, Supporting Information). The data indicates that the corresponding Fe‐OH intermediates' O‐H bond BDFEs are increased by increasing the strength of intramolecular H‐bonding.

This suggests that Fe‐ OH intermediate in the C‐H activation reactions may be stabilized by the intramolecular H‐bonding.

**Figure 7:**
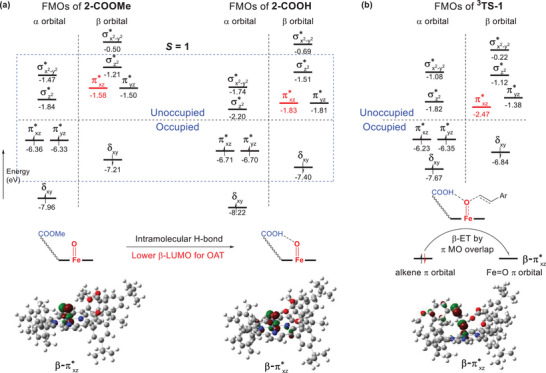
Energy diagrams and FMOs of a) **2‐COOMe**, **2‐COOH** and b) OAT transition state **^3^TS‐1**.

To determine how the electron transfer of **2‐COOH** occurs in OAT reaction, frontier molecular orbital (FMO) analysis was performed for the reactions with and without intramolecular H‐bonding. The energy diagram and 3D surfaces of relevant high‐lying occupied and low‐lying virtual orbitals of **2‐COOMe**, **2‐COOH** and the OAT transition state **^3^TS‐1** are shown in **Figure** **7**. It should be noted that the diagrams were simplified by omitting orbitals that are of little consequence to the Fe‐O species’ reactivity, which means molecular orbitals presented in Figure 7 are mainly composed of metal d orbitals and oxygen p orbitals. Those orbitals are labeled according to the type of d orbitals that contribute to it. In Figure 7a, the lowest energy of both the *α* and *β* orbitals are the non‐bonding orbital δ_*xy*_, which is basically composed of metal d_*xy*_. This orbital is oriented toward the angular bisector of the xy plane, which prevents it from interacting with ligands in the octahedral geometry. Above δ_*xy*_ are *π* anti‐bonding orbitals, $\pi_{xz}^{*}\;$ and $\pi_{yz}^{*}$, formed through the interaction of d_*xz*_ and d_*yz*_ with the p_*x*_ and p_*y*_ orbitals of oxygen. Unlike the fully occupied δ_*xy*_ orbital, both the $\pi_{xz}^{*}$ and $\pi_{yz}^{*}$ orbitals are only presented on the *α* manifold, which makes the *α*‐π* energy much lower than that for their *β* counterpart. With the two unpaired *α* electrons in $\;\pi_{xz}^{*}\;$ and $\pi_{yz}^{*}$, the calculated positive spin are respectively located at the iron and oxygen atoms, leaving the remaining orbitals unoccupied. The non‐degenerate nature of $\pi_{xz}^{*}\;$ and $\pi_{yz}^{*}$, results from the HPX side chain. The following two *π* anti‐bonding orbitals are the *σ* anti‐bonding $\sigma_{z^{2}}^{*}$ orbital and $\sigma_{x^{2}-y^{2}}^{*}$ orbital, which are formed through the interaction of $d_{z^{2}}^{*}$ with oxygen p_*z*_ and $d_{x^{2}-y^{2}}^{*}$ with the porphyrin ring.

Comparison between the FMOs of **2‐COOMe** and **2‐COOH** indicates that the presence of intramolecular H‐bonding significantly reduces the energy of all molecular orbitals, especially the $\pi_{xz}^{*}$ orbital (red in Figure 7a, −1.83 eV vs −1.58 eV) that spatially overlaps with the H‐bonding. When substrate 4‐methoxystyrene reacts with **2‐COOH**, the electron transfer from the *π* bonding orbital to the Fe = O anti‐bonding *β*‐$\pi_{xz}^{*}$ orbital through *π*‐style molecular orbital overlap is observed in the FMO of **^3^TS‐1** (Figure 7b). The lower orbital energy of *β*‐$\pi_{xz}^{*}$ resulting from the intramolecular H‐bonding can promote the electron transfer, which reduces the energy barrier of the OAT reaction and increases the reactivity of the iron(IV)‐oxo porphyrin species.

Like the OAT reaction, FMO analysis of the C‐H activation reaction suggests that the presence of intramolecular H‐bonding can also reduce the energy of the anti‐bonding $\sigma_{z^{2}}^{*}$ orbital. As shown in Figure S39 (Supporting Information), the $\sigma_{z^{2}}^{*}\;$ orbital interacts with the substrate C‐H *σ* bond in the transition state, which corresponds to the *α* electron transfer through *σ*‐style molecular orbital overlap.

As mentioned in the introduction section, several studies have investigated the reactivities of high‐valent metal‐oxo complexes with secondary sphere H‐bonding interactions, such as Borovik's [Fe^III^H_3_buea(O)]^2−^ or the [Mn^III^H_3_buea(O)]^2−^ system and Karlin's work on intermolecular H‐bonding in Fe(IV)‐oxo porphyrin species.^[^ ^20^, ^21^, ^25^ ^]^ Although no experimental studies were performed for OAT reactions in these studies, theoretical calculations by de Visser et al. comparing the bond dissociation energies of the product O‐H bonds showed that H‐bonding in Borovik's tripodal system could reduce the reactivity of the OAT reaction.^[^ ^18^ ^]^ However, in addition to the coordination structure of the studied pentacoordinate compounds differing from that of the Hangman iron(IV)‐oxo, the close coordination of the porphyrin further leads to more distinctions in the electronic configuration of the two compounds. These differences resulting in a totally different effect of H‐bonding in the heme system.

Recently, Kojima and his co‐workers reported the effect of H‐bonding on the reactivity of high‐valent Ru = O compounds.^[^ ^22^ ^]^ They found that the reactivity of non‐heme Ru = O compounds with phenolic substrates was reduced under the influence of intramolecular H‐bonding. The authors attributed this phenomenon to H‐bonding that decreased the basicity of the oxygen atom and made it more difficult for Ru = O compounds to grab the proton in the O‐H bond and slow down proton coupled electron transfer's rate‐determining step proton transfer.

They also found that intramolecular H‐bonding increased the electrophilicity of the intermediate by weakening the metal‐oxygen bonding, thereby increasing the rate of the OAT reaction with several substrates. Further DFT calculations give a quantitative explanation that intramolecular H‐bonding improves the oxidizing ability of the intermediate by lowering the energy of the lowest unoccupied molecular orbitals that accepts electrons. This conclusion is very similar to our results for the heme‐Fe system.

**Figure 8:**
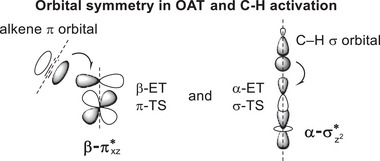
Illustration of the orbital symmetry in OAT and C‐H activation reactions.

Interestingly, Shaik and co‐workers found that H‐bonding resulted in a hybrid nature of compound I, which affected the FMOs and spin distribution.^[^ ^32^ ^]^ This result suggests that although H‐bonding is a non‐covalent interaction, its strong covalency allows for further hybridization of the FMOs, which is consistent with our findings. Our calculations reveal that hydrogen bonding lowers the energies of the anti‐bonding orbitals that will accept electrons, and the stronger H‐bonding interaction will result in more covalent hybridization. This accounts for the lower energies of the FMOs. Moreover, the illustration of orbital symmetry (**Figure** **8**) shows that the alkene *π* orbital prefers to overlap with Fe = O $\pi_{xz}^{*}$ orbital in OAT reaction because both have similar symmetry planes, which allows for strong orbital interactions. While in C‐H activation reaction, the σ orbital of the C–H bond shares the same symmetry axis as the Fe = O $\sigma_{z^{2}}^{*}$ orbital. The large orbital overlap plus the lower orbital energies of Fe = O $\pi_{xz}^{*}$ and $\sigma_{z^{2}}^{*}$ orbitals make the electron transfer easier. Therefore, it can be concluded that the unified enhanced reactivity of iron(IV)‐oxo porphyrin species, which is promoted by intramolecular H‐bonding in both OAT and C‐H activation reactions, is a result of the influence of intramolecular H‐bonding on the molecular orbital of iron(IV)‐oxo porphyrin species. The energies of all anti‐bonding orbitals of iron(IV)‐oxo species will be lowered because of the intramolecular H‐bonding interaction. This will facilitate the electron transfer from the substrate to the iron(IV)‐oxo porphyrin species, affording higher reactivities in all investigated oxidation reactions.

## 3 Conclusion

In summary, we have synthesized a series of iron(IV)‐oxo porphyrin species with or without intramolecular H‐bonding moiety. Their structures have been confirmed by various spectroscopies, such as UV‐vis, ESI‐MS, EPR, and rRaman. Kinetic studies revealed unified enhanced reactivity of iron(IV)‐oxo porphyrin species promoted by intramolecular H‐bonding interaction in OAT, C‐H activation, and ET reactions. The stronger the intramolecular H‐bonding interaction, the higher reactivity of the iron(IV)‐oxo porphyrin.

Theoretical studies showed that intramolecular H‐bonding stabilized the FMOs of the iron(IV)‐oxo species and reduced the energy of anti‐bonding orbitals during the oxidation reaction process. Therefore, the electron transfer from the substrate to the iron(IV)‐oxo porphyrin species enhanced in all investigated oxidation reactions because of the intramolecular H‐bonding interaction. This resulted in enhanced reactivities in kinetic studies. To the best of our knowledge, this is the first extensive investigation of the intramolecular H‐bonding effect on heme iron(IV)‐oxo species in various oxidation reactions. Moreover, our work presents the general effect of H‐bonding in different types of oxidation reactions. We believe that this work is valuable and informative for understanding the mechanisms of the “secondary coordination sphere” in both biological and biomimetic systems. Further examples of the “secondary coordination sphere” interaction with high‐valent metal‐oxo complexes and related mechanistic studies are under investigation in our laboratory.

## Conflict of Interest

The authors declare no conflict of interest.

## Supporting information

Supporting Information

## Data Availability

The data that support the findings of this study are available in the supplementary material of this article.
