# Supplementary material for: Enhanced Reactivities of Iron(IV)‐Oxo Porphyrin Species in Oxidation Reactions Promoted by Intramolecular Hydrogen‐Bonding
Source: Adv Sci (Weinh). 2024 Mar 13;11(19):2310333. doi: 10.1002/advs.202310333 (PMC11109629; doi:10.1002/advs.202310333)
Supplement: Supplementary file 1 — Supporting Information [file ADVS-11-2310333-s001.pdf]

ADVANCED SCIENCE
Open Access

Supporting Information

for *Adv. Sci.*, DOI 10.1002/advs.202310333

Enhanced Reactivities of Iron(IV)-Oxo Porphyrin Species in Oxidation Reactions Promoted by Intramolecular Hydrogen-Bonding

Zhe Gong, Liwei Wang, Yiran Xu, Duanfeng Xie, Xiaotian Qi, Wonwoo Nam* and Mian Guo**

Supporting Information

Enhanced Reactivities of Iron(IV)-Oxo Porphyrin Species in Oxidation Reactions Promoted by Intramolecular Hydrogen-Bonding

Zhe Gong,[a],# Liwei Wang,[a],# Yiran Xu,[a],# Duanfeng Xie,[a] Xiaotian Qi,*[a] Wonwoo Nam,*[b] and Mian Guo*[a]

[a]College of Chemistry and Molecular Sciences, Wuhan University, Wuhan 430072, PR China

[b]Department of Chemistry and Nano Science, Ewha Womans University, Seoul 03760, Korea

#These authors contributed equally

* E-mail: qi7xiaotian@whu.edu.cn, wwnam@ewha.ac.kr, whguomian@whu.edu.cn.

Table of Contents

Materials 4
Instrumentation 4
Generation of Fe^{IV}-Oxo Porphyrin Species 4
Kinetic Measurements 5
Product Analysis 5
General Procedure 6
Scheme S1 6
Scheme S2 7
Scheme S3 8
Scheme S4 9
Scheme S5 9
Scheme S6 10
Table S1 11
Table S2 12
Table S3 13
Table S4 14
Figure S1 15
Figure S2 16
Figure S3 17
Figure S4 18
Figure S5 19
Figure S6 20
Figure S7 21
Figure S8 22
Figure S9 23
Figure S10 24
Figure S11 25
Figure S12 26

Figure S13 27

Figure S14 28

Figure S15 29

Figure S16 30

Figure S17 31

Figure S18 32

Figure S19 33

Figure S20 34

Figure S21 35

Figure S22 36

Figure S23 37

Figure S24 38

Figure S25 39

Figure S26 40

Figure S27 41

Figure S28 42

Figure S29 43

Figure S30 44

Figure S31 45

Figure S32 46

Figure S33 47

Figure S34 48

Computational Details 50

Figure S36 50

Free Energy Profile 51

Figure S37 51

Figure S38 51

Energy Diagram 52

Figure S39 52

Cartesian Coordinates and Energies of Optimized Structures 54

References 75

Experimental Section

Materials. Commercially available chemicals were used without further purification unless otherwise indicated. Solvents were dried according to the literature procedures and redistilled under an argon atmosphere before use.[S1] Iodosylbenzene (PhIO) was synthesized by a literature method.[S2] [Fe^{III}(PFHPX-COOH)(OH)], [Fe^{III}(PFHPX-$CONH_2$)(OH)], [Fe^{III}(PFHPX-COOMe)(OH)], [Fe^{III}(MesHPX-COOH)(OH)], [Fe^{III}(MesHPX-$CONH_2$)(OH)], [Fe^{III}(MesHPX-COOMe)(OH)] and [Fe^{III}(TMP)(OH)] were prepared using established literature protocol.[S3, S4] $H_2{}^{18}O$ (98% ^{18}O-enriched) was purchased from Meryer.

Instrumentation. UV-vis spectra were recorded on a Hewlett Packard Agilent Cary 60 UV-vis spectrophotometer equipped with an UNISOKU cryostat system (USP-203; UNISOKU, Japan). Electrospray ionization mass (ESI-MS) spectra were collected on a LTQ Fleet Ion Trap Mass Spectrometer (Thermo Fisher Scientific, Waltham, MA USA), by infusing samples directly into the source using a sample syringe. The spray voltage was set at 2.5 kV and the capillary temperature at 120 °C. Resonance Raman (rRaman) spectra were collected with the UniDRON (UniNanoTech, Korea) Microscope Raman chamber with 20x long working objective lens and SR500i-A Raman spectrometer in combination with the DU420A-BU2 (ANDOR, UK) TE cooled CCD detector, cooled to 233 K. The LD laser (442nm Diode laser set, CNI laser, China) beam at the wavelength of 442 nm was used as the light source. The laser beam was focused using an objective lens with a magnifying power of 10. The frozen sample solution in NMR tube was put into a QUARTZ Large 150 mL Dewar Flasks (inner diameter 5.4 mm, outer diameter 10.5 mm, CortecNet, France) filled by liquid N_2, and the data acquisition time was normally 10 s and accumulated for 12 times. The wavenumber of the Raman spectra measured in this work was calibrated by using well-known Raman peaks of an acetone-d_6: CH_3CN=1:1 solution. X-band electron paramagnetic resonance (EPR) spectra was recorded at 90 K on Bruker E500 operated at 9.410 GHz. Typical spectrometer parameters are shown as follows, scan range: 4000 G; center field set: 2500 G; time constant: 0.00 ms; scan time: 5.24 s; modulation amplitude: 1.00 G; modulation frequency: 100.00 kHz; receiver gain: 2×10^4; microwave power: 0.02 mW. Product analysis was performed on an Agilent GC-8890 chromatograph using a J&W HP-5 column (30 m × 0.32 mm i.d.; 0.25 μm film thickness) with FID detector. The product was identified by comparison to the GC retention time of the authentic compound.

Generation of Fe^{IV}-Oxo Porphyrin Species. [Fe^{IV}(O)(PFHPX-COOH)] (**1-COOH**) was generated by adding PhIO (1.25×10^{-1} mM) into a 1 cm UV-vis cuvette containing a acetonitrile/CH_3OH (v/v 100:1) solution of [Fe^{III}(PFHPX-COOH)OH] (5.0×10^{-2} mM) at 258 K. Formation of **1-COOH** was confirmed by monitoring UV-vis spectral changes for the formation of absorption bands at 547 nm due to **1-COOH**, accompanied by the decay of absorption bands at 470

and 580 nm due to [Fe^{III}(PFHPX-COOH)OH]. [Fe^{IV}(O)(PFHPX-COOMe)] (**1-COOMe**) was synthesized by reacting [Fe^{III}(PFHPX-COOMe)OH] with 2.5 equiv of PhIO in acetonitrile/CH_3OH (v/v 100:1) at 258 K. [Fe^{IV}(O)(MesHPX-COOH)] (**2-COOH**), [Fe^{IV}(O)(MesHPX-$CONH_2$)] (**2-CONH₂**), [Fe^{IV}(O)(MesHPX-COOMe)] (**2-COOMe**) and [Fe^{IV}(O)(TMP)], were synthesized by reacting [Fe^{III}(MesHPX-COOH)OH], [Fe^{III}(MesHPX-$CONH_2$)OH], [Fe^{III}(MesHPX-COOMe)OH] and [Fe^{III}(TMP)OH] with 2.5 equiv of PhIO in acetonitrile/CH_3OH (v/v 100:1) at 258 K. All reactions were performed under air atmosphere using dried solvent unless otherwise noted. Samples for resonance Raman were prepared as follows: [Fe^{IV}(^{16}O)(PFHPX-COOH)] (**1-COOH-^{16}O**) was prepared by reacting [Fe^{III}(PFHPX-COOH)OH] (1.0 mM) with 5 equiv of PhIO (5.0 mM) in acetone-d_6: CH_3CN=1:1 (0.4 mL) at 233 K for 2 minutes, whereas [Fe^{IV}(^{18}O)(PFHPX-COOH)] (**1-COOH-^{18}O**) was prepared by reacting [Fe^{III}(PFHPX-COOH)OH] (1.0 mM) with 5 equiv of PhIO (5.0 mM) in the presence of $H_2{}^{18}O$ (7 μL) in acetone-d_6: CH_3CN=1:1 (0.4 mL) at 233 K for 2 minutes. [Fe^{IV}(O)(PFHPX-COOMe)] complexes (**1-COOMe-^{16}O** and **1-COOMe-^{18}O**), [Fe^{IV}(O)(MesHPX-COOH)] complexes (**2-COOH-^{16}O** and **2-COOH-^{18}O**), [Fe^{IV}(O)(MesHPX-$CONH_2$)] complexes (**2-CONH₂-^{16}O** and **2-CONH₂-^{18}O**) and [Fe^{IV}(O)(MesHPX-COOMe)] complexes (**2-COOMe-^{16}O** and **2-COOMe-^{18}O**), were prepared from [Fe^{III}(PFHPX-COOMe)OH], [Fe^{III}(MesHPX-COOH)OH], [Fe^{III}(MesHPX-$CONH_2$)OH] and [Fe^{III}(MesHPX-COOMe)OH] in acetone-d_6: CH_3CN=1:1 (0.4 mL) at 233 K by the same procedure for **1-COOH**.[S6]

Kinetic Measurements. Reactions of **1-COOH** with substrates, such as 4-methoxystyrene, *cis*-stibene, 1,1-diphnylethylene, xanthene, 9,10-dihydroanthracene (DHA), 1,4-cyclohexadiene (CHD), tetralin (THN), 4-bromo-*N*, *N*-dimethylaniline (4-Br-DMA), *N*, *N*-dimethylaniline (DMA) were performed by adding substrates into a 1 cm UV-vis cuvette containing a CH_3CN/CH_3OH (v/v 100:1) solution of Fe^{IV}-oxo porphyrin species (5×10^{-2} mM) at 258 K. The kinetic studies were followed by monitoring the change of the absorption bands at 545-550 nm due to the decay of Fe^{IV}-oxo porphyrin species. The kinetic experiments were run at least in triplicate, and the data reported here represented the average of these reactions. Rate constants were determined under pseudo-first-order conditions (i.e., [substrate]/[Fe^{IV}-oxo porphyrin species] > 10). The k_2 values for the oxidation of 1,1-diphenylethylene and xanthene by **2-COOH**, **2-COOMe** and [Fe^{IV}(O)(TMP)] were obtained at various temperatures (i.e., 0 ~ –35 °C with interval of 5 or 10 degree) to determine the activation parameters.

Product Analysis. Product analysis was performed by GC. Before the reaction solution was analyzed, internal standard (dodecane) was added to the solution. The reaction solution was filtered through a basic silica gel plug and analyzed by GC. Products were identified by comparing with authentic samples, and product yields were determined by comparing peak areas with that of

dodecane as an internal standard.

General Procedure

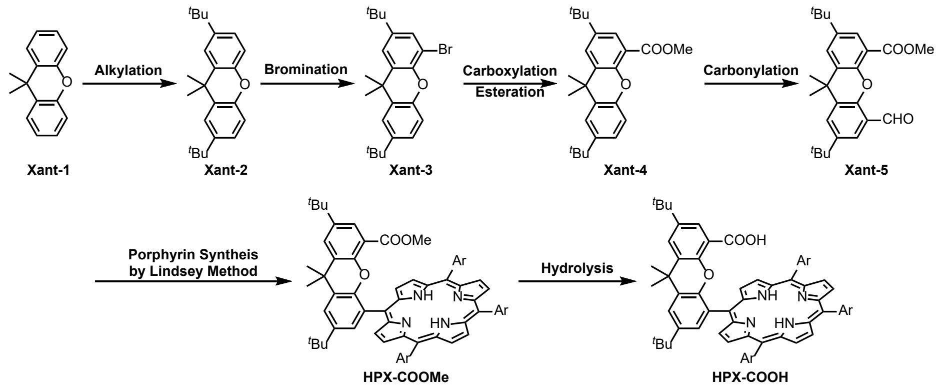

Scheme S1. Synthetic Routine of Hangman Porphyrin

Xanthene synthons, HPX-COOMe, and [Fe(TMP)OH] were synthesized according to previously reported procedure.[S3-S5]

5-[4-(5-Methoxycarbonyl-2,7-di-*tert*-butyl-9,9-dimethylxanthenyl)]-10,15,20-trimesitylporphyrin, H_2(MesHPX-COOMe).

^{1}H NMR (400 MHz, Chloroform-*d*) δ 8.74 (d, *J* = 4.8 Hz, 2H), 8.63 (s, 6H), 7.87 – 7.83 (m, 2H), 7.64 (d, *J* = 2.5 Hz, 1H), 7.32 (d, *J* = 1.7 Hz, 1H), 7.31 (d, *J* = 2.5 Hz, 1H), 7.28 (m, 4H), 7.25 (s, 1H), 2.64 (s, 3H), 2.62 (s, 6H), 2.08 (s, 3H), 1.94 (s, 6H), 1.94 (s, 6H), 1.84 (s, 6H), 1.72 (s, 3H), 1.48 (s, 9H), 1.26 (s, 9H), -0.13 (s, 3H), -2.42 (s, 2H)(Figure S23).

5-[4-(5-Methoxycarbonyl-2,7-di-*tert*-butyl-9,9-dimethylxanthenyl)]-10,15,20-tripentafluorophenylporphyrin, H_2(PFHPX-COOMe).

^{1}H NMR (400 MHz, Chloroform-*d*) δ 9.02 – 8.96 (d, 2H), 8.92 (s, 4H), 8.84 – 8.78 (m, 2H), 7.96 (d, *J* = 2.4 Hz, 1H), 7.94 (d, *J* = 2.4 Hz, 1H), 7.66 (d, *J* = 2.4 Hz, 1H), 7.33 (d, *J* = 2.4 Hz, 1H), 1.96 (s, 6H), 1.53 (s, 9H), 1.26 (s, 9H), -0.02 (s, 3H), -2.75 (s, 2H) (Figure S26).

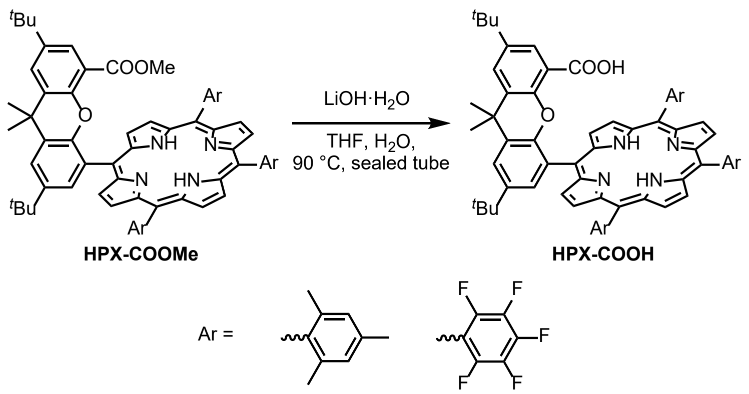

Scheme S2. General Procedure for Hydrolysis of Hangman Porphyrin Ester

To a pressure tube equipped with magnetic stirrer were added **HPX-COOMe** (0.1 mmol), LiOH·H_2O (10 mmol), THF (2 mL) and H_2O (10 mL). Then the tube was sealed and heated to 90 °C for about 24 hours. Then the reaction was monitored by TLC. Once the reaction ended then the solution was diluted with 100 mL H_2O, acidized to pH = 4 with 0.5 M HCl. The acidified solution was extracted with 50 mL DCM three times and the combined organic phase was washed with 50 mL brine once. Next the organic phase was dried over Na_2SO_4 and the solvent was removed under vacuum. The residue was purified by flash column chromatography (silica, DCM and petroleum ether as eluent) to afford purple solid.

5-[4-(5-Hydroxycarbonyl-2,7-di-*tert*-butyl-9,9-dimethylxanthenyl)]-10,15,20-trimesitylporphyrin, H_2(MesHPX-COOH)

^{1}H NMR (400 MHz, Chloroform-*d*) δ 8.67 (s, 2H), 8.65 – 8.56 (m, 6H), 8.02 (d, *J* = 2.4 Hz, 1H), 7.90 (d, *J* = 2.4 Hz, 1H), 7.71 (d, *J* = 2.5 Hz, 1H), 7.67 (d, *J* = 2.5 Hz, 1H), 7.30 (s, 1H), 7.26 – 7.24 (m, 6H), 2.63 (s, 3H), 2.60 (s, 6H), 1.98 (s, 3H), 1.95 (s, 6H), 1.90 (s, 6H), 1.86 (s, 3H), 1.83 (s, 6H), 1.52 (s, 9H), 1.25 (s, 9H), -2.46 (s, 2H) (yield: 90 %, Figure S24).

5-[4-(5-Hydroxycarbonyl-2,7-di-*tert*-butyl-9,9-dimethylxanthenyl)]-10,15,20-tripentafluorophenylporphyrin, H_2(PFHPX-COOH)

^{1}H NMR (400 MHz, Chloroform-*d*) δ 8.99 – 8.83 (m, 6H), 8.79 (d, *J* = 4.8 Hz, 2H), 8.08 (d, *J* = 2.3 Hz, 1H), 7.97 (d, *J* = 2.3 Hz, 1H), 7.71 (d, *J* = 2.5 Hz, 1H), 7.59 (d, *J* = 2.4 Hz, 1H), 1.96 (s, 6H), 1.55 (s, 9H), 1.23 (s, 9H), -2.81 (s, 2H) (yield: 88 %, Figure S27).

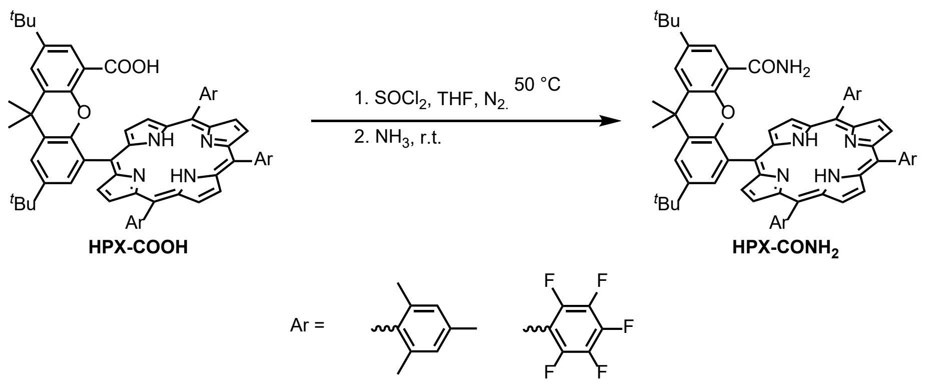

Scheme S3. General Procedure for the Amination of Hangman Porphyrin Acid

HPX-COOH (0.2 mmol) was dissolved in anhydrous THF (10 mL) under N_2 atmosphere at 50 °C. Then $SOCl_2$ (2 mmol) was added dropwise to the solution and the mixture was heated for 0.5 hour. The mixture was cooled to room temperature and was treated with 0.4 M NH_3-dioxane solution (10 mL). The resulting mixture was stirred at room temperature for 2 h. Then the solvent was removed under vacuum and the residue was purified by flash column chromatography (silica, DCM and petroleum ether as eluent) to afford purple solid.

5-[4-(5-Aminocarbonyl-2,7-di-*tert*-butyl-9,9-dimethylxanthenyl)]-10,15,20-trimesitylporphyrin, H_2(MesHPX-$CONH_2$)

^{1}H NMR (400 MHz, Chloroform-*d*) δ 8.69 (d, *J* = 4.7 Hz, 2H), 8.61 (dd, *J* = 7.1, 4.9 Hz, 6H), 8.05 (d, *J* = 2.3 Hz, 1H), 7.84 (d, *J* = 2.4 Hz, 1H), 7.53 (d, *J* = 5.1 Hz, 2H), 7.20 (d, *J* = 1.4 Hz, 6H), 2.58 (s, 3H), 2.55 (s, 6H), 1.98 (s, 3H), 1.87 (s, 6H), 1.80 (s, 15H), 1.50 (s, 9H), 1.16 (s, 9H), -2.59 (s, 2H) (yield: 78 %, Figure S25).

5-[4-(5-Aminocarbonyl-2,7-di-*tert*-butyl-9,9-dimethylxanthenyl)]-10,15,20-tripentafluorophenylporphyrin, H_2(PFHPX-$CONH_2$)

^{1}H NMR (400 MHz, Chloroform-*d*) δ 8.99 (d, *J* = 4.8 Hz, 2H), 8.95 (s, 4H), 8.84 (d, *J* = 5.0 Hz, 2H), 8.15 (d, *J* = 2.3 Hz, 1H), 7.98 (d, *J* = 2.3 Hz, 1H), 7.66 – 7.49 (m, 2H), 1.96 (s, 6H), 1.59 (s, 9H), 1.23 (s, 9H), -2.81 (s, 2H) (yield: 73 %, Figure S28).

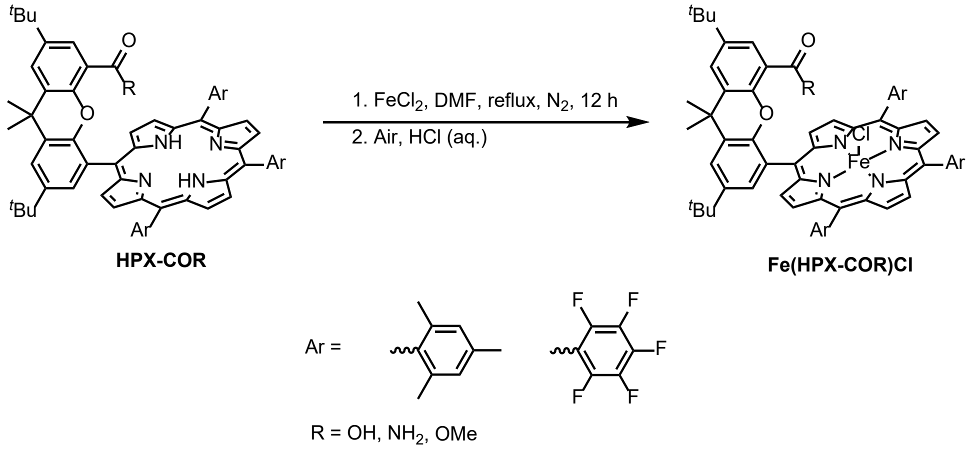

Scheme S4. General Procedure for the Iron Metalation of Hangman Porphyrin with $FeCl_2$

In an Argon-filled glovebox, a three-neck flask was added with free chlorin (0.2 mmol), anhydrous $FeCl_2$ (2 mmol) and DMF (20 mL). The mixture was refluxed under argon atmosphere for 12 hours. Then the solvent was removed under vacuum. The residue was dissolved in DCM, washed with 0.5 M HCl twice and brine once, purified by flash column chromatography (silica, DCM and MeOH as eluent) to afford brown solid.

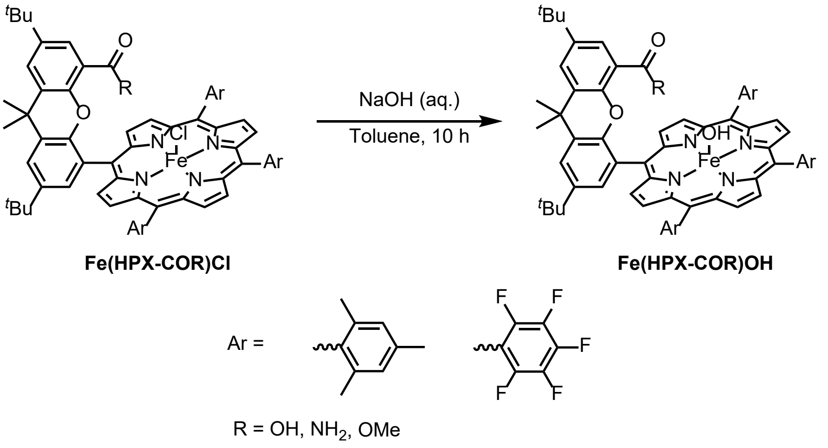

Scheme S5. General Procedure for Ligand Exchange of the Iron Porphyrin

A solution of Iron porphyrin (0.2 mmol) in toluene (5 mL) was treated with 1 M NaOH aqueous solution (10 mL) and stirred overnight. Then the aqueous phase was separated, and the organic phase was dried over Na_2SO_4. The solution was dried under vacuum to afford brown solid. Then the residue was recrystallized in DCM/*n*-hexane and dried under reduced pressure.

[Fe(MesHPX-COOMe)OH]

ESI-MS for [M-OH$^-$]$^+$: calcd for $C_{72}H_{72}FeN_4O_3$, 1096.5, found: 1096.5.

(yield: 90 %, Figure S29).

[Fe(MesHPX-COOH)OH]

ESI-MS for [M-OH$^-$]$^+$: calcd for $C_{71}H_{70}FeN_4O_3$: 1082.5. found: 1082.5.

(yield: 92 %, Figure S30).

[Fe(MesHPX-CONH$_2$)OH]

ESI-MS for [M-OH$^-$]$^+$: calcd for $C_{71}H_{71}FeN_5O_2$: 1081.5. found: 1081.5.

(yield: 85 %, Figure S31).

[Fe(PFHPX-COOMe)OH]

ESI-MS for [M-OH$^-$]$^+$: calcd for $C_{63}H_{39}F_{15}FeN_4O_3$: 1240.2. found: 1040.2.

(yield: 75 %, Figure S32).

[Fe(PFHPX-COOH)OH]

ESI-MS for [M-OH$^-$]$^+$: calcd for $C_{62}H_{37}F_{15}FeN_4O_3$: 1226.2. found: 1226.2.

(yield: 79 %, Figure S33).

[Fe(PFHPX-CONH$_2$)OH]

ESI-MS for [M-OH$^-$]$^+$: calcd for $C_{62}H_{38}F_{15}FeN_5O_2$: 1225.2. found: 1225.2.

(yield: 78 %, Figure S34).

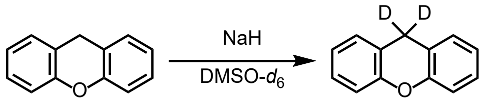

Scheme S6. General Procedure for Xanthene-d_2

Xanthene (2.7 mmol) was reacted with NaH (8.1 mmol) in DMSO-d_6 (3.0 mL) under an inert atmosphere. The deep red solution was stirred at room temperature for 8 h and then quenched with D_2O (5.0 mL). The crude product was filtered and washed with copious amounts of D_2O. 1H NMR confirmed >99% deuteration.

Xanthene-d_2

^{1}H NMR (400 MHz, Dimethyl Sulfoxide-d_6) δ 6.96 – 6.87 (m, 4H), 6.77 – 6.69 (m, 4H) (Figure S35).

Table S1. Products with Yields Obtained in the Stoichiometric Oxidation of 4-Methoxystyrene by Iron(IV)-Oxo Species[a].

entry	iron(IV)-oxo species	substrate	product and yield[b] (%)
1	**[Fe^{IV}(O)(TMP)]**		2-(4-methoxyphenyl)oxirane (65)
2	**2-COOH**		2-(4-methoxyphenyl)oxirane (99)
3	**2-COOMe**	4-methoxystyrene	2-(4-methoxyphenyl)oxirane (52)
4	**2-CONH$_2$**		2-(4-methoxyphenyl)oxirane (67)
5	**1-COOH**		2-(4-methoxyphenyl)oxirane (99)

[a]Reactions with iron(IV)-oxo species (1.0×10^{-1} mM) were performed in 2.5 mL of CH_3CN/CH_3OH (v/v 100:1) at 258 K. [b]Yields (shown in parentheses) were determined based on [iron(IV)-oxo species].

Table S2. Products with Yields Obtained in the Stoichiometric C-H Activation Reactions by Iron(IV)-Oxo Species[a].

entry	iron(IV)-oxo species	substrate	product and yield[b] (%)
1	**[Fe^{IV}(O)(TMP)]**	9, 10-dihydroanthracene	anthracene (69)
2	**2-COOH**		anthracene (68)
3	**2-COOMe**		anthracene (65)
4	**2-$CONH_2$**		anthracene (66)
5	**1-COOH**		anthracene (99)
6	**1-COOMe**		anthracene (54)

[a]Reactions with iron(IV)-oxo species (1.0×10^{-1} mM) were performed in 2.5 mL of CH_3CN/CH_3OH (v/v 100:1) at 258 K. [b]Yields (shown in parentheses) were determined based on [iron(IV)-oxo species].

Table S3. The half-lives of all Iron(IV)-Oxo Species[a].

entry	iron(IV)-oxo species	$t_{1/2}$
1	**[Fe^{IV}(O)(TMP)]**	30 min
2	**2-COOH**	25 min
3	**2-COOMe**	30 min
4	**2-$CONH_2$**	25 min
5	**1-COOH**	30 min
6	**1-COOMe**	35 min
7	**1-$CONH_2$**	30 min

[a]Iron(IV)-oxo species (1.0×10^{-1} mM) were formed in 2.5 mL of CH_3CN/CH_3OH (v/v 100:1) at 258 K.

Table S4. The bond dissociation free energy (BDFE) of O-H bonds in intermediates **8**, **9**, and **10** was investigated through theoretical calculations.

Cpd II	2-COOH	2-CONH$_2$	2-COOMe
O-H bond BDFE of corresponding Fe-OH intermediates in kacl/mol	85.5	83.8	81.9

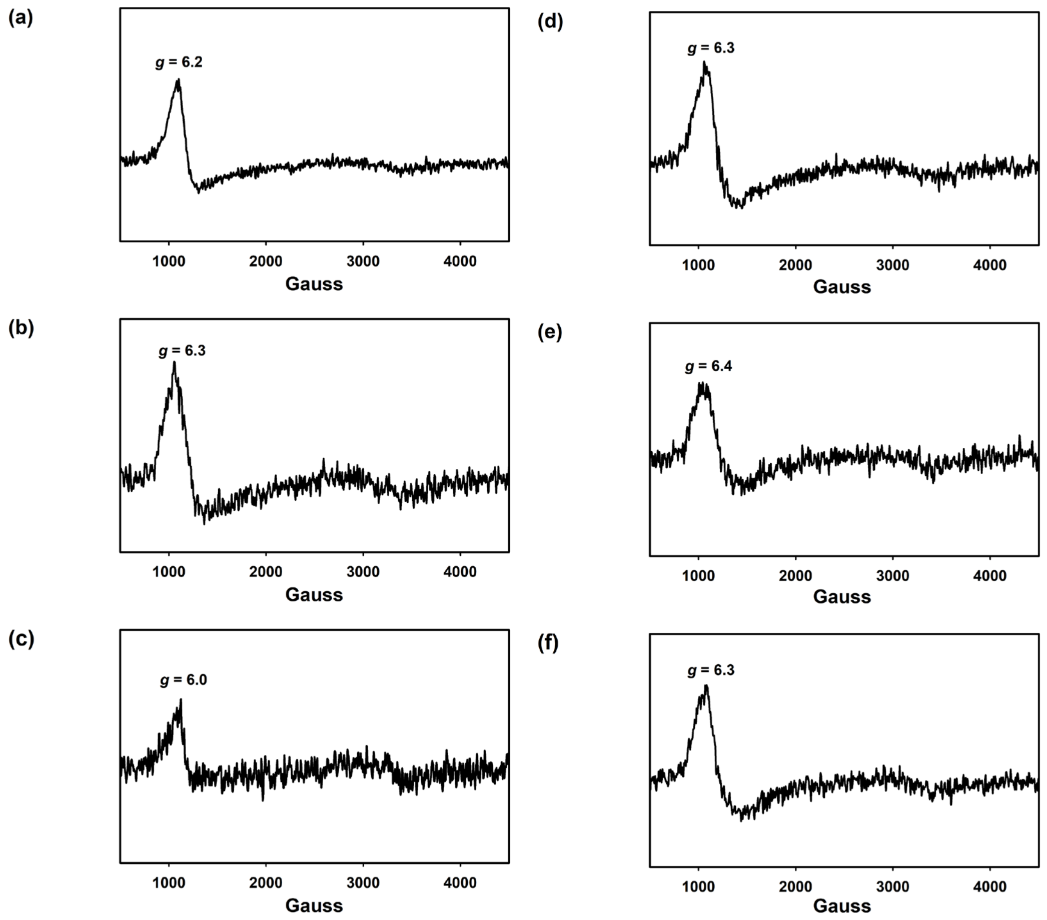

Figure S1. EPR spectrum of (a) [**Fe(PFHPX-COOH)OH**], (b) [**Fe(PFHPX-CONH$_2$)OH**], (c) [**Fe(PFHPX-COOMe)OH**], (d) [**Fe(MesHPX-COOH)OH**] and (e) [**Fe(MesHPX-CONH$_2$)OH**] (f) [**Fe(MesHPX-COOMe)OH**], in acetone at 90 K.

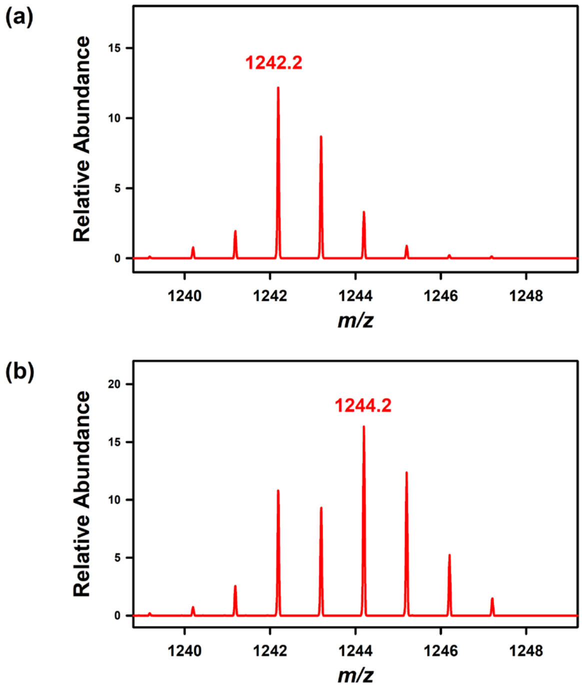

Figure S2. ESI-MS spectra of (a) **1-COOH**-^{16}O and (b) **1-COOH**-^{18}O in CH_3CN/CH_3OH (v/v 100:1) in positive mode. The peaks at *m/z*= 1242.2 and 1244.2 correspond to [Fe^{IV}(^{16}O)(PFHPX-COOH)] (calcd *m/z*= 1242.2) and [Fe^{IV}(^{18}O)(PFHPX-COOH)] (calcd *m/z*= 1244.2), respectively.

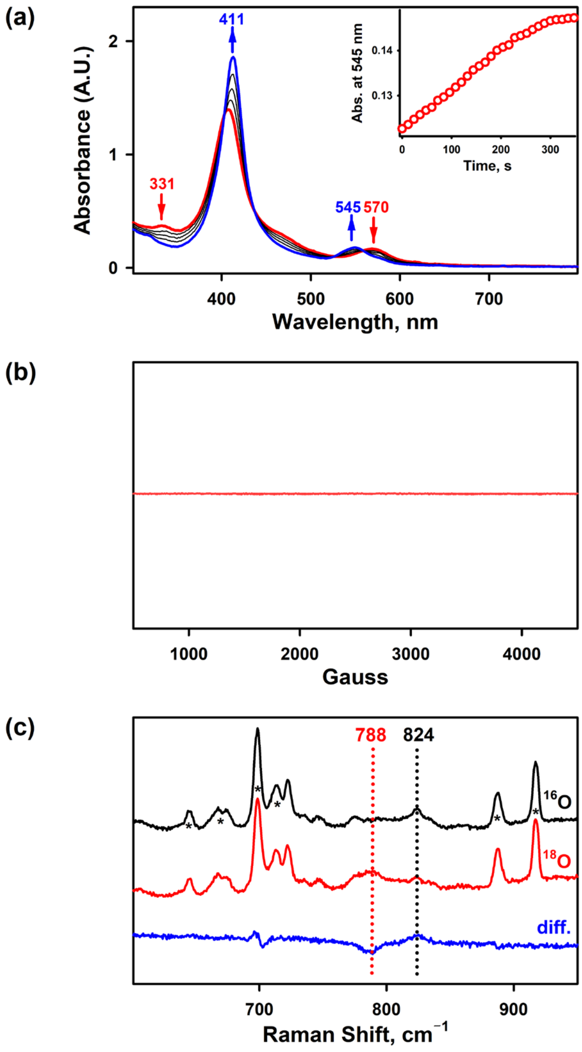

Figure S3. (a) UV–vis spectral changes showing the formation of **1-CONH₂** (blue line) in the reaction of [Fe^{III}(PFHPX-$CONH_2$)(OH)] (1.0×10^{-2} mM, red line) and PhIO (2.5×10^{-2} mM) in CH_3CN/CH_3OH (v/v 100:1) at 258 K. (b) X-band EPR spectrum of **1-CONH₂**. Spectrum was recorded in acetone at 90 K. (c) rRaman spectra of **1-CONH₂-^{16}O** (1.0 mM, black line) and **1-CONH₂-^{18}O** (1.0 mM, red line). The blue line shows the difference spectrum of **1-CONH₂-^{16}O** and **1-CONH₂-^{18}O.** The peaks marked with an asterisk (*) are from the solvent. The rRaman spectra were recorded with 442 nm excitation in acetone-d_6: CH_3CN=1:1 at 77 K.

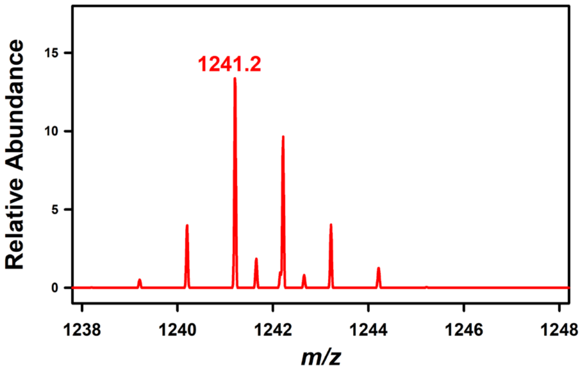

Figure S4. ESI-MS spectra of **1-CONH$_2$**-^{16}O in CH_3CN/CH_3OH (v/v 100:1) in positive mode. The peaks at *m/z*= 1241.2 correspond to [Fe^{IV}(O)(PFHPX-CONH$_2$)] (calcd *m/z*= 1241.2).

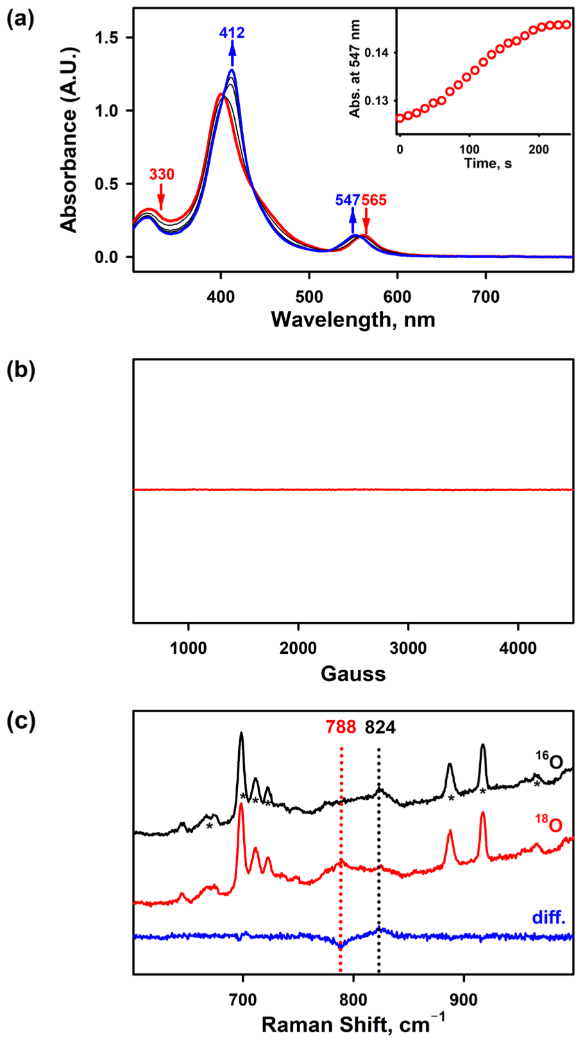

Figure S5. (a) UV–vis spectral changes showing the formation of **1-COOMe** (blue line) in the reaction of [Fe^{III}(PFHPX-COOMe)(OH)] (1.0×10^{-2} mM, red line) and PhIO (2.5×10^{-2} mM) in CH_3CN/CH_3OH (v/v 100:1) at 258 K. (b) X-band EPR spectrum of **1-COOMe**. Spectrum was recorded in acetone at 90 K. (c) rRaman spectra of **1-COOMe-^{16}O** (1.0 mM, black line) and **1-COOMe-^{18}O** (1.0 mM, red line). The blue line shows the difference spectrum of **1-COOMe-^{16}O** and **1-COOMe-^{18}O.** The peaks marked with an asterisk (*) are from the solvent. The rRaman spectra were recorded with 442 nm excitation in acetone-d_6: CH_3CN=1:1 at 77 K.

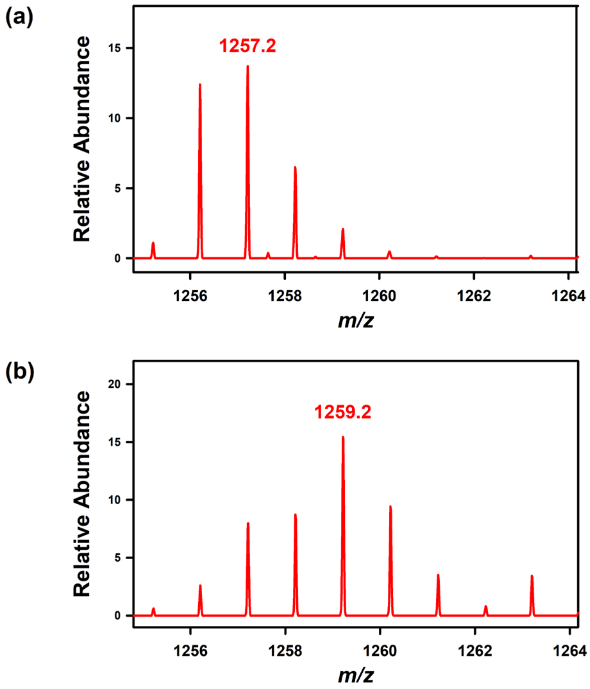

Figure S6. ESI-MS spectra of (a) **1-COOMe**-^{16}O and (b) **1-COOMe**-^{18}O in CH_3CN/CH_3OH (v/v 100:1) in positive mode. The peaks at *m*/*z*= 1257.2 and 1259.2 correspond to [Fe^{IV}(^{16}OH)(PFHPX-COOMe)]$^+$ (calcd *m*/*z*= 1257.2) and [Fe^{IV}(^{18}OH)(PFHPX-COOMe)]$^+$ (calcd *m*/*z*= 1259.2), respectively.

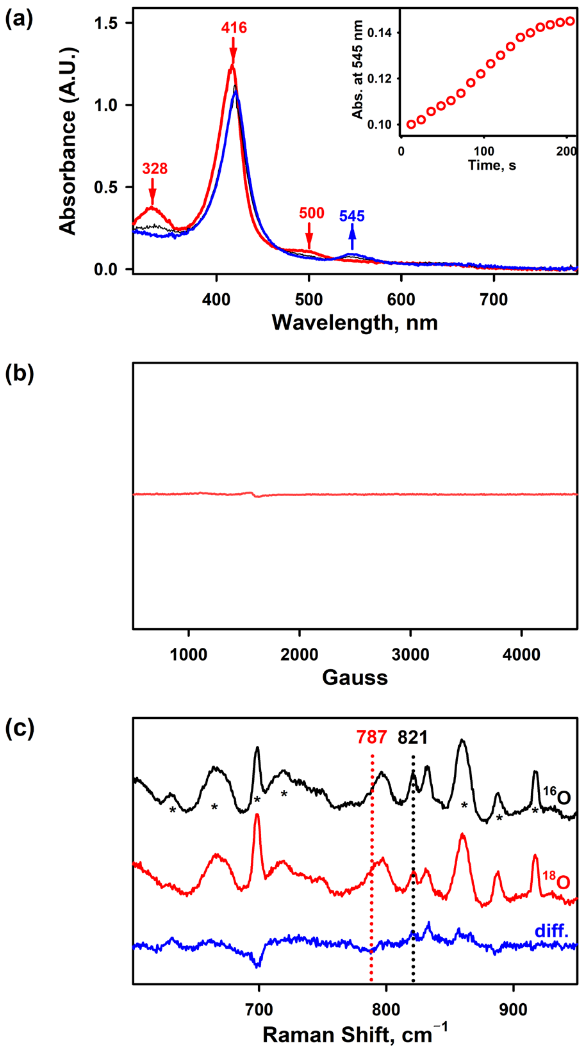

Figure S7. (a) UV–vis spectral changes showing the formation of **2-COOH** (blue line) in the reaction of [Fe^{III}(MesHPX-COOH)(OH)] (1.0×10^{-2} mM, red line) and PhIO (2.5×10^{-2} mM) in CH_3CN/CH_3OH (v/v 100:1) at 258 K. (b) X-band EPR spectrum of **2-COOH**. Spectrum was recorded in acetone at 90 K. (c) rRaman spectra of **2-COOH-^{16}O** (1.0 mM, black line) and **2-COOH-^{18}O** (1.0 mM, red line). The blue line shows the difference spectrum of **2-COOH-^{16}O** and **2-COOH-^{18}O.** The peaks marked with an asterisk (*) are from the solvent. The rRaman spectra were recorded with 442 nm excitation in acetone-*d*$_6$: CH_3CN=1:1 at 77 K.

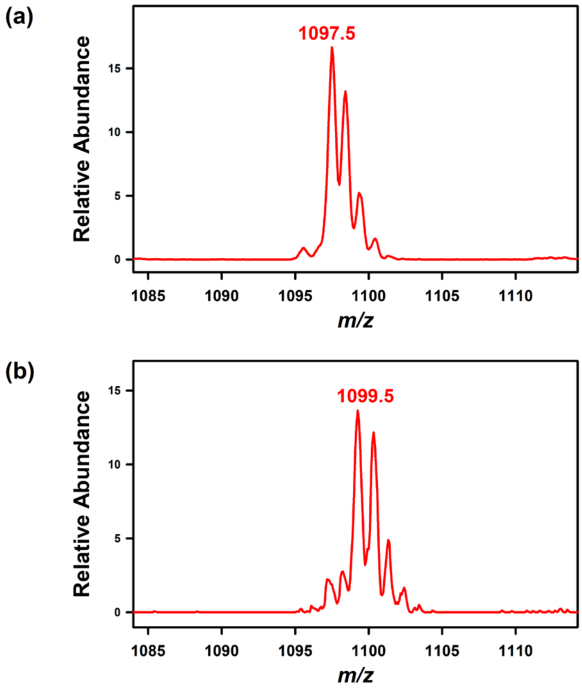

Figure S8. ESI-MS spectra of (a) **2-COOH**-^{16}O and (b) **2-COOH**-^{18}O in CH_3CN/CH_3OH (v/v 100:1) in negative mode. The peaks at *m/z*= 1097.5 and 1099.5 correspond to [Fe^{IV}(^{16}O)(MesHPX-COO^-)] (calcd *m/z*= 1097.5) and [Fe^{IV}(^{18}O)(MesHPX-COO^-)] (calcd *m/z*= 1099.5), respectively.

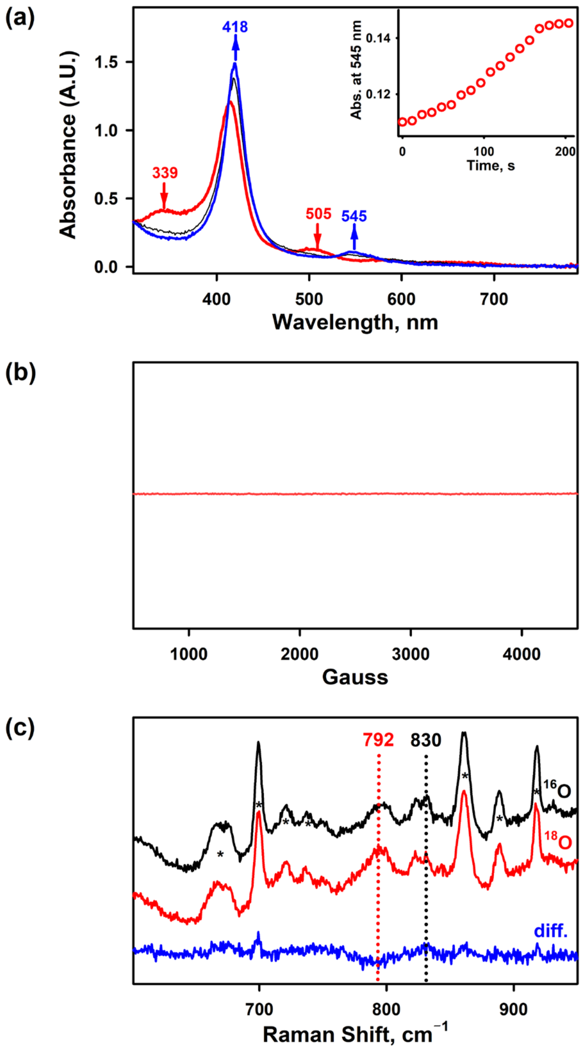

Figure S9. (a) UV–vis spectral changes showing the formation of **2-CONH$_2$** (blue line) in the reaction of [Fe^{III}(MesHPX-CO**NH$_2$**)(OH)] (1.0 × 10^{-2} mM, red line) and PhIO (2.5 × 10^{-2} mM) in CH_3CN/CH_3OH (v/v 100:1) at 258 K. (b) X-band EPR spectrum of **2-CONH$_2$**. Spectrum was recorded in acetone at 90 K. (c) rRaman spectra of **2-CONH$_2$-^{16}O** (1.0 mM, black line) and **2-CONH$_2$ -^{18}O** (1.0 mM, red line). The blue line shows the difference spectrum of **2-CONH$_2$-^{16}O** and **2-CONH$_2$-^{18}O.** The peaks marked with an asterisk (*) are from the solvent. The rRaman spectra were recorded with 442 nm excitation in acetone-d_6: CH_3CN=1:1 at 77 K.

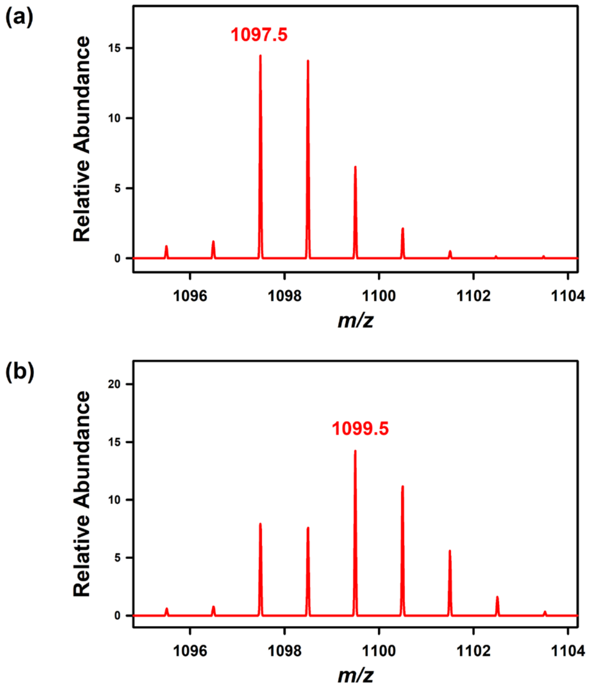

Figure S10. ESI-MS spectra of (a) **2-CONH₂**-^{16}O and (b) **2-CONH₂**-^{18}O in CH_3CN/CH_3OH (v/v 100:1) in positive mode. The peaks at *m/z*= 1097.5 and 1099.5 correspond to [Fe^{IV}(^{16}O)(MesHPX-$CONH_2$)] (calcd *m/z*= 1097.5) and [Fe^{IV}(^{18}O)(MesHPX-$CONH_2$)] (calcd *m/z*= 1099.5), respectively.

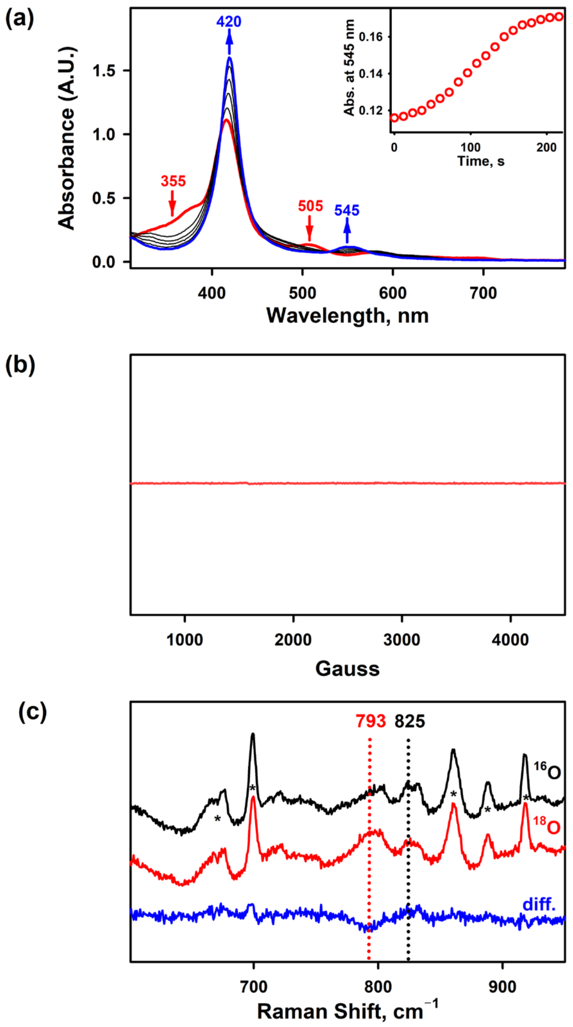

Figure S11. (a) UV–vis spectral changes showing the formation of **2-COOMe** (blue line) in the reaction of [Fe^{III}(MesHPX-COOMe)(OH)] (1.0×10^{-2} mM, red line) and PhIO (2.5×10^{-2} mM) in CH_3CN/CH_3OH (v/v 100:1) at 258 K. (b) X-band EPR spectrum of **2-COOMe**. Spectrum was recorded in acetone at 90 K. (c) rRaman spectra of **2-COOMe-^{16}O** (1.0 mM, black line) and **2-COOMe-^{18}O** (1.0 mM, red line). The blue line shows the difference spectrum of **2-COOMe-^{16}O** and **2-COOMe-^{18}O.** The peaks marked with an asterisk (*) are from the solvent. The rRaman spectra were recorded with 442 nm excitation in acetone-d_6: CH_3CN=1:1 at 77 K.

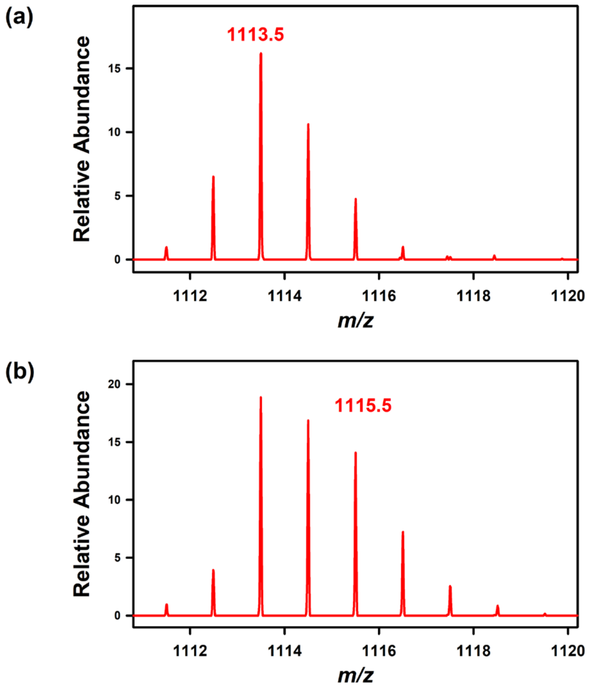

Figure S12. ESI-MS spectra of (a) **2-COOMe**-^{16}O and (b) **2-COOMe**-^{18}O in CH_3CN/CH_3OH (v/v 100:1) in positive mode. The peaks at *m/z*= 1113.5 and 1115.5 correspond to $[Fe^{IV}(^{16}OH)(MesHPX\text{-}COOMe)]^+$ (calcd *m/z*= 1113.5) and $[Fe^{IV}(^{18}OH)(MesHPX\text{-}COOMe)]^+$ (calcd *m/z*= 1115.5), respectively.

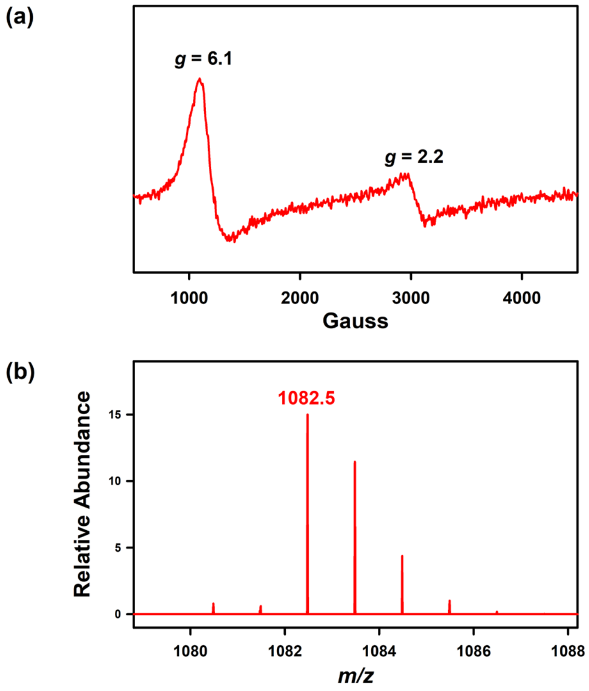

Figure S13. (a) X-band EPR spectrum of the reaction with **2-COOH** and 4-methoxystyrene. Spectrum was recorded at 90 K. (b) ESI-MS spectra of the reaction with **2-COOH** and 4-methoxystyrene. The peaks at *m*/*z*= 1082.5 correspond to $[Fe^{III}(MesHPX\text{-}COOH)]^{+}$.

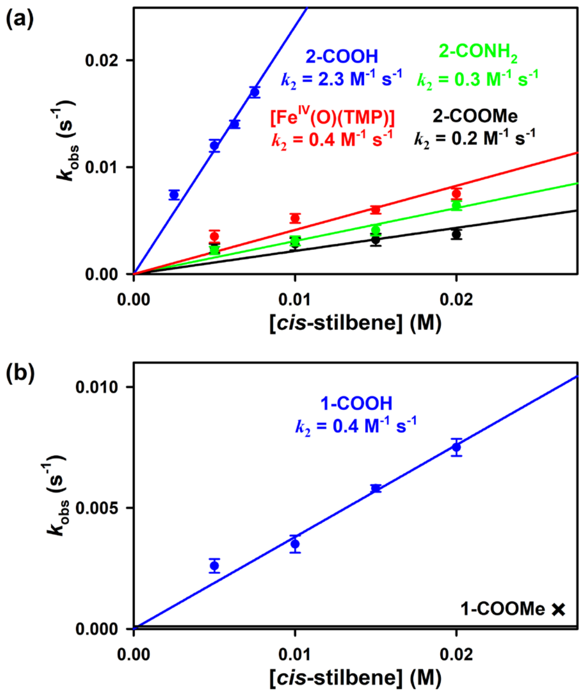

Figure S14. (a) Plots of k_{obs} against concentration of *cis*-stilbene to determine the second-order rate constants in epoxidation by **2-COOH** (5.0×10^{-2} mM, blue line), **2-CONH$_2$** (5.0×10^{-2} mM, green line), **2-COOMe** (5.0×10^{-2} mM, black line) and [Fe^{IV}(O)(TMP)] (5.0×10^{-2} mM, red line) at 258 K. (b) Plots of k_{obs} against concentration of *cis*-stilbene to determine the second-order rate constants in epoxidation by **1-COOH** (5.0×10^{-2} mM, blue line) and **1-COOMe** (5.0×10^{-2} mM, black line) at 258 K.

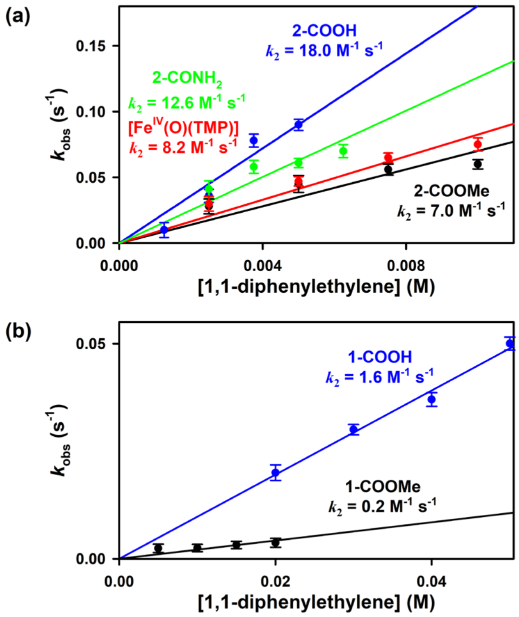

Figure S15. (a) Plots of k_{obs} against concentration of 1,1-diphenylethylene to determine the second-order rate constants in epoxidation by **2-COOH** (5.0×10^{-2} mM, blue line), **2-CONH₂** (5.0×10^{-2} mM, green line), **2-COOMe** (5.0×10^{-2} mM, black line) and [Fe^{IV}(O)(TMP)] (5.0×10^{-2} mM, red line) at 258 K. (b) Plots of k_{obs} against concentration of 1,1-diphenylethylene to determine the second-order rate constants in epoxidation by **1-COOH** (5.0×10^{-2} mM, blue line) and **1-COOMe** (5.0×10^{-2} mM, black line) at 258 K.

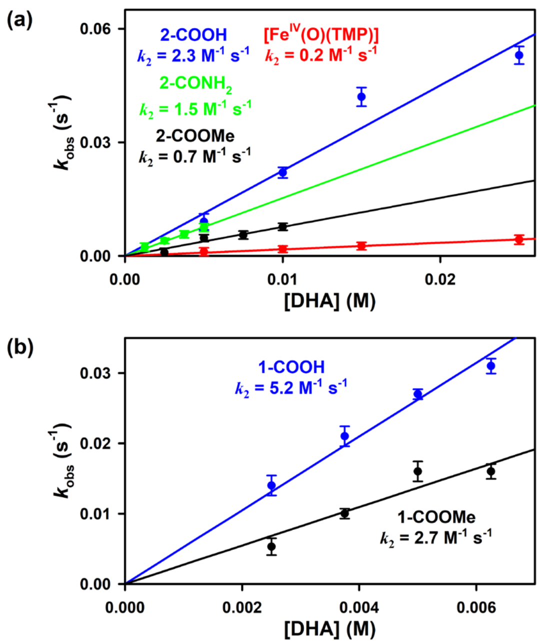

Figure S16. (a) Plots of k_{obs} against concentration of DHA to determine the second-order rate constants in C-H activation reaction by **2-COOH** (5.0×10^{-2} mM, blue line), **2-CONH₂** (5.0×10^{-2} mM, green line), **2-COOMe** (5.0×10^{-2} mM, black line) and [Fe^{IV}(O)(TMP)] (5.0×10^{-2} mM, red line) at 258 K. (b) Plots of k_{obs} against concentration of DHA to determine the second-order rate constants in C-H activation reaction by **1-COOH** (5.0×10^{-2} mM, blue line) and **1-COOMe** (5.0×10^{-2} mM, black line) at 258 K.

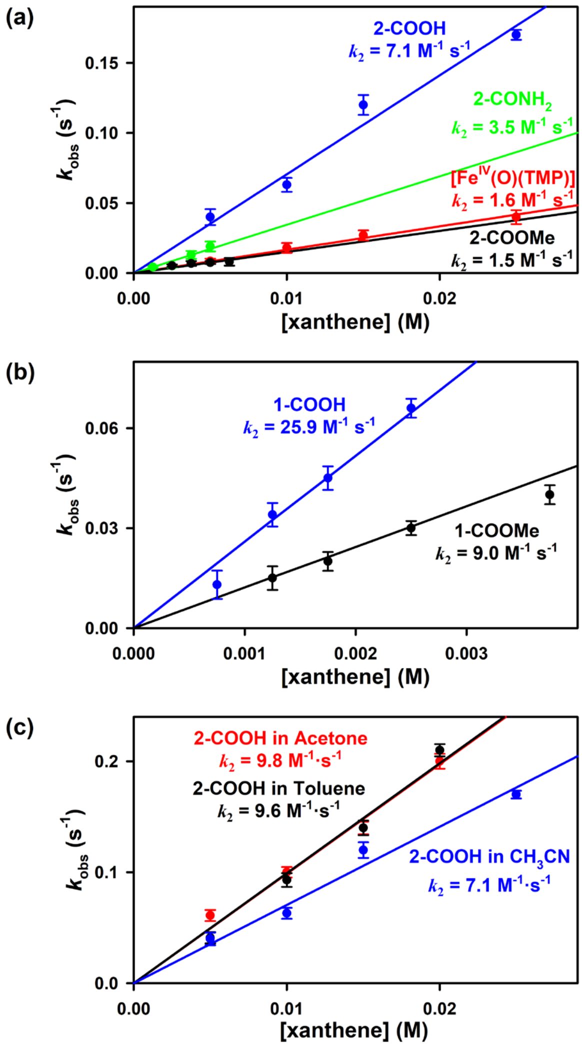

Figure S17. (a) Plots of k_{obs} against concentration of xanthene to determine the second-order rate constants in C-H activation reaction by **2-COOH** (5.0×10^{-2} mM, blue line), **2-CONH₂** (5.0×10^{-2} mM, green line), **2-COOMe** (5.0×10^{-2} mM, black line) and [Fe^{IV}(O)(TMP)] (5.0×10^{-2} mM, red line) at 258 K. (b) Plots of k_{obs} against concentration of xanthene to determine the second-order rate constants in C-H activation reaction by **1-COOH** (5.0×10^{-2} mM, blue line) and **1-COOMe** (5.0×10^{-2} mM, black line) at 258 K. (c) Plots of k_{obs} against concentration of xanthene to determine the second-order rate constants in C-H activation reaction by **2-COOH** in different solvent system such as in acetone, toluene and CH_3CN (trace amount of MeOH was used to dissolve PhIO) at 258 K.

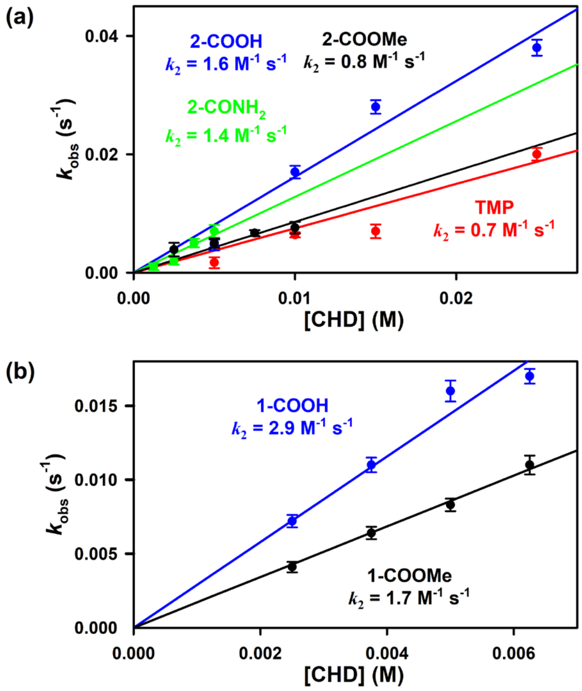

Figure S18. (a) Plots of k_{obs} against concentration of CHD to determine the second-order rate constants in C-H activation reaction by **2-COOH** (5.0×10^{-2} mM, blue line), **2-CONH$_2$** (5.0×10^{-2} mM, green line), **2-COOMe** (5.0×10^{-2} mM, black line) and [Fe^{IV}(O)(TMP)] (5.0×10^{-2} mM, red line) at 258 K. (b) Plots of k_{obs} against concentration of CHD to determine the second-order rate constants in C-H activation reaction by **1-COOH** (5.0×10^{-2} mM, blue line) and **1-COOMe** (5.0×10^{-2} mM, black line) at 258 K.

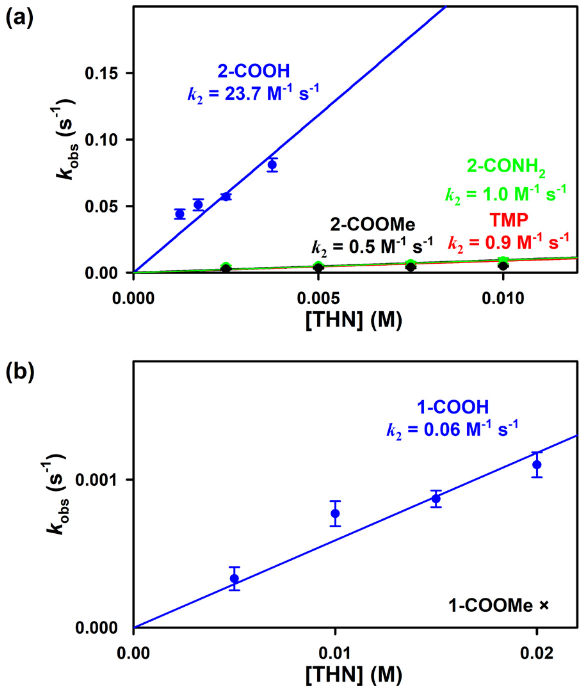

Figure S19. (a) Plots of k_{obs} against concentration of THN to determine the second-order rate constants in C-H activation reaction by **2-COOH** (5.0×10^{-2} mM, blue line), **2-CONH₂** (5.0×10^{-2} mM, green line), **2-COOMe** (5.0×10^{-2} mM, black line) at 258 K. (b) Plots of k_{obs} against concentration of THN to determine the second-order rate constants in C-H activation reaction by **1-COOH** (5.0×10^{-2} mM, blue line) and **1-COOMe** (5.0×10^{-2} mM, black line) at 258 K.

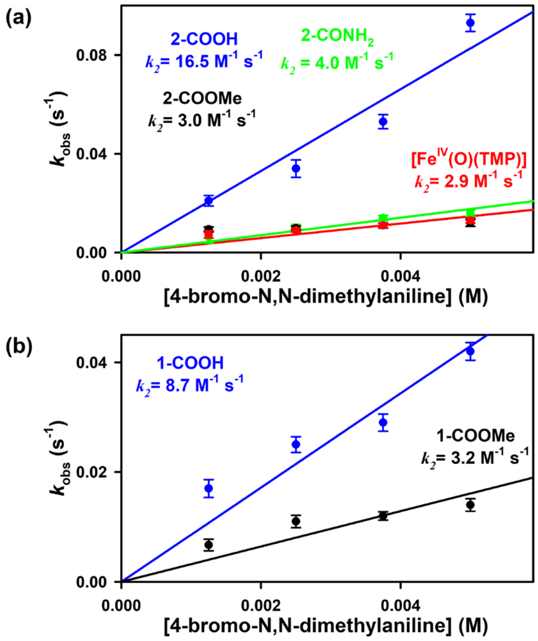

Figure S20. (a) Plots of k_{obs} against concentration of 4-bromo-*N, N*-dimethylaniline to determine the second-order rate constants in electron transfer reaction by **2-COOH** (5.0×10^{-2} mM, blue line), **2-CONH₂** (5.0×10^{-2} mM, green line), **2-COOMe** (5.0×10^{-2} mM, black line) and [Fe^{IV}(O)(TMP)] (5.0×10^{-2} mM, red line) at 258 K. (b) Plots of k_{obs} against concentration of 4-bromo-*N, N*-dimethylaniline to determine the second-order rate constants in electron transfer reaction by **1-COOH** (5.0×10^{-2} mM, blue line) and **1-COOMe** (5.0×10^{-2} mM, black line) at 258 K.

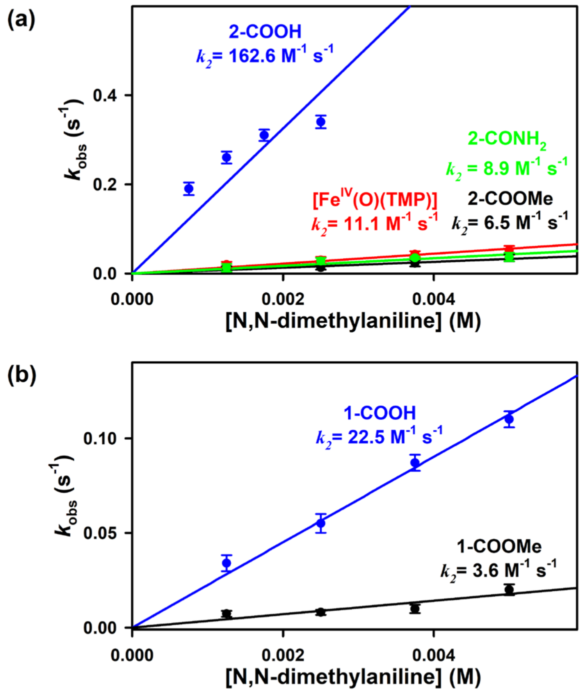

Figure S21. (a) Plots of k_{obs} against concentration of *N, N*-dimethylaniline to determine the second-order rate constants in electron transfer reaction by **2-COOH** (5.0×10^{-2} mM, blue line), **2-CONH₂** (5.0×10^{-2} mM, green line), **2-COOMe** (5.0×10^{-2} mM, black line) and [Fe^{IV}(O)(TMP)] (5.0×10^{-2} mM, red line) at 258 K. (b) Plots of k_{obs} against concentration of *N, N*-dimethylaniline to determine the second-order rate constants in electron transfer reaction by **1-COOH** (5.0×10^{-2} mM, blue line) and **1-COOMe** (5.0×10^{-2} mM, black line) at 258 K.

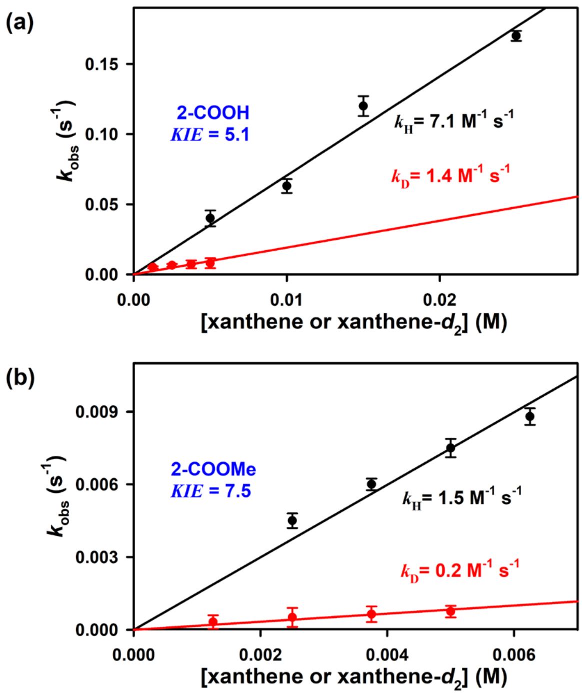

Figure S22. (a) Plots of pseudo-first-order rate constants vs. concentrations of xanthene (black circles) and xanthene-d_2 (red circles) obtained in the oxidation of xanthene by **2-COOH** in CH_3CN at 258 K. (b) Plots of pseudo-first-order rate constants vs. concentrations of xanthene (black circles) and xanthene-d_2 (red circles) obtained in the oxidation of xanthene by **2-COOMe** in CH_3CN at 258 K.

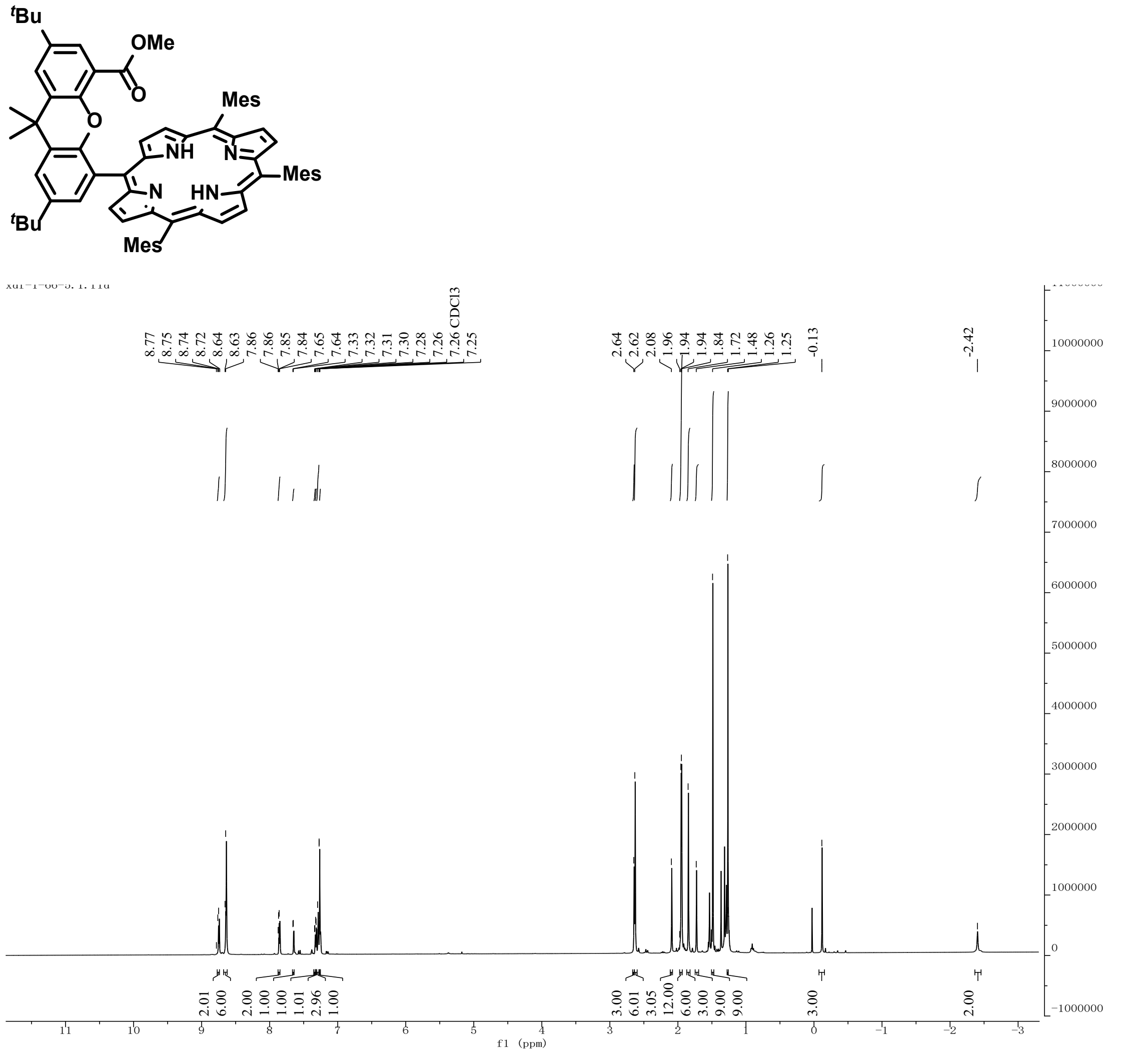

Figure S23. ^{1}H NMR spectrum of **H_2(MesHPX-COOMe)** in $CDCl_3$.

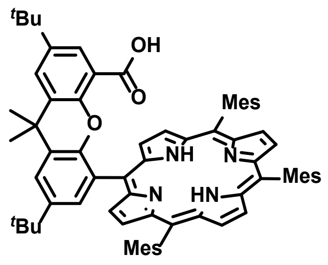

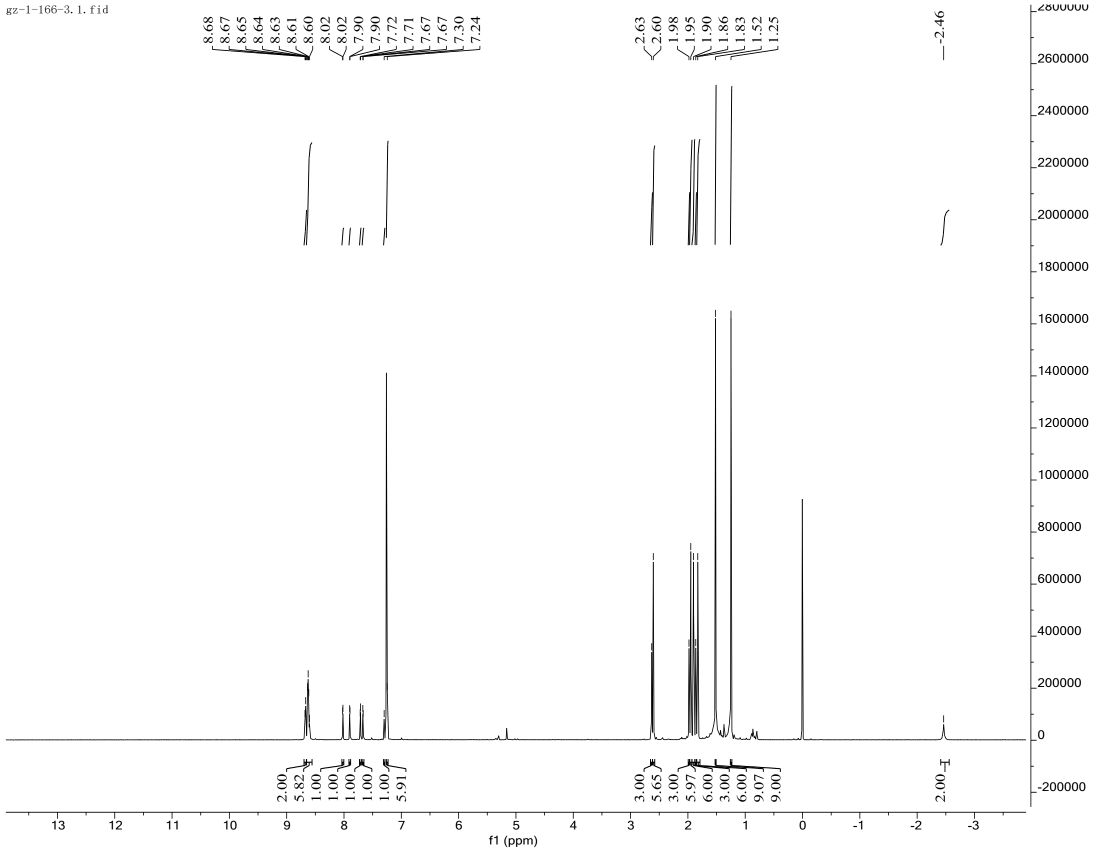

Figure S24. ^{1}H NMR spectrum of **H_2(MesHPX-COOH)** in $CDCl_3$.

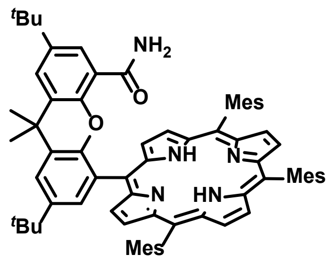

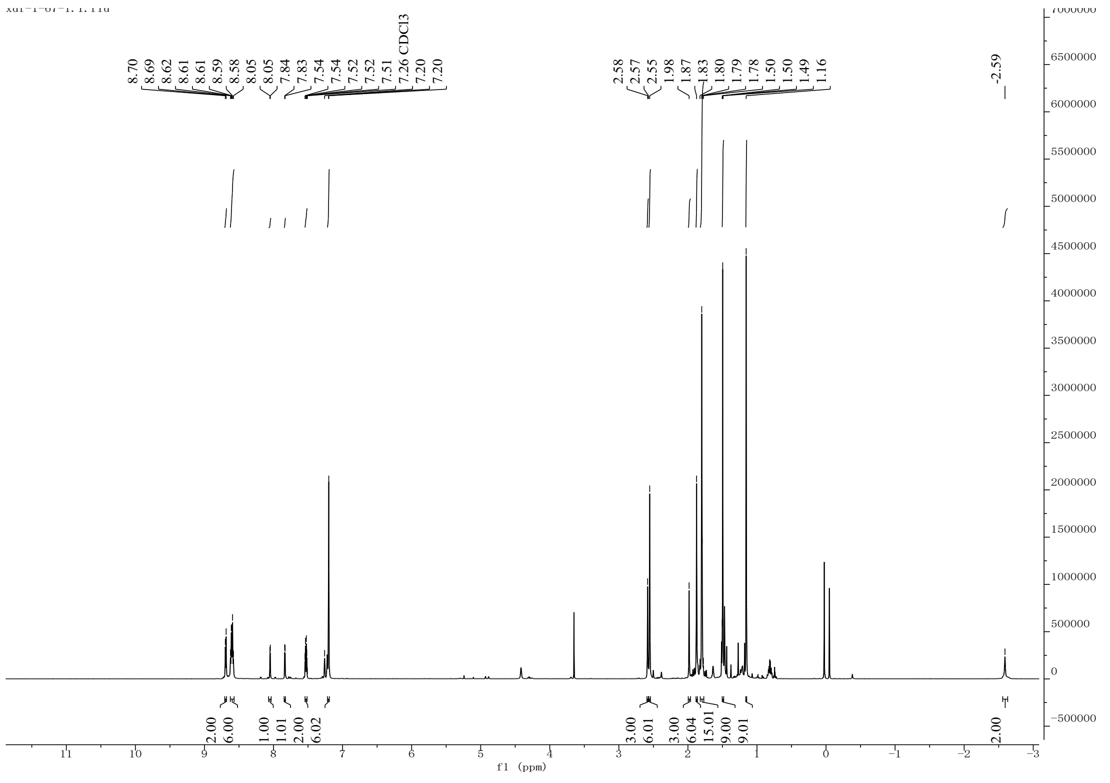

Figure S25. ^{1}H NMR spectrum of **H_2(MesHPX-CONH$_2$)** in $CDCl_3$.

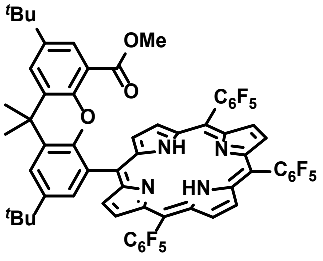

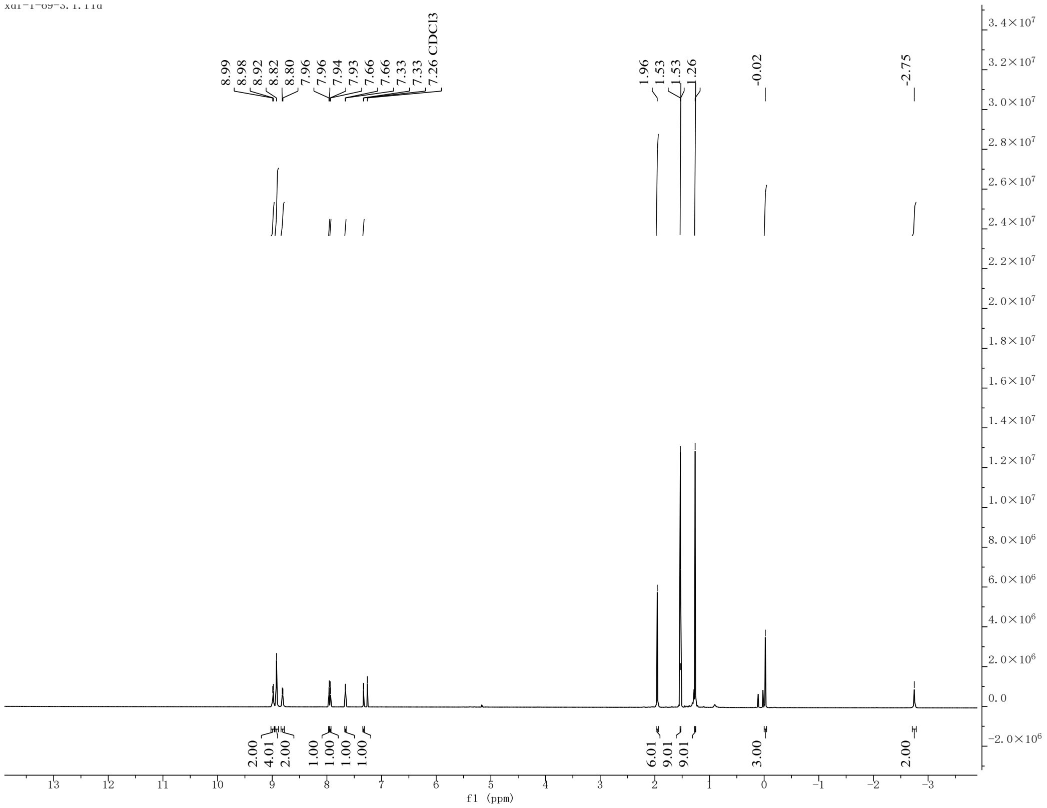

Figure S26. ^{1}H NMR spectrum of **H_2(PFHPX-COOMe)** in $CDCl_3$.

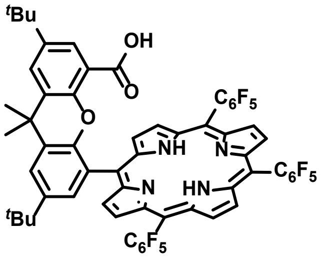

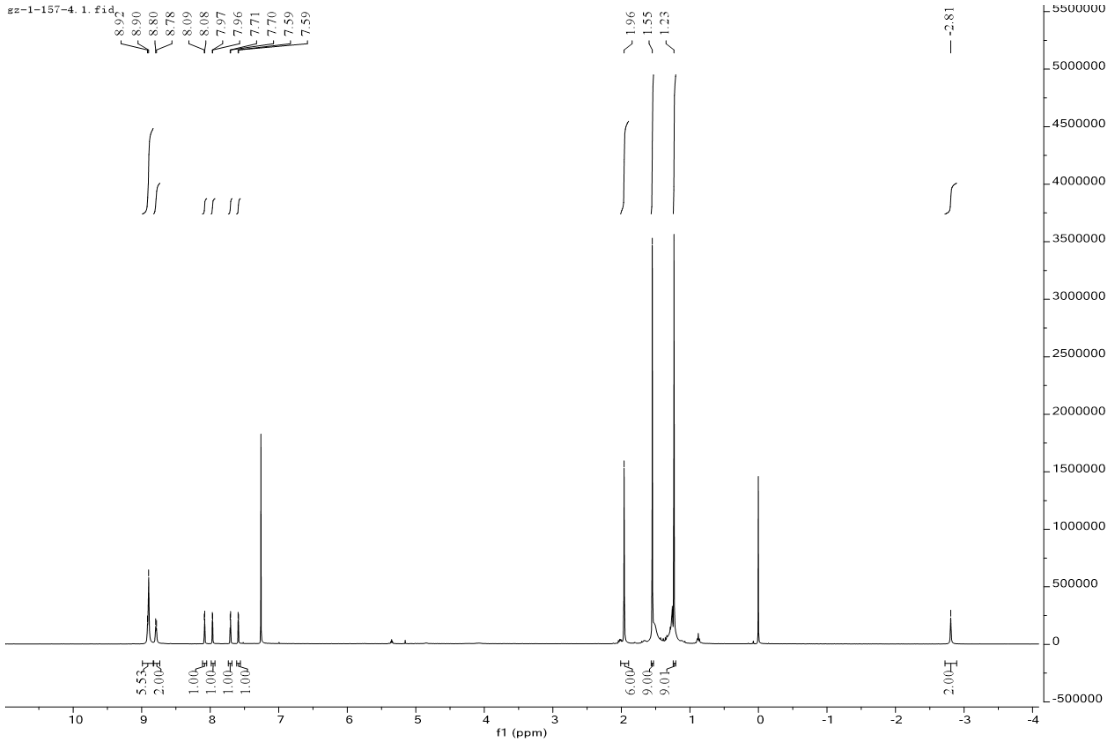

Figure S27. ^{1}H NMR spectrum of **H_2(PFHPX-COOH)** in $CDCl_3$.

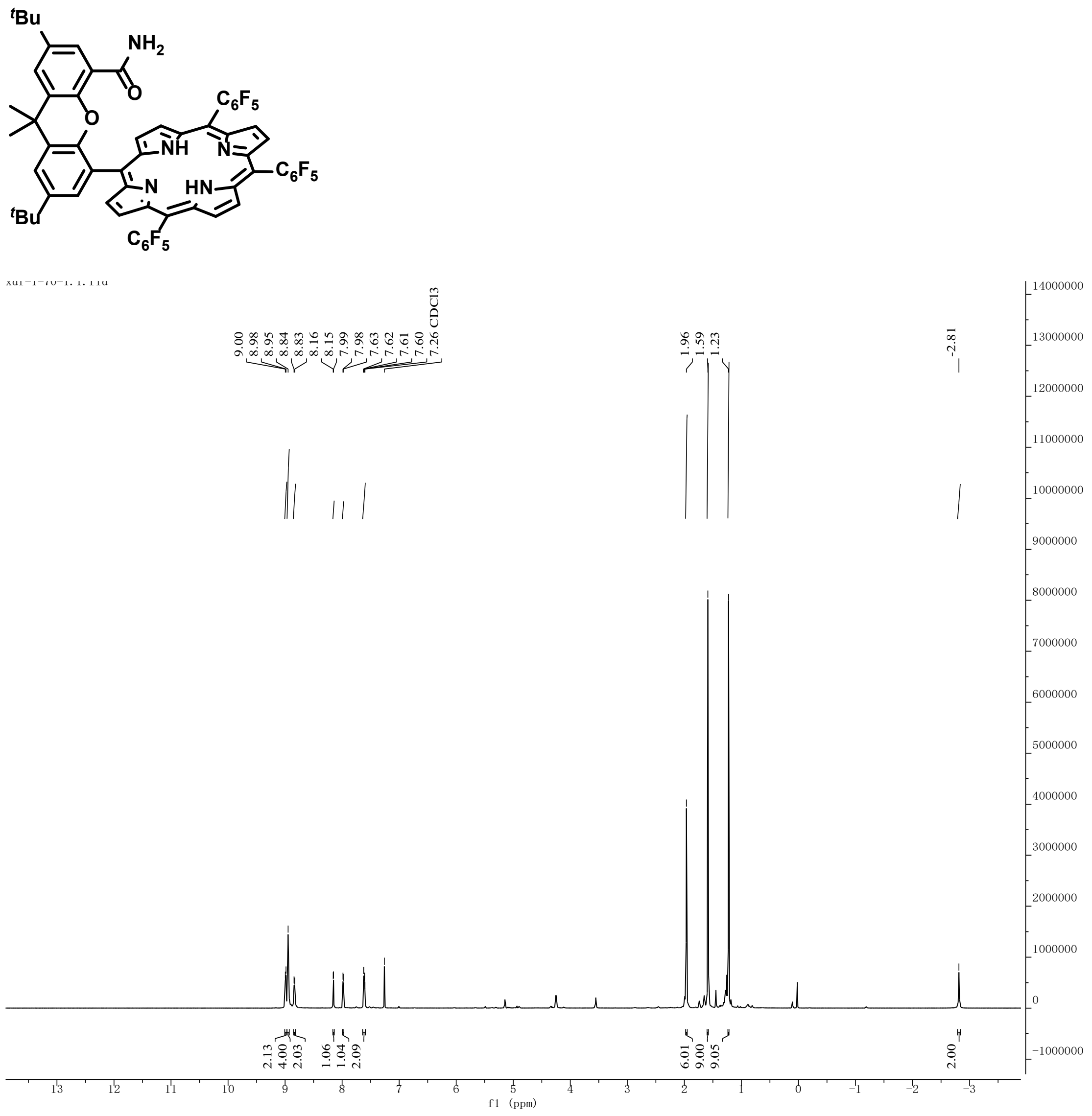

Figure S28. ¹H NMR spectrum of **H_2(PFHPX-CONH$_2$)** in $CDCl_3$.

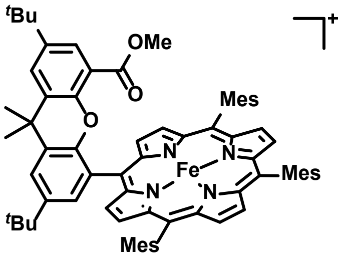

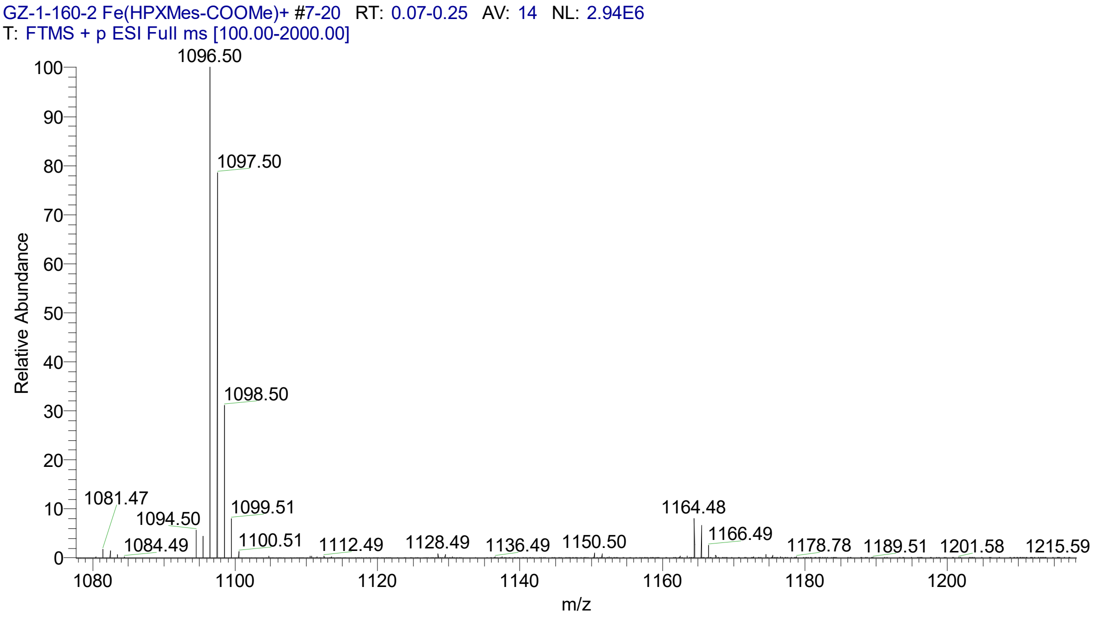

Figure S29. ESI-MS spectrum of [**Fe(MesHPX-COOMe)OH**] in CH_3CN.

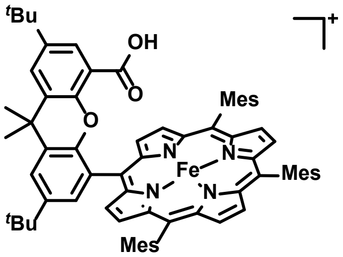

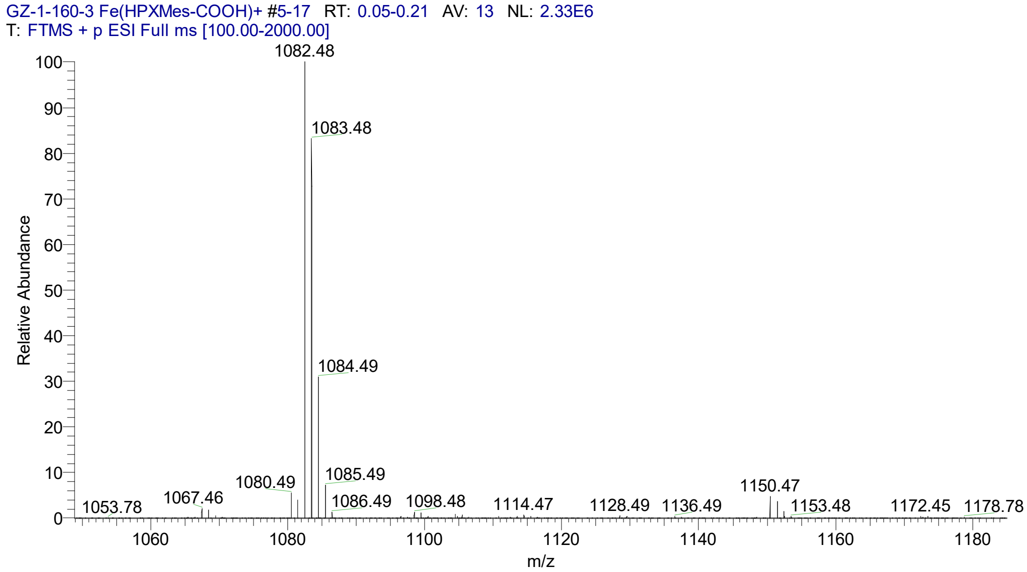

Figure S30. ESI-MS spectrum of [**Fe(MesHPX-COOH)OH**] in CH_3CN.

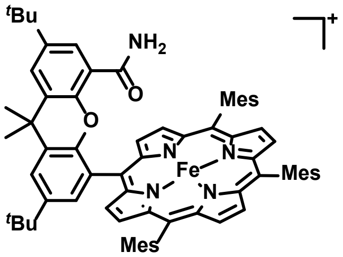

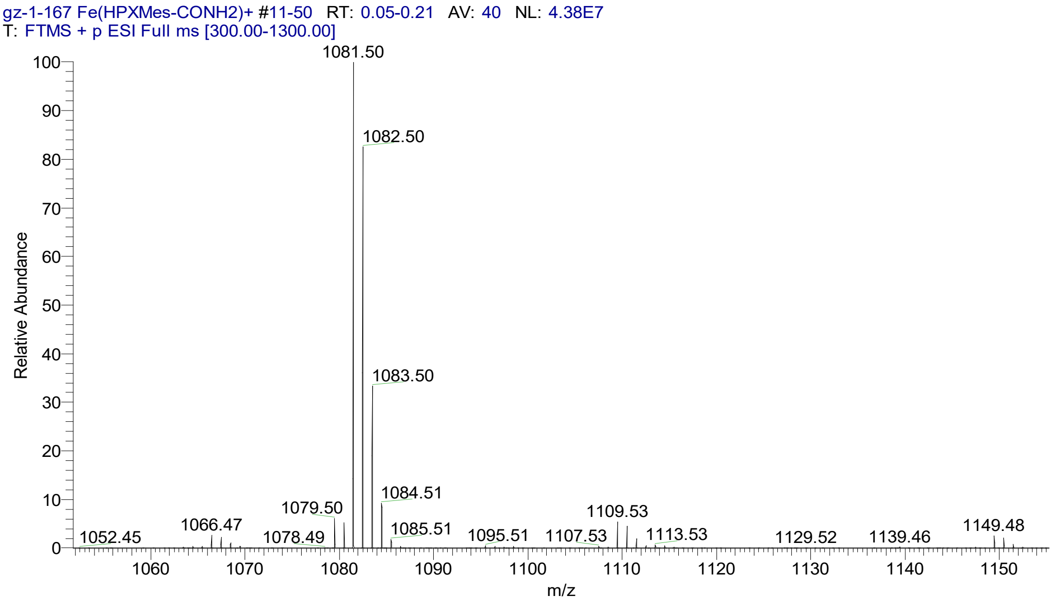

Figure S31. ESI-MS spectrum of [**Fe(MesHPX-CONH$_2$)OH**] in CH_3CN.

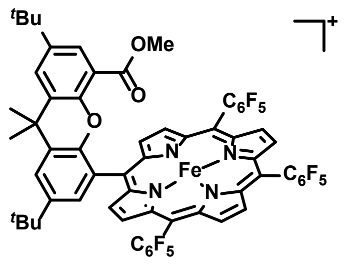

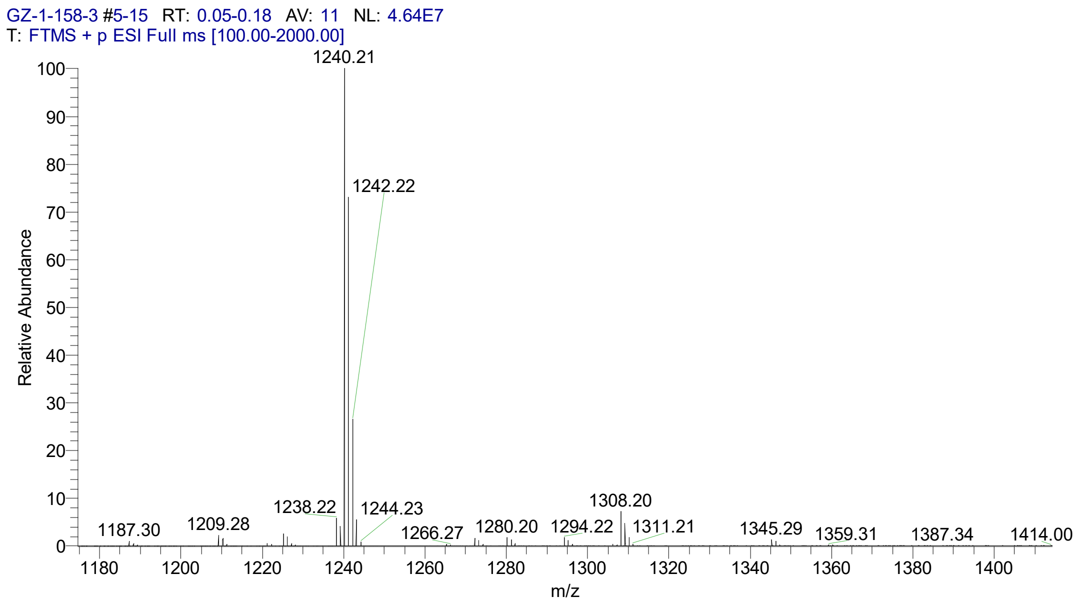

Figure S32. ESI-MS spectrum of [**Fe(PFHPX-COOMe)OH**] in CH_3CN.

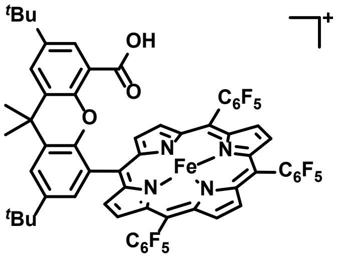

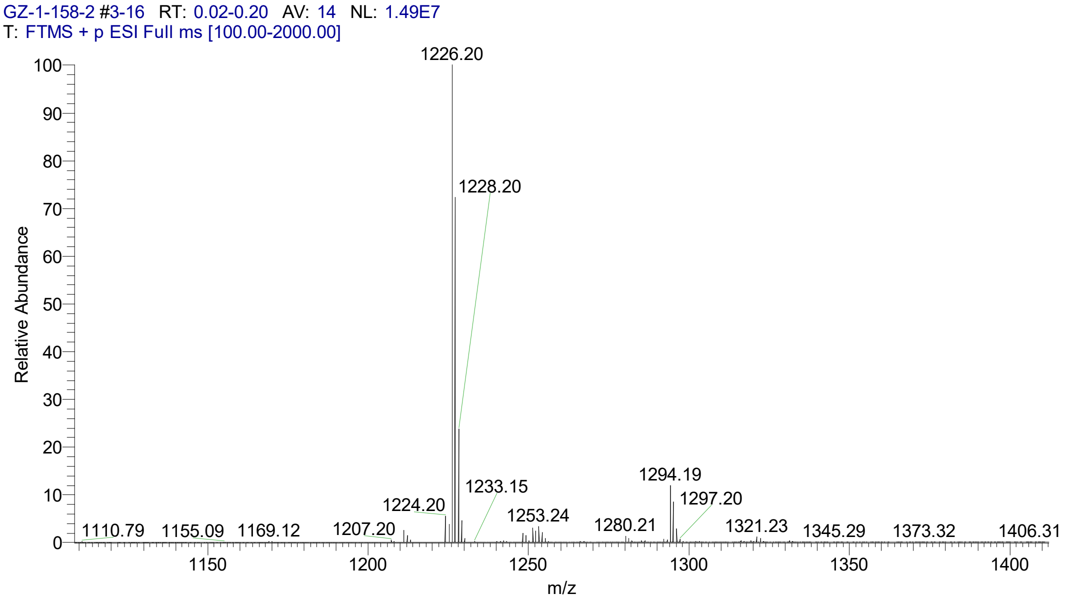

Figure S33. ESI-MS spectrum of [**Fe(PFHPX-COOH)OH**] in CH_3CN.

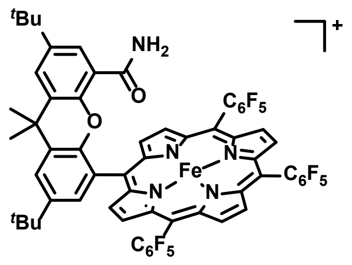

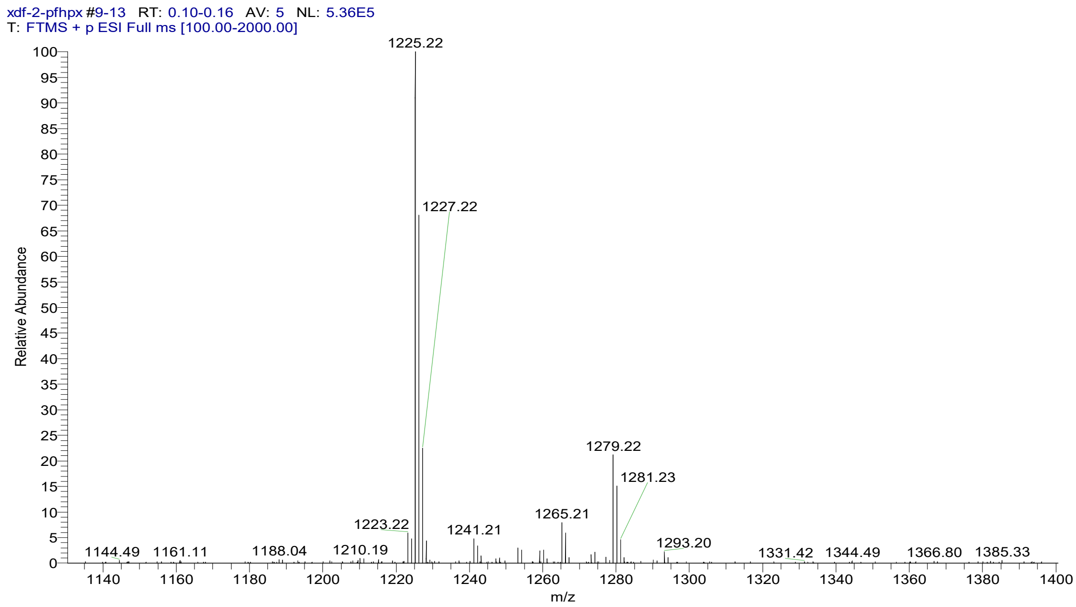

Figure S34. ESI-MS spectrum of [**Fe(PFHPX-CONH$_2$)OH**] in CH_3CN.

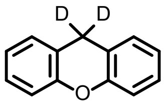

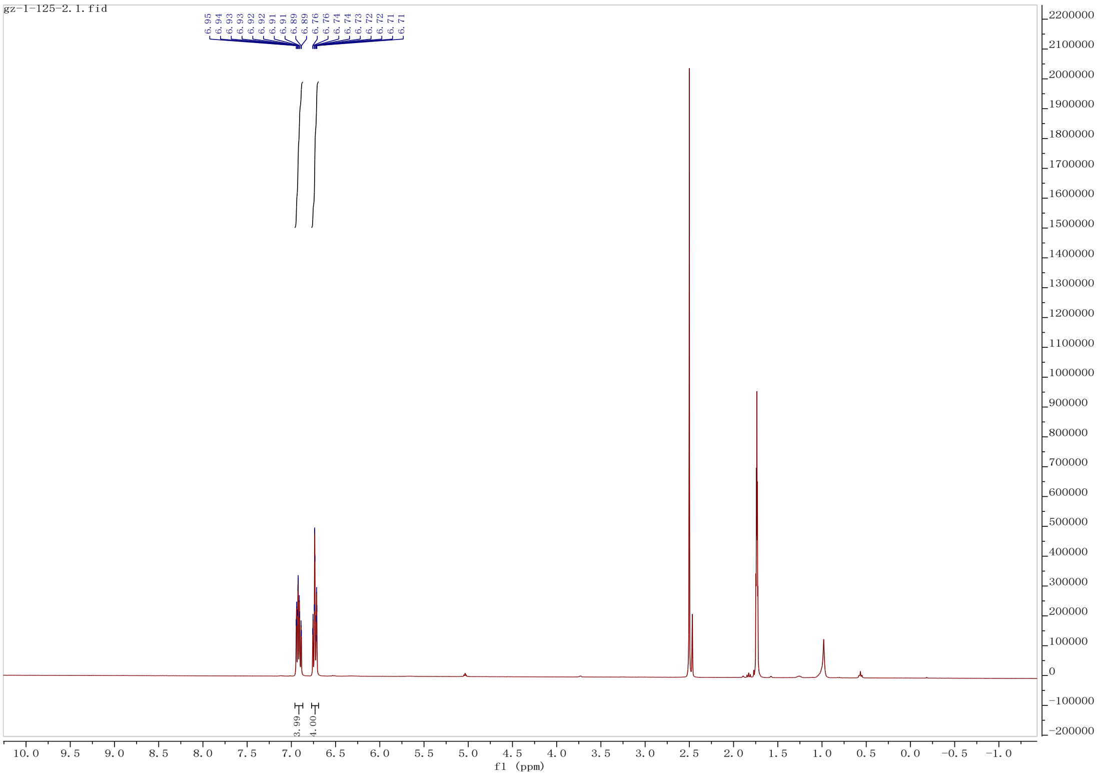

Figure S35. ^{1}H NMR spectrum of **Xanthene** in DMSO-d_6.

Computational Details

All density functional theory (DFT) calculations were carried out using the Gaussian 16 software package.[S7] All geometries were optimized using the B3LYP hybrid functional[S8, S9] with a basis set of 6-31G(d) for all light atoms and LANL2DZ for Fe, together with Grimme's D3(BJ) dispersion correction (GD3BJ).[S8] Frequencies were calculated for all the stationary points to confirm if each optimized structure is a local minimum on the respective potential energy surface or a transition state structure with only one imaginary frequency. Solvation energy correction was calculated in acetonitrile solvent with the SMD continuum solvation model[S10] based on the gas phase optimized geometries. The same functional with basis set 6-311G(d,p) for all light atoms and SDD for Fe were used for single point energy calculations.

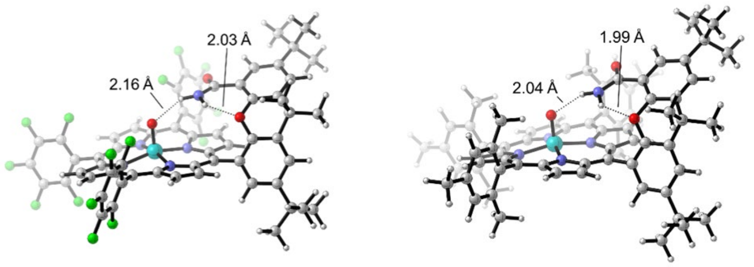

1-CONH$_2$ **2-CONH$_2$**

Figure S36. Structures of **1-CONH$_2$** and **2-CONH$_2$** in the back view.

Free Energy Profile

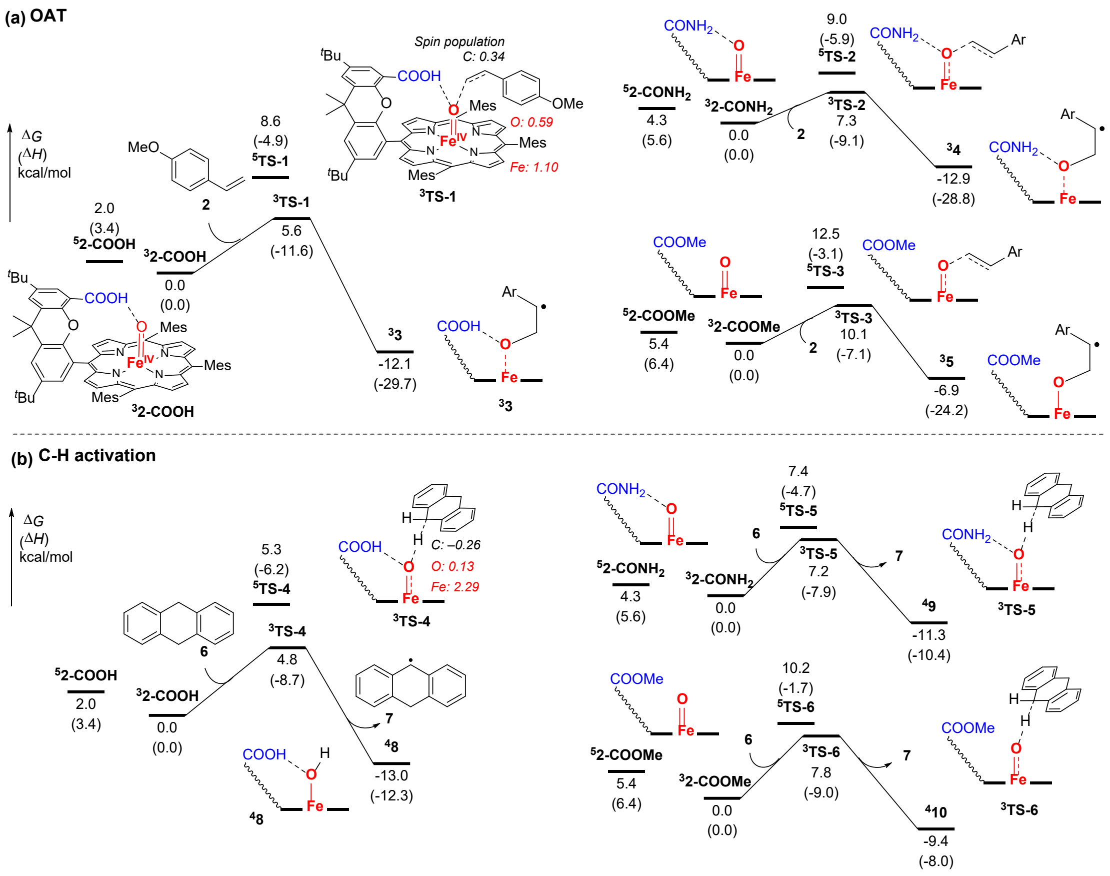

Figure S37. Free energy profile including quintet state iron(IV)-oxo porphyrin species.

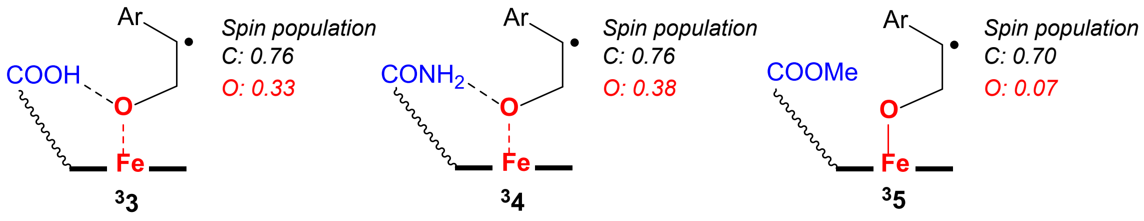

Figure S38. Spin population of **3**, **4** and **5**.

Due to intramolecular H-bonding, intermediates **3** and **4** have a portion of spin populated on the O atom. This indicates that **3** and **4** may be closer to the epoxidation product on the potential energy surface compared to **5**.

Energy Diagram

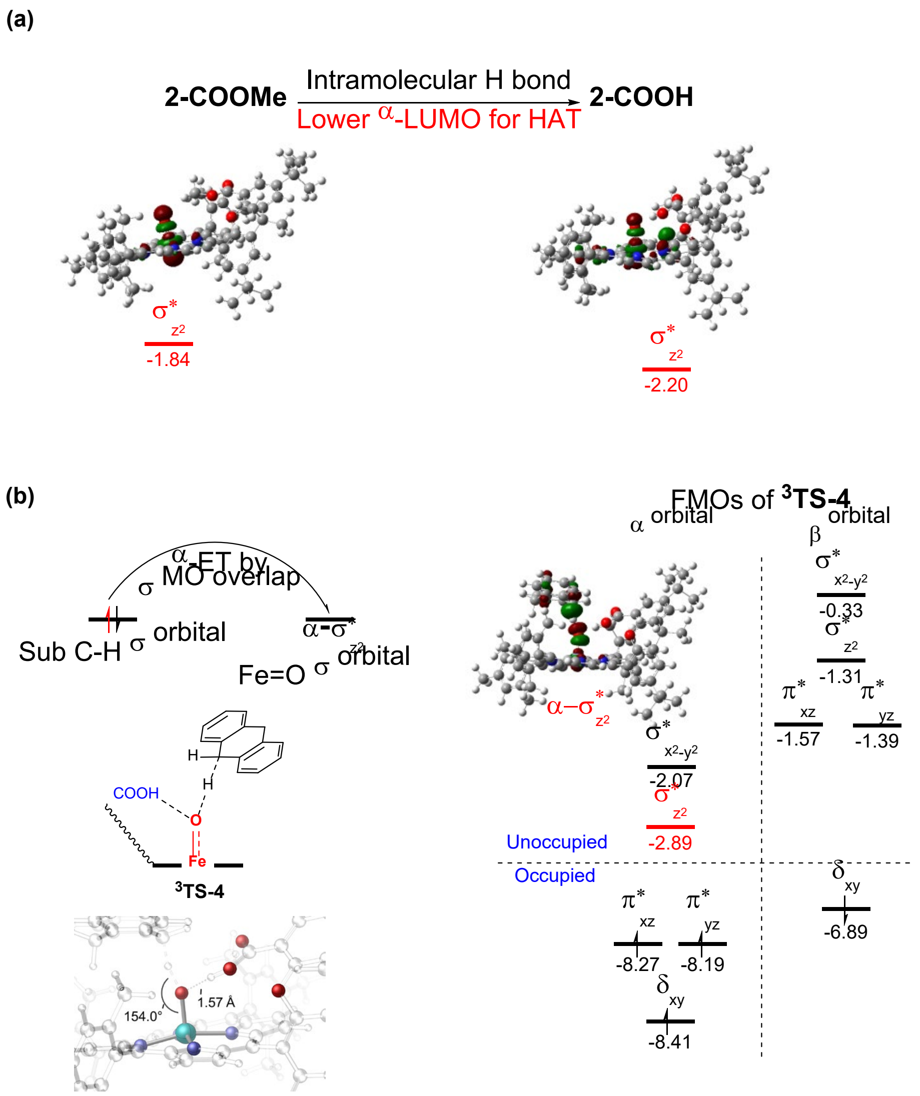

Figure S39. Energy diagram and FMOs HAT transition state ³TS-4

The C-H activation reaction occurs on a triplet potential energy surface as well. However, the energy barrier of the quintet transition state is only about 0.5 kcal/mol higher compared to triplet iron(IV)-oxo species, and the β electron in $\boldsymbol{\delta_{xy}}$ flips to a positive spin and transfers to the α manifold of antibonding $\boldsymbol{\sigma^*_{x^2-y^2}}$ orbital, resulting in 4 unpaired electrons in α molecular orbitals. Back to the potential energy surface of the triplet state, nonbonding $\boldsymbol{\delta_{xy}}$ is still the lowest of both α and β orbitals. Similarly, intramolecular H-bonding significantly reduces the energy of the antibonding $\boldsymbol{\sigma^*_{z^2}}$ orbital. As we can see in Figure S34(b), $\boldsymbol{\sigma^*_{z^2}}$ orbital interacts with the substrate C-H σ bond in the transition state, performing an α electron transfer through σ-style molecular orbital overlap. Hence, with either

reaction, we come to the same conclusion that intramolecular H-bonding stabilizes frontline antibonding orbitals, which will receive the electrons from the substrate, to lower the energy barrier.

Cartesian Coordinates and Energies of Optimized Structures

1-CONH$_2$-3tri
B3LYP SCF energy: -4506.17883951 a.u.
B3LYP enthalpy: -4505.219779 a.u.
B3LYP free energy: -4505.409049 a.u.
B3LYP SCF energy in solution: -4503.60484516 a.u.
B3LYP enthalpy in solution: -4502.645785 a.u.
B3LYP free energy in solution: -4502.835055 a.u.

Cartesian coordinates

ATOM	X	Y	Z
N	-3.291736	0.458154	0.096855
N	-1.879544	-1.918856	0.579055
N	0.455659	-0.445460	0.960112
N	-0.920568	1.924963	0.335327
C	-3.801327	1.682841	-0.287948
C	-5.223855	1.591375	-0.493046
C	-5.581424	0.310376	-0.215669
C	-4.373969	-0.397345	0.125903
C	-4.327687	-1.766880	0.363624
C	-3.142093	-2.471870	0.532983
C	-3.061034	-3.908628	0.620064
C	-1.740807	-4.227863	0.654454
C	-1.010869	-2.985961	0.646153
C	0.367669	-2.902031	0.799839
C	1.026388	-1.700980	1.042026
C	2.412534	-1.597686	1.417297
C	2.684794	-0.275799	1.561738
C	1.485354	0.442240	1.212343
C	1.451186	1.817722	1.006661
C	0.317613	2.490356	0.563449
C	0.290444	3.897598	0.255920
C	-0.970107	4.184678	-0.165609
C	-1.718378	2.954959	-0.116483
C	-3.068960	2.854593	-0.433249
H	-5.860547	2.405599	-0.805097
H	-6.566045	-0.130473	-0.262614
H	-3.907882	-4.577907	0.645183
H	-1.290475	-5.207034	0.723681
H	3.088340	-2.429075	1.535122
H	3.621369	0.184604	1.837084
H	1.133055	4.564747	0.358314
H	-1.369598	5.141556	-0.467527
Fe	-1.351153	-0.027627	0.223415
C	1.167600	-4.148364	0.673746
C	1.857619	-4.718991	1.743541
C	1.277756	-4.769087	-0.574932
C	2.653400	-5.848890	1.583390
C	2.066452	-5.900477	-0.756814
C	2.757012	-6.438920	0.326038
C	-3.769756	4.079525	-0.909192
C	-4.681953	4.761498	-0.100450
C	-3.526107	4.598707	-2.183359
C	-5.336958	5.909124	-0.538586
C	-4.163211	5.749429	-2.640413
C	-5.073267	6.404309	-1.813687
C	-5.612944	-2.519473	0.372667
C	-6.515195	-2.383876	1.430709
C	-5.972503	-3.372025	-0.674236
C	-7.727563	-3.067702	1.454386
C	-7.176077	-4.072260	-0.668892
C	-8.056132	-3.916460	0.399741
O	-1.014442	-0.217876	-1.341330
C	2.726672	2.576703	1.150354
C	3.764940	2.362465	0.242110
C	2.937215	3.483002	2.196899
C	5.012580	2.975008	0.379760
C	4.161530	4.135935	2.366177
H	2.122723	3.643809	2.892528
C	4.602166	0.671331	-1.154343
C	6.065456	2.658964	-0.683574
C	5.187577	3.851950	1.449364
C	4.345309	-0.642762	-1.556787
C	5.904011	1.179398	-1.051862
H	6.149199	4.335052	1.574890
C	5.446463	-1.464194	-1.835206
C	6.962148	0.322336	-1.343592
C	6.760487	-1.011642	-1.735467
H	5.215797	-2.483594	-2.114738
H	7.975272	0.698334	-1.265357
C	4.420719	5.126503	3.509405
C	4.799918	6.500898	2.915716
C	3.186513	5.314020	4.407082
C	5.581789	4.605597	4.384268
H	5.700617	6.441222	2.296267
H	3.988884	6.892244	2.291512
H	4.992750	7.222360	3.718298
H	2.886282	4.375856	4.886555
H	3.415089	6.033169	5.200782
H	2.330296	5.702124	3.844191
H	5.783427	5.304147	5.204793
H	5.334548	3.630380	4.817864
H	6.505476	4.491843	3.807684
O	3.536888	1.492078	-0.807748
C	7.971462	-1.905637	-2.032790
C	8.858619	-1.998928	-0.772213
C	7.553874	-3.329412	-2.435673
C	8.787531	-1.292463	-3.191563
H	9.222791	-1.015395	-0.457160
H	8.300275	-2.433859	0.064110
H	9.732225	-2.632233	-0.966811
H	6.939546	-3.330544	-3.342361
H	8.446664	-3.931475	-2.636737
H	6.988494	-3.826311	-1.639966
H	9.659758	-1.917650	-3.416363
H	8.177563	-1.216760	-4.098395
H	9.150414	-0.288688	-2.946377
C	2.985831	-1.292868	-1.678752
O	2.891354	-2.521172	-1.637959
C	7.484380	2.988297	-0.203817
H	7.755247	2.411365	0.686446
H	8.215041	2.777869	-0.989847
H	7.573923	4.052938	0.030955
C	5.753967	3.510398	-1.944191
H	5.826173	4.577765	-1.707211
H	6.468054	3.276971	-2.741314
H	4.746157	3.307714	-2.316804
N	1.924873	-0.477624	-1.889600
H	0.987599	-0.864515	-1.846603
H	2.007712	0.514528	-1.725112
F	0.608938	-4.287200	-1.621715
F	2.163748	-6.476030	-1.958723
F	3.514111	-7.526944	0.161664
F	3.308166	-6.374894	2.626055
F	1.774325	-4.168668	2.965870
F	-5.149585	-3.534548	-1.716858
F	-7.495286	-4.883548	-1.681831
F	-9.213715	-4.579830	0.413006
F	-8.572430	-2.919862	2.479589
F	-6.223268	-1.573969	2.456392
F	-2.656329	3.989713	-2.997071
F	-3.911979	6.225821	-3.863566
F	-5.691926	7.506053	-2.242561
F	-6.207310	6.540954	0.255500
F	-4.949262	4.310638	1.132350

1-CONH$_2$-5qui
B3LYP SCF energy: -4506.15958306 a.u.
B3LYP enthalpy: -4505.202341 a.u.
B3LYP free energy: -4505.392981 a.u.
B3LYP SCF energy in solution: -4503.59001924 a.u.
B3LYP enthalpy in solution: -4502.632777 a.u.
B3LYP free energy in solution: -4502.823417 a.u.

Cartesian coordinates

ATOM	X	Y	Z
N	-3.365424	0.389189	0.107137
N	-1.821267	-1.988617	0.601709
N	0.528399	-0.379606	0.965402
N	-0.986137	1.989748	0.345522
C	-3.924812	1.615665	-0.172812
C	-5.341377	1.464809	-0.413839
C	-5.627694	0.141411	-0.281886
C	-4.387466	-0.529097	0.034815
C	-4.253781	-1.911788	0.206033
C	-3.053408	-2.584125	0.456318
C	-2.920672	-4.017389	0.587894
C	-1.598341	-4.278521	0.772271
C	-0.914285	-3.006026	0.780860
C	0.458598	-2.844371	0.996962
C	1.111402	-1.614772	1.143376
C	2.509839	-1.453363	1.468554
C	2.765183	-0.118945	1.469208
C	1.536303	0.548836	1.103351
C	1.434193	1.919207	0.831065
C	0.252758	2.578481	0.469390
C	0.152290	3.992213	0.187274
C	-1.155559	4.249826	-0.090855
C	-1.861103	2.992660	-0.005106
C	-3.231537	2.828599	-0.236529
H	-6.022717	2.265374	-0.661306
H	-6.586258	-0.341231	-0.402448
H	-3.734515	-4.726318	0.551724
H	-1.126313	-5.238751	0.919284
H	3.209163	-2.255480	1.641583
H	3.704649	0.372963	1.670391
H	0.976646	4.688953	0.211285
H	-1.603804	5.203043	-0.329570
Fe	-1.320289	-0.047961	0.060301
C	1.297559	-4.073478	1.007403
C	1.979489	-4.507191	2.145020
C	1.463448	-4.811249	-0.169694
C	2.816698	-5.617926	2.119071
C	2.295329	-5.925537	-0.216988
C	2.974403	-6.326995	0.930637
C	-4.016821	4.048117	-0.582254
C	-4.926392	4.604508	0.320186
C	-3.870959	4.678485	-1.820526
C	-5.667672	5.740938	0.008418
C	-4.596240	5.819949	-2.151905
C	-5.498769	6.350423	-1.232810
C	-5.490109	-2.736559	0.088102
C	-6.486938	-2.688182	1.065827
C	-5.699838	-3.582774	-1.003892
C	-7.649326	-3.447767	0.967090
C	-6.851409	-4.356790	-1.120641
C	-7.829145	-4.286185	-0.130823
O	-0.956159	-0.244325	-1.522577
C	2.694561	2.714667	0.884009
C	3.727449	2.456640	-0.020127
C	2.897999	3.702167	1.857520
C	4.959503	3.111794	0.052637
C	4.105957	4.396485	1.959353
H	2.090856	3.891979	2.554169
C	4.599540	0.686452	-1.292147
C	6.008019	2.744541	-0.998434
C	5.126054	4.071268	1.049838
C	4.377405	-0.661989	-1.586957
C	5.887170	1.236539	-1.244945
H	6.076074	4.586814	1.123153
C	5.498754	-1.472967	-1.808451
C	6.967074	0.388431	-1.477698
C	6.800370	-0.977961	-1.758168
H	5.294655	-2.517478	-2.003340
H	7.969601	0.797577	-1.440623
C	4.353950	5.475050	3.022458
C	4.688614	6.810812	2.323205
C	3.127129	5.697126	3.922376
C	5.539920	5.049945	3.915491
H	5.582445	6.728580	1.696438
H	3.859369	7.133929	1.684047
H	4.872586	7.594477	3.067398
H	2.858803	4.790220	4.475261
H	3.346651	6.479589	4.656493
H	2.253198	6.019412	3.345278
H	5.733546	5.811986	4.679454
H	5.324486	4.103274	4.422995
H	6.458648	4.917829	3.334883
O	3.513818	1.502504	-0.998011
C	8.033696	-1.859370	-1.995107
C	8.934498	-1.824632	-0.741231
C	7.653269	-3.322325	-2.275830
C	8.821530	-1.320619	-3.209091
H	9.274290	-0.809108	-0.512137
H	8.396037	-2.204021	0.134254
H	9.823413	-2.448204	-0.893200
H	7.031322	-3.414562	-3.172565
H	8.561011	-3.913941	-2.436959
H	7.109193	-3.767805	-1.436033
H	9.709393	-1.937474	-3.391797
H	8.201743	-1.336509	-4.112271
H	9.157479	-0.290385	-3.050655
C	3.034842	-1.353590	-1.651300
O	2.966876	-2.574327	-1.495159
C	7.422302	3.152986	-0.568449
H	7.722378	2.658054	0.360972
H	8.148447	2.900894	-1.346362
H	7.482877	4.235299	-0.421599
C	5.654440	3.483329	-2.317599
H	5.697227	4.567777	-2.166849
H	6.365129	3.208247	-3.104392
H	4.648635	3.222328	-2.657730
N	1.960818	-0.584010	-1.945666
H	1.028611	-0.980014	-1.884449
H	2.022345	0.421028	-1.881682
F	0.807498	-4.464043	-1.276080

F	2.444413	-6.614941	-1.351723
F	3.772103	-7.397447	0.895007
F	3.461150	-6.011776	3.224254
F	1.846183	-3.838682	3.301507
F	-4.779752	-3.665657	-1.971535
F	-7.027605	-5.158652	-2.174978
F	-8.937860	-5.021012	-0.234372
F	-8.587254	-3.382660	1.917181
F	-6.338138	-1.891778	2.132031
F	-3.012972	4.188209	-2.722709
F	-4.437206	6.404717	-3.343082
F	-6.201022	7.442245	-1.541030
F	-6.531712	6.252730	0.890609
F	-5.105397	4.040037	1.521237

1-COOH-3tri

B3LYP SCF energy: -4526.04024803 a.u.
B3LYP enthalpy: -4525.094452 a.u.
B3LYP free energy: -4525.283643 a.u.
B3LYP SCF energy in solution: -4523.45214171 a.u.
B3LYP enthalpy in solution: -4522.506346 a.u.
B3LYP free energy in solution: -4522.695537 a.u.

Cartesian coordinates

ATOM	X	Y	Z
N	-3.210598	0.552127	0.105870
N	-1.883651	-1.867205	0.608029
N	0.497678	-0.474773	0.989832
N	-0.807945	1.939051	0.416711
C	-3.668460	1.786946	-0.311266
C	-5.084069	1.734888	-0.568093
C	-5.491460	0.470437	-0.282043
C	-4.319755	-0.268745	0.111512
C	-4.324739	-1.633687	0.377055
C	-3.165935	-2.375296	0.573140
C	-3.136001	-3.811546	0.687395
C	-1.827753	-4.177625	0.721160
C	-1.053311	-2.964193	0.684431
C	0.329572	-2.926535	0.811468
C	1.033970	-1.748343	1.029664
C	2.430628	-1.691165	1.369936
C	2.739638	-0.382120	1.556420
C	1.550627	0.377955	1.267540
C	1.539824	1.764578	1.156124
C	0.428288	2.473764	0.722751
C	0.436455	3.892177	0.477843
C	-0.794061	4.217177	-0.000350
C	-1.563751	3.000132	-0.036361
C	-2.898845	2.938360	-0.420822
H	-5.683720	2.563234	-0.914449
H	-6.487378	0.059574	-0.355772
H	-4.006252	-4.449356	0.727000
H	-1.413821	-5.171696	0.804434
H	3.087024	-2.543190	1.447452
H	3.692949	0.045113	1.827353
H	1.285274	4.536903	0.650743
H	-1.162836	5.191110	-0.286428
Fe	-1.290750	0.004583	0.254112
C	1.080810	-4.205617	0.713160
C	1.745984	-4.770670	1.802882
C	1.152415	-4.881408	-0.510088
C	2.481310	-5.946229	1.686072
C	1.880187	-6.060640	-0.647492
C	2.547470	-6.591566	0.453627
C	-3.542522	4.184939	-0.919572
C	-4.477781	4.884509	-0.153215
C	-3.216572	4.708849	-2.173467
C	-5.077348	6.053439	-0.613635
C	-3.797825	5.880364	-2.651821
C	-4.732821	6.552413	-1.867816
C	-5.635500	-2.340429	0.380949
C	-6.551242	-2.144221	1.417858
C	-6.007588	-3.206408	-0.650604
C	-7.788063	-2.782894	1.436239
C	-7.236043	-3.861990	-0.649994
C	-8.128718	-3.646457	0.397584
O	-0.960835	-0.191782	-1.316308
C	2.840554	2.476436	1.334467
C	3.816371	2.311247	0.350327
C	3.161155	3.229292	2.468472
C	5.117145	2.793913	0.496690
C	4.441146	3.768471	2.645127
H	2.397154	3.356086	3.225916
C	4.406367	0.748445	-1.273870
C	6.092563	2.505113	-0.647566
C	5.405519	3.518627	1.653070
C	4.001294	-0.482547	-1.801715
C	5.759893	1.104998	-1.183804
H	6.408666	3.905123	1.790683
C	4.991063	-1.376967	-2.231266
C	6.704973	0.186092	-1.633152
C	6.348155	-1.067190	-2.161910
H	4.648317	-2.333081	-2.604840
H	7.755225	0.445822	-1.570204
C	4.826912	4.593103	3.881236
C	5.293996	5.995334	3.433040
C	3.649749	4.767492	4.855004
C	5.974984	3.884274	4.632557
H	6.161146	5.940686	2.767009
H	4.493670	6.519504	2.898984
H	5.577539	6.598976	4.303229
H	3.294713	3.805318	5.240246
H	3.967975	5.370098	5.712405
H	2.805343	5.281759	4.382742
H	6.265523	4.462281	5.517740
H	5.665715	2.886144	4.961784
H	6.862820	3.769317	4.002400
O	3.453885	1.602468	-0.769749
C	7.445991	-2.032777	-2.627545
C	8.377149	-2.359223	-1.439566
C	6.866593	-3.352631	-3.162569
C	8.265685	-1.369896	-3.756001
H	8.854500	-1.459472	-1.037503
H	7.816986	-2.833814	-0.626293
H	9.171298	-3.046294	-1.754878
H	6.213181	-3.188223	-4.026119
H	7.682465	-4.009837	-3.482427
H	6.292324	-3.883681	-2.395621
H	9.058041	-2.045436	-4.099542
H	7.625102	-1.129905	-4.611668
H	8.740124	-0.441367	-3.421423
C	2.579377	-0.948421	-1.856541
O	2.281168	-2.131424	-1.832513
O	1.696746	0.047581	-1.943855
H	0.771317	-0.289358	-1.836665
C	7.553821	2.623675	-0.197840
H	7.790032	1.913113	0.601085
H	8.232354	2.442209	-1.036355
H	7.764127	3.634634	0.163452
C	5.828995	3.532362	-1.781194
H	6.022061	4.549548	-1.422266
H	6.485483	3.328827	-2.634265
H	4.792462	3.478862	-2.125354
F	-6.248196	-1.319196	2.428037
F	-8.644997	-2.577897	2.441367
F	-9.309897	-4.266889	0.406003
F	-7.566903	-4.687150	-1.647897
F	-5.173491	-3.425357	-1.673776
F	3.246176	-7.722686	0.330097
F	0.505402	-4.411394	-1.575215
F	1.939341	-6.688963	-1.825648
F	3.113553	-6.462558	2.746489
F	1.694478	-4.173014	3.003300
F	-5.298241	7.674266	-2.317574
F	-5.971248	6.702075	0.139823
F	-4.821539	4.431220	1.059699
F	-3.469496	6.360497	-3.854980
F	-2.321865	4.083487	-2.945829

1-COOH-5qui

B3LYP SCF energy: -4526.02197680 a.u.
B3LYP enthalpy: -4525.078299 a.u.
B3LYP free energy: -4525.268784 a.u.
B3LYP SCF energy in solution: -4523.43932923 a.u.
B3LYP enthalpy in solution: -4522.495651 a.u.
B3LYP free energy in solution: -4522.686136 a.u.

Cartesian coordinates

ATOM	X	Y	Z
N	-3.288861	0.444873	0.110271
N	-1.780628	-1.953286	0.629191
N	0.594953	-0.369926	0.971022
N	-0.897503	2.020814	0.400715
C	-3.828618	1.677395	-0.181061
C	-5.235648	1.539225	-0.477834
C	-5.537181	0.217457	-0.364169
C	-4.315658	-0.463973	-0.002624
C	-4.202828	-1.846897	0.184681
C	-3.019056	-2.532518	0.476926
C	-2.913517	-3.963873	0.647431
C	-1.599389	-4.241106	0.863960
C	-0.894209	-2.980102	0.845429
C	0.481511	-2.833587	1.055724
C	1.160884	-1.614471	1.147505
C	2.568759	-1.474655	1.434245
C	2.845557	-0.144062	1.428650
C	1.616146	0.544117	1.104953
C	1.516487	1.928513	0.904777
C	0.336936	2.604363	0.579050
C	0.241384	4.031219	0.376295
C	-1.059312	4.302837	0.080679
C	-1.763873	3.041098	0.082538
C	-3.127552	2.888254	-0.194512
H	-5.900695	2.346456	-0.746835
H	-6.494266	-0.256781	-0.523467
H	-3.738166	-4.660300	0.613545
H	-1.147326	-5.205346	1.043740
H	3.258391	-2.288010	1.597088
H	3.796864	0.333487	1.606968
H	1.066916	4.722632	0.457468
H	-1.504425	5.266787	-0.117266
Fe	-1.248368	-0.018251	0.063653
C	1.293723	-4.077754	1.150350
C	1.933389	-4.454238	2.333223
C	1.461747	-4.902133	0.032250
C	2.729295	-5.593117	2.407763
C	2.252450	-6.047388	0.086828
C	2.888388	-6.391057	1.277285
C	-3.897309	4.119727	-0.531187
C	-4.846166	4.645560	0.349066
C	-3.695941	4.793008	-1.738998
C	-5.572398	5.793565	0.044864
C	-4.406261	5.946244	-2.061970
C	-5.348543	6.445710	-1.165647
C	-5.445536	-2.657581	0.041327
C	-6.474045	-2.576496	0.983576
C	-5.628949	-3.523922	-1.039762
C	-7.642049	-3.323763	0.860971
C	-6.786241	-4.285445	-1.179851
C	-7.795686	-4.182312	-0.225332
O	-0.920988	-0.237097	-1.538454
C	2.792455	2.700230	0.973968
C	3.760965	2.469497	-0.004639
C	3.102341	3.573527	2.022834
C	5.048348	3.002158	0.077065
C	4.365305	4.167008	2.126202
H	2.345006	3.748369	2.777313
C	4.379612	0.777449	-1.481725
C	6.022937	2.627257	-1.042808
C	5.326520	3.846970	1.151200
C	4.000728	-0.505758	-1.888652
C	5.723773	1.172959	-1.434694
H	6.318767	4.274524	1.235833
C	5.008177	-1.416842	-2.230686
C	6.688572	0.235600	-1.796592
C	6.358654	-1.071495	-2.196649
H	4.686462	-2.412140	-2.508787
H	7.732808	0.523730	-1.765554
C	4.737149	5.124412	3.267167
C	5.150886	6.488202	2.672033
C	3.565681	5.358163	4.235301
C	5.917134	4.533735	4.069368
H	6.011751	6.394393	2.002237
H	4.327171	6.928591	2.099276
H	5.423487	7.186442	3.472136
H	3.248127	4.429108	4.721361
H	3.872556	6.055162	5.022392
H	2.698858	5.793558	3.725734
H	6.197264	5.207950	4.887268
H	5.646112	3.564497	4.502242
H	6.801496	4.385109	3.441377
O	3.407352	1.649980	-1.048375
C	7.477032	-2.053694	-2.569762
C	8.417642	-2.241148	-1.359359
C	6.926384	-3.432272	-2.970073
C	8.279498	-1.487236	-3.761357
H	8.876077	-1.296040	-1.050039
H	7.869686	-2.645023	-0.500821
H	9.225842	-2.938830	-1.608767
H	6.267737	-3.367846	-3.842821
H	7.756144	-4.099540	-3.227261
H	6.365635	-3.897975	-2.152321
H	9.086004	-2.175753	-4.040202
H	7.631960	-1.346730	-4.633736
H	8.733860	-0.520101	-3.521749
C	2.583779	-0.991665	-1.902462
O	2.295209	-2.162437	-1.714738
O	1.702558	-0.025059	-2.152420

H	0.769826	-0.341566	-2.013641
C	7.483647	2.827361	-0.621123
H	7.743785	2.207531	0.243241
H	8.160375	2.578418	-1.443602
H	7.670620	3.874673	-0.366179
C	5.724154	3.529927	-2.269916
H	5.893988	4.582232	-2.015717
H	6.379007	3.258738	-3.105207
H	4.686742	3.416958	-2.596503
F	-5.077749	4.040385	1.520971
F	-6.474284	6.275840	0.905505
F	-6.036487	7.548558	-1.466283
F	-4.195124	6.571717	-3.223800
F	-2.799864	4.332845	-2.618929
F	-6.350428	-1.760978	2.038397
F	-8.610186	-3.227941	1.777475
F	-8.909652	-4.905403	-0.351450
F	-6.937434	-5.106556	-2.223063
F	-4.678330	-3.638098	-1.973635
F	1.795669	-3.704138	3.436395
F	3.333161	-5.928462	3.553513
F	3.646686	-7.488113	1.337118
F	2.402724	-6.820387	-0.993012
F	0.850406	-4.612201	-1.115028

1-COOMe-3tri

B3LYP SCF energy: -4565.34557198 a.u.
B3LYP enthalpy: -4564.369413 a.u.
B3LYP free energy: -4564.563270 a.u.
B3LYP SCF energy in solution: -4562.72061907 a.u.
B3LYP enthalpy in solution: -4561.744460 a.u.
B3LYP free energy in solution: -4561.938317 a.u.

Cartesian coordinates

ATOM	X	Y	Z
N	-3.394940	0.353721	0.114060
N	-1.870552	-1.988518	0.431270
N	0.437074	-0.425693	0.667918
N	-1.047989	1.893686	0.144112
C	-3.971958	1.572743	-0.179634
C	-5.405235	1.443459	-0.248951
C	-5.697038	0.139951	-0.002094
C	-4.441846	-0.541006	0.194033
C	-4.329839	-1.919401	0.342871
C	-3.111832	-2.587044	0.406782
C	-2.976371	-4.022373	0.442349
C	-1.644820	-4.293325	0.449731
C	-0.961378	-3.025093	0.462950
C	0.411478	-2.889918	0.622655
C	1.033983	-1.661289	0.819454
C	2.409673	-1.509702	1.217782
C	2.648009	-0.176324	1.306644
C	1.441612	0.496431	0.897641
C	1.365947	1.865476	0.659229
C	0.191794	2.491029	0.246855
C	0.102939	3.886594	-0.103603
C	-1.198695	4.136341	-0.407177
C	-1.911091	2.895097	-0.245849
C	-3.287596	2.762438	-0.397461
H	-6.092018	2.248312	-0.464332
H	-6.668289	-0.331469	0.018803
H	-3.797266	-4.723457	0.463774
H	-1.158288	-5.256968	0.486636
H	3.100355	-2.319424	1.391419
H	3.564493	0.315934	1.592493
H	0.935578	4.573290	-0.109569
H	-1.645550	5.075280	-0.698758
Fe	-1.439958	-0.067228	0.069959
C	1.261575	-4.111568	0.582378
C	1.867702	-4.635460	1.724934
C	1.513204	-4.751747	-0.633515
C	2.710391	-5.741191	1.666136
C	2.350171	-5.859924	-0.716841
C	2.950675	-6.353765	0.438338
C	-4.069825	3.970513	-0.781833
C	-4.884640	4.634449	0.137976
C	-4.007314	4.488871	-2.077666
C	-5.617273	5.765315	-0.212032
C	-4.724494	5.623358	-2.448837
C	-5.533438	6.261158	-1.511137
C	-5.583186	-2.722983	0.388531
C	-6.418687	-2.688836	1.507657
C	-5.976261	-3.528818	-0.682898
C	-7.599232	-3.424410	1.565876
C	-7.148177	-4.279837	-0.644396
C	-7.962244	-4.224263	0.484686
O	-1.291003	-0.208150	-1.524864
C	2.589597	2.692268	0.851843
C	3.752477	2.470609	0.106679
C	2.610672	3.699883	1.827738
C	4.925494	3.195167	0.343419
C	3.754710	4.455951	2.088499
H	1.704284	3.857614	2.398790
C	4.876166	0.763622	-1.033332
C	6.126839	2.883604	-0.547433
C	4.905460	4.173304	1.335684
C	4.792783	-0.590241	-1.385511
C	6.117568	1.372376	-0.792544
H	5.809623	4.737987	1.528994
C	5.975056	-1.343822	-1.428863
C	7.263755	0.583983	-0.855063
C	7.222274	-0.786522	-1.158534
H	5.866867	-2.390971	-1.678833
H	8.223959	1.047375	-0.662764
C	3.795131	5.557728	3.155674
C	4.154897	6.901734	2.485282
C	2.444185	5.723212	3.871080
C	4.864649	5.207562	4.213012
H	5.130588	6.860297	1.990354
H	3.407606	7.172499	1.731076
H	4.192480	7.702672	3.232961
H	2.147813	4.808612	4.396274
H	2.517495	6.523600	4.615060
H	1.644755	5.992125	3.171648
H	4.910187	5.987633	4.982039
H	4.629469	4.256557	4.703378
H	5.861090	5.118149	3.768223
O	3.720055	1.496350	-0.870375
C	8.523194	-1.598534	-1.192340
C	9.203131	-1.528337	0.192445
C	8.272997	-3.076712	-1.533087
C	9.469127	-1.006919	-2.260013
H	9.442569	-0.498115	0.476049
H	8.551139	-1.946812	0.967103
H	10.138827	-2.099705	0.186974
H	7.816710	-3.193629	-2.521984
H	9.224648	-3.619021	-1.539582
H	7.620374	-3.557474	-0.796240
H	10.406493	-1.574522	-2.295527
H	9.007325	-1.045791	-3.252675
H	9.719386	0.037703	-2.046990
C	3.540116	-1.320015	-1.746002
O	3.435026	-2.533584	-1.670543
O	2.585601	-0.516593	-2.224846
C	7.445507	3.369383	0.066476
H	7.641128	2.896976	1.034754
H	8.284021	3.153698	-0.601842
H	7.427967	4.454009	0.206508
C	5.919671	3.596208	-1.911620
H	5.883946	4.681885	-1.768094
H	6.745300	3.357035	-2.590809
H	4.984860	3.280070	-2.382510
C	1.396757	-1.169999	-2.711593
H	0.966038	-1.809135	-1.944941
H	0.704943	-0.368150	-2.955654
H	1.642311	-1.772553	-3.590760
F	0.938224	-4.306428	-1.754544
F	2.579547	-6.452740	-1.891895
F	3.754673	-7.417859	0.371008
F	3.283282	-6.221387	2.776146
F	1.657362	-4.058013	2.917593
F	-5.216378	-3.596310	-1.782543
F	-7.500131	-5.044913	-1.682409
F	-9.089552	-4.936879	0.530490
F	-8.380547	-3.372948	2.649362
F	-6.092636	-1.928130	2.560459
F	-3.238273	3.895546	-2.997553
F	-4.646446	6.100064	-3.695147
F	-6.227803	7.347146	-1.856964
F	-6.391484	6.380370	0.687972
F	-4.979870	4.180992	1.394954

1-COOMe-5qui

B3LYP SCF energy: -4565.32631994 a.u.
B3LYP enthalpy: -4564.351933 a.u.
B3LYP free energy: -4564.546617 a.u.
B3LYP SCF energy in solution: -4562.70606965 a.u.
B3LYP enthalpy in solution: -4561.731683 a.u.
B3LYP free energy in solution: -4561.926367 a.u.

Cartesian coordinates

ATOM	X	Y	Z
N	-3.457525	0.341644	0.150569
N	-1.849548	-2.029709	0.446162
N	0.501077	-0.396116	0.679011
N	-1.067963	1.958549	0.201784
C	-4.042053	1.566701	-0.069951
C	-5.471777	1.406461	-0.214311
C	-5.735188	0.076414	-0.105129
C	-4.468674	-0.588927	0.105468
C	-4.302492	-1.975972	0.190744
C	-3.078350	-2.638965	0.334122
C	-2.921104	-4.073308	0.418963
C	-1.590783	-4.320073	0.566042
C	-0.924552	-3.038890	0.588984
C	0.443456	-2.857980	0.813624
C	1.083169	-1.619081	0.928400
C	2.465590	-1.436882	1.306847
C	2.709745	-0.100316	1.276619
C	1.493460	0.544325	0.836527
C	1.376909	1.913355	0.559500
C	0.175032	2.552745	0.223126
C	0.045108	3.957307	-0.094633
C	-1.281164	4.204467	-0.278730
C	-1.973490	2.950140	-0.100666
C	-3.359677	2.781041	-0.198388
H	-6.175822	2.205606	-0.393718
H	-6.694539	-0.413961	-0.179828
H	-3.725709	-4.793097	0.389451
H	-1.102791	-5.277009	0.680000
H	3.160368	-2.227353	1.543181
H	3.632226	0.405909	1.515690
H	0.864549	4.656618	-0.160989
H	-1.748914	5.149816	-0.511054
Fe	-1.422705	-0.065373	-0.080209
C	1.298428	-4.075283	0.909139
C	1.877493	-4.480512	2.112605
C	1.584956	-4.829524	-0.231439
C	2.724956	-5.581910	2.184044
C	2.427452	-5.935739	-0.184192
C	2.999301	-6.310564	1.028955
C	-4.178363	3.995431	-0.479285
C	-4.999278	4.556636	0.501641
C	-4.157875	4.613290	-1.732127
C	-5.773608	5.686519	0.252751
C	-4.917998	5.748315	-2.002131
C	-5.729579	6.284178	-1.004754
C	-5.530110	-2.817061	0.103758
C	-6.471036	-2.834170	1.136431
C	-5.786853	-3.616192	-1.013476
C	-7.624347	-3.610647	1.066180
C	-6.929836	-4.406300	-1.102886
C	-7.851585	-4.400687	-0.058421
O	-1.340561	-0.150211	-1.705450
C	2.605342	2.747900	0.670787
C	3.747132	2.489969	-0.096809
C	2.654815	3.803016	1.595488
C	4.918199	3.240417	0.057117
C	3.799992	4.578737	1.777947
H	1.769475	3.981957	2.192214
C	4.861296	0.730417	-1.164584
C	6.089248	2.895804	-0.860899
C	4.924257	4.269250	0.996519
C	4.791482	-0.645892	-1.419992
C	6.099461	1.372596	-1.008112
H	5.828436	4.851928	1.125369
C	5.985999	-1.380942	-1.453028
C	7.257614	0.601030	-1.056571
C	7.231298	-0.787152	-1.265128
H	5.888545	-2.444282	-1.627288
H	8.214787	1.092198	-0.928956
C	3.868379	5.731322	2.788334
C	4.190201	7.043816	2.040682
C	2.542695	5.920487	3.544057
C	4.979164	5.441703	3.821133
H	5.147634	6.986269	1.512832
H	3.413387	7.271390	1.302188
H	4.246966	7.880567	2.746745
H	2.274753	5.030397	4.123798
H	2.634978	6.757018	4.244887
H	1.715590	6.148131	2.862326
H	5.045621	6.259066	4.548765
H	4.771010	4.514309	4.365912
H	5.959371	5.337953	3.344494
O	3.698643	1.457474	-1.012215
C	8.544733	-1.578975	-1.289942
C	9.269431	-1.404994	0.062629
C	8.309986	-3.080427	-1.522689

C	9.443360	-1.045429	-2.426829
H	9.500080	-0.354396	0.268048
H	8.651275	-1.780067	0.885741
H	10.214322	-1.961042	0.062958
H	7.822457	-3.270403	-2.484968
H	9.270570	-3.606781	-1.526650
H	7.691610	-3.521038	-0.733103
H	10.389304	-1.598949	-2.456756
H	8.949454	-1.158515	-3.398128
H	9.681313	0.015061	-2.292757
C	3.545414	-1.424489	-1.691178
O	3.472184	-2.631890	-1.530350
O	2.559009	-0.676905	-2.196120
C	7.421131	3.443017	-0.333682
H	7.665102	3.037318	0.653701
H	8.236258	3.199492	-1.021065
H	7.387076	4.533940	-0.262323
C	5.813869	3.516813	-2.257453
H	5.761884	4.608584	-2.180811
H	6.617047	3.249877	-2.952992
H	4.868105	3.155032	-2.669752
C	1.382320	-1.391027	-2.624504
H	1.041809	-2.073495	-1.849233
H	0.628211	-0.630922	-2.810651
H	1.612459	-1.961021	-3.529653
F	1.037953	-4.500227	-1.405689
F	2.688785	-6.639676	-1.289036
F	3.808039	-7.371338	1.086821
F	3.270458	-5.947241	3.349876
F	1.634660	-3.790520	3.236731
F	-4.921173	-3.637121	-2.033556
F	-7.151296	-5.162022	-2.182741
F	-8.951887	-5.151568	-0.135116
F	-8.508216	-3.608225	2.069030
F	-6.275981	-2.086369	2.230047
F	-3.389984	4.117503	-2.709218
F	-4.879241	6.321579	-3.208958
F	-6.464546	7.369829	-1.253699
F	-6.550927	6.203113	1.209897
F	-5.058536	4.002663	1.719467

2

B3LYP SCF energy: -424.19738501 a.u.
B3LYP enthalpy: -424.020357 a.u.
B3LYP free energy: -424.065546 a.u.
B3LYP SCF energy in solution: -423.91087335 a.u.
B3LYP enthalpy in solution: -423.733845 a.u.
B3LYP free energy in solution: -423.779034 a.u.

Cartesian coordinates

ATOM	X	Y	Z
C	0.624862	1.461642	0.000087
C	-0.761832	1.425031	-0.000015
C	-1.470298	0.208793	-0.000131
C	-0.717299	-0.974813	-0.000242
C	0.675262	-0.957861	-0.000144
C	1.356736	0.267016	0.000022
H	1.166318	2.402022	0.000203
H	-1.317183	2.359800	0.000045
H	-1.223422	-1.935409	-0.000438
H	1.216690	-1.896504	-0.000200
C	-2.938396	0.233368	-0.000185
C	-3.774406	-0.812985	0.000360
H	-3.371166	1.233944	-0.000741
H	-4.850035	-0.667930	0.000194
H	-3.428080	-1.842982	0.000994
O	2.713884	0.398204	0.000164
C	3.496155	-0.783496	0.000023
H	3.307199	-1.391722	-0.894472
H	4.536843	-0.455017	0.000164
H	3.307058	-1.392006	0.894297

2-CONH$_2$-3tri

B3LYP SCF energy: -3371.71155359 a.u.
B3LYP enthalpy: -3370.377711 a.u.
B3LYP free energy: -3370.573260 a.u.
B3LYP SCF energy in solution: -3369.98746047 a.u.
B3LYP enthalpy in solution: -3368.653618 a.u.
B3LYP free energy in solution: -3368.849167 a.u.

Cartesian coordinates

ATOM	X	Y	Z
N	-3.452225	0.799544	-0.023001
N	-2.457565	-1.756425	0.551798
N	0.101469	-0.698887	0.860918
N	-0.899723	1.867312	0.332747
C	-3.738011	2.081770	-0.448546
C	-5.145917	2.216990	-0.720498
C	-5.719992	1.014440	-0.439699
C	-4.659429	0.129234	-0.030982
C	-4.852918	-1.216740	0.258938
C	-3.799257	-2.086384	0.514171
C	-3.968423	-3.505648	0.695294
C	-2.721709	-4.044184	0.789994
C	-1.785963	-2.952919	0.699813
C	-0.406604	-3.115953	0.793968
C	0.460386	-2.034944	0.893931
C	1.867857	-2.165425	1.169337
C	2.355302	-0.908700	1.347839
C	1.262338	0.004115	1.118793
C	1.406829	1.387998	1.073777
C	0.386675	2.241682	0.668416
C	0.570289	3.654205	0.447079
C	-0.598888	4.130431	-0.061741
C	-1.512338	3.018724	-0.124974
C	-2.832374	3.132886	-0.543244
H	-5.616450	3.127868	-1.061361
H	-6.759565	0.728615	-0.509103
H	-4.925417	-4.006000	0.725972
H	-2.438727	-5.078282	0.921559
H	2.391722	-3.107860	1.227461
H	3.366361	-0.612248	1.583751
H	1.486597	4.190412	0.645750
H	-0.845413	5.139422	-0.359215
Fe	-1.624147	0.010079	0.151328
C	0.144320	-4.504338	0.878145
C	0.452662	-5.058583	2.135539
C	0.325650	-5.254741	-0.300463
C	0.966119	-6.357111	2.194372
C	0.842064	-6.549697	-0.193663
C	1.176598	-7.114965	1.039502
H	1.200251	-6.787567	3.165763
H	0.985431	-7.130770	-1.102006
C	-3.308492	4.448530	-1.072368
C	-3.857094	5.407107	-0.200984
C	-3.195098	4.716303	-2.449532
C	-4.288804	6.628379	-0.726709
C	-3.638862	5.949320	-2.934951
C	-4.191525	6.916729	-2.090721
H	-4.709972	7.372016	-0.053519
C	-6.245382	-1.763762	0.228574
C	-7.033182	-1.726765	1.393229
C	-6.760207	-2.303957	-0.965134
C	-8.333462	-2.237404	1.346334
C	-8.065557	-2.803277	-0.970287
C	-8.866435	-2.782679	0.175266
H	-8.943980	-2.207173	2.246271
H	-8.466424	-3.217201	-1.893051
O	-1.325511	-0.220245	-1.419129
C	-5.920044	-2.339845	-2.219031
H	-5.581654	-1.336535	-2.501850
H	-5.017702	-2.945725	-2.077049
H	-6.484527	-2.759831	-3.056600
C	-6.484714	-1.140279	2.671950
H	-5.569157	-1.655242	2.985431
H	-6.223653	-0.082914	2.545167
H	-7.214401	-1.216123	3.483445
C	-10.261680	-3.358662	0.153715
H	-10.249910	-4.433686	0.377476
H	-10.904453	-2.878274	0.898760
H	-10.729472	-3.237308	-0.829057
C	-3.974211	5.124724	1.277705
H	-4.607139	4.249802	1.466872
H	-2.995344	4.907347	1.720695
H	-4.406597	5.978901	1.807006
C	-4.694324	8.228652	-2.643043
H	-4.103206	8.552135	-3.506403
H	-5.737148	8.144403	-2.976683
H	-4.657087	9.021153	-1.888345
C	-0.022450	-4.681275	-1.651750
H	-1.059091	-4.326762	-1.679906
H	0.623784	-3.832881	-1.904716
H	0.097514	-5.439024	-2.432235
C	0.219971	-4.276862	3.407290
H	0.891054	-3.412806	3.478800
H	-0.802962	-3.885959	3.453203
H	0.385145	-4.906264	4.286822
C	1.766347	-8.502274	1.119719
H	1.552813	-8.973398	2.085014
H	1.373074	-9.150539	0.329327
H	2.858062	-8.475472	1.003676
H	-3.549360	6.158824	-3.998737
C	-2.598538	3.690994	-3.383559
H	-1.561765	3.459639	-3.112873
H	-3.147719	2.743603	-3.340700
H	-2.610232	4.048340	-4.417374
C	2.759153	1.961803	1.337873
C	3.769163	1.806678	0.389944
C	3.071705	2.619640	2.533096
C	5.082614	2.221411	0.616636
C	4.366272	3.076748	2.800613
H	2.278699	2.741969	3.260673
C	4.386827	0.321694	-1.316331
C	6.091647	1.983405	-0.508478
C	5.357269	2.850352	1.830327
C	3.960353	-0.850115	-1.947060
C	5.742767	0.635789	-1.153170
H	6.370116	3.179801	2.029830
C	4.947211	-1.729000	-2.414791
C	6.681621	-0.272601	-1.635064
C	6.308540	-1.468152	-2.272217
H	4.586128	-2.639360	-2.874426
H	7.735006	-0.048974	-1.514300
C	4.737421	3.794245	4.105854
C	5.282662	5.201161	3.776508
C	3.530583	3.954233	5.045114
C	5.822518	2.982328	4.846223
H	6.172744	5.152730	3.140917
H	4.527948	5.797317	3.251622
H	5.556815	5.729386	4.697380
H	3.116574	2.984703	5.343052
H	3.840548	4.476707	5.956529
H	2.730564	4.541573	4.581001
H	6.102273	3.483014	5.780714
H	5.457174	1.978961	5.091197
H	6.728934	2.870718	4.242462
O	3.437495	1.190627	-0.800885
C	7.394600	-2.429088	-2.773819
C	8.265079	-2.882592	-1.581564
C	6.797470	-3.680091	-3.438781
C	8.280775	-1.704725	-3.810478
H	8.752924	-2.034976	-1.088732
H	7.656541	-3.401348	-0.832679
H	9.049869	-3.569130	-1.921096
H	6.184402	-3.422286	-4.308950
H	7.605652	-4.335634	-3.781517
H	6.175744	-4.251761	-2.741588
H	9.065396	-2.376781	-4.178156
H	7.683891	-1.373864	-4.667508
H	8.769777	-0.823419	-3.382025
C	2.526198	-1.299264	-2.130338
O	2.296321	-2.481365	-2.394605
C	7.538103	2.027859	-0.000685
H	7.725250	1.257534	0.754534
H	8.241298	1.883639	-0.825857
H	7.762609	3.005233	0.436482
C	5.900829	3.095775	-1.574739
H	6.108945	4.078877	-1.137667
H	6.583148	2.931855	-2.415958
H	4.877209	3.099455	-1.959285
N	1.556054	-0.358942	-2.050264
H	0.582348	-0.651117	-2.012868
H	1.766100	0.546129	-1.657080

2-CONH$_2$-5qui

B3LYP SCF energy: -3371.69362605 a.u.
B3LYP enthalpy: -3370.361790 a.u.
B3LYP free energy: -3370.559515 a.u.
B3LYP SCF energy in solution: -3369.97496109 a.u.
B3LYP enthalpy in solution: -3368.643125 a.u.
B3LYP free energy in solution: -3368.840850 a.u.

Cartesian coordinates

ATOM	X	Y	Z
N	-3.541117	0.722617	-0.017105
N	-2.405037	-1.848860	0.586589
N	0.182045	-0.635972	0.886850
N	-0.962186	1.943280	0.334342
C	-3.892434	2.016296	-0.331878
C	-5.303062	2.080901	-0.638038
C	-5.795409	0.815714	-0.509233
C	-4.687658	-0.030655	-0.128627
C	-4.787680	-1.409186	0.094789
C	-3.717129	-2.240743	0.440201
C	-3.831473	-3.662969	0.671656
C	-2.576116	-4.124484	0.934378
C	-1.686114	-2.987465	0.865030
C	-0.298478	-3.058470	1.049840

C 0.558876 -1.952588 1.050883
C 1.980580 -2.025606 1.297519
C 2.450410 -0.747952 1.301906
C 1.326254 0.118477 1.021843
C 1.407365 1.511150 0.881218
C 0.334860 2.349858 0.551925
C 0.441795 3.777860 0.349180
C -0.797719 4.223005 -0.001589
C -1.670392 3.071535 -0.015857
C -3.030577 3.119017 -0.341484
H -5.832780 2.982963 -0.908688
H -6.809723 0.472313 -0.653587
H -4.757051 -4.219205 0.634115
H -2.264171 -5.134896 1.155341
H 2.531119 -2.942489 1.447547
H 3.465142 -0.417052 1.465337
H 1.350598 4.351180 0.458859
H -1.107307 5.232044 -0.233077
Fe -1.592956 -0.025584 -0.020995
C 0.298341 -4.408952 1.297888
C 0.599436 -4.801861 2.616187
C 0.536207 -5.280711 0.216419
C 1.156070 -6.064992 2.834392
C 1.095991 -6.534086 0.482713
C 1.419615 -6.942075 1.779243
H 1.382987 -6.371522 3.853319
H 1.283494 -7.208543 -0.349860
C -3.604488 4.444670 -0.735108
C -4.166900 5.285802 0.241780
C -3.570309 4.839116 -2.085809
C -4.692217 6.519853 -0.152181
C -4.106610 6.080684 -2.437695
C -4.674666 6.933588 -1.486885
H -5.124259 7.173126 0.602859
C -6.133448 -2.043517 -0.072533
C -7.031840 -2.078401 1.009067
C -6.489650 -2.595553 -1.317426
C -8.284081 -2.672135 0.826742
C -7.752037 -3.178261 -1.457987
C -8.661572 -3.229893 -0.397700
H -8.980659 -2.698413 1.661954
H -8.031295 -3.600995 -2.420690
O -1.274991 -0.269085 -1.618167
C -5.527369 -2.556459 -2.480244
H -5.212967 -1.531129 -2.705873
H -4.613805 -3.121313 -2.261013
H -5.982611 -2.981202 -3.379591
C -6.651874 -1.482138 2.343217
H -5.734605 -1.936480 2.735401
H -6.459674 -0.406006 2.258677
H -7.447588 -1.628978 3.079376
C -10.006465 -3.894925 -0.564655
H -9.943241 -4.972429 -0.362562
H -10.747719 -3.477349 0.124640
H -10.386359 -3.778479 -1.585218
C -4.199340 4.865069 1.691497
H -4.772796 3.940178 1.824526
H -3.190316 4.667892 2.071913
H -4.653983 5.640448 2.315002
C -5.277170 8.255803 -1.896364
H -4.744836 8.691215 -2.748700
H -6.327054 8.136968 -2.195493
H -5.252758 8.978442 -1.074078
C 0.204361 -4.880673 -1.200005
H -0.853683 -4.613394 -1.302066
H 0.795569 -4.017329 -1.525847
H 0.411111 -5.705550 -1.888753
C 0.316580 -3.885610 3.783673
H 0.964494 -3.001195 3.767604
H -0.716448 -3.520069 3.763699
H 0.477326 -4.404406 4.733396
C 2.055099 -8.288130 2.030390
H 1.832477 -8.654605 3.038073
H 1.705808 -9.035993 1.310482
H 3.147910 -8.233475 1.936653
H -4.078698 6.388617 -3.480725
C -2.960051 3.939813 -3.133866
H -1.899773 3.750607 -2.929553
H -3.451630 2.960547 -3.156076
H -3.041345 4.386567 -4.129005
C 2.752466 2.135038 1.053871
C 3.756834 1.907147 0.114310
C 3.062697 2.915696 2.174763
C 5.062877 2.371066 0.285840
C 4.348478 3.423295 2.382145
H 2.274156 3.091992 2.895941
C 4.395358 0.278930 -1.448664
C 6.068208 2.046065 -0.820472
C 5.335341 3.121962 1.428233
C 3.990605 -0.954916 -1.964775
C 5.745104 0.634602 -1.325975
H 6.342677 3.489918 1.582978
C 4.993158 -1.855638 -2.349613
C 6.700662 -0.297245 -1.723022
C 6.349582 -1.556400 -2.238278
H 4.648439 -2.812104 -2.719519
H 7.749645 -0.041483 -1.631409
C 4.714727 4.273115 3.606613
C 5.231837 5.649865 3.135091
C 3.511147 4.504165 4.535229
C 5.819365 3.557582 4.414390
H 6.118721 5.554784 2.500290
H 4.462924 6.177699 2.560040
H 5.501963 6.271327 3.997035
H 3.118330 3.562063 4.933074
H 3.816836 5.121756 5.386607
H 2.696581 5.026339 4.021149
H 6.096413 4.153215 5.292257
H 5.473840 2.577277 4.760554
H 6.723406 3.402376 3.816692
O 3.430019 1.172950 -1.008733
C 7.453036 -2.540253 -2.649427
C 8.338474 -2.855366 -1.424068
C 6.878806 -3.863041 -3.182577
C 8.318694 -1.904607 -3.759013
H 8.810466 -1.953281 -1.020668
H 7.744633 -3.310029 -0.623493
H 9.136112 -3.555719 -1.699333
H 6.257446 -3.705976 -4.070679
H 7.698757 -4.533613 -3.462576
H 6.271282 -4.373988 -2.428062
H 9.115493 -2.593648 -4.063667
H 7.710666 -1.672851 -4.640378
H 8.790948 -0.975203 -3.423569
C 2.564235 -1.441704 -2.110388
O 2.352347 -2.647769 -2.255415
C 7.517385 2.172156 -0.334526
H 7.728268 1.485012 0.491407
H 8.216509 1.961586 -1.148805
H 7.723778 3.192802 0.000812
C 5.843836 3.043568 -1.988983
H 6.032799 4.069288 -1.652826
H 6.523373 2.812387 -2.816520
H 4.817585 2.986924 -2.362088
N 1.585149 -0.509896 -2.135030
H 0.608825 -0.796252 -2.096059
H 1.783560 0.436198 -1.847353

2-COOH-3tri

B3LYP SCF energy: -3391.57383168 a.u.
B3LYP enthalpy: -3390.253422 a.u.
B3LYP free energy: -3390.448953 a.u.
B3LYP SCF energy in solution: -3389.83487069 a.u.
B3LYP enthalpy in solution: -3388.514461 a.u.
B3LYP free energy in solution: -3388.709992 a.u.

Cartesian coordinates

ATOM X Y Z
N -3.353663 0.902490 0.029945
N -2.465829 -1.690688 0.597104
N 0.122028 -0.735630 0.944936
N -0.781003 1.866920 0.469080
C -3.578359 2.193233 -0.407449
C -4.969749 2.377387 -0.728290
C -5.596974 1.198340 -0.459936
C -4.584092 0.276653 -0.014183
C -4.835136 -1.059305 0.276772
C -3.819630 -1.967976 0.550082
C -4.044388 -3.379507 0.727012
C -2.819527 -3.967019 0.821663
C -1.841685 -2.913748 0.737245
C -0.469338 -3.130540 0.821129
C 0.436997 -2.084298 0.934423
C 1.840906 -2.267390 1.191709
C 2.369243 -1.032990 1.411237
C 1.304103 -0.079030 1.228869
C 1.487791 1.300078 1.259898
C 0.502172 2.193930 0.865027
C 0.732265 3.605792 0.699216
C -0.397844 4.130159 0.151680
C -1.338533 3.048161 0.016140
C -2.633713 3.212255 -0.458807
H -5.395059 3.302964 -1.088493
H -6.643691 0.950730 -0.561639
H -5.020261 -3.842292 0.752526
H -2.577985 -5.012157 0.949430
H 2.335066 -3.227335 1.214783
H 3.390352 -0.775284 1.649460
H 1.655234 4.106546 0.952627
H -0.602377 5.153333 -0.128575
Fe -1.561468 0.045400 0.223170
C 0.030209 -4.539473 0.871865
C 0.355017 -5.125674 2.110430
C 0.147652 -5.277303 -0.322668
C 0.822498 -6.442797 2.132650
C 0.622093 -6.590468 -0.253308
C 0.972442 -7.188017 0.960328
H 1.069284 -6.897852 3.089587
H 0.717863 -7.160856 -1.174691
C -3.038013 4.545864 -1.001438
C -3.617293 5.511204 -0.157914
C -2.822808 4.823352 -2.364576
C -3.979128 6.748881 -0.697873
C -3.199885 6.072496 -2.864865
C -3.782451 7.046742 -2.049069
H -4.423379 7.498150 -0.046100
C -6.246462 -1.552785 0.220408
C -7.059726 -1.466745 1.364766
C -6.753464 -2.091249 -0.977518
C -8.377306 -1.927884 1.293942
C -8.076816 -2.539700 -1.006995
C -8.903134 -2.470530 0.118441
H -9.007329 -1.860149 2.178257
H -8.471735 -2.951687 -1.933192
O -1.251654 -0.175076 -1.353553
C -5.886179 -2.178775 -2.210291
H -5.492044 -1.195797 -2.492066
H -5.018321 -2.826889 -2.042038
H -6.450409 -2.577867 -3.058201
C -6.519465 -0.880977 2.647337
H -5.625981 -1.418685 2.985269
H -6.224558 0.166569 2.513335
H -7.267103 -0.924940 3.444725
C -10.318799 -2.992647 0.071427
H -10.353874 -4.064234 0.308850
H -10.959387 -2.477903 0.795090
H -10.758868 -2.866617 -0.923500
C -3.838434 5.219681 1.307086
H -4.520533 4.372802 1.446535
H -2.899423 4.953628 1.806154
H -4.263982 6.087416 1.819625
C -4.211039 8.377173 -2.619435
H -3.552794 8.693203 -3.435712
H -5.229875 8.324408 -3.025779
H -4.205785 9.160811 -1.854642
C -0.242286 -4.674104 -1.649881
H -1.317652 -4.459874 -1.683274
H 0.280441 -3.731668 -1.838040
H -0.012041 -5.363205 -2.468263
C 0.187451 -4.359210 3.401584
H 0.906076 -3.534825 3.478442
H -0.811387 -3.914080 3.473462
H 0.332818 -5.015200 4.265044
C 1.515367 -8.596006 1.001052
H 1.308685 -9.077442 1.962718
H 1.080851 -9.215504 0.209208
H 2.604312 -8.604760 0.858976
H -3.032581 6.289589 -3.917681
C -2.187974 3.792220 -3.266549
H -1.169211 3.550483 -2.941660
H -2.747784 2.850250 -3.254013
H -2.140804 4.150613 -4.299011
C 2.868264 1.806647 1.525898
C 3.804346 1.715690 0.497063
C 3.295376 2.284751 2.768220
C 5.156699 2.004429 0.680292
C 4.634946 2.624965 2.995403
H 2.562760 2.356104 3.562980
C 4.173426 0.406290 -1.387949
C 6.069650 1.819425 -0.535212
C 5.548610 2.457800 1.939805
C 3.610867 -0.667237 -2.085268
C 5.561848 0.586083 -1.296971
H 6.593510 2.690301 2.110428
C 4.476743 -1.577695 -2.706935
C 6.379573 -0.340607 -1.937970
C 5.861992 -1.434137 -2.655334
H 4.013641 -2.415237 -3.211916

H	7.454711	-0.216659	-1.878820
C	5.135968	3.147637	4.349388
C	5.761397	4.546513	4.156618
C	4.003464	3.266735	5.382663
C	6.201514	2.180966	4.910644
H	6.605542	4.520694	3.459997
H	5.021798	5.251304	3.760736
H	6.128282	4.936196	5.113660
H	3.540101	2.296102	5.590931
H	4.405261	3.651570	6.326235
H	3.220650	3.956596	5.048518
H	6.573048	2.541613	5.877233
H	5.779045	1.180825	5.057570
H	7.059275	2.086555	4.236955
O	3.343058	1.271738	-0.718669
C	6.825333	-2.418491	-3.331870
C	7.742093	-3.053437	-2.263533
C	6.080157	-3.548863	-4.060479
C	7.688154	-1.661654	-4.365029
H	8.333103	-2.298535	-1.734415
H	7.150560	-3.598434	-1.519676
H	8.440624	-3.758468	-2.729899
H	5.426493	-3.160365	-4.848895
H	6.804059	-4.224143	-4.529741
H	5.468963	-4.141308	-3.371152
H	8.386249	-2.349363	-4.857213
H	7.058244	-1.203320	-5.135349
H	8.277174	-0.866021	-3.896767
C	2.143495	-0.968621	-2.127514
O	1.730204	-2.102912	-2.308259
O	1.364797	0.098654	-1.968339
H	0.411765	-0.171214	-1.858417
C	7.542545	1.686721	-0.130150
H	7.706380	0.819962	0.518562
H	8.179419	1.584891	-1.013728
H	7.877958	2.584023	0.398413
C	5.909514	3.058519	-1.456317
H	6.228628	3.965136	-0.929907
H	6.521232	2.940097	-2.357517
H	4.867635	3.186133	-1.762680

2-COOH-5qui

B3LYP SCF energy: -3391.55753711 a.u.
B3LYP enthalpy: -3390.239616 a.u.
B3LYP free energy: -3390.437326 a.u.
B3LYP SCF energy in solution: -3389.82554956 a.u.
B3LYP enthalpy in solution: -3388.507628 a.u.
B3LYP free energy in solution: -3388.705338 a.u.

Cartesian coordinates

ATOM	X	Y	Z
N	-3.458048	0.784896	0.022138
N	-2.368342	-1.805175	0.639512
N	0.234475	-0.628168	0.964382
N	-0.869493	1.970179	0.434331
C	-3.784182	2.083239	-0.297968
C	-5.182361	2.165397	-0.652961
C	-5.693393	0.905433	-0.544652
C	-4.608729	0.046234	-0.128185
C	-4.732433	-1.330852	0.099551
C	-3.683664	-2.176828	0.478998
C	-3.827254	-3.592926	0.731135
C	-2.583955	-4.070975	1.021615
C	-1.674027	-2.950164	0.944694
C	-0.287426	-3.040949	1.138965
C	0.590894	-1.951301	1.126132
C	2.012889	-2.048028	1.357498
C	2.504011	-0.777681	1.357653
C	1.389567	0.107346	1.098775
C	1.485876	1.504305	1.000258
C	0.424152	2.361516	0.692316
C	0.545217	3.794250	0.543894
C	-0.682570	4.259542	0.178992
C	-1.560342	3.113305	0.103742
C	-2.910373	3.177390	-0.263679
H	-5.692086	3.074082	-0.939265
H	-6.706056	0.574266	-0.724702
H	-4.761459	-4.134082	0.687651
H	-2.293329	-5.082607	1.265164
H	2.549898	-2.973861	1.501916
H	3.525505	-0.462087	1.508796
H	1.456723	4.354357	0.693684
H	-0.981091	5.277537	-0.026147
Fe	-1.515996	0.007636	0.021651
C	0.285254	-4.397249	1.409611
C	0.598276	-4.766539	2.732149
C	0.490841	-5.297559	0.345197
C	1.133265	-6.035446	2.970199
C	1.032725	-6.554433	0.630603
C	1.366130	-6.940380	1.931478
H	1.368943	-6.323750	3.992427
H	1.197118	-7.249941	-0.189548
C	-3.457955	4.513320	-0.658285
C	-4.080269	5.330964	0.301942
C	-3.338073	4.939671	-1.994446
C	-4.579318	6.575119	-0.094254
C	-3.852007	6.189908	-2.348803
C	-4.478412	7.020519	-1.415004
H	-5.056756	7.211194	0.647999
C	-6.081539	-1.947247	-0.100772
C	-7.018133	-1.942730	0.948434
C	-6.401624	-2.521531	-1.345492
C	-8.272707	-2.520588	0.734114
C	-7.668147	-3.086140	-1.518554
C	-8.615643	-3.099273	-0.490788
H	-8.998834	-2.517277	1.544169
H	-7.920256	-3.525215	-2.481424
O	-1.194042	-0.242939	-1.593250
C	-5.397476	-2.524552	-2.473072
H	-5.044077	-1.512052	-2.698554
H	-4.509590	-3.113404	-2.214816
H	-5.832659	-2.946767	-3.383451
C	-6.676125	-1.322194	2.281841
H	-5.772275	-1.771052	2.709773
H	-6.478672	-0.248209	2.182791
H	-7.493881	-1.453013	2.996523
C	-9.964887	-3.745785	-0.691787
H	-9.926222	-4.820331	-0.468855
H	-10.722032	-3.304088	-0.035628
H	-10.308034	-3.641798	-1.726614
C	-4.202518	4.875607	1.736380
H	-4.801708	3.960686	1.813940
H	-3.220637	4.647243	2.166990
H	-4.675499	5.645249	2.353285
C	-5.053848	8.353172	-1.829514
H	-4.466006	8.810376	-2.632537
H	-6.081053	8.242514	-2.201613
H	-5.085680	9.054129	-0.988936
C	0.129893	-4.927030	-1.072473
H	-0.955371	-4.814879	-1.186270
H	0.584568	-3.980134	-1.377599
H	0.459774	-5.704752	-1.768097
C	0.351279	-3.819962	3.883508
H	1.027698	-2.957810	3.847687
H	-0.669340	-3.421466	3.861598
H	0.500277	-4.326170	4.841875
C	1.980735	-8.291919	2.204026
H	1.759859	-8.634744	3.220345
H	1.613081	-9.047602	1.501686
H	3.073575	-8.257804	2.101267
H	-3.759218	6.522650	-3.380375
C	-2.661856	4.065377	-3.023068
H	-1.608997	3.892738	-2.771034
H	-3.133076	3.077671	-3.081397
H	-2.703575	4.524228	-4.015110
C	2.851420	2.089834	1.155623
C	3.779583	1.881400	0.136669
C	3.273025	2.757371	2.310435
C	5.125133	2.228900	0.263178
C	4.602872	3.164203	2.470174
H	2.545081	2.920231	3.095900
C	4.165708	0.347178	-1.568361
C	6.036141	1.897479	-0.922209
C	5.513239	2.868673	1.440008
C	3.620565	-0.812278	-2.126605
C	5.550037	0.563366	-1.507510
H	6.552461	3.150795	1.563708
C	4.500719	-1.781844	-2.624745
C	6.383449	-0.424944	-2.025329
C	5.884056	-1.611493	-2.591356
H	4.051161	-2.683720	-3.019328
H	7.456275	-0.275694	-1.987011
C	5.098035	3.892567	3.727719
C	5.683587	5.263769	3.325081
C	3.969171	4.132523	4.743776
C	6.193356	3.046909	4.413272
H	6.523270	5.158009	2.630605
H	4.922286	5.883358	2.838246
H	6.046314	5.799111	4.210619
H	3.534925	3.191300	5.098393
H	4.365696	4.663806	5.615744
H	3.164914	4.743652	4.319349
H	6.560871	3.554911	5.312838
H	5.799590	2.068273	4.709146
H	7.049124	2.877586	3.751812
O	3.318150	1.269856	-1.002067
C	6.863647	-2.661417	-3.132636
C	7.788744	-3.134710	-1.990232
C	6.137133	-3.890018	-3.704097
C	7.715970	-2.035575	-4.258043
H	8.367296	-2.307192	-1.566418
H	7.204625	-3.585019	-1.180092
H	8.498619	-3.884655	-2.359319
H	5.479085	-3.619857	-4.537000
H	6.872015	-4.610711	-4.079404
H	5.533642	-4.394540	-2.941863
H	8.425270	-2.771851	-4.654749
H	7.079936	-1.693928	-5.082034
H	8.292073	-1.175557	-3.900657
C	2.153142	-1.124127	-2.144759
O	1.748786	-2.276592	-2.148031
O	1.377453	-0.048500	-2.178070
H	0.409011	-0.287461	-2.048531
C	7.513012	1.848746	-0.512355
H	7.696075	1.082156	0.247762
H	8.147660	1.638209	-1.378085
H	7.833931	2.815976	-0.114225
C	5.848217	2.997094	-2.001612
H	6.152424	3.972525	-1.605409
H	6.457878	2.768365	-2.882699
H	4.802756	3.062876	-2.315216

2-COOMe-3tri

B3LYP SCF energy: -3430.87647657 a.u.
B3LYP enthalpy: -3429.525621 a.u.
B3LYP free energy: -3429.725522 a.u.
B3LYP SCF energy in solution: -3429.10133274 a.u.
B3LYP enthalpy in solution: -3427.750477 a.u.
B3LYP free energy in solution: -3427.950378 a.u.

Cartesian coordinates

ATOM	X	Y	Z
N	-3.603450	0.661864	-0.023683
N	-2.472391	-1.868243	0.419644
N	0.068168	-0.705607	0.549432
N	-1.060240	1.814004	0.079864
C	-3.970179	1.938108	-0.399108
C	-5.403549	2.034737	-0.508297
C	-5.907766	0.809837	-0.192549
C	-4.782513	-0.049692	0.076158
C	-4.900757	-1.413451	0.319712
C	-3.798970	-2.252297	0.445896
C	-3.901690	-3.683817	0.574094
C	-2.630932	-4.172476	0.587599
C	-1.745425	-3.039373	0.499604
C	-0.358926	-3.142450	0.570959
C	0.467544	-2.027289	0.641878
C	1.875477	-2.103354	0.939071
C	2.321228	-0.825505	1.069531
C	1.206661	0.043430	0.782767
C	1.301985	1.430452	0.701407
C	0.235148	2.233458	0.305118
C	0.358773	3.642067	0.019720
C	-0.860708	4.069513	-0.407223
C	-1.745497	2.934678	-0.349377
C	-3.108265	3.009398	-0.610357
H	-5.935124	2.935938	-0.777842
H	-6.939798	0.492213	-0.156178
H	-4.834790	-4.225189	0.632892
H	-2.301309	-5.198010	0.667902
H	2.427194	-3.025203	1.047910
H	3.317289	-0.491815	1.316662
H	1.272117	4.209284	0.119228
H	-1.158373	5.060430	-0.718386
Fe	-1.742834	-0.062178	-0.021333
C	0.250364	-4.505108	0.694343
C	0.452696	-5.056131	1.975945
C	0.597324	-5.233996	-0.457565
C	1.012462	-6.330888	2.084165
C	1.151133	-6.509725	-0.303407
C	1.373055	-7.072233	0.954671
H	1.165155	-6.757096	3.073523
H	1.415831	-7.075509	-1.194064
C	-3.679087	4.305400	-1.092590
C	-4.108073	5.278380	-0.171483
C	-3.777447	4.540153	-2.476849
C	-4.633719	6.480352	-0.654460
C	-4.307850	5.755175	-2.918805

C	-4.744612	6.736159	-2.023889
H	-4.963334	7.234574	0.056974
C	-6.270075	-2.013893	0.382985
C	-6.936912	-2.099637	1.618412
C	-6.884315	-2.483120	-0.793511
C	-8.217453	-2.658609	1.658517
C	-8.165465	-3.035489	-0.710800
C	-8.847042	-3.135199	0.505597
H	-8.734904	-2.721975	2.613411
H	-8.642639	-3.395507	-1.619811
O	-1.621485	-0.263155	-1.614866
C	-6.174141	-2.387262	-2.122360
H	-5.936181	-1.347082	-2.373017
H	-5.222208	-2.930159	-2.105417
H	-6.789997	-2.800903	-2.926305
C	-6.281022	-1.592045	2.880113
H	-5.336766	-2.113967	3.074741
H	-6.040705	-0.525194	2.802443
H	-6.933975	-1.733019	3.746472
C	-10.216899	-3.765853	0.574979
H	-10.146166	-4.853291	0.710684
H	-10.797423	-3.370316	1.414980
H	-10.785224	-3.590349	-0.344618
C	-3.999348	5.031302	1.314120
H	-4.554515	4.133482	1.609683
H	-2.957602	4.871447	1.615882
H	-4.393149	5.879009	1.882584
C	-5.345133	8.027523	-2.524776
H	-4.896076	8.335417	-3.475055
H	-6.425015	7.922887	-2.694676
H	-5.206448	8.839674	-1.803494
C	0.406256	-4.657390	-1.838786
H	-0.534077	-4.102979	-1.920169
H	1.221760	-3.968024	-2.085817
H	0.402096	-5.451857	-2.591915
C	0.063727	-4.287936	3.216729
H	0.617490	-3.344967	3.293768
H	-1.001402	-4.028743	3.207628
H	0.264206	-4.874773	4.118020
C	2.005279	-8.435921	1.094663
H	1.630006	-8.962978	1.978550
H	1.808512	-9.059824	0.216588
H	3.095478	-8.357826	1.201353
H	-4.380356	5.939543	-3.988561
C	-3.313573	3.497708	-3.465613
H	-2.255725	3.251188	-3.319546
H	-3.868609	2.560109	-3.345950
H	-3.446540	3.846053	-4.494093
C	2.587321	2.096591	1.060511
C	3.755679	1.920447	0.316202
C	2.648492	2.916891	2.196465
C	4.971239	2.498113	0.697595
C	3.836308	3.522342	2.611705
H	1.733935	3.051028	2.760693
C	4.782230	0.376600	-1.110138
C	6.171295	2.268922	-0.220832
C	4.989705	3.285727	1.846970
C	4.606776	-0.877372	-1.709178
C	6.060034	0.840470	-0.760716
H	5.925020	3.737371	2.155575
C	5.734172	-1.686024	-1.913549
C	7.149190	0.003417	-0.988361
C	7.015773	-1.274034	-1.557576
H	5.554130	-2.658162	-2.353142
H	8.138212	0.352551	-0.716390
C	3.920418	4.417118	3.855565
C	4.402390	5.824136	3.439506
C	2.561046	4.563533	4.559125
C	4.922430	3.806142	4.859083
H	5.389347	5.791628	2.966699
H	3.704497	6.278876	2.727684
H	4.472391	6.478663	4.316439
H	2.178542	3.598016	4.907828
H	2.667582	5.214085	5.433937
H	1.809786	5.012572	3.900043
H	4.998941	4.435657	5.753631
H	4.599510	2.806506	5.170008
H	5.924429	3.715292	4.427247
O	3.678166	1.142628	-0.819219
C	8.260026	-2.146673	-1.766399
C	8.939082	-2.400518	-0.402842
C	7.913218	-3.506791	-2.393655
C	9.246724	-1.417028	-2.703799
H	9.245712	-1.465951	0.078505
H	8.257747	-2.921175	0.279002
H	9.834697	-3.019824	-0.531924
H	7.450733	-3.391982	-3.379950
H	8.827199	-4.097144	-2.520947
H	7.229361	-4.082283	-1.760467
H	10.144743	-2.026149	-2.861480
H	8.786443	-1.226259	-3.679500
H	9.564669	-0.454664	-2.289090
C	3.295074	-1.444469	-2.141676
O	3.114094	-2.641237	-2.296761
O	2.373556	-0.510622	-2.391173
C	7.504346	2.520756	0.494714
H	7.635876	1.853279	1.352680
H	8.344183	2.376177	-0.190980
H	7.562612	3.554578	0.846956
C	6.055958	3.247809	-1.420978
H	6.094190	4.284275	-1.067449
H	6.880781	3.083275	-2.123179
H	5.114048	3.101572	-1.956958
C	1.084570	-0.988906	-2.816754
H	0.654465	-1.645674	-2.062825
H	0.462715	-0.103345	-2.917777
H	1.177672	-1.525192	-3.765431

2-COOMe-5qui

B3LYP SCF energy: -3430.85819294 a.u.
B3LYP enthalpy: -3429.509116 a.u.
B3LYP free energy: -3429.710495 a.u.
B3LYP SCF energy in solution: -3429.08830638 a.u.
B3LYP enthalpy in solution: -3427.739229 a.u.
B3LYP free energy in solution: -3427.940608 a.u.

Cartesian coordinates

ATOM	X	Y	Z
N	-3.680201	0.644765	0.013884
N	-2.461108	-1.928230	0.427308
N	0.129536	-0.682773	0.587305
N	-1.083270	1.881454	0.143459
C	-4.067355	1.939296	-0.242646
C	-5.501802	1.996632	-0.413385
C	-5.968323	0.722227	-0.281022
C	-4.821557	-0.122258	-0.031401
C	-4.879909	-1.512952	0.111303
C	-3.771771	-2.340773	0.323365
C	-3.846831	-3.774775	0.483069
C	-2.573159	-4.220693	0.678843
C	-1.709915	-3.062766	0.636569
C	-0.321711	-3.107195	0.823207
C	0.516226	-1.987736	0.817296
C	1.928931	-2.032514	1.119870
C	2.380775	-0.748762	1.095020
C	1.258725	0.091112	0.737121
C	1.320625	1.483251	0.577882
C	0.224717	2.297380	0.253957
C	0.306132	3.718436	-0.008400
C	-0.958600	4.148926	-0.276183
C	-1.825577	2.998525	-0.175606
C	-3.212742	3.042615	-0.350380
H	-6.061964	2.899586	-0.609789
H	-6.987864	0.370345	-0.347598
H	-4.761756	-4.348992	0.455110
H	-2.233924	-5.233259	0.842305
H	2.484693	-2.934715	1.328691
H	3.381740	-0.398528	1.295960
H	1.216762	4.298356	0.002922
H	-1.288787	5.148787	-0.518741
Fe	-1.729841	-0.073492	-0.174171
C	0.292232	-4.440797	1.126724
C	0.453648	-4.825873	2.472964
C	0.682687	-5.303816	0.086542
C	1.017698	-6.071095	2.757998
C	1.239325	-6.544156	0.417674
C	1.422093	-6.943248	1.742815
H	1.137854	-6.369220	3.797432
H	1.537936	-7.213562	-0.386133
C	-3.827672	4.364067	-0.693800
C	-4.220654	5.244759	0.329902
C	-4.002423	4.715252	-2.045654
C	-4.788793	6.473685	-0.018042
C	-4.573236	5.953821	-2.350919
C	-4.976109	6.845045	-1.352159
H	-5.091143	7.156713	0.772941
C	-6.223721	-2.165138	0.006156
C	-7.016968	-2.326853	1.156312
C	-6.684639	-2.606233	-1.248356
C	-8.269814	-2.934091	1.032205
C	-7.943445	-3.207928	-1.329465
C	-8.749249	-3.384104	-0.200958
H	-8.885447	-3.057197	1.920696
H	-8.303037	-3.545885	-2.299018
O	-1.647512	-0.212710	-1.803616
C	-5.836013	-2.428813	-2.484402
H	-5.594094	-1.373523	-2.655016
H	-4.879530	-2.955819	-2.390155
H	-6.350684	-2.810363	-3.371070
C	-6.524584	-1.849583	2.501451
H	-5.581000	-2.336176	2.774677
H	-6.332380	-0.770271	2.495259
H	-7.257561	-2.061257	3.285457
C	-10.091384	-4.066935	-0.307844
H	-9.992344	-5.155000	-0.197240
H	-10.778984	-3.722123	0.471527
H	-10.558399	-3.881149	-1.280854
C	-4.027799	4.871432	1.780126
H	-4.541578	3.933613	2.021236
H	-2.967601	4.719204	2.014305
H	-4.414306	5.652669	2.441244
C	-5.621082	8.163195	-1.706495
H	-5.225865	8.561981	-2.646850
H	-6.706472	8.052481	-1.831388
H	-5.458239	8.911574	-0.923911
C	0.534235	-4.908673	-1.362151
H	-0.418468	-4.402746	-1.548664
H	1.337945	-4.227899	-1.665228
H	0.584188	-5.790100	-2.009282
C	0.018313	-3.913055	3.594709
H	0.589969	-2.977507	3.594147
H	-1.038120	-3.637317	3.497691
H	0.157610	-4.395470	4.566673
C	2.057951	-8.272020	2.072712
H	1.655052	-8.689506	3.001804
H	1.896589	-9.002050	1.272728
H	3.143108	-8.169250	2.206319
H	-4.704734	6.228920	-3.395249
C	-3.577461	3.772153	-3.145435
H	-2.514229	3.518478	-3.066751
H	-4.126880	2.825077	-3.092514
H	-3.753034	4.214612	-4.130363
C	2.619386	2.168834	0.840051
C	3.765648	1.934998	0.076065
C	2.714701	3.074309	1.908317
C	4.984174	2.560832	0.362889
C	3.908738	3.719018	2.233614
H	1.819905	3.244690	2.493448
C	4.777001	0.287418	-1.242442
C	6.151336	2.279987	-0.582191
C	5.034899	3.437691	1.444239
C	4.612736	-1.020430	-1.716671
C	6.055524	0.807128	-0.985643
H	5.974302	3.924177	1.678114
C	5.752698	-1.819781	-1.887464
C	7.156757	-0.023469	-1.174802
C	7.035485	-1.350465	-1.618601
H	5.581083	-2.831648	-2.229824
H	8.145391	0.370971	-0.972551
C	4.026009	4.706308	3.402338
C	4.471417	6.083153	2.862810
C	2.691563	4.889617	4.143953
C	5.073754	4.185139	4.409694
H	5.440615	6.026635	2.356680
H	3.740990	6.473990	2.145676
H	4.564073	6.803374	3.684363
H	2.336157	3.948809	4.578335
H	2.820962	5.605528	4.962840
H	1.909923	5.278790	3.482101
H	5.174874	4.881862	5.250322
H	4.777273	3.208477	4.807740
H	6.059941	4.073216	3.947424
O	3.665557	1.056285	-0.982059
C	8.292411	-2.212208	-1.792979
C	9.025060	-2.324739	-0.438189
C	7.956948	-3.631405	-2.279806
C	9.227369	-1.551512	-2.829338
H	9.325823	-1.343532	-0.056167
H	8.381007	-2.794347	0.313398
H	9.930233	-2.934552	-0.543523
H	7.457673	-3.617861	-3.254682
H	8.879941	-4.211779	-2.386191
H	7.309679	-4.160053	-1.571745
H	10.133777	-2.153646	-2.964185
H	8.728450	-1.461149	-3.800489
H	9.536343	-0.548591	-2.516427
C	3.306061	-1.658559	-2.054974
O	3.159304	-2.869329	-2.099047

O 2.348376 -0.779287 -2.359368
C 7.505568 2.627242 0.048277
H 7.691987 2.043540 0.955758
H 8.319185 2.441204 -0.658659
H 7.549805 3.689876 0.303561
C 5.959566 3.143978 -1.858455
H 5.984298 4.208617 -1.600394
H 6.758988 2.936207 -2.578325
H 5.000844 2.928252 -2.338011
C 1.073729 -1.339540 -2.726897
H 0.698140 -1.979089 -1.929744
H 0.403512 -0.494200 -2.856524
H 1.173847 -1.921798 -3.647464

3-3tri

B3LYP SCF energy: -3815.82117701 a.u.
B3LYP enthalpy: -3814.321126 a.u.
B3LYP free energy: -3814.533753 a.u.
B3LYP SCF energy in solution: -3813.78445643 a.u.
B3LYP enthalpy in solution: -3812.284405 a.u.
B3LYP free energy in solution: -3812.497032 a.u.

Cartesian coordinates
ATOM X Y Z
N -2.935931 -0.225360 -1.103292
N -1.723869 2.299091 -0.951722
N 0.796463 1.095645 -1.239908
N -0.409382 -1.414493 -1.430744
C -3.329815 -1.537425 -0.934941
C -4.763476 -1.603953 -0.793607
C -5.238308 -0.330639 -0.908714
C -4.092989 0.529742 -1.065225
C -4.162740 1.921579 -1.069502
C -3.033701 2.735595 -1.004049
C -3.085653 4.169027 -0.856922
C -1.804286 4.594238 -0.669931
C -0.960817 3.426576 -0.735202
C 0.429956 3.466012 -0.634442
C 1.235310 2.360593 -0.892565
C 2.673859 2.418500 -0.953916
C 3.103355 1.198857 -1.379368
C 1.932667 0.369839 -1.533950
C 1.979554 -0.988699 -1.849977
C 0.867464 -1.817076 -1.766363
C 0.924773 -3.254438 -1.877441
C -0.310226 -3.724164 -1.551646
C -1.138340 -2.573069 -1.279294
C -2.488749 -2.653184 -0.950421
H -5.322139 -2.514922 -0.638029
H -6.261462 0.013927 -0.864615
H -3.994205 4.753506 -0.878737
H -1.442504 5.600660 -0.515777
H 3.260747 3.293827 -0.716855
H 4.117662 0.872577 -1.554302
H 1.810577 -3.811733 -2.146356
H -0.655088 -4.747558 -1.509283
Fe -1.053252 0.416991 -0.978877
C 1.087776 4.777748 -0.336955
C 1.540905 5.585501 -1.398072
C 1.241363 5.200655 0.996994
C 2.157334 6.805077 -1.105158
C 1.866248 6.426678 1.245343
C 2.336059 7.239724 0.210885
H 2.503327 7.430652 -1.925355
H 1.986326 6.753715 2.275978
C -3.057625 -3.979581 -0.561208
C -3.919637 -4.688988 -1.419235
C -2.734547 -4.497086 0.712213
C -4.506452 -5.873383 -0.961416
C -3.351053 -5.679333 1.132285
C -4.252549 -6.372652 0.318868
H -5.172180 -6.421637 -1.624684
C -5.513638 2.565858 -1.059864
C -6.150424 2.856239 -2.280546
C -6.144141 2.869442 0.161809
C -7.414651 3.451547 -2.260505
C -7.409157 3.463979 0.138578
C -8.059307 3.766030 -1.061116
H -7.907095 3.674155 -3.204748
H -7.898229 3.696168 1.082222
O -0.930325 0.193627 0.833287
C -5.476176 2.552434 1.478454
H -5.319420 1.474446 1.600925
H -4.490776 3.026118 1.550428
H -6.083304 2.900720 2.319123
C -5.478528 2.526514 -3.591905
H -4.507947 3.028690 -3.678406
H -5.284742 1.451074 -3.680559
H -6.099035 2.832868 -4.439217
C -9.410395 4.439499 -1.061419
H -9.996668 4.159949 -1.943146
H -9.989054 4.175993 -0.169820
H -9.308199 5.532828 -1.071783
C -4.191702 -4.206043 -2.825544
H -4.880681 -3.353815 -2.843075
H -3.268799 -3.873217 -3.312353
H -4.631819 -5.004650 -3.430512
C -4.938402 -7.621084 0.818671
H -5.314116 -8.230995 -0.009157
H -4.260034 -8.238744 1.417212
H -5.796028 -7.372773 1.458593
C 0.733508 4.358229 2.141036
H -0.356823 4.247377 2.090180
H 1.157741 3.348852 2.134527
H 0.976832 4.822001 3.101964
C 1.360113 5.150062 -2.832960
H 1.941961 4.247323 -3.053263
H 0.312461 4.910088 -3.047494
H 1.679675 5.936728 -3.522923
C 3.037902 8.542947 0.507712
H 2.900549 9.265638 -0.303652
H 2.666883 8.995359 1.433494
H 4.118847 8.392236 0.630073
H -3.121372 -6.064396 2.123481
C -1.712315 -3.829920 1.600173
H -0.695866 -4.099502 1.286908
H -1.773049 -2.741371 1.559077
H -1.838986 -4.140628 2.639893
C 3.336091 -1.584829 -2.051019
C 4.108152 -1.830955 -0.915824
C 3.922730 -1.784732 -3.303773
C 5.459071 -2.170333 -0.981360
C 5.262434 -2.175938 -3.426241
H 3.319275 -1.591953 -4.182502
C 4.235674 -1.051449 1.273691
C 6.190130 -2.340193 0.353446
C 6.014617 -2.339718 -2.249787
C 3.607287 -0.167343 2.158579
C 5.616983 -1.294438 1.322809
H 7.062256 -2.606063 -2.331252
C 4.402537 0.493300 3.104962
C 6.361865 -0.614171 2.282473
C 5.778627 0.289685 3.188732
H 3.891659 1.190539 3.756080
H 7.430693 -0.787365 2.332535
C 5.936074 -2.400519 -4.787650
C 6.457494 -3.852157 -4.865239
C 4.967882 -2.175352 -5.960811
C 7.119207 -1.420368 -4.943807
H 7.185946 -4.063692 -4.075807
H 5.633471 -4.566674 -4.760774
H 6.946718 -4.031687 -5.830095
H 4.590515 -1.147127 -5.984420
H 5.487578 -2.359548 -6.907370
H 4.109928 -2.855095 -5.914260
H 7.613311 -1.568873 -5.911435
H 6.772208 -0.382453 -4.891939
H 7.870161 -1.561948 -4.159871
O 3.488332 -1.661086 0.297703
C 6.664653 1.011037 4.213115
C 7.731266 1.844162 3.469367
C 5.854338 1.959043 5.112311
C 7.362996 -0.031586 5.112985
H 8.370214 1.216552 2.839321
H 7.257734 2.593400 2.825421
H 8.376947 2.366056 4.185877
H 5.092311 1.420187 5.685707
H 6.523735 2.451257 5.826468
H 5.354146 2.740280 4.529923
H 8.004604 0.467167 5.849343
H 6.624688 -0.633891 5.653783
H 7.990845 -0.714534 4.531087
C 2.147723 0.175232 2.115688
O 1.710692 1.188748 2.653025
C 7.709819 -2.212665 0.193051
H 7.993410 -1.229082 -0.195679
H 8.214820 -2.366623 1.151194
H 8.089673 -2.978221 -0.490083
C 5.858802 -3.749433 0.913638
H 6.222638 -4.524034 0.229066
H 6.335601 -3.889244 1.890175
H 4.779764 -3.877190 1.036572
H 0.467862 -0.330678 1.323605
H -0.798076 0.402960 3.699036
C -1.646736 0.289962 3.029589
C -2.586596 -0.732924 3.288911
C -1.605494 1.057544 1.757198
C -2.463403 -1.568261 4.437639
C -3.664124 -1.014004 2.406586
H -1.040530 1.986577 1.873919
H -2.603508 1.299696 1.380758
C -3.325264 -2.622475 4.659682
H -1.654530 -1.377163 5.137678
C -4.530739 -2.072941 2.622682
H -3.791748 -0.416528 1.514734
C -4.356680 -2.902216 3.741069
H -3.216299 -3.269974 5.523923
H -5.313517 -2.263899 1.900290
O -5.116029 -3.996424 4.013424
C -6.124340 -4.354682 3.077434
H -6.888822 -3.571344 2.995320
H -5.692783 -4.555439 2.090183
H -6.581968 -5.265046 3.468608
O 1.398599 -0.707677 1.479652

4-3tri

B3LYP SCF energy: -3795.95678324 a.u.
B3LYP enthalpy: -3794.443901 a.u.
B3LYP free energy: -3794.659363 a.u.
B3LYP SCF energy in solution: -3793.92868041 a.u.
B3LYP enthalpy in solution: -3792.415798 a.u.
B3LYP free energy in solution: -3792.631260 a.u.

Cartesian coordinates
ATOM X Y Z
N -2.960679 -0.224080 -1.069532
N -1.637951 2.258259 -0.974913
N 0.834614 0.940527 -1.140444
N -0.483068 -1.530637 -1.242737
C -3.422159 -1.509066 -0.874155
C -4.862934 -1.506706 -0.818866
C -5.269591 -0.219741 -1.009136
C -4.078172 0.583595 -1.125155
C -4.088144 1.975936 -1.170552
C -2.927325 2.741943 -1.081675
C -2.921348 4.178904 -0.993500
C -1.626758 4.560952 -0.794469
C -0.829691 3.362782 -0.793601
C 0.560091 3.356036 -0.678610
C 1.320074 2.206055 -0.872715
C 2.758557 2.206082 -0.946895
C 3.138598 0.950300 -1.309128
C 1.937423 0.155653 -1.403528
C 1.931365 -1.220214 -1.641695
C 0.784629 -1.999159 -1.521930
C 0.785091 -3.440100 -1.537427
C -0.478262 -3.840394 -1.216059
C -1.267302 -2.647237 -1.047998
C -2.634376 -2.657230 -0.781019
H -5.470694 -2.385710 -0.664536
H -6.275940 0.172390 -1.038362
H -3.804291 4.798186 -1.059601
H -1.228099 5.557741 -0.672920
H 3.378958 3.070700 -0.762668
H 4.139000 0.579910 -1.475388
H 1.653133 -4.048877 -1.746347
H -0.864767 -4.844806 -1.117248
Fe -1.052066 0.349376 -0.827344
C 1.267828 4.656272 -0.454255
C 1.736963 5.391445 -1.560402
C 1.452009 5.139965 0.855113
C 2.400364 6.601001 -1.337041
C 2.122440 6.354308 1.032696
C 2.609179 7.095586 -0.046819
H 2.759173 7.170681 -2.191703
H 2.265386 6.729290 2.043821
C -3.280533 -3.927977 -0.333440
C -4.119142 -4.667168 -1.189125
C -3.066479 -4.348789 0.996941
C -4.790751 -5.782890 -0.678914
C -3.766379 -5.463563 1.467630
C -4.645538 -6.182580 0.652557
H -5.439550 -6.354504 -1.339207
C -5.408019 2.678194 -1.239647
C -5.975854 2.964840 -2.494780
C -6.077837 3.039539 -0.054828
C -7.212608 3.613602 -2.546513
C -7.313139 3.687075 -0.150475

C -7.895571 3.985511 -1.385380
H -7.652331 3.832890 -3.517162
H -7.832913 3.964222 0.764124
O -1.032862 0.221309 1.047245
C -5.484521 2.727595 1.298106
H -5.397461 1.647646 1.462849
H -4.475584 3.142934 1.398356
H -6.102952 3.139845 2.100652
C -5.261510 2.574281 -3.766474
H -4.268184 3.034715 -3.822234
H -5.108722 1.489983 -3.821104
H -5.831272 2.883431 -4.647704
C -9.214702 4.715448 -1.462807
H -9.767441 4.447974 -2.369535
H -9.848086 4.489926 -0.598409
H -9.065019 5.803169 -1.481521
C -4.279753 -4.286086 -2.643035
H -4.906707 -3.395878 -2.769328
H -3.311054 -4.054292 -3.098867
H -4.739780 -5.101895 -3.208866
C -5.426313 -7.350588 1.204323
H -4.821715 -7.945346 1.897557
H -6.309021 -7.007516 1.760613
H -5.778693 -8.011051 0.405476
C 0.935433 4.372069 2.045865
H -0.149116 4.225180 1.977765
H 1.388681 3.377640 2.125974
H 1.142672 4.912511 2.974609
C 1.524079 4.890505 -2.969384
H 2.057475 3.948757 -3.144633
H 0.464061 4.694022 -3.167296
H 1.878500 5.622227 -3.701434
C 3.359809 8.386310 0.175920
H 4.434213 8.201919 0.309148
H 3.249758 9.065853 -0.675847
H 3.006457 8.904747 1.073570
H -3.624910 -5.770582 2.501423
C -2.073630 -3.652788 1.896334
H -1.057636 -4.018946 1.698123
H -2.056065 -2.572303 1.745539
H -2.301131 -3.843377 2.947583
C 3.256583 -1.874790 -1.863150
C 4.111757 -2.046405 -0.776311
C 3.738006 -2.204677 -3.134832
C 5.448679 -2.418319 -0.916510
C 5.054367 -2.640396 -3.326918
H 3.068907 -2.073727 -3.976536
C 4.445244 -1.057631 1.318729
C 6.290354 -2.471547 0.361952
C 5.897146 -2.712790 -2.203861
C 3.893442 -0.083389 2.154376
C 5.819962 -1.319691 1.263862
H 6.931080 -3.006443 -2.343033
C 4.778150 0.660666 2.945828
C 6.654793 -0.550754 2.071113
C 6.156657 0.452331 2.921327
H 4.326897 1.427265 3.562029
H 7.723022 -0.731431 2.041893
C 5.610557 -3.004017 -4.710899
C 6.105484 -4.466827 -4.692288
C 4.551597 -2.869491 -5.817627
C 6.788988 -2.066417 -5.053119
H 6.894254 -4.618666 -3.948413
H 5.284156 -5.151916 -4.454554
H 6.510894 -4.744987 -5.672337
H 4.188247 -1.840073 -5.909487
H 4.988509 -3.153632 -6.781045
H 3.690989 -3.522941 -5.636754
H 7.199722 -2.314200 -6.039102
H 6.460620 -1.021366 -5.071539
H 7.600137 -2.149987 -4.322642
O 3.606674 -1.771612 0.477538
C 7.133507 1.271773 3.775500
C 8.133452 2.000087 2.851107
C 6.410537 2.326710 4.628903
C 7.904014 0.326386 4.722622
H 8.711923 1.296522 2.243202
H 7.607935 2.678577 2.170068
H 8.841929 2.590542 3.444387
H 5.701985 1.867125 5.326193
H 7.143590 2.888209 5.218635
H 5.861389 3.042436 4.007862
H 8.610099 0.895228 5.339447
H 7.213561 -0.200786 5.390164
H 8.475172 -0.426251 4.168843
C 2.424299 0.276914 2.243808
O 2.091872 1.350190 2.759132
C 7.791795 -2.394756 0.059859
H 8.053362 -1.463505 -0.453150
H 8.374599 -2.458181 0.983303
H 8.101821 -3.235802 -0.567482
C 5.991944 -3.809987 1.089198
H 6.285839 -4.656992 0.459035
H 6.550509 -3.860004 2.030234
H 4.926682 -3.903487 1.317217
N 1.529438 -0.631758 1.800000
H 1.855670 -1.404433 1.239943
H 0.559455 -0.326436 1.645645
H -0.984576 0.472362 4.041896
C -1.756596 0.440356 3.277043
C -2.825789 -0.472828 3.441231
C -1.496419 1.171634 2.002722
C -2.930437 -1.280239 4.610847
C -3.816738 -0.669717 2.442492
H -0.729093 1.938281 2.158087
H -2.401331 1.668140 1.621769
C -3.919507 -2.234451 4.748915
H -2.194451 -1.152082 5.400361
C -4.814054 -1.625268 2.577757
H -3.766308 -0.095081 1.527239
C -4.860995 -2.434003 3.721758
H -3.979959 -2.861596 5.633045
H -5.526216 -1.754390 1.773774
O -5.761726 -3.439427 3.920954
C -6.666082 -3.730606 2.866986
H -7.333097 -2.882229 2.663485
H -6.131644 -4.005626 1.949829
H -7.260758 -4.579711 3.209796

5-3tri

B3LYP SCF energy: -3855.11497883 a.u.
B3LYP enthalpy: -3853.584466 a.u.
B3LYP free energy: -3853.801990 a.u.
B3LYP SCF energy in solution: -3853.04335066 a.u.
B3LYP enthalpy in solution: -3851.512838 a.u.
B3LYP free energy in solution: -3851.730362 a.u.

Cartesian coordinates

ATOM X Y Z
N 3.081580 -0.159871 0.931052
N 1.724203 2.297016 0.895339
N -0.728561 0.925436 0.861476
N 0.623902 -1.516502 0.842284
C 3.580491 -1.425904 0.700023
C 5.021653 -1.402056 0.759189
C 5.391342 -0.122216 1.049940
C 4.180682 0.657168 1.117589
C 4.159118 2.046404 1.231129
C 2.994742 2.799863 1.093908
C 2.972124 4.241077 1.045168
C 1.686874 4.607588 0.779088
C 0.911045 3.393502 0.703559
C -0.476271 3.368005 0.553209
C -1.227379 2.203369 0.687114
C -2.666014 2.192676 0.793123
C -3.032027 0.914025 1.080426
C -1.828387 0.118072 1.077538
C -1.805169 -1.271558 1.206021
C -0.643706 -2.023294 1.032122
C -0.619972 -3.465775 0.945881
C 0.663371 -3.823600 0.668335
C 1.437700 -2.606298 0.625356
C 2.821671 -2.578397 0.478191
H 5.655573 -2.261752 0.600770
H 6.386173 0.279773 1.177110
H 3.838525 4.871657 1.184026
H 1.278680 5.601302 0.664488
H -3.295080 3.063010 0.678585
H -4.026441 0.533772 1.257137
H -1.483770 -4.102781 1.065549
H 1.072794 -4.813943 0.528491
Fe 1.184408 0.388354 0.661593
C -1.195471 4.669635 0.372200
C -1.599878 5.400972 1.507846
C -1.462537 5.157170 -0.919475
C -2.270589 6.612528 1.329707
C -2.133677 6.377909 -1.052961
C -2.544863 7.119529 0.055392
H -2.585637 7.174239 2.206730
H -2.342025 6.753545 -2.052419
C 3.528355 -3.827889 0.059828
C 4.308038 -4.566337 0.971548
C 3.421656 -4.244521 -1.284404
C 5.034019 -5.667333 0.505904
C 4.171463 -5.345599 -1.708934
C 4.997734 -6.058482 -0.835319
H 5.636178 -6.236882 1.210712
C 5.457159 2.763428 1.436112
C 5.913026 3.003726 2.745664
C 6.217939 3.185816 0.329371
C 7.129337 3.666836 2.929287
C 7.429121 3.846404 0.556040
C 7.900656 4.099023 1.847011
H 7.481965 3.849687 3.942154
H 8.018448 4.171035 -0.298878
O 1.257940 0.370691 -1.129550
C 5.746434 2.926282 -1.081393
H 5.698342 1.853122 -1.299312
H 4.740763 3.327943 -1.246827
H 6.420931 3.385802 -1.809806
C 5.101014 2.548908 3.934649
H 4.102038 3.000309 3.929379
H 4.954857 1.462422 3.925114
H 5.593596 2.819429 4.873286
C 9.195131 4.843999 2.066899
H 9.684717 4.530353 2.994972
H 9.895341 4.682032 1.240661
H 9.022260 5.925999 2.139894
C 4.344021 -4.206270 2.439271
H 4.967511 -3.326177 2.633779
H 3.341257 -3.968570 2.810033
H 4.742633 -5.036080 3.030914
C 5.832639 -7.213410 -1.333194
H 5.295492 -7.801345 -2.085318
H 6.759427 -6.858392 -1.804084
H 6.117765 -7.883747 -0.515950
C -1.070732 4.375640 -2.149448
H -0.087911 3.907649 -2.032672
H -1.796642 3.579754 -2.352398
H -1.036913 5.027658 -3.028195
C -1.318246 4.884177 2.898690
H -1.783704 3.905332 3.062639
H -0.242644 4.754327 3.065752
H -1.700041 5.574706 3.656563
C -3.242153 8.447941 -0.113008
H -3.731258 8.521167 -1.089752
H -4.002370 8.601686 0.660747
H -2.531570 9.282016 -0.038976
H 4.107867 -5.649967 -2.751488
C 2.483834 -3.563580 -2.251144
H 1.463318 -3.948585 -2.128049
H 2.434924 -2.485357 -2.093255
H 2.790612 -3.745135 -3.283846
C -3.066268 -1.994164 1.542682
C -4.156657 -2.049153 0.671870
C -3.187069 -2.629538 2.787081
C -5.358190 -2.671504 1.026266
C -4.362375 -3.272191 3.182031
H -2.331774 -2.586544 3.449994
C -5.136652 -0.857367 -1.087271
C -6.465443 -2.704181 -0.027132
C -5.440694 -3.267736 2.283096
C -4.993427 0.293827 -1.872656
C -6.405702 -1.376698 -0.787810
H -6.366144 -3.751714 2.571831
C -6.149499 0.950012 -2.318649
C -7.523945 -0.693172 -1.258190
C -7.427134 0.482360 -2.021497
H -5.997482 1.851257 -2.897945
H -8.507269 -1.084781 -1.026392
C -4.511354 -3.965575 4.542949
C -4.831088 -5.460078 4.320909
C -3.230401 -3.868653 5.387976
C -5.663043 -3.304866 5.331251
H -5.759539 -5.596743 3.756993
H -4.025225 -5.950783 3.763842
H -4.944656 -5.973634 5.283074
H -2.966464 -2.828137 5.606598
H -3.380628 -4.381278 6.344313
H -2.377910 -4.340983 4.887456
H -5.787071 -3.791474 6.306062
H -5.456759 -2.242580 5.502183
H -6.615590 -3.379314 4.796585
O -4.015691 -1.457260 -0.565304
C -8.704263 1.189205 -2.493268
C -9.545341 1.596364 -1.263789
C -8.396066 2.456057 -3.308007
C -9.524437 0.228360 -3.381425

H	-9.830744	0.727782	-0.661102
H	-8.983926	2.281823	-0.619468
H	-10.465960	2.100435	-1.581176
H	-7.821194	2.226574	-4.211756
H	-9.333090	2.929833	-3.620447
H	-7.831438	3.188017	-2.720473
H	-10.443737	0.717283	-3.725264
H	-8.947329	-0.074283	-4.262119
H	-9.811469	-0.679475	-2.840432
C	-3.687120	0.901939	-2.262060
O	-3.572155	2.074028	-2.581571
C	-7.845402	-2.961056	0.591116
H	-8.119617	-2.179918	1.307741
H	-8.614248	-3.006738	-0.185579
H	-7.862894	-3.925955	1.105806
C	-6.146061	-3.847169	-1.028836
H	-6.143713	-4.812852	-0.510984
H	-6.899308	-3.874367	-1.824114
H	-5.164990	-3.701398	-1.489443
H	1.599568	0.829840	-3.947687
C	2.349437	0.663658	-3.177404
C	3.368614	-0.279869	-3.441075
C	2.107639	1.271652	-1.837426
C	3.432781	-0.965486	-4.690123
C	4.354481	-0.622705	-2.477493
H	1.613686	2.248802	-1.936733
H	3.045709	1.436124	-1.299211
C	4.375597	-1.943976	-4.931101
H	2.703100	-0.722262	-5.458124
C	5.301993	-1.606408	-2.712564
H	4.348464	-0.134859	-1.513711
C	5.308794	-2.293704	-3.935047
H	4.406639	-2.478188	-5.875527
H	6.007895	-1.850097	-1.928865
O	6.159412	-3.310232	-4.244981
C	7.075341	-3.734395	-3.244959
H	7.776662	-2.932593	-2.979492
H	6.549068	-4.075528	-2.345817
H	7.629281	-4.566725	-3.682914
O	-2.683192	0.021356	-2.273439
C	-1.384694	0.541287	-2.606571
H	-1.104758	1.331321	-1.912394
H	-0.693931	-0.289150	-2.492605
H	-1.386216	0.928486	-3.630230

6

B3LYP SCF energy: -540.78089548 a.u.
B3LYP enthalpy: -540.552031 a.u.
B3LYP free energy: -540.598661 a.u.
B3LYP SCF energy in solution: -540.42309936 a.u.
B3LYP enthalpy in solution: -540.194235 a.u.
B3LYP free energy in solution: -540.240865 a.u.

Cartesian coordinates

ATOM	X	Y	Z
C	-3.543729	0.697607	-0.525355
C	-2.399003	1.390524	-0.129773
C	-1.253805	0.701882	0.275981
C	-1.253798	-0.701768	0.276207
C	-2.398987	-1.390566	-0.129305
C	-3.543724	-0.697801	-0.525118
C	-0.000012	1.424691	0.720518
C	-0.000016	-1.424430	0.721017
C	1.253790	-0.701782	0.276252
C	1.253788	0.701865	0.276010
C	2.399009	1.390521	-0.129664
H	2.391849	2.478025	-0.138665
C	3.543752	0.697615	-0.525204
C	3.543739	-0.697796	-0.525004
C	2.398998	-1.390572	-0.129226
H	0.000004	2.456530	0.352666
H	-4.427765	1.244905	-0.840329
H	-2.391863	2.478028	-0.138755
H	-2.391825	-2.478073	-0.137933
H	-4.427764	-1.245213	-0.839878
H	-0.000025	-1.494471	1.821840
H	4.427804	1.244909	-0.840137
H	4.427783	-1.245197	-0.839775
H	2.391833	-2.478078	-0.137878
H	-0.000016	1.495088	1.821323
H	-0.000020	-2.456394	0.353501

7

B3LYP SCF energy: -540.14959744 a.u.
B3LYP enthalpy: -539.934131 a.u.
B3LYP free energy: -539.983072 a.u.
B3LYP SCF energy in solution: -539.79269516 a.u.
B3LYP enthalpy in solution: -539.577229 a.u.
B3LYP free energy in solution: -539.626170 a.u.

Cartesian coordinates

ATOM	X	Y	Z
C	-3.687812	0.755203	-0.025954
C	-2.474267	1.423322	-0.000393
C	-1.247766	0.713771	0.018216
C	-1.282127	-0.707758	0.013609
C	-2.513093	-1.359767	-0.014641
C	-3.713235	-0.646003	-0.034605
C	0.000000	1.397559	0.033014
C	0.000000	-1.509740	0.054426
C	1.282127	-0.707758	0.013609
C	1.247766	0.713771	0.018216
C	2.474267	1.423322	-0.000393
H	2.446311	2.510105	0.003492
C	3.687812	0.755203	-0.025954
C	3.713235	-0.646003	-0.034605
C	2.513093	-1.359767	-0.014641
H	0.000000	2.484373	0.037942
H	-4.617331	1.317347	-0.040612
H	-2.446311	2.510105	0.003492
H	-2.534797	-2.447691	-0.019901
H	-4.660654	-1.176423	-0.056562
H	0.000000	-2.130278	0.964697
H	4.617331	1.317347	-0.040612
H	4.660654	-1.176423	-0.056561
H	2.534797	-2.447691	-0.019901
H	0.000000	-2.232893	-0.774896

8-4qua

B3LYP SCF energy: -3374.44117338 a.u.
B3LYP enthalpy: -3390.890101 a.u.
B3LYP free energy: -3391.087191 a.u.
B3LYP SCF energy in solution: -3390.48939158 a.u.
B3LYP enthalpy in solution: -3406.938319 a.u.
B3LYP free energy in solution: -3407.135409 a.u.

Cartesian coordinates

ATOM	X	Y	Z
N	-3.344599	0.827555	0.081142
N	-2.327681	-1.756488	0.568819
N	0.234863	-0.668140	0.939504
N	-0.785569	1.913576	0.490657
C	-3.637734	2.127928	-0.275618
C	-5.040664	2.254645	-0.574573
C	-5.601237	1.028124	-0.378666
C	-4.538202	0.137314	0.009534
C	-4.720770	-1.224197	0.231240
C	-3.665547	-2.093647	0.491922
C	-3.826404	-3.513805	0.659570
C	-2.577785	-4.041846	0.807295
C	-1.648381	-2.944887	0.753989
C	-0.270755	-3.092335	0.899125
C	0.590535	-2.004402	0.992708
C	1.997383	-2.129320	1.264364
C	2.488791	-0.868585	1.413348
C	1.392520	0.041421	1.195807
C	1.527604	1.429013	1.204218
C	0.494965	2.286611	0.850466
C	0.651604	3.710931	0.723261
C	-0.530166	4.200374	0.254405
C	-1.424260	3.079583	0.118073
C	-2.746807	3.197420	-0.297266
H	-5.518216	3.174744	-0.878944
H	-6.633590	0.731271	-0.494463
H	-4.778400	-4.024657	0.651610
H	-2.293166	-5.074149	0.950688
H	2.521121	-3.070700	1.341738
H	3.501977	-0.568711	1.634966
H	1.562127	4.246129	0.951077
H	-0.795298	5.222331	0.025036
Fe	-1.501736	0.047775	0.233370
C	0.291186	-4.472304	1.045027
C	0.512712	-4.999703	2.332059
C	0.582505	-5.238083	-0.100154
C	1.037927	-6.289035	2.454191
C	1.107917	-6.523007	0.067980
C	1.348343	-7.064001	1.333523
H	1.204730	-6.697828	3.448552
H	1.333172	-7.115949	-0.815901
C	-3.236879	4.533139	-0.758692
C	-3.852512	5.416825	0.146487
C	-3.066355	4.895894	-2.108268
C	-4.296576	6.658234	-0.318144
C	-3.525619	6.145791	-2.532552
C	-4.146306	7.038910	-1.654471
H	-4.769025	7.344337	0.381629
C	-6.104825	-1.785480	0.138063
C	-6.937233	-1.783119	1.272238
C	-6.568058	-2.304751	-1.085692
C	-8.228655	-2.306799	1.164443
C	-7.866480	-2.817717	-1.152108
C	-8.710477	-2.831359	-0.037935
H	-8.873303	-2.303441	2.040797
H	-8.227465	-3.214444	-2.098602
O	-1.172344	-0.163612	-1.639319
C	-5.680137	-2.304448	-2.306837
H	-5.318291	-1.296293	-2.538117
H	-4.791514	-2.927785	-2.152825
H	-6.215953	-2.686118	-3.180830
C	-6.445365	-1.219268	2.583749
H	-5.528386	-1.721074	2.913809
H	-6.205618	-0.153133	2.493952
H	-7.199465	-1.333938	3.367927
C	-10.097319	-3.421291	-0.125239
H	-10.083834	-4.498467	0.087938
H	-10.776493	-2.955532	0.596486
H	-10.523899	-3.294100	-1.125831
C	-4.025094	5.035180	1.597267
H	-4.664683	4.151129	1.704038
H	-3.063334	4.785682	2.060196
H	-4.477049	5.852689	2.166680
C	-4.663500	8.370307	-2.143345
H	-4.048473	8.762925	-2.960049
H	-5.689939	8.281413	-2.523449
H	-4.677494	9.113814	-1.339530
C	0.331257	-4.690478	-1.483262
H	-0.730629	-4.456239	-1.627910
H	0.884154	-3.763493	-1.664636
H	0.619895	-5.420185	-2.246183
C	0.182837	-4.193184	3.565743
H	0.813388	-3.299378	3.640284
H	-0.856312	-3.845125	3.548677
H	0.329565	-4.788250	4.471983
C	1.948122	-8.440955	1.485978
H	1.630534	-8.914883	2.420809
H	1.661110	-9.096357	0.656868
H	3.045333	-8.395992	1.499946
H	-3.393549	6.428399	-3.574784
C	-2.389622	3.955362	-3.076491
H	-1.343414	3.781360	-2.798512
H	-2.873429	2.972339	-3.086697
H	-2.408527	4.359406	-4.092903
C	2.906677	1.964478	1.423073
C	3.796274	1.889221	0.352959
C	3.400227	2.379675	2.663456
C	5.168772	2.091399	0.495505
C	4.760346	2.662274	2.845987
H	2.706305	2.435997	3.493415
C	4.011065	0.591610	-1.555661
C	6.029377	1.849855	-0.749458
C	5.627689	2.483419	1.753101
C	3.341626	-0.429754	-2.233162
C	5.410020	0.655869	-1.494044
H	6.689317	2.651472	1.894295
C	4.108883	-1.422792	-2.854825
C	6.130693	-0.346326	-2.139086
C	5.503391	-1.401057	-2.827593
H	3.565943	-2.222565	-3.341624
H	7.213689	-0.316729	-2.103234
C	5.334675	3.118517	4.194986
C	6.018954	4.491759	4.018825
C	4.247487	3.259024	5.273301
C	6.372362	2.086443	4.687916
H	6.833705	4.447578	3.288925
H	5.299778	5.242424	3.672828
H	6.440134	4.834014	4.971663
H	3.750159	2.304076	5.475733
H	4.700407	3.601641	6.209999
H	3.483726	3.990006	4.985777
H	6.793962	2.398520	5.650870
H	5.908334	1.102721	4.819778
H	7.200833	1.973998	3.981178
O	3.270026	1.516872	-0.859949
C	6.362730	-2.479663	-3.501036
C	7.239666	-3.173715	-2.435970
C	5.506563	-3.553462	-4.192427
C	7.269346	-1.823711	-4.565091
H	7.906093	-2.464930	-1.933453

H	6.616147	-3.648378	-1.670302
H	7.862920	-3.947835	-2.899539
H	4.875657	-3.124106	-4.978198
H	6.158548	-4.300251	-4.658820
H	4.858046	-4.074788	-3.480012
H	7.894003	-2.580693	-5.054393
H	6.667887	-1.326466	-5.334014
H	7.935588	-1.074590	-4.124520
C	1.848678	-0.559466	-2.242232
O	1.303798	-1.660336	-2.299975
O	1.198281	0.586860	-2.196720
H	0.197383	0.383098	-2.034310
C	7.499965	1.609503	-0.389227
H	7.619474	0.733426	0.256710
H	8.099286	1.459704	-1.292184
H	7.916618	2.479947	0.126563
C	5.930813	3.098799	-1.665197
H	6.331395	3.978693	-1.149154
H	6.504255	2.938320	-2.584853
H	4.892062	3.302357	-1.938940
H	-0.918635	-1.085728	-1.831609

9-4qua

B3LYP SCF energy: -3372.35155381 a.u.
B3LYP enthalpy: -3371.007662 a.u.
B3LYP free energy: -3371.205093 a.u.
B3LYP SCF energy in solution: -3370.63764692 a.u.
B3LYP enthalpy in solution: -3369.293755 a.u.
B3LYP free energy in solution: -3369.491186 a.u.

Cartesian coordinates

ATOM	X	Y	Z
N	-3.460314	0.763746	-0.030562
N	-2.415864	-1.789303	0.507461
N	0.142562	-0.674177	0.827045
N	-0.898550	1.875244	0.268651
C	-3.766887	2.041694	-0.449430
C	-5.183948	2.159539	-0.680232
C	-5.738052	0.952395	-0.374198
C	-4.657591	0.078513	0.006933
C	-4.826302	-1.271795	0.302804
C	-3.754561	-2.133306	0.522870
C	-3.893979	-3.551390	0.723779
C	-2.634115	-4.070691	0.792811
C	-1.717228	-2.968873	0.669177
C	-0.331383	-3.101578	0.755680
C	0.517986	-2.004140	0.856110
C	1.929415	-2.110423	1.125118
C	2.395900	-0.845752	1.308772
C	1.286783	0.050332	1.085699
C	1.405937	1.439353	1.044160
C	0.373490	2.273724	0.624969
C	0.525274	3.690128	0.410111
C	-0.652629	4.142662	-0.105223
C	-1.542632	3.012669	-0.175526
C	-2.873922	3.103771	-0.571383
H	-5.674740	3.062551	-1.013558
H	-6.776752	0.657260	-0.412537
H	-4.840658	-4.068315	0.787831
H	-2.334175	-5.099181	0.931862
H	2.471841	-3.042816	1.174146
H	3.403547	-0.534921	1.540512
H	1.427604	4.246908	0.617240
H	-0.918804	5.147815	-0.398932
Fe	-1.604762	0.001633	0.100378
C	0.246988	-4.479406	0.825913
C	0.585112	-5.038241	2.073373
C	0.427329	-5.215975	-0.361728
C	1.127445	-6.325952	2.112567
C	0.973936	-6.499691	-0.275315
C	1.338502	-7.068407	0.947949
H	1.385059	-6.759978	3.076399
H	1.117741	-7.069010	-1.191027
C	-3.378822	4.410632	-1.095777
C	-3.932706	5.362701	-0.220256
C	-3.286508	4.676954	-2.474942
C	-4.391489	6.575031	-0.743769
C	-3.757197	5.900942	-2.958055
C	-4.315951	6.861103	-2.109679
H	-4.816521	7.313556	-0.067353
C	-6.213306	-1.832954	0.325393
C	-6.960689	-1.795141	1.516744
C	-6.765283	-2.387930	-0.844615
C	-8.256382	-2.319316	1.519412
C	-8.064776	-2.900458	-0.800778
C	-8.824930	-2.879051	0.372064
H	-8.835135	-2.288227	2.440074
H	-8.493702	-3.325121	-1.705877
O	-1.262957	-0.287196	-1.736018
C	-5.970132	-2.425651	-2.127649
H	-5.641651	-1.422554	-2.422966
H	-5.065265	-3.034742	-2.016684
H	-6.563961	-2.845642	-2.944658
C	-6.374369	-1.193193	2.771214
H	-5.440451	-1.692906	3.053524
H	-6.133349	-0.133075	2.629104
H	-7.072761	-1.274601	3.609277
C	-10.214131	-3.468902	0.404475
H	-10.184684	-4.538348	0.652077
H	-10.839800	-2.978047	1.157248
H	-10.711673	-3.374360	-0.566504
C	-4.026287	5.083715	1.260812
H	-4.647616	4.203164	1.461900
H	-3.039232	4.877375	1.690627
H	-4.459742	5.935285	1.793483
C	-4.847892	8.162776	-2.658998
H	-4.278962	8.489163	-3.536033
H	-5.896101	8.062463	-2.970664
H	-4.806235	8.960210	-1.909693
C	0.040454	-4.638713	-1.701181
H	-1.024570	-4.377012	-1.724921
H	0.612973	-3.732528	-1.929987
H	0.225146	-5.363922	-2.499657
C	0.354609	-4.273207	3.355599
H	1.014258	-3.400623	3.429359
H	-0.672977	-3.896881	3.415795
H	0.537645	-4.909323	4.226751
C	1.960463	-8.442659	1.006800
H	1.778625	-8.923756	1.973627
H	1.564428	-9.095200	0.221275
H	3.048482	-8.390903	0.867017
H	-3.684151	6.109053	-4.023384
C	-2.683116	3.659702	-3.413358
H	-1.636870	3.453004	-3.160063
H	-3.208222	2.699713	-3.354593
H	-2.721350	4.010260	-4.448911
C	2.747360	2.030591	1.328643
C	3.767077	1.893147	0.388722
C	3.052157	2.656718	2.542806
C	5.085614	2.271653	0.645600
C	4.349089	3.092185	2.836044
H	2.253445	2.766205	3.266238
C	4.371127	0.419155	-1.326747
C	6.109130	2.016615	-0.463784
C	5.352313	2.868574	1.877155
C	3.914442	-0.732217	-1.972341
C	5.732388	0.685668	-1.129908
H	6.368159	3.172161	2.101491
C	4.879126	-1.644045	-2.421573
C	6.648954	-0.253229	-1.596396
C	6.245897	-1.431855	-2.247700
H	4.495906	-2.540487	-2.890829
H	7.706970	-0.069020	-1.451065
C	4.711803	3.776297	4.161597
C	5.283676	5.180929	3.869804
C	3.493707	3.936124	5.086253
C	5.772788	2.932375	4.901128
H	6.182049	5.131448	3.246076
H	4.546135	5.799695	3.346681
H	5.552473	5.685606	4.805388
H	3.061558	2.967321	5.359809
H	3.797651	4.436798	6.011848
H	2.709066	4.543527	4.621718
H	6.045522	3.408937	5.850220
H	5.388386	1.929891	5.119105
H	6.686755	2.819329	4.309161
O	3.442845	1.317323	-0.822530
C	7.307649	-2.429780	-2.729261
C	8.134037	-2.919480	-1.520032
C	6.681000	-3.655930	-3.413142
C	8.242839	-1.734504	-3.742466
H	8.641368	-2.092184	-1.012596
H	7.490074	-3.418173	-0.787283
H	8.900575	-3.633256	-1.844939
H	6.097009	-3.372721	-4.295322
H	7.472566	-4.338875	-3.741002
H	6.023661	-4.207359	-2.732512
H	9.010958	-2.433114	-4.095371
H	7.677816	-1.379157	-4.611221
H	8.753243	-0.872912	-3.299190
C	2.464118	-1.122275	-2.180855
O	2.193402	-2.301866	-2.426915
C	7.545578	2.011035	0.073068
H	7.693321	1.227834	0.823778
H	8.260259	1.852427	-0.739591
H	7.791468	2.976560	0.525056
C	5.973393	3.145159	-1.521090
H	6.202971	4.116886	-1.069469
H	6.666797	2.969041	-2.350700
H	4.958141	3.184144	-1.925158
N	1.539521	-0.140415	-2.138497
H	0.536628	-0.368108	-2.108507
H	1.801512	0.764995	-1.779372
H	-1.659791	-1.128149	-2.016808

10-4qua

B3LYP SCF energy: -3431.51211505 a.u.
B3LYP enthalpy: -3430.151260 a.u.
B3LYP free energy: -3430.353727 a.u.
B3LYP SCF energy in solution: -3429.74736549 a.u.
B3LYP enthalpy in solution: -3428.386510 a.u.
B3LYP free energy in solution: -3428.588977 a.u.

Cartesian coordinates

ATOM	X	Y	Z
N	-3.629672	0.643311	-0.073598
N	-2.472633	-1.891725	0.370093
N	0.088714	-0.711086	0.491619
N	-1.067427	1.820125	0.067080
C	-4.003328	1.938122	-0.369025
C	-5.437936	2.033460	-0.443036
C	-5.933520	0.790079	-0.178808
C	-4.803145	-0.076674	0.031525
C	-4.906681	-1.448792	0.245676
C	-3.797433	-2.280608	0.376483
C	-3.891740	-3.712567	0.511173
C	-2.617612	-4.191422	0.565388
C	-1.737438	-3.053225	0.485748
C	-0.350693	-3.147555	0.590705
C	0.476592	-2.029387	0.639776
C	1.879816	-2.098202	0.959593
C	2.328298	-0.816732	1.051762
C	1.219295	0.045325	0.727602
C	1.309771	1.434159	0.636121
C	0.230949	2.237264	0.264872
C	0.342001	3.650401	-0.007089
C	-0.892189	4.080925	-0.386910
C	-1.773055	2.942605	-0.320154
C	-3.143641	3.020353	-0.547807
H	-5.979221	2.944325	-0.655089
H	-6.964782	0.470263	-0.135699
H	-4.821391	-4.261707	0.550639
H	-2.283683	-5.213873	0.664936
H	2.428471	-3.016601	1.106385
H	3.322350	-0.478542	1.301135
H	1.256225	4.219714	0.068457
H	-1.199857	5.075468	-0.675930
Fe	-1.737486	-0.073298	-0.089661
C	0.257450	-4.503351	0.783828
C	0.416689	-5.003741	2.092604
C	0.647585	-5.275353	-0.325199
C	0.976514	-6.270345	2.270493
C	1.199645	-6.541887	-0.101597
C	1.379016	-7.053841	1.184214
H	1.096161	-6.656559	3.280635
H	1.497415	-7.140682	-0.959635
C	-3.728420	4.331361	-0.969712
C	-4.082792	5.294837	-0.007258
C	-3.916451	4.590972	-2.340162
C	-4.620645	6.511866	-0.435610
C	-4.454799	5.821094	-2.727699
C	-4.815972	6.793189	-1.790684
H	-4.892733	7.258081	0.307902
C	-6.271073	-2.061826	0.296637
C	-6.927122	-2.205730	1.532864
C	-6.892695	-2.486201	-0.892718
C	-8.203068	-2.775820	1.559433
C	-8.168679	-3.052213	-0.823824
C	-8.839078	-3.208501	0.392680
H	-8.712051	-2.883329	2.514932
H	-8.650259	-3.378405	-1.743220
O	-1.623819	-0.262341	-1.945065
C	-6.195208	-2.328214	-2.222539
H	-6.004916	-1.272132	-2.448117
H	-5.221812	-2.831933	-2.224043
H	-6.798294	-2.745906	-3.034007
C	-6.264520	-1.748544	2.810173
H	-5.326641	-2.288201	2.986296

H	-6.010975	-0.682892	2.767947
H	-6.918662	-1.910928	3.671873
C	-10.203247	-3.852745	0.447255
H	-10.122721	-4.942545	0.555758
H	-10.786275	-3.483710	1.297562
H	-10.774127	-3.659134	-0.467043
C	-3.882932	5.021587	1.464235
H	-4.393513	4.101332	1.770391
H	-2.821643	4.887599	1.704280
H	-4.268287	5.845668	2.071909
C	-5.426651	8.102235	-2.229524
H	-5.061724	8.402529	-3.217401
H	-6.520257	8.027154	-2.295628
H	-5.198510	8.907148	-1.522926
C	0.504873	-4.756103	-1.734625
H	-0.423561	-4.191754	-1.866806
H	1.337494	-4.089743	-1.988131
H	0.509473	-5.582278	-2.452948
C	-0.016653	-4.189411	3.288499
H	0.527051	-3.239113	3.343905
H	-1.082905	-3.940118	3.236371
H	0.160363	-4.737493	4.218660
C	2.009854	-8.408194	1.400416
H	1.583001	-8.914639	2.273008
H	1.872638	-9.056722	0.528973
H	3.090548	-8.318522	1.573726
H	-4.593609	6.024847	-3.787341
C	-3.545272	3.555409	-3.374756
H	-2.511628	3.214297	-3.249567
H	-4.181685	2.666052	-3.288465
H	-3.657203	3.954304	-4.387241
C	2.593203	2.107674	0.986289
C	3.768220	1.919322	0.254092
C	2.643803	2.956912	2.102140
C	4.974934	2.523810	0.623542
C	3.823193	3.584408	2.507016
H	1.725629	3.096863	2.658864
C	4.825216	0.351879	-1.125541
C	6.179589	2.291594	-0.287688
C	4.981162	3.341801	1.751487
C	4.673933	-0.921465	-1.689489
C	6.094370	0.847668	-0.788104
H	5.910175	3.812911	2.049753
C	5.816540	-1.714225	-1.870301
C	7.199238	0.025119	-0.991474
C	7.090112	-1.269509	-1.525372
H	5.654406	-2.701357	-2.282560
H	8.181200	0.400017	-0.728405
C	3.892858	4.511053	3.728169
C	4.360419	5.912520	3.278295
C	2.529343	4.659818	4.423205
C	4.898109	3.936995	4.749996
H	5.349364	5.879346	2.809722
H	3.660001	6.341070	2.552811
H	4.419829	6.589818	4.138584
H	2.156267	3.699069	4.794541
H	2.625334	5.333225	5.281816
H	1.775563	5.083662	3.750430
H	4.965025	4.589922	5.628412
H	4.585030	2.942153	5.085405
H	5.902338	3.845993	4.323409
O	3.706917	1.106941	-0.857882
C	8.350287	-2.124482	-1.710028
C	9.032699	-2.329304	-0.339911
C	8.029088	-3.507064	-2.300753
C	9.324432	-1.402351	-2.666191
H	9.322168	-1.376767	0.116193
H	8.360176	-2.843491	0.355466
H	9.939332	-2.935800	-0.451962
H	7.565392	-3.427155	-3.289910
H	8.953865	-4.083781	-2.411773
H	7.355215	-4.077743	-1.652695
H	10.233704	-1.998844	-2.806941
H	8.861674	-1.246436	-3.646899
H	9.624131	-0.423453	-2.277391
C	3.374169	-1.527506	-2.107608
O	3.219897	-2.733124	-2.221504
O	2.436385	-0.621959	-2.389513
C	7.507583	2.587441	0.420408
H	7.651274	1.946275	1.296318
H	8.350009	2.439538	-0.261367
H	7.546884	3.631464	0.744062
C	6.047029	3.235324	-1.513981
H	6.066003	4.281551	-1.188717
H	6.875231	3.066851	-2.211268
H	5.108370	3.057135	-2.045950
C	1.154956	-1.139695	-2.798774
H	0.769810	-1.828642	-2.049881
H	0.488462	-0.285182	-2.867930
H	1.252707	-1.657452	-3.757661
H	-2.500979	-0.121603	-2.337058

TS-1-3tri

B3LYP SCF energy: -3815.79025711 a.u.
B3LYP enthalpy: -3814.292262 a.u.
B3LYP free energy: -3814.505599 a.u.
B3LYP SCF energy in solution: -3813.75161699 a.u.
B3LYP enthalpy in solution: -3812.253622 a.u.
B3LYP free energy in solution: -3812.466959 a.u.
Imaginary frequency: -452.3621 cm-1

Cartesian coordinates

ATOM	X	Y	Z
N	-2.919448	-0.257071	-1.109668
N	-1.698016	2.264378	-0.925925
N	0.808209	1.058845	-1.260842
N	-0.406997	-1.457528	-1.417608
C	-3.325708	-1.565933	-0.947907
C	-4.760719	-1.624980	-0.826557
C	-5.225237	-0.348293	-0.938911
C	-4.072136	0.505335	-1.072318
C	-4.138666	1.895835	-1.049568
C	-3.007352	2.703087	-0.962797
C	-3.057484	4.136567	-0.813733
C	-1.773655	4.562299	-0.651815
C	-0.931841	3.394566	-0.732848
C	0.460233	3.440794	-0.683389
C	1.255506	2.331186	-0.947404
C	2.691969	2.384503	-1.030962
C	3.114061	1.152362	-1.425590
C	1.940803	0.321851	-1.544278
C	1.987137	-1.044001	-1.822836
C	0.873621	-1.867844	-1.726402
C	0.930450	-3.307104	-1.805711
C	-0.309108	-3.769282	-1.486836
C	-1.139722	-2.613281	-1.251554
C	-2.492751	-2.686077	-0.939920
H	-5.326363	-2.534024	-0.685663
H	-6.245986	0.004270	-0.903943
H	-3.966587	4.720416	-0.823046
H	-1.408366	5.569125	-0.509643
H	3.282672	3.264942	-0.824617
H	4.125378	0.818125	-1.602603
H	1.819208	-3.869937	-2.052251
H	-0.656514	-4.790980	-1.428014
Fe	-1.029093	0.373552	-0.935921
C	1.125414	4.762398	-0.450950
C	1.544851	5.528844	-1.556162
C	1.316917	5.238957	0.859635
C	2.166603	6.759819	-1.330109
C	1.945421	6.474896	1.040717
C	2.382845	7.246879	-0.038320
H	2.486037	7.353025	-2.184401
H	2.094335	6.842788	2.053649
C	-3.072500	-4.000066	-0.526431
C	-3.911152	-4.736655	-1.383517
C	-2.788211	-4.470691	0.774049
C	-4.511003	-5.905380	-0.902499
C	-3.415931	-5.638669	1.216234
C	-4.292954	-6.360422	0.400673
H	-5.159719	-6.475203	-1.564430
C	-5.486943	2.546316	-1.059610
C	-6.109365	2.819769	-2.292022
C	-6.128890	2.874443	0.149669
C	-7.370603	3.421536	-2.295797
C	-7.390454	3.475095	0.102277
C	-8.026490	3.759673	-1.109058
H	-7.851300	3.631064	-3.249033
H	-7.888255	3.725688	1.036574
O	-0.889469	0.146328	0.729192
C	-5.479005	2.577640	1.479781
H	-5.319812	1.502536	1.619477
H	-4.497534	3.057560	1.562432
H	-6.100356	2.933687	2.306647
C	-5.425894	2.466028	-3.591170
H	-4.442842	2.945151	-3.666258
H	-5.255823	1.385883	-3.670996
H	-6.026872	2.781106	-4.449273
C	-9.373954	4.439787	-1.135317
H	-9.952383	4.147928	-2.018231
H	-9.963255	4.194720	-0.245446
H	-9.265902	5.532235	-1.163539
C	-4.146712	-4.295293	-2.809715
H	-4.806496	-3.421867	-2.866504
H	-3.206314	-4.009503	-3.293337
H	-4.604001	-5.099331	-3.394307
C	-4.994318	-7.590504	0.923672
H	-5.352721	-8.225617	0.107176
H	-4.332230	-8.190466	1.557419
H	-5.865438	-7.319088	1.535252
C	0.846488	4.442367	2.051026
H	-0.243868	4.323386	2.035849
H	1.278162	3.436504	2.072325
H	1.113297	4.946645	2.984926
C	1.321884	5.038780	-2.967350
H	1.906393	4.134598	-3.174288
H	0.270517	4.780632	-3.137947
H	1.609690	5.802800	-3.695643
C	3.089571	8.561908	0.185730
H	2.927067	9.251283	-0.649606
H	2.744255	9.050505	1.103017
H	4.173969	8.417678	0.282726
H	-3.218461	-5.987318	2.227449
C	-1.798317	-3.763804	1.668059
H	-0.769796	-4.006827	1.372959
H	-1.890488	-2.677550	1.612405
H	-1.932084	-4.064841	2.709609
C	3.343103	-1.647869	-2.003168
C	4.112818	-1.862894	-0.859858
C	3.929660	-1.888332	-3.248759
C	5.461326	-2.214145	-0.914119
C	5.267431	-2.289689	-3.358300
H	3.327558	-1.719459	-4.133320
C	4.244179	-1.031486	1.311776
C	6.190199	-2.355753	0.425039
C	6.016919	-2.423839	-2.176517
C	3.621897	-0.124144	2.177165
C	5.623440	-1.283436	1.368328
H	7.062721	-2.699781	-2.248736
C	4.420522	0.554014	3.107859
C	6.371986	-0.587222	2.313780
C	5.795011	0.341919	3.198104
H	3.914938	1.270530	3.742007
H	7.439305	-0.767663	2.369582
C	5.941447	-2.557435	-4.711655
C	6.453250	-4.014125	-4.747100
C	4.976390	-2.359154	-5.892208
C	7.131380	-1.590034	-4.893581
H	7.179290	-4.207921	-3.950912
H	5.624360	-4.719917	-4.623585
H	6.942511	-4.224272	-5.705723
H	4.605168	-1.329765	-5.944873
H	5.496371	-2.572589	-6.832435
H	4.114361	-3.032297	-5.828304
H	7.626054	-1.769503	-5.855639
H	6.791325	-0.548746	-4.872104
H	7.880084	-1.714128	-4.104546
O	3.491997	-1.652974	0.346897
C	6.685351	1.079709	4.206884
C	7.760156	1.886253	3.445844
C	5.881502	2.055221	5.082146
C	7.373407	0.052611	5.132193
H	8.395131	1.238674	2.832222
H	7.294130	2.623955	2.783369
H	8.408694	2.419567	4.151258
H	5.114249	1.536318	5.666884
H	6.553817	2.558564	5.785687
H	5.388753	2.826720	4.480706
H	8.017709	0.563146	5.858036
H	6.629186	-0.530864	5.685372
H	7.996726	-0.648772	4.567610
C	2.164734	0.224904	2.122603
O	1.738259	1.275979	2.588357
C	7.710944	-2.242580	0.263360
H	8.001723	-1.270800	-0.149012
H	8.213823	-2.376467	1.225607
H	8.086418	-3.027286	-0.400148
C	5.848613	-3.748694	1.018931
H	6.207763	-4.542148	0.353770
H	6.323324	-3.868016	1.999191
H	4.768561	-3.866015	1.143630
H	0.487521	-0.337529	1.353004
H	-0.827483	0.384465	3.893131
C	-1.680384	0.425205	3.218404
C	-2.685687	-0.601625	3.384613
C	-1.640717	1.346870	2.202562
C	-2.630890	-1.479000	4.490661
C	-3.699160	-0.826567	2.432010

H -0.830193 2.061638 2.167655
H -2.509515 1.567467 1.596418
C -3.525544 -2.527514 4.630074
H -1.856338 -1.334359 5.239439
C -4.603021 -1.872870 2.560001
H -3.746130 -0.207878 1.545329
C -4.510532 -2.745263 3.653531
H -3.470739 -3.210077 5.472054
H -5.342342 -2.022453 1.785004
O -5.310214 -3.830707 3.839776
C -6.266456 -4.129394 2.832121
H -7.005687 -3.324141 2.730990
H -5.780351 -4.309371 1.866486
H -6.769555 -5.040479 3.161181
O 1.408913 -0.703138 1.559502

TS-1-5qui

B3LYP SCF energy: -3815.77774912 a.u.
B3LYP enthalpy: -3814.281596 a.u.
B3LYP free energy: -3814.500862 a.u.
B3LYP SCF energy in solution: -3813.75220313 a.u.
B3LYP enthalpy in solution: -3812.256050 a.u.
B3LYP free energy in solution: -3812.475316 a.u.
Imaginary frequency: -111.0043 cm-1

Cartesian coordinates

ATOM X Y Z
N 3.684064 0.930139 -0.206192
N 2.615684 -1.519201 -1.270985
N 0.059085 -0.248487 -1.626851
N 1.144692 2.214412 -0.625994
C 3.990788 2.153677 0.343230
C 5.368837 2.161813 0.778029
C 5.887225 0.935371 0.478971
C 4.825280 0.164813 -0.126920
C 4.954182 -1.163219 -0.551923
C 3.916551 -1.931650 -1.091449
C 4.047169 -3.314790 -1.492060
C 2.809039 -3.734063 -1.881022
C 1.914495 -2.608312 -1.727513
C 0.538675 -2.647909 -1.997892
C -0.311303 -1.537612 -1.951485
C -1.711716 -1.562504 -2.305136
C -2.169447 -0.282374 -2.219685
C -1.062987 0.535782 -1.773183
C -1.147167 1.906869 -1.493265
C -0.113178 2.672611 -0.946082
C -0.237076 4.062927 -0.570512
C 0.946451 4.429043 -0.002089
C 1.808245 3.268746 -0.038104
C 3.120679 3.246566 0.445878
H 5.863981 3.004971 1.238263
H 6.891474 0.572984 0.646854
H 4.969316 -3.877741 -1.468839
H 2.513766 -4.708331 -2.243158
H -2.259321 -2.452167 -2.579161
H -3.167188 0.080299 -2.418532
H -1.122564 4.663802 -0.719877
H 1.227092 5.390715 0.403253
Fe 1.740919 0.210635 -0.490808
C -0.050256 -3.972063 -2.372704
C -0.333339 -4.261580 -3.721273
C -0.298437 -4.929836 -1.368260
C -0.888878 -5.502777 -4.044890
C -0.858102 -6.156412 -1.737518
C -1.168200 -6.459478 -3.065911
H -1.102555 -5.728134 -5.087651
H -1.056148 -6.894812 -0.963389
C 3.629956 4.476296 1.131056
C 4.294896 5.475290 0.397862
C 3.430678 4.620641 2.516960
C 4.754958 6.612120 1.068958
C 3.906196 5.771774 3.150585
C 4.573587 6.777078 2.444806
H 5.266029 7.387416 0.502050
C 6.288068 -1.821780 -0.380209
C 7.252876 -1.729165 -1.399526
C 6.568630 -2.527504 0.804638
C 8.491274 -2.351102 -1.216382
C 7.818684 -3.135142 0.949433
C 8.791998 -3.061569 -0.051139
H 9.238568 -2.277895 -2.003662
H 8.037293 -3.677810 1.866692
O 1.269943 -0.295746 1.047551
C 5.539717 -2.617415 1.906135
H 5.281440 -1.622210 2.287202
H 4.608697 -3.070053 1.545800
H 5.910244 -3.216334 2.743181
C 6.955988 -0.967096 -2.668564
H 6.075928 -1.374183 -3.179744
H 6.738034 0.086439 -2.457725
H 7.803163 -1.009224 -3.359449
C 10.123383 -3.754257 0.112092
H 10.911605 -3.243967 -0.451337
H 10.427465 -3.794151 1.163435
H 10.079351 -4.788939 -0.253218
C 4.503729 5.323894 -1.089734
H 5.098185 4.431657 -1.318635
H 3.548779 5.209503 -1.615637
H 5.020803 6.194268 -1.504401
C 5.107762 7.997044 3.155836
H 6.123453 7.822126 3.535011
H 5.155373 8.861888 2.485825
H 4.483009 8.265329 4.014418
C 0.042338 -4.652861 0.076787
H 1.129167 -4.606929 0.221643
H -0.375240 -3.701076 0.415614
H -0.348637 -5.448031 0.720160
C -0.028984 -3.260693 -4.811409
H -0.693696 -2.390580 -4.757599
H 0.995023 -2.879838 -4.727279
H -0.146234 -3.714638 -5.800039
C -1.805364 -7.778623 -3.430292
H -1.565521 -8.068761 -4.458713
H -1.473101 -8.581488 -2.763414
H -2.899583 -7.724889 -3.353235
H 3.750278 5.885997 4.221270
C 2.710249 3.550986 3.303044
H 1.687588 3.406707 2.935420
H 3.214113 2.581785 3.210118
H 2.659935 3.811582 4.364429
C -2.483494 2.551689 -1.684034
C -3.486295 2.293277 -0.751053
C -2.805244 3.336998 -2.796022
C -4.805997 2.713191 -0.924706
C -4.105350 3.815251 -3.000019
H -2.021131 3.538890 -3.515607
C -4.053743 0.616744 0.753934
C -5.804231 2.318815 0.169108
C -5.092096 3.472743 -2.058645
C -3.596469 -0.628879 1.188876
C -5.420667 0.908844 0.641401
H -6.109019 3.812163 -2.218969
C -4.545112 -1.604174 1.523962
C -6.327174 -0.089526 0.990477
C -5.915644 -1.358342 1.439395
H -4.161692 -2.569542 1.825575
H -7.387795 0.113655 0.898267
C -4.488596 4.672580 -4.214691
C -5.043762 6.028851 -3.727377
C -3.287937 4.948586 -5.134811
C -5.570175 3.940687 -5.038932
H -5.930172 5.901453 -3.097545
H -4.291263 6.568383 -3.141584
H -5.326804 6.655199 -4.581809
H -2.869123 4.022946 -5.544582
H -3.606244 5.570571 -5.978478
H -2.489281 5.483807 -4.609434
H -5.857858 4.540969 -5.910353
H -5.197500 2.974407 -5.396289
H -6.473164 3.753832 -4.448757
O -3.131963 1.553525 0.351274
C -6.968251 -2.432439 1.748208
C -7.702639 -2.795537 0.438400
C -6.336609 -3.710967 2.325191
C -7.993612 -1.895728 2.769525
H -8.203642 -1.924225 0.002697
H -6.997540 -3.183411 -0.304804
H -8.462656 -3.564005 0.624990
H -5.777233 -3.507134 3.243572
H -7.124682 -4.435918 2.558181
H -5.656782 -4.182867 1.607548
H -8.748652 -2.660598 2.985743
H -7.504019 -1.626259 3.710789
H -8.517743 -1.008879 2.398637
C -2.150329 -1.020226 1.179686
O -1.812523 -2.173584 0.964690
O -1.319539 -0.016476 1.423227
H -0.357783 -0.290056 1.294510
C -7.254040 2.391660 -0.324266
H -7.427584 1.715550 -1.167845
H -7.949356 2.130318 0.479009
H -7.502702 3.409656 -0.638814
C -5.628215 3.295370 1.362679
H -5.858535 4.320336 1.050861
H -6.301853 3.016435 2.180817
H -4.601501 3.272144 1.738302
H 1.368910 -3.265068 2.775966
C 1.042875 -2.313627 3.193319
C -0.304286 -2.308823 3.738309
C 1.885468 -1.258165 3.094629
C -1.059774 -3.500191 3.733882
C -0.932930 -1.145156 4.214529
H 2.869917 -1.374648 2.660581
H 1.658914 -0.284838 3.514060
C -2.376698 -3.526599 4.159020
H -0.602666 -4.410908 3.357128
C -2.258255 -1.150565 4.629950
H -0.395878 -0.203398 4.213555
C -2.992263 -2.344354 4.594975
H -2.961416 -4.440316 4.136347
H -2.716333 -0.223205 4.950642
O -4.303066 -2.448279 4.954160
C -5.002686 -1.251646 5.263190
H -4.569246 -0.748092 6.136414
H -5.009105 -0.567633 4.406132
H -6.024063 -1.556452 5.494974

TS-2-3tri

B3LYP SCF energy: -3795.92426547 a.u.
B3LYP enthalpy: -3794.412596 a.u.
B3LYP free energy: -3794.627130 a.u.
B3LYP SCF energy in solution: -3793.90010231 a.u.
B3LYP enthalpy in solution: -3792.388433 a.u.
B3LYP free energy in solution: -3792.602967 a.u.
Imaginary frequency: -472.6166 cm-1

Cartesian coordinates

ATOM X Y Z
N -2.965626 -0.252142 -1.091320
N -1.643221 2.226492 -0.971436
N 0.818630 0.905088 -1.198225
N -0.494467 -1.571897 -1.259391
C -3.428810 -1.534444 -0.878962
C -4.868103 -1.529122 -0.789064
C -5.275946 -0.241029 -0.968109
C -4.084682 0.557939 -1.111214
C -4.093186 1.950494 -1.139177
C -2.931989 2.714989 -1.053883
C -2.926768 4.154922 -0.960673
C -1.630177 4.535062 -0.789821
C -0.835092 3.331633 -0.809442
C 0.556976 3.324148 -0.740391
C 1.310959 2.172914 -0.943641
C 2.749345 2.170067 -1.018713
C 3.127803 0.906303 -1.350794
C 1.924626 0.113011 -1.433016
C 1.921158 -1.268765 -1.634367
C 0.775219 -2.046985 -1.513838
C 0.776457 -3.490869 -1.511062
C -0.489937 -3.885435 -1.203689
C -1.278972 -2.685923 -1.056928
C -2.644041 -2.685685 -0.788632
H -5.474623 -2.406639 -0.620673
H -6.281448 0.154465 -0.971034
H -3.810889 4.773966 -1.011203
H -1.226559 5.531226 -0.679853
H 3.371433 3.036750 -0.850411
H 4.128164 0.530871 -1.505151
H 1.646594 -4.102056 -1.703803
H -0.879995 -4.888182 -1.101233
Fe -1.057102 0.310596 -0.893444
C 1.274018 4.627022 -0.560946
C 1.715450 5.333187 -1.697300
C 1.496531 5.143145 0.729623
C 2.389402 6.544826 -1.522619
C 2.176933 6.358125 0.858786
C 2.636500 7.070369 -0.251581
H 2.726258 7.091442 -2.400974
H 2.349478 6.757084 1.856057
C -3.293981 -3.950650 -0.328873
C -4.137125 -4.693608 -1.176175
C -3.076695 -4.363301 1.003687
C -4.806605 -5.806935 -0.657464
C -3.773324 -5.476386 1.482979
C -4.655005 -6.200626 0.674796
H -5.459023 -6.381176 -1.311886
C -5.413139 2.654235 -1.201718

C	-6.001644	2.903595	-2.455658
C	-6.062732	3.055469	-0.018728
C	-7.237077	3.554745	-2.507398
C	-7.297670	3.703839	-0.114091
C	-7.899826	3.965480	-1.347725
H	-7.691601	3.745590	-3.477256
H	-7.801479	4.010889	0.799915
O	-0.986403	0.131686	0.778047
C	-5.449852	2.785330	1.334414
H	-5.337582	1.711208	1.519153
H	-4.450946	3.227795	1.417762
H	-6.070684	3.199051	2.134375
C	-5.309969	2.472281	-3.726730
H	-4.306948	2.908281	-3.800804
H	-5.182955	1.383879	-3.762118
H	-5.881964	2.777773	-4.607811
C	-9.217758	4.697420	-1.426508
H	-9.789698	4.397745	-2.311022
H	-9.834307	4.507778	-0.541555
H	-9.064883	5.783006	-1.490099
C	-4.306777	-4.316157	-2.629760
H	-4.923606	-3.418278	-2.751584
H	-3.339582	-4.097388	-3.095128
H	-4.782201	-5.127856	-3.188735
C	-5.430994	-7.367746	1.235324
H	-4.818978	-7.964011	1.920827
H	-6.306826	-7.024356	1.802316
H	-5.793620	-8.027273	0.440347
C	1.006952	4.411336	1.953974
H	-0.084275	4.303574	1.934517
H	1.429779	3.403699	2.033698
H	1.270975	4.958485	2.864183
C	1.461926	4.798757	-3.087069
H	1.992972	3.854519	-3.255817
H	0.397067	4.594878	-3.247723
H	1.791800	5.514042	-3.846364
C	3.398640	8.362438	-0.082094
H	4.476573	8.176865	0.016729
H	3.260639	9.024772	-0.943327
H	3.078829	8.900647	0.816498
H	-3.626488	-5.779027	2.517438
C	-2.085045	-3.656617	1.896358
H	-1.062459	-3.982676	1.666502
H	-2.106085	-2.572996	1.767190
H	-2.281580	-3.878457	2.947811
C	3.251628	-1.923770	-1.823671
C	4.102524	-2.054809	-0.727279
C	3.743642	-2.288790	-3.081508
C	5.443274	-2.418777	-0.849081
C	5.064717	-2.718648	-3.253354
H	3.078288	-2.188990	-3.930408
C	4.413953	-1.012741	1.349431
C	6.279132	-2.428570	0.434089
C	5.901786	-2.748407	-2.124437
C	3.848252	-0.034191	2.172324
C	5.791710	-1.261110	1.306329
H	6.939022	-3.036815	-2.249009
C	4.721543	0.731897	2.955301
C	6.614580	-0.469063	2.103574
C	6.102627	0.541951	2.935790
H	4.258752	1.499448	3.561702
H	7.684795	-0.638539	2.081489
C	5.631575	-3.119665	-4.622580
C	6.141056	-4.576068	-4.555965
C	4.576885	-3.030552	-5.737946
C	6.802232	-2.181208	-4.988255
H	6.927744	-4.696635	-3.804160
H	5.325483	-5.261699	-4.300636
H	6.553966	-4.880575	-5.525007
H	4.204115	-2.008166	-5.864075
H	5.021262	-3.340791	-6.689842
H	3.721686	-3.686214	-5.540272
H	7.220540	-2.455336	-5.964046
H	6.463365	-1.140686	-5.040809
H	7.610371	-2.233661	-4.251550
O	3.587817	-1.748381	0.515891
C	7.067230	1.387965	3.777763
C	8.058745	2.113940	2.842524
C	6.329004	2.447291	4.612468
C	7.848825	0.468925	4.741576
H	8.647059	1.407947	2.247014
H	7.525274	2.774044	2.149726
H	8.758692	2.723337	3.426706
H	5.625853	1.990058	5.316771
H	7.053643	3.028533	5.193393
H	5.771086	3.144931	3.978779
H	8.546163	1.057497	5.349763
H	7.164468	-0.056330	5.416880
H	8.431104	-0.285030	4.201340
C	2.374780	0.304143	2.260146
O	2.019815	1.365830	2.785329
C	7.781113	-2.341714	0.136909
H	8.034217	-1.420582	-0.398131
H	8.360405	-2.375463	1.064120
H	8.103862	-3.194405	-0.467789
C	5.993084	-3.751696	1.193752
H	6.299807	-4.610685	0.586357
H	6.547571	-3.771463	2.138348
H	4.927811	-3.852075	1.418960
N	1.495281	-0.614999	1.804409
H	1.837693	-1.366459	1.224686
H	0.537871	-0.305627	1.609930
H	-0.856995	0.537766	3.920552
C	-1.699465	0.604249	3.234758
C	-2.776094	-0.339478	3.434566
C	-1.580333	1.458288	2.162027
C	-2.809495	-1.150177	4.591662
C	-3.776411	-0.556772	2.465419
H	-0.715700	2.105991	2.101667
H	-2.434188	1.736236	1.557708
C	-3.775353	-2.128143	4.764710
H	-2.047295	-1.010787	5.353957
C	-4.750774	-1.533237	2.626921
H	-3.754598	0.005819	1.541240
C	-4.745623	-2.341376	3.772403
H	-3.788566	-2.760497	5.646679
H	-5.475501	-1.683419	1.838273
O	-5.618820	-3.361737	3.996712
C	-6.553388	-3.669839	2.972029
H	-7.239032	-2.832025	2.789927
H	-6.043627	-3.939670	2.039880
H	-7.122151	-4.527224	3.336683

TS-2-5qui

B3LYP SCF energy: -3795.91780013 a.u.
B3LYP enthalpy: -3794.407538 a.u.
B3LYP free energy: -3794.624445 a.u.
B3LYP SCF energy in solution: -3793.90210773 a.u.
B3LYP enthalpy in solution: -3792.391846 a.u.
B3LYP free energy in solution: -3792.608753 a.u.
Imaginary frequency: -115.4388 cm-1

Cartesian coordinates

ATOM	X	Y	Z
N	-3.061158	-0.208169	-1.039397
N	-1.658155	2.310480	-1.134123
N	0.847754	0.902324	-1.284575
N	-0.554880	-1.604002	-1.236926
C	-3.537130	-1.486116	-0.861831
C	-4.976899	-1.447795	-0.725123
C	-5.355946	-0.141245	-0.827526
C	-4.148895	0.635816	-0.996713
C	-4.113399	2.034141	-1.050397
C	-2.943397	2.800960	-1.113468
C	-2.911020	4.246656	-1.101067
C	-1.597638	4.615049	-1.081536
C	-0.817830	3.397737	-1.094868
C	0.582879	3.353476	-1.067546
C	1.342630	2.183413	-1.166485
C	2.785846	2.152701	-1.234005
C	3.149143	0.852912	-1.419606
C	1.932002	0.069321	-1.430634
C	1.882448	-1.329690	-1.532972
C	0.716966	-2.096569	-1.429392
C	0.683465	-3.542752	-1.424992
C	-0.609368	-3.910055	-1.196292
C	-1.380571	-2.693209	-1.074351
C	-2.758685	-2.654033	-0.831459
H	-5.606190	-2.313399	-0.579458
H	-6.352973	0.273225	-0.781374
H	-3.785508	4.881524	-1.099240
H	-1.180084	5.611490	-1.062758
H	3.423264	3.021891	-1.161414
H	4.146989	0.454567	-1.528191
H	1.544676	-4.179020	-1.570740
H	-1.020840	-4.906862	-1.123047
Fe	-1.076560	0.351344	-0.714076
C	1.316809	4.653416	-0.949476
C	1.770723	5.309488	-2.108785
C	1.539386	5.211677	0.324224
C	2.456547	6.520033	-1.975802
C	2.229924	6.424411	0.411072
C	2.701231	7.090116	-0.723610
H	2.803705	7.030263	-2.871848
H	2.402141	6.858929	1.393453
C	-3.446199	-3.924408	-0.441331
C	-4.320492	-4.580888	-1.328613
C	-3.240861	-4.429670	0.861370
C	-5.027019	-5.700836	-0.878517
C	-3.975245	-5.546265	1.271741
C	-4.884865	-6.185269	0.424483
H	-5.702083	-6.207765	-1.564817
C	-5.419794	2.763937	-0.982150
C	-6.119346	3.054855	-2.167678
C	-5.942587	3.149595	0.266659
C	-7.339381	3.731656	-2.084727
C	-7.165481	3.825591	0.306364
C	-7.877570	4.128334	-0.857451
H	-7.881321	3.954375	-3.001429
H	-7.571011	4.122154	1.271325
O	-0.996911	0.373248	0.956882
C	-5.205864	2.829618	1.545277
H	-5.154459	1.748725	1.719162
H	-4.173177	3.194799	1.513531
H	-5.701894	3.283794	2.407940
C	-5.562068	2.639600	-3.508085
H	-4.572338	3.078340	-3.680023
H	-5.438498	1.551957	-3.568624
H	-6.221697	2.954310	-4.322119
C	-9.179873	4.889112	-0.792360
H	-9.840909	4.622886	-1.623890
H	-9.712714	4.690865	0.143592
H	-9.008957	5.972520	-0.846324
C	-4.485926	-4.104713	-2.753449
H	-5.083703	-3.187796	-2.812407
H	-3.516077	-3.876619	-3.208400
H	-4.981218	-4.867371	-3.362035
C	-5.701470	-7.357020	0.912654
H	-5.121382	-7.997105	1.586085
H	-6.584097	-7.017921	1.471620
H	-6.058207	-7.972028	0.080107
C	1.049882	4.518861	1.572107
H	-0.024687	4.309954	1.515075
H	1.552010	3.558571	1.736430
H	1.227515	5.140999	2.454657
C	1.517198	4.721384	-3.476571
H	2.028620	3.759254	-3.599051
H	0.449471	4.533142	-3.637913
H	1.868549	5.395299	-4.263587
C	3.473841	8.381036	-0.597625
H	4.545666	8.189203	-0.454430
H	3.370993	8.998741	-1.496043
H	3.132930	8.969105	0.261184
H	-3.836401	-5.919257	2.283904
C	-2.229124	-3.815035	1.798455
H	-1.212187	-4.123954	1.526210
H	-2.246689	-2.723662	1.769580
H	-2.413929	-4.129298	2.827896
C	3.195100	-2.029427	-1.680886
C	4.072543	-2.085326	-0.599025
C	3.633355	-2.540950	-2.907855
C	5.388558	-2.533184	-0.717246
C	4.927811	-3.046195	-3.070531
H	2.945223	-2.499154	-3.743278
C	4.496553	-0.899232	1.383876
C	6.256585	-2.487048	0.542451
C	5.793679	-3.008304	-1.964256
C	4.010564	0.160501	2.156703
C	5.856305	-1.232540	1.332364
H	6.810620	-3.363321	-2.083060
C	4.946354	0.918718	2.872692
C	6.743386	-0.442078	2.059211
C	6.313085	0.647671	2.835427
H	4.542669	1.744689	3.443337
H	7.800603	-0.677903	2.026958
C	5.434505	-3.606745	-4.406772
C	5.874003	-5.074072	-4.209363
C	4.353352	-3.572028	-5.499546
C	6.638644	-2.769632	-4.890589
H	6.675320	-5.161438	-3.468482
H	5.033638	-5.688861	-3.868343
H	6.243339	-5.492039	-5.153357
H	4.025287	-2.549122	-5.714320
H	4.754854	-3.993003	-6.427637
H	3.474427	-4.162878	-5.218933
H	7.015255	-3.158723	-5.844031
H	6.349665	-1.723177	-5.037747
H	7.463785	-2.791460	-4.171362

O	3.606783	-1.636359	0.620302
C	7.345639	1.484771	3.602129
C	8.351131	2.095327	2.601558
C	6.690345	2.632230	4.388051
C	8.099985	0.580964	4.601438
H	8.882310	1.322232	2.036337
H	7.837384	2.743102	1.882728
H	9.099796	2.696622	3.131188
H	5.981117	2.258509	5.134274
H	7.461440	3.203701	4.916439
H	6.156258	3.322560	3.726477
H	8.844574	1.163753	5.156838
H	7.405615	0.136185	5.322757
H	8.625433	-0.235269	4.094485
C	2.562642	0.587747	2.274483
O	2.287540	1.676197	2.793891
C	7.752931	-2.507500	0.206098
H	8.041291	-1.645897	-0.404854
H	8.354053	-2.502626	1.119818
H	8.013256	-3.420935	-0.336633
C	5.920459	-3.729899	1.410038
H	6.164581	-4.648923	0.865366
H	6.497557	-3.707135	2.340935
H	4.857522	-3.753456	1.665816
N	1.618134	-0.280645	1.853460
H	1.891617	-1.071378	1.290100
H	0.661127	0.054606	1.728623
H	-0.594907	-0.078333	4.356363
C	-1.428045	0.266219	3.745014
C	-2.599438	-0.596384	3.729741
C	-1.260660	1.407499	3.032665
C	-2.765317	-1.580825	4.726677
C	-3.560894	-0.541164	2.703458
H	-0.312158	1.934227	3.060757
H	-2.071034	1.868786	2.481018
C	-3.833508	-2.463533	4.704022
H	-2.031931	-1.651180	5.526050
C	-4.635641	-1.422780	2.667117
H	-3.422638	0.154067	1.884228
C	-4.768278	-2.404115	3.659025
H	-3.952655	-3.228677	5.464425
H	-5.327103	-1.368372	1.837977
O	-5.745560	-3.351513	3.675114
C	-6.614888	-3.423540	2.553538
H	-7.215800	-2.510607	2.451590
H	-6.051677	-3.601510	1.630566
H	-7.276091	-4.270835	2.743690

TS-3-3tri

B3LYP SCF energy: -3855.08599169 a.u.
B3LYP enthalpy: -3853.557299 a.u.
B3LYP free energy: -3853.774995 a.u.
B3LYP SCF energy in solution: -3853.01255574 a.u.
B3LYP enthalpy in solution: -3851.483863 a.u.
B3LYP free energy in solution: -3851.701559 a.u.
Imaginary frequency: -519.3869 cm-1

Cartesian coordinates

ATOM	X	Y	Z
N	3.066273	-0.181282	0.920707
N	1.710262	2.275671	0.868664
N	-0.741081	0.910330	0.880050
N	0.614678	-1.536598	0.861522
C	3.567167	-1.446825	0.699499
C	5.007547	-1.425380	0.773672
C	5.375114	-0.144863	1.063058
C	4.163913	0.636074	1.112376
C	4.144681	2.025682	1.214913
C	2.981987	2.778283	1.061595
C	2.961170	4.219322	1.006470
C	1.674879	4.586785	0.747318
C	0.896822	3.373562	0.683689
C	-0.493104	3.351859	0.562079
C	-1.241820	2.188276	0.711772
C	-2.679508	2.176193	0.830696
C	-3.042306	0.896329	1.114708
C	-1.837846	0.100842	1.099583
C	-1.814988	-1.289099	1.219039
C	-0.654299	-2.041093	1.041578
C	-0.633700	-3.482398	0.937122
C	0.648560	-3.840146	0.653827
C	1.426260	-2.625305	0.627168
C	2.808537	-2.597855	0.473139
H	5.641410	-2.287719	0.629778
H	6.368528	0.257411	1.200266
H	3.829092	4.849083	1.139448
H	1.266761	5.580685	0.634201
H	-3.309666	3.046751	0.724268
H	-4.034974	0.513782	1.296550
H	-1.499208	-4.118511	1.048811
H	1.055378	-4.829494	0.499870
Fe	1.163305	0.364549	0.634664
C	-1.213673	4.656329	0.406575
C	-1.615536	5.363808	1.558170
C	-1.482036	5.172549	-0.873694
C	-2.288374	6.577994	1.407171
C	-2.155317	6.394613	-0.979995
C	-2.566481	7.111404	0.144710
H	-2.601142	7.121061	2.296654
H	-2.365216	6.791594	-1.970824
C	3.511962	-3.840120	0.029516
C	4.276263	-4.610465	0.926591
C	3.415827	-4.213843	-1.327967
C	4.994212	-5.706166	0.436139
C	4.155751	-5.311618	-1.777030
C	4.964057	-6.059537	-0.915759
H	5.584934	-6.301410	1.129299
C	5.438609	2.742004	1.448661
C	5.870789	2.961807	2.770246
C	6.218445	3.184601	0.363436
C	7.082064	3.624003	2.986787
C	7.423892	3.843824	0.623026
C	7.871828	4.075769	1.926060
H	7.415735	3.790808	4.008804
H	8.027706	4.183614	-0.215724
O	1.185447	0.308785	-1.046210
C	5.775090	2.948661	-1.060324
H	5.724321	1.879342	-1.294657
H	4.777155	3.361433	-1.243527
H	6.468843	3.412432	-1.767621
C	5.039197	2.486370	3.937475
H	4.035478	2.926499	3.915343
H	4.905648	1.398458	3.914870
H	5.509084	2.753330	4.888673
C	9.160473	4.819387	2.181736
H	9.636472	4.487511	3.110547
H	9.873928	4.676758	1.363320
H	8.983217	5.899124	2.274912
C	4.303625	-4.287533	2.402898
H	4.904792	-3.397131	2.619405
H	3.295081	-4.083540	2.778573
H	4.721948	-5.121688	2.974378
C	5.790331	-7.209619	-1.438503
H	5.256970	-7.764639	-2.217760
H	6.729254	-6.852712	-1.883151
H	6.053192	-7.910422	-0.639511
C	-1.085967	4.420628	-2.120419
H	-0.086230	3.984864	-2.023787
H	-1.788565	3.604493	-2.323693
H	-1.085555	5.085231	-2.990215
C	-1.327154	4.820521	2.937497
H	-1.807839	3.847605	3.092572
H	-0.252083	4.669402	3.089217
H	-1.687844	5.505895	3.710232
C	-3.266248	8.441905	0.005737
H	-3.763811	8.531861	-0.965342
H	-4.019844	8.581294	0.788586
H	-2.555685	9.275216	0.088165
H	4.102189	-5.582984	-2.829037
C	2.499866	-3.485739	-2.281273
H	1.464694	-3.827383	-2.153293
H	2.497759	-2.407463	-2.112546
H	2.791711	-3.670137	-3.317689
C	-3.079144	-2.013849	1.537656
C	-4.160178	-2.057172	0.654490
C	-3.212753	-2.664992	2.772401
C	-5.365144	-2.684196	0.988154
C	-4.392188	-3.312566	3.146610
H	-2.364304	-2.630720	3.444586
C	-5.121714	-0.842735	-1.099373
C	-6.460992	-2.703485	-0.077172
C	-5.460799	-3.296566	2.236309
C	-4.971073	0.317626	-1.869667
C	-6.393731	-1.365888	-0.819171
H	-6.389174	-3.784302	2.508864
C	-6.123265	0.979299	-2.318067
C	-7.507605	-0.676463	-1.291180
C	-7.403654	0.508682	-2.038350
H	-5.965759	1.887119	-2.885430
H	-8.492996	-1.071178	-1.073890
C	-4.555688	-4.023497	4.496742
C	-4.872950	-5.514980	4.251950
C	-3.283856	-3.937619	5.356612
C	-5.715807	-3.372995	5.281166
H	-5.795289	-5.644244	3.676386
H	-4.061142	-5.998506	3.697240
H	-4.996794	-6.040922	5.206116
H	-3.022264	-2.900033	5.591370
H	-3.444316	-4.462480	6.304613
H	-2.426064	-4.403605	4.859217
H	-5.850285	-3.872251	6.248164
H	-5.511412	-2.313049	5.468142
H	-6.662550	-3.440376	4.735370
O	-4.005993	-1.448854	-0.573377
C	-8.676156	1.221801	-2.513119
C	-9.528810	1.613397	-1.286574
C	-8.360100	2.498873	-3.308669
C	-9.487987	0.272575	-3.421212
H	-9.819990	0.737231	-0.697789
H	-8.973473	2.290436	-0.628233
H	-10.446330	2.121684	-1.606207
H	-7.776964	2.280756	-4.209933
H	-9.294071	2.976969	-3.623670
H	-7.800688	3.223011	-2.706631
H	-10.403926	0.766064	-3.767495
H	-8.902577	-0.018933	-4.300182
H	-9.780254	-0.642018	-2.894584
C	-3.662809	0.929750	-2.246517
O	-3.549041	2.104389	-2.557429
C	-7.847288	-2.969453	0.522741
H	-8.129811	-2.198171	1.246778
H	-8.607737	-3.005282	-0.262682
H	-7.869488	-3.941169	1.024255
C	-6.130171	-3.832777	-1.090646
H	-6.132906	-4.805336	-0.585821
H	-6.874769	-3.849577	-1.894310
H	-5.144263	-3.680447	-1.538627
H	1.764134	0.933129	-4.110493
C	2.479268	0.824983	-3.296684
C	3.503239	-0.176765	-3.481484
C	2.261492	1.541606	-2.136646
C	3.586735	-0.899254	-4.693928
C	4.421446	-0.515793	-2.467601
H	1.530068	2.340472	-2.133475
H	3.035363	1.625949	-1.386918
C	4.512708	-1.914066	-4.871872
H	2.894671	-0.661572	-5.497944
C	5.354550	-1.530117	-2.632582
H	4.377849	-0.007513	-1.514247
C	5.395497	-2.252163	-3.833412
H	4.562757	-2.477670	-5.798035
H	6.016674	-1.770081	-1.811258
O	6.236180	-3.295228	-4.076096
C	7.118180	-3.690396	-3.034658
H	7.818254	-2.884836	-2.777316
H	6.563526	-3.997125	-2.140550
H	7.677384	-4.542018	-3.426479
O	-2.656581	0.051782	-2.262546
C	-1.362817	0.576957	-2.608600
H	-1.078609	1.367619	-1.916960
H	-0.667921	-0.251106	-2.501371
H	-1.377092	0.964416	-3.631999

TS-3-5qui

B3LYP SCF energy: -3855.07837100 a.u.
B3LYP enthalpy: -3853.550941 a.u.
B3LYP free energy: -3853.771079 a.u.
B3LYP SCF energy in solution: -3853.01317050 a.u.
B3LYP enthalpy in solution: -3851.485740 a.u.
B3LYP free energy in solution: -3851.705878 a.u.
Imaginary frequency: -241.2281 cm-1

Cartesian coordinates

ATOM	X	Y	Z
N	3.137155	-0.126344	0.932416
N	1.675302	2.361468	0.917449
N	-0.812054	0.901324	0.904186
N	0.650129	-1.571626	0.895235
C	3.652756	-1.390617	0.780800
C	5.098182	-1.325526	0.800726
C	5.439384	-0.014554	0.964845
C	4.205652	0.738367	1.015583
C	4.135526	2.135744	1.066912
C	2.948655	2.877521	1.005490
C	2.887662	4.321977	1.000912
C	1.571600	4.665251	0.890524
C	0.816403	3.433244	0.844737
C	-0.583739	3.365106	0.792686

C -1.324872 2.180588 0.861108
C -2.764500 2.128791 0.990318
C -3.102447 0.819074 1.149201
C -1.879785 0.048709 1.064166
C -1.805536 -1.351426 1.123621
C -0.619752 -2.090919 0.997693
C -0.555820 -3.535596 0.917570
C 0.754138 -3.869654 0.744976
C 1.508232 -2.637302 0.737729
C 2.901472 -2.567677 0.628357
H 5.755590 -2.177617 0.708030
H 6.427782 0.417754 1.029946
H 3.746298 4.974262 1.073468
H 1.137210 5.654024 0.856826
H -3.415215 2.990786 0.975401
H -4.088755 0.407997 1.302158
H -1.408227 -4.196088 0.974631
H 1.186493 -4.855234 0.645430
Fe 1.162792 0.388607 0.446685
C -1.336025 4.660559 0.742018
C -1.726210 5.281799 1.945199
C -1.632229 5.258438 -0.496347
C -2.419023 6.493053 1.888502
C -2.323802 6.474653 -0.507844
C -2.731488 7.103495 0.669901
H -2.716872 6.973709 2.818085
H -2.549291 6.939286 -1.465352
C 3.644409 -3.814209 0.262942
C 4.433024 -4.495562 1.209984
C 3.574620 -4.276058 -1.069589
C 5.192948 -5.593995 0.795021
C 4.356773 -5.373431 -1.442358
C 5.184526 -6.035157 -0.531057
H 5.801498 -6.119912 1.527721
C 5.425125 2.891180 1.177463
C 5.968341 3.147394 2.450656
C 6.090637 3.335053 0.019597
C 7.174979 3.846016 2.544809
C 7.295362 4.031101 0.156545
C 7.852611 4.298997 1.409554
H 7.594709 4.041235 3.529410
H 7.810543 4.371344 -0.739225
O 1.251924 0.382582 -1.226146
C 5.522530 3.062022 -1.352117
H 5.466424 1.987012 -1.557335
H 4.505968 3.459624 -1.447757
H 6.139950 3.519318 -2.130783
C 5.259779 2.672694 3.696582
H 4.242581 3.077083 3.753073
H 5.164656 1.580659 3.709666
H 5.800921 2.979464 4.596539
C 9.137377 5.081649 1.535343
H 9.708707 4.774000 2.417620
H 9.774037 4.949947 0.654175
H 8.938297 6.156929 1.635742
C 4.446829 -4.071287 2.660547
H 5.018794 -3.148877 2.813435
H 3.431940 -3.874261 3.022348
H 4.891637 -4.849683 3.287981
C 6.057748 -7.183493 -0.974975
H 5.559111 -7.796351 -1.733509
H 6.993975 -6.819497 -1.419629
H 6.326166 -7.831783 -0.134508
C -1.240794 4.601992 -1.798019
H -0.243473 4.153630 -1.738728
H -1.949069 3.807915 -2.061375
H -1.240331 5.331615 -2.614194
C -1.395950 4.654060 3.278421
H -1.861838 3.667063 3.381402
H -0.315785 4.506280 3.392490
H -1.743881 5.282801 4.103426
C -3.508190 8.397423 0.630304
H -4.590227 8.210685 0.649279
H -3.273867 9.032187 1.491773
H -3.292200 8.965638 -0.280454
H 4.322899 -5.712090 -2.475386
C 2.650614 -3.640700 -2.080428
H 1.621705 -3.994033 -1.937770
H 2.624741 -2.552991 -1.992747
H 2.957173 -3.893713 -3.097961
C -3.059763 -2.116363 1.386564
C -4.139424 -2.127072 0.499892
C -3.185468 -2.841805 2.581206
C -5.327906 -2.806782 0.788836
C -4.349434 -3.538097 2.911057
H -2.341507 -2.826568 3.259374
C -5.137461 -0.827216 -1.171020
C -6.418449 -2.792713 -0.281617
C -5.413528 -3.496177 1.996591
C -5.024440 0.388271 -1.858485
C -6.393082 -1.407050 -0.930891
H -6.329961 -4.023477 2.233367
C -6.197059 1.043235 -2.261896
C -7.528279 -0.721945 -1.355806
C -7.462050 0.514918 -2.018413
H -6.067772 1.992721 -2.764415
H -8.500581 -1.161781 -1.168249
C -4.500272 -4.329587 4.216970
C -4.780920 -5.811503 3.884826
C -3.233576 -4.263545 5.086155
C -5.678410 -3.753293 5.031906
H -5.697973 -5.929222 3.298416
H -3.955885 -6.242791 3.307010
H -4.895294 -6.394509 4.806437
H -2.997926 -3.235503 5.382231
H -3.384259 -4.846655 6.001150
H -2.363084 -4.678726 4.566307
H -5.804963 -4.311236 5.967381
H -5.499765 -2.701630 5.281498
H -6.621054 -3.810648 4.477882
O -4.003440 -1.434930 -0.685890
C -8.756252 1.219093 -2.445228
C -9.620811 1.497238 -1.196214
C -8.480405 2.557692 -3.149310
C -9.537658 0.310284 -3.418953
H -9.883685 0.573409 -0.670325
H -9.087232 2.144200 -0.491284
H -10.554137 1.997043 -1.481654
H -7.890547 2.421167 -4.062176
H -9.428879 3.026991 -3.432587
H -7.944132 3.255386 -2.497083
H -10.468515 0.797727 -3.732450
H -8.943115 0.098930 -4.314570
H -9.801211 -0.647337 -2.957742
C -3.738063 1.066604 -2.192905
O -3.662355 2.262437 -2.423854
C -7.797339 -3.145052 0.290002
H -8.109204 -2.434604 1.062624
H -8.552658 -3.153093 -0.501139
H -7.788682 -4.148759 0.724427
C -6.045772 -3.840126 -1.366076
H -6.017557 -4.843636 -0.926923
H -6.786622 -3.828266 -2.173290
H -5.064024 -3.625604 -1.797247
H 1.609391 0.397009 -4.568211
C 2.332639 0.560225 -3.770299
C 3.432783 -0.385483 -3.715827
C 2.103306 1.570241 -2.890859
C 3.654424 -1.272718 -4.790827
C 4.277804 -0.498811 -2.595775
H 1.246192 2.222967 -3.009020
H 2.829688 1.867649 -2.146520
C 4.663934 -2.221115 -4.752974
H 3.011637 -1.213318 -5.665591
C 5.297176 -1.441443 -2.547414
H 4.098196 0.118522 -1.724203
C 5.487251 -2.322519 -3.620642
H 4.825149 -2.910089 -5.575788
H 5.902527 -1.507941 -1.654811
O 6.419904 -3.312127 -3.643750
C 7.221564 -3.494474 -2.483997
H 7.838579 -2.609403 -2.282169
H 6.603665 -3.724124 -1.608902
H 7.870468 -4.344276 -2.703028
O -2.703904 0.224497 -2.270393
C -1.431765 0.815505 -2.586333
H -1.172311 1.570439 -1.845976
H -0.706744 0.008261 -2.538410
H -1.466481 1.270231 -3.581349

TS-4-3tri

B3LYP SCF energy: -3932.36303331 a.u.
B3LYP enthalpy: -3930.819245 a.u.
B3LYP free energy: -3931.039933 a.u.
B3LYP SCF energy in solution: -3930.26345671 a.u.
B3LYP enthalpy in solution: -3928.719668 a.u.
B3LYP free energy in solution: -3928.940356 a.u.
Imaginary frequency: -1524.9908 cm-1

Cartesian coordinates

ATOM X Y Z
N 2.891714 1.056595 -0.900961
N 1.996572 -1.543517 -1.461183
N -0.650360 -0.624590 -1.506431
N 0.258396 1.996518 -1.111421
C 3.133153 2.348435 -0.479322
C 4.542318 2.537627 -0.246786
C 5.160241 1.369747 -0.584304
C 4.126933 0.447458 -0.980557
C 4.372098 -0.864239 -1.377322
C 3.350646 -1.786414 -1.583378
C 3.580728 -3.180633 -1.865612
C 2.363226 -3.792064 -1.860563
C 1.380611 -2.767762 -1.614949
C 0.008801 -3.007140 -1.585645
C -0.926469 -1.979778 -1.536396
C -2.345865 -2.191301 -1.648205
C -2.925908 -0.962910 -1.733726
C -1.868957 0.011875 -1.628686
C -2.080616 1.389147 -1.616786
C -1.065679 2.302043 -1.359579
C -1.285711 3.717270 -1.210111
C -0.098311 4.265622 -0.831845
C 0.858980 3.190761 -0.764543
C 2.191179 3.370911 -0.404936
H 4.985350 3.460249 0.099489
H 6.214391 1.132885 -0.565782
H 4.556000 -3.618586 -2.021570
H 2.129537 -4.834749 -2.020260
H -2.816940 -3.162728 -1.678787
H -3.973458 -0.726051 -1.844321
H -2.237600 4.205427 -1.361302
H 0.130529 5.299008 -0.615226
Fe 1.092373 0.177932 -0.941845
C -0.477504 -4.417238 -1.709958
C -0.849758 -4.920428 -2.971309
C -0.549210 -5.234601 -0.566271
C -1.305524 -6.238463 -3.065597
C -1.013318 -6.545868 -0.704372
C -1.401823 -7.064385 -1.942469
H -1.588684 -6.629099 -4.040761
H -1.070359 -7.177472 0.179635
C 2.639148 4.733673 0.018096
C 3.260722 5.592786 -0.907914
C 2.429573 5.147292 1.347126
C 3.677855 6.856997 -0.481545
C 2.864599 6.418591 1.732699
C 3.489331 7.287970 0.834449
H 4.159547 7.521154 -1.196124
C 5.789323 -1.298523 -1.582873
C 6.407009 -1.054028 -2.824437
C 6.497729 -1.938147 -0.549801
C 7.729635 -1.462636 -3.014307
C 7.820345 -2.329340 -0.779415
C 8.452296 -2.105531 -2.005212
H 8.206606 -1.273301 -3.973657
H 8.369578 -2.819180 0.021875
O 0.885302 -0.054310 0.781575
C 5.847395 -2.187466 0.786451
H 5.456328 -1.261755 1.220949
H 4.994743 -2.870781 0.708295
H 6.557944 -2.620233 1.495557
C 5.655663 -0.357046 -3.933462
H 4.719880 -0.876843 -4.169002
H 5.383726 0.666228 -3.648274
H 6.259001 -0.307371 -4.844702
C 9.869595 -2.567810 -2.242874
H 10.393644 -1.908713 -2.943211
H 10.442220 -2.597976 -1.309973
H 9.890189 -3.578945 -2.670852
C 3.473027 5.163592 -2.340344
H 4.194890 4.341302 -2.410435
H 2.540473 4.803164 -2.789087
H 3.846536 5.994696 -2.945936
C 3.920592 8.668758 1.266581
H 4.768253 9.027531 0.673305
H 3.106246 9.395327 1.144062
H 4.212599 8.684362 2.322029
C -0.119028 -4.708183 0.779441
H 0.945185 -4.446118 0.773617
H -0.666817 -3.801556 1.058290
H -0.278133 -5.459308 1.559963
C -0.752380 -4.058901 -4.208011
H -1.455498 -3.218619 -4.167021
H 0.249180 -3.626974 -4.314765
H -0.972130 -4.641248 -5.107770
C -1.931853 -8.473053 -2.059792
H -1.743162 -8.890404 -3.054537

H	-1.472547	-9.136411	-1.319159
H	-3.017066	-8.503324	-1.893980
H	2.710644	6.735636	2.762013
C	1.744005	4.240982	2.337993
H	0.702095	4.052828	2.054431
H	2.227106	3.260647	2.401523
H	1.746790	4.678994	3.338144
C	-3.495792	1.858996	-1.718828
C	-4.322978	1.687518	-0.609131
C	-4.067560	2.349022	-2.897017
C	-5.702293	1.887815	-0.657479
C	-5.439864	2.615708	-2.984802
H	-3.421125	2.482674	-3.755961
C	-4.418598	0.311149	1.265021
C	-6.484906	1.595002	0.626091
C	-6.238696	2.354321	-1.857671
C	-3.715287	-0.720649	1.897846
C	-5.819465	0.384320	1.299272
H	-7.307668	2.521533	-1.923670
C	-4.451981	-1.703086	2.573547
C	-6.504504	-0.614635	1.986035
C	-5.843951	-1.673834	2.634530
H	-3.881440	-2.502404	3.028195
H	-7.587122	-0.575104	2.021258
C	-6.096777	3.150541	-4.265401
C	-6.785615	4.498754	-3.960373
C	-5.075622	3.378021	-5.392307
C	-7.149307	2.136963	-4.765085
H	-7.556767	4.394965	-3.190202
H	-6.056355	5.236150	-3.606969
H	-7.264709	4.896001	-4.863210
H	-4.577624	2.446657	-5.683452
H	-5.586385	3.771519	-6.277854
H	-4.306676	4.102477	-5.102083
H	-7.630281	2.504995	-5.679223
H	-6.682326	1.171372	-4.988473
H	-7.933431	1.965525	-4.020560
O	-3.721706	1.248506	0.544825
C	-6.666542	-2.743652	3.364974
C	-7.613506	-3.434958	2.359884
C	-5.775531	-3.820283	4.005948
C	-7.499928	-2.076821	4.480952
H	-8.303988	-2.722633	1.896261
H	-7.042715	-3.918264	1.559334
H	-8.213120	-4.201749	2.864906
H	-5.093463	-3.391933	4.748326
H	-6.401635	-4.560674	4.515955
H	-5.176394	-4.348733	3.256596
H	-8.097564	-2.827264	5.012185
H	-6.847901	-1.580610	5.208238
H	-8.187757	-1.324963	4.079877
C	-2.226536	-0.886344	1.831167
O	-1.692451	-1.967667	2.055453
O	-1.572856	0.227448	1.543040
H	-0.605219	0.043834	1.274533
C	-7.973841	1.357982	0.345955
H	-8.128169	0.500254	-0.316950
H	-8.519777	1.180380	1.277160
H	-8.422757	2.240749	-0.118986
C	-6.338044	2.815258	1.574142
H	-6.771686	3.708711	1.110869
H	-6.854990	2.622315	2.520739
H	-5.285471	3.016352	1.791847
H	1.284019	-0.379694	1.957367
C	1.296514	-0.708479	3.182471
C	2.343100	-1.738326	3.311518
C	1.509127	0.535472	3.948115
H	0.285590	-1.113166	3.255310
C	2.038064	-3.095444	3.126780
C	3.659385	-1.359290	3.639708
C	2.820953	0.927737	4.283979
C	3.020628	-4.068970	3.278684
H	1.018201	-3.368487	2.876607
C	4.628797	-2.345363	3.821587
C	3.981441	0.112583	3.758853
C	4.319266	-3.694510	3.637265
H	2.773042	-5.116823	3.134076
H	5.638918	-2.054081	4.099787
H	4.872393	0.264635	4.378782
H	4.242224	0.492062	2.754963
H	5.087880	-4.449865	3.775213
C	3.017264	2.060850	5.073933
H	4.029025	2.349853	5.348956
C	0.425170	1.324357	4.364514
H	-0.579093	1.035707	4.067502
C	0.633091	2.456987	5.145896
C	1.931797	2.819994	5.514303
H	-0.214223	3.053289	5.472362
H	2.099576	3.695533	6.135514

TS-4-5qui

B3LYP SCF energy: -3932.35886729 a.u.
B3LYP enthalpy: -3930.815404 a.u.
B3LYP free energy: -3931.039117 a.u.
B3LYP SCF energy in solution: -3930.26448536 a.u.
B3LYP enthalpy in solution: -3928.721022 a.u.
B3LYP free energy in solution: -3928.944735 a.u.
Imaginary frequency: -758.9729 cm-1

Cartesian coordinates

ATOM	X	Y	Z
N	2.952859	1.132604	-1.048869
N	1.973310	-1.510913	-1.603988
N	-0.721752	-0.511142	-1.595438
N	0.258675	2.139316	-1.107974
C	3.221954	2.434472	-0.693770
C	4.648312	2.614726	-0.550524
C	5.231833	1.414382	-0.835401
C	4.165356	0.486814	-1.135571
C	4.355867	-0.863699	-1.450610
C	3.321725	-1.781725	-1.666355
C	3.523184	-3.182818	-1.957445
C	2.287248	-3.752986	-2.045614
C	1.321177	-2.702102	-1.821934
C	-0.067490	-2.886413	-1.847851
C	-1.004866	-1.854239	-1.738321
C	-2.432908	-2.038663	-1.842258
C	-3.000917	-0.800915	-1.788827
C	-1.927136	0.153010	-1.621057
C	-2.106437	1.536948	-1.480426
C	-1.078502	2.447657	-1.224471
C	-1.278354	3.855804	-0.970024
C	-0.057356	4.384530	-0.673467
C	0.901950	3.306538	-0.759930
C	2.274898	3.453558	-0.533469
H	5.125881	3.545856	-0.280914
H	6.283205	1.164451	-0.842504
H	4.488940	-3.653888	-2.071877
H	2.036625	-4.784035	-2.249831
H	-2.921931	-2.995473	-1.953874
H	-4.049661	-0.550304	-1.848608
H	-2.235469	4.356280	-1.004166
H	0.188727	5.406010	-0.420946
Fe	1.083546	0.228782	-0.861023
C	-0.580890	-4.277201	-2.061756
C	-0.946435	-4.694407	-3.355817
C	-0.683162	-5.161543	-0.970960
C	-1.424388	-5.995241	-3.537219
C	-1.168197	-6.453174	-1.196498
C	-1.549158	-6.887308	-2.468861
H	-1.701828	-6.319333	-4.538091
H	-1.248605	-7.136969	-0.354098
C	2.769647	4.795242	-0.091524
C	3.159737	5.754982	-1.042996
C	2.837525	5.082629	1.284993
C	3.616861	6.999166	-0.599133
C	3.300853	6.339013	1.686346
C	3.697931	7.308641	0.761349
H	3.915411	7.743623	-1.334239
C	5.763667	-1.363368	-1.550808
C	6.461889	-1.238896	-2.766489
C	6.377461	-1.958616	-0.433764
C	7.773071	-1.715892	-2.844604
C	7.691498	-2.421038	-0.552846
C	8.406704	-2.306982	-1.747763
H	8.311180	-1.624855	-3.785810
H	8.166209	-2.882523	0.310535
O	0.916743	-0.032726	0.835633
C	5.630766	-2.102997	0.868385
H	5.217968	-1.144486	1.201411
H	4.781856	-2.790062	0.779897
H	6.284978	-2.484343	1.656472
C	5.803814	-0.605709	-3.968883
H	4.870842	-1.119169	-4.228457
H	5.543116	0.441316	-3.774386
H	6.465462	-0.636042	-4.839600
C	9.835525	-2.784718	-1.844672
H	10.540725	-1.978874	-1.601028
H	10.030011	-3.608094	-1.149422
H	10.074428	-3.129616	-2.856328
C	3.082832	5.447689	-2.519340
H	3.706476	4.584085	-2.778562
H	2.058956	5.198697	-2.821623
H	3.417291	6.301524	-3.116024
C	4.225628	8.646396	1.221067
H	5.310453	8.609014	1.387952
H	4.038119	9.427826	0.477118
H	3.763112	8.956110	2.164273
C	-0.270828	-4.728138	0.413670
H	0.793024	-4.466061	0.445040
H	-0.823295	-3.843190	0.746586
H	-0.443127	-5.530254	1.138663
C	-0.818341	-3.758947	-4.534619
H	-1.505841	-2.909315	-4.447687
H	0.192308	-3.340669	-4.603188
H	-1.038511	-4.279019	-5.471666
C	-2.101765	-8.276240	-2.679972
H	-3.191734	-8.293861	-2.546593
H	-1.892303	-8.641123	-3.691098
H	-1.676333	-8.989038	-1.965559
H	3.350522	6.564739	2.749513
C	2.413768	4.059532	2.309738
H	1.369992	3.756515	2.172550
H	3.009906	3.142527	2.240266
H	2.520253	4.449056	3.324672
C	-3.520569	2.023060	-1.501021
C	-4.330854	1.753581	-0.398679
C	-4.108283	2.624782	-2.618415
C	-5.709880	1.962014	-0.406511
C	-5.480087	2.903303	-2.659540
H	-3.474169	2.835167	-3.471092
C	-4.400620	0.230522	1.362245
C	-6.476182	1.558251	0.856468
C	-6.262634	2.539658	-1.549593
C	-3.688692	-0.840150	1.916244
C	-5.800935	0.298021	1.419257
H	-7.331635	2.715962	-1.582710
C	-4.414892	-1.873194	2.524319
C	-6.475644	-0.751853	2.036966
C	-5.806336	-1.854858	2.597678
H	-3.837387	-2.699642	2.917526
H	-7.557803	-0.717787	2.087818
C	-6.153819	3.557858	-3.874094
C	-6.831360	4.873790	-3.433172
C	-5.148739	3.886390	-4.990481
C	-7.218617	2.598991	-4.449861
H	-7.591307	4.700580	-2.664362
H	-6.093266	5.572058	-3.023182
H	-7.321984	5.355848	-4.287177
H	-4.659617	2.984716	-5.375038
H	-5.671055	4.362680	-5.827301
H	-4.372025	4.577984	-4.645900
H	-7.712092	3.052800	-5.317607
H	-6.759577	1.657265	-4.770410
H	-7.991859	2.360758	-3.712357
O	-3.711357	1.214816	0.702261
C	-6.618888	-2.979280	3.253408
C	-7.574096	-3.595642	2.208111
C	-5.718775	-4.098379	3.802301
C	-7.442834	-2.401348	4.424619
H	-8.271505	-2.852783	1.806967
H	-7.010205	-4.014557	1.367411
H	-8.166133	-4.400783	2.659488
H	-5.031296	-3.725525	4.569246
H	-6.337997	-4.877771	4.259841
H	-5.125014	-4.565915	3.009433
H	-8.033956	-3.191574	4.902760
H	-6.784955	-1.960059	5.181420
H	-8.135856	-1.623082	4.088143
C	-2.200657	-0.985078	1.837531
O	-1.648702	-2.070227	1.981639
O	-1.558923	0.157435	1.633807
H	-0.596645	-0.001215	1.362330
C	-7.967854	1.340580	0.574401
H	-8.127673	0.541385	-0.156801
H	-8.502593	1.083396	1.493419
H	-8.424515	2.257756	0.190935
C	-6.321178	2.694548	1.902637
H	-6.763023	3.622228	1.521737
H	-6.825481	2.420513	2.835978
H	-5.266466	2.880880	2.123463
H	1.293236	-0.452232	2.127090
C	1.289822	-0.804420	3.277398
C	2.221880	-1.948913	3.364517
C	1.624006	0.391107	4.083923
H	0.246818	-1.115020	3.375579
C	1.787562	-3.234967	3.004626
C	3.547875	-1.752813	3.791707

C	2.948536	0.608213	4.508156
C	2.653745	-4.319914	3.081746
H	0.762684	-3.359352	2.668703
C	4.398993	-2.855141	3.895234
C	4.036885	-0.353723	4.087649
C	3.963083	-4.131607	3.538272
H	2.309463	-5.310594	2.798851
H	5.417088	-2.709686	4.249190
H	4.833705	-0.379722	4.840857
H	4.510460	0.043525	3.173072
H	4.641471	-4.976605	3.616691
C	3.241334	1.730917	5.285939
H	4.261538	1.889792	5.628020
C	0.628679	1.325419	4.413781
H	-0.383315	1.161413	4.052816
C	0.933388	2.444496	5.180713
C	2.244089	2.644647	5.626772
H	0.154688	3.158136	5.434245
H	2.487979	3.510508	6.236211

TS-5-3tri

B3LYP SCF energy: -3912.49987253 a.u.
B3LYP enthalpy: -3910.942264 a.u.
B3LYP free energy: -3911.160513 a.u.
B3LYP SCF energy in solution: -3910.41375842 a.u.
B3LYP enthalpy in solution: -3908.856150 a.u.
B3LYP free energy in solution: -3909.074399 a.u.
Imaginary frequency: -1620.3189 cm-1

Cartesian coordinates

ATOM	X	Y	Z
N	2.787649	1.518226	-0.847955
N	2.320101	-1.191081	-1.314470
N	-0.389848	-0.652941	-1.636967
N	0.052364	2.020210	-0.977627
C	2.822289	2.820642	-0.405276
C	4.179928	3.305833	-0.410180
C	4.960140	2.297297	-0.894357
C	4.095666	1.161469	-1.106590
C	4.549987	-0.132397	-1.358724
C	3.695600	-1.235986	-1.318647
C	4.141547	-2.603246	-1.218142
C	3.025300	-3.378740	-1.112728
C	1.888197	-2.500026	-1.231588
C	0.567408	-2.918382	-1.391273
C	-0.462947	-2.026789	-1.696568
C	-1.777947	-2.431391	-2.122774
C	-2.502988	-1.293215	-2.314769
C	-1.644230	-0.186492	-1.970683
C	-2.082208	1.131460	-1.839295
C	-1.285657	2.136675	-1.295820
C	-1.770385	3.436507	-0.905058
C	-0.729631	4.089399	-0.312932
C	0.414885	3.217456	-0.395410
C	1.714821	3.600319	-0.073490
H	4.473372	4.301868	-0.110498
H	6.027380	2.293494	-1.063380
H	5.177538	-2.907821	-1.186607
H	2.962491	-4.448027	-0.981848
H	-2.086270	-3.457747	-2.260575
H	-3.530856	-1.198733	-2.632766
H	-2.782968	3.782943	-1.054941
H	-0.711241	5.082992	0.111244
Fe	1.158947	0.366752	-0.895139
C	0.233712	-4.367759	-1.256036
C	0.631900	-5.297578	-2.238850
C	-0.501234	-4.794644	-0.129504
C	0.346353	-6.651266	-2.043487
C	-0.753051	-6.161153	0.030008
C	-0.326337	-7.106029	-0.905490
H	0.648876	-7.367524	-2.804638
H	-1.305973	-6.491702	0.906880
C	1.936688	4.913101	0.603746
C	2.032708	6.103867	-0.139002
C	2.052821	4.930418	2.007407
C	2.236490	7.307946	0.541760
C	2.251453	6.156545	2.648190
C	2.341737	7.355177	1.934622
H	2.316070	8.229842	-0.030488
C	6.000457	-0.357999	-1.650244
C	6.393645	-0.559009	-2.989261
C	6.962147	-0.368208	-0.623009
C	7.745874	-0.758120	-3.276830
C	8.305277	-0.574840	-0.952888
C	8.717844	-0.772311	-2.272332
H	8.047395	-0.904375	-4.311924
H	9.045795	-0.584809	-0.155944
O	0.957022	0.039871	0.795726
C	6.555230	-0.174258	0.815551
H	6.248894	0.858861	1.016232
H	5.705035	-0.812608	1.065771
H	7.380066	-0.418626	1.492203
C	5.373627	-0.555506	-4.103254
H	4.690280	-1.408929	-4.020329
H	4.753180	0.347401	-4.075516
H	5.862847	-0.606452	-5.080480
C	10.168872	-1.023906	-2.603673
H	10.439252	-0.589167	-3.572014
H	10.832324	-0.599943	-1.842863
H	10.381677	-2.099729	-2.660475
C	1.928106	6.083832	-1.645568
H	2.663925	5.401766	-2.086934
H	0.941338	5.736706	-1.973708
H	2.093274	7.081587	-2.062884
C	2.524271	8.670347	2.653185
H	1.557933	9.090668	2.962254
H	3.129461	8.551130	3.558264
H	3.012175	9.412311	2.012447
C	-1.063461	-3.813368	0.872268
H	-0.457338	-2.912476	0.968637
H	-2.062382	-3.474447	0.572125
H	-1.166775	-4.265482	1.862997
C	1.316313	-4.852245	-3.511327
H	0.852345	-3.945057	-3.912874
H	2.375016	-4.619320	-3.351202
H	1.257532	-5.635159	-4.273635
C	-0.578470	-8.578781	-0.691787
H	0.255788	-9.049249	-0.154414
H	-1.483160	-8.748080	-0.098516
H	-0.690633	-9.108283	-1.643959
H	2.339517	6.173688	3.732463
C	1.985708	3.650037	2.803963
H	1.112130	3.043527	2.543358
H	2.860141	3.016223	2.614849
H	1.950833	3.851090	3.877194
C	-3.525594	1.398599	-2.119143
C	-4.450680	1.329923	-1.078231
C	-4.009443	1.596183	-3.417038
C	-5.828509	1.402080	-1.288070
C	-5.379936	1.691850	-3.681799
H	-3.284869	1.642963	-4.220772
C	-4.688389	0.325925	1.036109
C	-6.727784	1.316899	-0.052992
C	-6.268764	1.579157	-2.599248
C	-4.008194	-0.478711	1.956932
C	-6.084653	0.319510	0.919916
H	-7.334188	1.633008	-2.789492
C	-4.778404	-1.322469	2.769098
C	-6.799490	-0.544210	1.745987
C	-6.167888	-1.380530	2.682025
H	-4.222385	-1.945268	3.457245
H	-7.879938	-0.568212	1.666498
C	-5.936772	1.895535	-5.097724
C	-6.757974	3.202720	-5.140561
C	-4.822302	1.992936	-6.152608
C	-6.847181	0.703552	-5.465532
H	-7.597001	3.177928	-4.437663
H	-6.131005	4.063824	-4.884135
H	-7.167066	3.363577	-6.145051
H	-4.221284	1.077883	-6.193960
H	-5.265391	2.143691	-7.142929
H	-4.151119	2.836357	-5.956713
H	-7.256910	0.833413	-6.474235
H	-6.284216	-0.236136	-5.443065
H	-7.689145	0.606335	-4.772580
O	-3.956693	1.148656	0.197041
C	-7.016021	-2.310841	3.559338
C	-7.817363	-3.274148	2.656774
C	-6.152183	-3.151010	4.514168
C	-7.993891	-1.466903	4.405659
H	-8.485822	-2.734056	1.978018
H	-7.142844	-3.885355	2.047068
H	-8.432237	-3.946548	3.266947
H	-5.575653	-2.520607	5.199498
H	-6.796068	-3.799757	5.118231
H	-5.450398	-3.790509	3.968221
H	-8.610373	-2.116553	5.038444
H	-7.446807	-0.775005	5.055451
H	-8.668183	-0.874991	3.777699
C	-2.506039	-0.564385	2.134529
O	-2.032209	-1.478716	2.825186
C	-8.166295	0.928481	-0.416719
H	-8.209810	-0.053938	-0.898083
H	-8.798038	0.906570	0.475756
H	-8.604971	1.667233	-1.093872
C	-6.742337	2.711964	0.628835
H	-7.165508	3.461102	-0.049832
H	-7.347889	2.679395	1.541179
H	-5.730480	3.025678	0.900703
N	-1.748507	0.388985	1.558540
H	-2.175480	1.034726	0.912433
H	-0.737001	0.250307	1.458494
H	1.204392	-0.773132	1.751891
C	1.308724	-1.435871	2.831303
C	2.455319	-2.339831	2.612280
C	1.500204	-0.392218	3.859400
H	0.331231	-1.917278	2.873433
C	2.285200	-3.675929	2.219075
C	3.758372	-1.844784	2.829127
C	2.795213	0.116801	4.076081
C	3.383786	-4.518125	2.066094
H	1.284489	-4.057077	2.050982
C	4.849938	-2.702252	2.690675
C	3.913383	-0.384890	3.191572
C	4.670380	-4.033924	2.307685
H	3.232877	-5.551947	1.767574
H	5.850426	-2.328487	2.891960
H	4.891528	-0.196881	3.647916
H	3.888900	0.200939	2.256728
H	5.531476	-4.688486	2.205014
C	2.996466	1.089772	5.053499
H	3.999356	1.472723	5.229129
C	0.420241	0.111002	4.599940
H	-0.572525	-0.285448	4.407590
C	0.631326	1.093596	5.564496
C	1.920931	1.579102	5.799262
H	-0.207472	1.476060	6.139634
H	2.090109	2.336371	6.560212

TS-5-5qui

B3LYP SCF energy: -3912.49435394 a.u.
B3LYP enthalpy: -3910.937231 a.u.
B3LYP free energy: -3911.160114 a.u.
B3LYP SCF energy in solution: -3910.41506540 a.u.
B3LYP enthalpy in solution: -3908.857942 a.u.
B3LYP free energy in solution: -3909.080825 a.u.
Imaginary frequency: -863.5018 cm-1

Cartesian coordinates

ATOM	X	Y	Z
N	3.124845	0.917614	-0.861243
N	2.014723	-1.673034	-1.417182
N	-0.624317	-0.531435	-1.496277
N	0.486967	2.072918	-0.997141
C	3.457411	2.209603	-0.524192
C	4.889653	2.315346	-0.361268
C	5.412914	1.083328	-0.627814
C	4.303114	0.209380	-0.933661
C	4.428393	-1.146398	-1.260858
C	3.348440	-2.010770	-1.475784
C	3.479926	-3.419513	-1.773536
C	2.217877	-3.927245	-1.865245
C	1.304548	-2.828149	-1.649343
C	-0.090076	-2.938458	-1.720159
C	-0.972939	-1.855546	-1.654388
C	-2.403831	-1.963089	-1.823494
C	-2.906373	-0.697667	-1.786017
C	-1.791099	0.196118	-1.560730
C	-1.903134	1.583968	-1.391678
C	-0.832063	2.445420	-1.119377
C	-0.961127	3.864643	-0.870200
C	0.286422	4.333789	-0.583209
C	1.189409	3.207113	-0.656039
C	2.565290	3.283512	-0.410305
H	5.412686	3.222515	-0.094339
H	6.450065	0.779371	-0.621007
H	4.420959	-3.937721	-1.889928
H	1.916061	-4.943530	-2.073676
H	-2.937550	-2.891744	-1.963995
H	-3.936640	-0.391942	-1.892817
H	-1.889425	4.416093	-0.907873
H	0.583409	5.344391	-0.341838
Fe	1.201695	0.121875	-0.716267
C	-0.671336	-4.300042	-1.947607
C	-0.986621	-4.712308	-3.256600
C	-0.898943	-5.156082	-0.853308
C	-1.538142	-5.981380	-3.451695
C	-1.453234	-6.417325	-1.094242

C	-1.779034	-6.848515	-2.382448
H	-1.783047	-6.299662	-4.462911
H	-1.635692	-7.078003	-0.249380
C	3.127433	4.611663	-0.008366
C	3.647087	5.483070	-0.983042
C	3.126439	4.977972	1.350622
C	4.167042	6.716018	-0.578756
C	3.656329	6.219893	1.712729
C	4.184836	7.100479	0.764831
H	4.565265	7.392318	-1.332269
C	5.810654	-1.700686	-1.427135
C	6.477001	-1.523413	-2.655348
C	6.432367	-2.402632	-0.378505
C	7.760660	-2.052979	-2.812049
C	7.717576	-2.916326	-0.575269
C	8.399914	-2.749770	-1.782974
H	8.271390	-1.921098	-3.763594
H	8.195894	-3.460450	0.236408
O	0.956980	-0.100358	0.960899
C	5.726589	-2.596702	0.939622
H	5.448423	-1.634724	1.382045
H	4.797892	-3.166133	0.827963
H	6.365958	-3.127135	1.651719
C	5.815718	-0.779861	-3.791194
H	4.834104	-1.205660	-4.028142
H	5.648508	0.272887	-3.534564
H	6.432713	-0.815855	-4.693894
C	9.799559	-3.285654	-1.963562
H	9.988407	-3.568412	-3.004628
H	10.550876	-2.532930	-1.689773
H	9.975737	-4.164568	-1.334610
C	3.639950	5.095853	-2.442508
H	4.252531	4.204193	-2.620908
H	2.627041	4.855453	-2.785463
H	4.029351	5.906580	-3.065436
C	4.781989	8.422137	1.184256
H	4.696597	9.170381	0.389187
H	4.289331	8.817899	2.078750
H	5.849485	8.318107	1.420274
C	-0.565159	-4.724623	0.552348
H	0.471669	-4.382832	0.634331
H	-1.200879	-3.899408	0.893716
H	-0.701947	-5.555154	1.252068
C	-0.734940	-3.801837	-4.435126
H	-1.358837	-2.901513	-4.386656
H	0.306821	-3.462113	-4.460445
H	-0.950940	-4.313955	-5.377530
C	-2.346628	-8.227752	-2.616952
H	-2.983570	-8.252427	-3.507562
H	-1.547466	-8.965866	-2.767691
H	-2.942700	-8.565111	-1.762393
H	3.654201	6.505275	2.762632
C	2.557310	4.054038	2.398334
H	1.496562	3.846783	2.217539
H	3.064614	3.083080	2.406192
H	2.651722	4.484843	3.397391
C	-3.277395	2.163975	-1.469346
C	-4.219279	1.891025	-0.476355
C	-3.683983	2.937685	-2.563600
C	-5.550168	2.301573	-0.570216
C	-4.998781	3.394299	-2.694774
H	-2.945216	3.148228	-3.327048
C	-4.694072	0.266027	1.164263
C	-6.478022	1.946225	0.592637
C	-5.916978	3.046979	-1.690172
C	-4.209866	-0.920657	1.729396
C	-6.062372	0.565112	1.114734
H	-6.945109	3.375569	-1.784732
C	-5.150708	-1.830939	2.229879
C	-6.953072	-0.380465	1.617227
C	-6.522500	-1.594116	2.178147
H	-4.743547	-2.743302	2.645073
H	-8.015009	-0.168693	1.579352
C	-5.467192	4.235912	-3.889831
C	-6.008622	5.589014	-3.378889
C	-4.328530	4.518290	-4.883773
C	-6.588160	3.482753	-4.638660
H	-6.853297	5.456049	-2.695154
H	-5.228655	6.142776	-2.844574
H	-6.351358	6.204576	-4.219030
H	-3.922777	3.594114	-5.309619
H	-4.705977	5.127484	-5.712136
H	-3.506797	5.069324	-4.413161
H	-6.937769	4.072018	-5.494696
H	-6.225793	2.518459	-5.011544
H	-7.449861	3.289420	-3.991545
O	-3.796368	1.173389	0.626407
C	-7.558428	-2.594092	2.708256
C	-8.498484	-3.013103	1.556965
C	-6.899264	-3.860660	3.278394
C	-8.385157	-1.929799	3.830732
H	-9.029847	-2.154482	1.133033
H	-7.933015	-3.490016	0.748858
H	-9.249210	-3.726431	1.917551
H	-6.236721	-3.629002	4.119163
H	-7.672793	-4.546340	3.641433
H	-6.313391	-4.388881	2.518646
H	-9.133066	-2.630821	4.220114
H	-7.737937	-1.623909	4.660070
H	-8.915266	-1.040641	3.473344
C	-2.760007	-1.339929	1.836788
O	-2.479656	-2.485095	2.211478
C	-7.955319	1.990846	0.181246
H	-8.173801	1.275055	-0.617899
H	-8.601188	1.767154	1.034951
H	-8.228981	2.991870	-0.164317
C	-6.243747	2.978515	1.728872
H	-6.500683	3.986259	1.383615
H	-6.866132	2.732379	2.596205
H	-5.197356	2.980327	2.046840
N	-1.819335	-0.412989	1.559208
H	-2.087225	0.458562	1.127961
H	-0.851212	-0.693572	1.420744
H	1.042400	-0.141867	2.329297
C	1.021089	-0.439884	3.508594
C	1.618539	-1.795924	3.517972
C	1.790701	0.603853	4.223896
H	-0.048146	-0.429856	3.732962
C	0.822472	-2.947703	3.438523
C	3.021620	-1.910020	3.556500
C	3.195713	0.501210	4.261276
C	1.418372	-4.206872	3.441144
H	-0.257114	-2.849111	3.352178
C	3.604046	-3.176400	3.575475
C	3.846067	-0.642446	3.519443
C	2.808948	-4.323661	3.519026
H	0.798250	-5.096235	3.383495
H	4.685722	-3.266803	3.629950
H	4.860442	-0.822815	3.891738
H	3.952379	-0.341202	2.462945
H	3.274771	-5.305169	3.532917
C	3.936295	1.456806	4.956073
H	5.019515	1.369034	4.993174
C	1.159920	1.679001	4.865403
H	0.076147	1.753990	4.828810
C	1.907466	2.628029	5.558165
C	3.299585	2.515975	5.606612
H	1.407692	3.450018	6.062840
H	3.888042	3.250166	6.149709

TS-6-3tri

B3LYP SCF energy: -3971.66707674 a.u.
B3LYP enthalpy: -3970.092022 a.u.
B3LYP free energy: -3970.311727 a.u.
B3LYP SCF energy in solution: -3969.52601555 a.u.
B3LYP enthalpy in solution: -3967.950961 a.u.
B3LYP free energy in solution: -3968.170666 a.u.
Imaginary frequency: -1835.2264 cm-1

Cartesian coordinates

ATOM	X	Y	Z
N	2.994766	0.956856	-0.779527
N	1.738514	-1.402626	-1.567590
N	-0.733480	-0.114311	-1.449067
N	0.508377	2.238084	-0.610719
C	3.433074	2.123013	-0.185329
C	4.875160	2.153434	-0.165075
C	5.307350	1.022593	-0.792842
C	4.133372	0.263697	-1.145835
C	4.173409	-1.015725	-1.698239
C	3.031887	-1.804177	-1.834150
C	3.054961	-3.210710	-2.153484
C	1.780331	-3.671859	-2.000521
C	0.957639	-2.534265	-1.670534
C	-0.434562	-2.564282	-1.585397
C	-1.203414	-1.403740	-1.577428
C	-2.628866	-1.391458	-1.787525
C	-3.016090	-0.086953	-1.807153
C	-1.837006	0.706266	-1.538104
C	-1.859974	2.076586	-1.264238
C	-0.758777	2.761785	-0.767193
C	-0.804147	4.112522	-0.266866
C	0.436445	4.396390	0.220003
C	1.256936	3.230711	-0.013085
C	2.626315	3.180802	0.238217
H	5.463994	2.953794	0.258934
H	6.321818	0.702433	-0.983154
H	3.945145	-3.763833	-2.417057
H	1.412315	-4.681364	-2.112089
H	-3.234254	-2.275690	-1.925520
H	-4.009566	0.306786	-1.957614
H	-1.685195	4.738583	-0.281682
H	0.790346	5.311092	0.673793
Fe	1.098508	0.355641	-0.882170
C	-1.140624	-3.878984	-1.507918
C	-1.332662	-4.680205	-2.649117
C	-1.639239	-4.286375	-0.252565
C	-1.976080	-5.913467	-2.506376
C	-2.270054	-5.530118	-0.155832
C	-2.437048	-6.362739	-1.265633
H	-2.129597	-6.532441	-3.387944
H	-2.644529	-5.851496	0.814053
C	3.283515	4.361293	0.883856
C	3.915108	5.336393	0.083859
C	3.267533	4.495663	2.283682
C	4.549398	6.414313	0.704882
C	3.920971	5.587800	2.865839
C	4.573878	6.551964	2.096696
H	5.033946	7.167143	0.086542
C	5.490978	-1.565420	-2.152275
C	5.908448	-1.293563	-3.470658
C	6.300514	-2.339588	-1.300022
C	7.131544	-1.800885	-3.915289
C	7.518191	-2.828670	-1.784652
C	7.953514	-2.568683	-3.085904
H	7.448399	-1.592417	-4.935009
H	8.141155	-3.429221	-1.125420
O	0.882637	-0.155597	0.722140
C	5.877144	-2.651613	0.114511
H	5.709604	-1.739484	0.696674
H	4.941312	-3.220573	0.139888
H	6.640831	-3.241515	0.629510
C	5.045748	-0.467520	-4.394820
H	4.051569	-0.912850	-4.516859
H	4.890947	0.542731	-3.998065
H	5.504905	-0.378909	-5.383808
C	9.286404	-3.080467	-3.575892
H	10.075153	-2.329381	-3.435749
H	9.593374	-3.980416	-3.033064
H	9.254503	-3.320994	-4.643980
C	3.897648	5.232680	-1.423006
H	4.485884	4.377557	-1.775654
H	2.878003	5.090171	-1.798284
H	4.308437	6.137789	-1.880196
C	5.302296	7.703832	2.745859
H	5.211081	8.620409	2.152782
H	4.914583	7.907675	3.749242
H	6.374490	7.488874	2.846561
C	-1.559868	-3.381454	0.951226
H	-1.718823	-3.940529	1.876041
H	-0.599959	-2.868340	1.032274
H	-2.324848	-2.599471	0.895551
C	-0.897014	-4.205727	-4.016396
H	-1.238958	-3.181604	-4.204138
H	0.193688	-4.195727	-4.121801
H	-1.304282	-4.852174	-4.799659
C	-3.090803	-7.716224	-1.125659
H	-2.353892	-8.487650	-0.864776
H	-3.849953	-7.713809	-0.336087
H	-3.571004	-8.027985	-2.059268
H	3.913566	5.685723	3.949464
C	2.518493	3.519075	3.156150
H	1.463853	3.811313	3.233306
H	2.547953	2.501953	2.760236
H	2.929974	3.497528	4.168572
C	-3.206155	2.717540	-1.381264
C	-4.148575	2.443156	-0.390022
C	-3.649271	3.318014	-2.563763
C	-5.519809	2.552905	-0.617290
O	-3.671589	1.931975	0.795968
C	-5.008628	3.568133	-2.790065
H	-2.913488	3.526224	-3.331208
C	-6.441837	1.962301	0.451552
C	-5.927202	3.131839	-1.819826
C	-4.353773	0.855316	1.300782
C	-5.525030	4.231090	-4.074622
C	-5.725581	0.729719	1.028927
C	-7.824286	1.618041	-0.114848

C -6.609684 2.997087 1.595672
H -6.987899 3.237612 -2.016622
C -3.660843 -0.110076 2.040350
C -6.318462 5.503298 -3.704553
C -4.381905 4.635751 -5.020090
C -6.449381 3.248924 -4.826864
C -6.346468 -0.470298 1.367045
H -7.756933 0.892205 -0.931941
H -8.468402 1.204115 0.666707
H -8.320833 2.517226 -0.491356
H -7.092922 3.904088 1.215141
H -7.227198 2.578757 2.398181
H -5.639856 3.275618 2.017062
C -4.335611 -1.292983 2.373432
C -2.261365 0.035485 2.543551
H -7.172709 5.273907 -3.059322
H -5.680398 6.218538 -3.173805
H -6.701764 5.991048 -4.608764
H -3.809142 3.766388 -5.361141
H -4.794894 5.128238 -5.907111
H -3.689641 5.336721 -4.540835
H -6.825689 3.708777 -5.748425
H -5.907380 2.335647 -5.096121
H -7.314237 2.959352 -4.221248
C -5.659003 -1.520107 2.004220
H -7.395415 -0.601393 1.126898
H -3.764615 -2.030751 2.920744
O -1.648004 -0.881003 3.066165
O -1.797123 1.282895 2.436974
C -6.367356 -2.856504 2.258075
C -0.471507 1.530108 2.935745
C -6.778337 -3.463548 0.898034
C -5.454445 -3.866224 2.974032
C -7.623468 -2.631717 3.126039
H 0.252463 0.944207 2.368236
H -0.313263 2.594441 2.769416
H -0.414302 1.292481 4.002145
H -7.453041 -2.800410 0.346293
H -5.896103 -3.642143 0.273134
H -7.293452 -4.420391 1.045794
H -5.139233 -3.502807 3.958214
H -5.994392 -4.807706 3.123547
H -4.556292 -4.085143 2.386111
H -8.138835 -3.582676 3.305960
H -7.353055 -2.200077 4.096084
H -8.335855 -1.953883 2.644011
H 1.754065 -0.929068 1.206455
C 2.655445 -1.639275 1.795678
C 3.147318 -0.764735 2.876391
C 1.982829 -2.880891 2.217925
H 3.353838 -1.764599 0.969311
C 4.243567 0.089856 2.683039
C 2.490727 -0.781259 4.124224
C 1.315747 -2.913669 3.459329
C 4.707768 0.896961 3.717130
H 4.726649 0.119831 1.712245
C 2.966159 0.033131 5.154004
C 1.276551 -1.662480 4.303906
C 4.070532 0.865414 4.960552
H 5.557416 1.553586 3.551984
H 2.468580 0.009659 6.120968
H 1.143627 -1.918269 5.361851
H 0.375872 -1.103544 4.007106
H 4.429984 1.487571 5.775738
C 0.669707 -4.084665 3.856759
H 0.158749 -4.107959 4.816387
C 1.977782 -4.023700 1.402772
H 2.487859 -3.990320 0.446686
C 1.318559 -5.181598 1.802665
C 0.663229 -5.214347 3.036574
H 1.312000 -6.053600 1.155032
H 0.148853 -6.115642 3.358865

TS-6-5qui

B3LYP SCF energy: -3971.65510932 a.u.
B3LYP enthalpy: -3970.080408 a.u.
B3LYP free energy: -3970.307915 a.u.
B3LYP SCF energy in solution: -3969.52338022 a.u.
B3LYP enthalpy in solution: -3967.948679 a.u.
B3LYP free energy in solution: -3968.176186 a.u.
Imaginary frequency: -955.7845 cm-1

Cartesian coordinates

ATOM X Y Z
N 2.810523 1.549715 -0.677964
N 2.605209 -1.210299 -1.458121
N -0.224287 -0.847226 -1.965764
N -0.018887 1.908534 -1.130106
C 2.718640 2.883817 -0.349151
C 4.032592 3.401888 -0.042682
C 4.915476 2.374471 -0.209806
C 4.147282 1.217163 -0.608706
C 4.705038 -0.039156 -0.884042
C 3.962817 -1.172213 -1.241516
C 4.511019 -2.504521 -1.368911
C 3.461425 -3.344945 -1.605733
C 2.271386 -2.525102 -1.674822
C 0.980157 -3.010763 -1.933211
C -0.158186 -2.215488 -2.105000
C -1.471401 -2.731209 -2.419441
C -2.323995 -1.668556 -2.441828
C -1.542467 -0.491096 -2.122152
C -2.079649 0.786935 -1.905381
C -1.361373 1.891299 -1.429430
C -1.924869 3.202396 -1.200375
C -0.912850 4.001896 -0.759596
C 0.282481 3.188476 -0.718377
C 1.549479 3.659939 -0.357305
H 4.239522 4.421952 0.247266
H 5.988650 2.385820 -0.083054
H 5.557903 -2.751963 -1.263725
H 3.479016 -4.417804 -1.732713
H -1.695618 -3.774069 -2.592669
H -3.387166 -1.672491 -2.630767
H -2.960249 3.464944 -1.361640
H -0.953711 5.047704 -0.490448
Fe 1.187132 0.232809 -0.903670
C 0.796862 -4.496007 -1.926208
C 1.117916 -5.270844 -3.055443
C 0.328124 -5.106265 -0.744665
C 0.985673 -6.660167 -2.980595
C 0.220618 -6.500042 -0.711117
C 0.543621 -7.293278 -1.815167
H 1.235570 -7.261305 -3.852406
H -0.125237 -6.975639 0.204295
C 1.692599 5.124665 -0.065464
C 1.971882 6.001941 -1.135499
C 1.571139 5.623206 1.242271
C 2.123404 7.364131 -0.874037
C 1.735535 6.996496 1.462320
C 2.007475 7.882808 0.420490
H 2.342812 8.037283 -1.700261
C 6.200333 -0.162325 -0.863898
C 6.905178 0.094594 -2.057788
C 6.897192 -0.528686 0.302217
C 8.297425 -0.017219 -2.063115
C 8.291729 -0.629288 0.253708
C 9.010751 -0.375062 -0.915842
H 8.837201 0.179493 -2.987031
H 8.827554 -0.915299 1.156144
O 0.765243 -0.399992 0.609062
C 6.170627 -0.825837 1.590557
H 5.530007 0.007289 1.896417
H 5.525496 -1.706310 1.494258
H 6.877715 -1.020264 2.402135
C 6.170043 0.485556 -3.317684
H 5.447208 -0.283394 -3.613602
H 5.602448 1.412693 -3.176263
H 6.868123 0.635849 -4.146425
C 10.517806 -0.458277 -0.935386
H 10.886447 -0.811719 -1.904366
H 10.971436 0.525334 -0.754795
H 10.890938 -1.136406 -0.161003
C 2.113434 5.477383 -2.544389
H 2.926302 4.745533 -2.617223
H 1.200334 4.968320 -2.873793
H 2.322908 6.291161 -3.244849
C 2.158221 9.363379 0.673353
H 1.267408 9.914763 0.345125
H 2.302179 9.574096 1.737752
H 3.012691 9.777984 0.126435
C -0.083082 -4.284786 0.454569
H -1.091151 -3.873763 0.314552
H -0.086896 -4.891798 1.363531
H 0.578310 -3.432319 0.622420
C 1.597421 -4.615001 -4.328935
H 0.918889 -3.813077 -4.641319
H 2.585723 -4.157867 -4.200124
H 1.665735 -5.343303 -5.142605
C 0.388006 -8.794108 -1.761317
H 1.088360 -9.294200 -2.438652
H 0.559767 -9.177346 -0.749835
H -0.624837 -9.099189 -2.056565
H 1.648560 7.378267 2.477425
C 1.243820 4.716038 2.401997
H 0.159382 4.581272 2.499520
H 1.680607 3.721839 2.286448
H 1.603289 5.138938 3.345465
C -3.539455 0.942527 -2.172798
C -4.463557 1.032679 -1.128846
C -4.022326 0.898473 -3.486998
C -5.842225 1.044718 -1.368087
O -3.965286 1.079310 0.154860
C -5.388986 0.916429 -3.774171
H -3.290781 0.823124 -4.282135
C -6.764933 1.176541 -0.158702
C -6.278429 0.981383 -2.690399
C -4.726162 0.502581 1.143919
C -5.938014 0.855863 -5.205705
C -6.118558 0.407305 0.995905
C -8.189525 0.693736 -0.459486
C -6.820611 2.676284 0.241897
H -7.343760 0.985313 -2.887695
C -4.084875 -0.012659 2.277615
C -6.778765 2.120692 -5.484958
C -4.816644 0.780595 -6.255013
C -6.827632 -0.397130 -5.358791
C -6.835655 -0.298704 1.958921
H -8.205908 -0.362001 -0.749281
H -8.834258 0.825245 0.414160
H -8.633931 1.282590 -1.266938
H -7.245315 3.270275 -0.575264
H -7.441909 2.806961 1.134884
H -5.819872 3.059378 0.462091
C -4.848393 -0.721611 3.215290
C -2.638786 0.174810 2.607926
H -7.621416 2.208144 -4.791354
H -6.166580 3.023722 -5.384395
H -7.184281 2.092933 -6.503378
H -4.199951 -0.115661 -6.125749
H -5.253591 0.741364 -7.258734
H -4.161198 1.657351 -6.211608
H -7.232450 -0.456116 -6.376143
H -6.250538 -1.308484 -5.166640
H -7.672658 -0.382893 -4.662817
C -6.223065 -0.895475 3.072615
H -7.909517 -0.387001 1.844938
H -4.310879 -1.131149 4.060448
O -2.026000 -0.577610 3.346544
O -2.136418 1.300840 2.095750
C -7.073842 -1.679501 4.079814
C -0.770421 1.602426 2.446092
C -7.773402 -2.848816 3.353099
C -6.226217 -2.260278 5.223472
C -8.139364 -0.744296 4.691910
H -0.107140 0.831223 2.057149
H -0.565942 2.551401 1.955994
H -0.664953 1.689338 3.530608
H -8.423766 -2.494257 2.546576
H -7.035739 -3.529426 2.914079
H -8.392104 -3.419821 4.055722
H -5.728632 -1.472536 5.799520
H -6.869629 -2.819774 5.911365
H -5.458954 -2.946907 4.849812
H -8.758462 -1.290874 5.413343
H -7.665900 0.095033 5.213161
H -8.804844 -0.331182 3.926611
H 1.659471 -0.914759 1.608178
C 2.549871 -1.137631 2.347356
C 2.449553 -0.093424 3.397545
C 2.393645 -2.538622 2.805903
H 3.405122 -0.995062 1.683653
C 3.071102 1.154047 3.243129
C 1.655970 -0.343563 4.532053
C 1.587444 -2.801729 3.929508
C 2.936163 2.136749 4.220606
H 3.651838 1.352717 2.346097
C 1.528176 0.649108 5.506171
C 0.901531 -1.650270 4.629045
C 2.163204 1.883309 5.357733
H 3.431070 3.095504 4.097030
H 0.918952 0.454727 6.385765
H 0.705519 -1.902568 5.678013
H -0.084965 -1.492070 4.164260
H 2.055874 2.644114 6.125994
C 1.406185 -4.123843 4.340125
H 0.789557 -4.329842 5.212002
C 2.986549 -3.601076 2.108317

H	3.583922	-3.387717	1.226294
C	2.791664	-4.915829	2.521550
C	1.999827	-5.178396	3.643170
H	3.248503	-5.732337	1.969547
H	1.845918	-6.201326	3.975950

References

(S1) Armarego, W. L. F.; Chai, C. L. L. *Purification of Laboratory Chemicals,* 6th ed.; Pergamon Press: Oxford, 2009.

(S2) H. Saltzman.; J. G. Sharefkin. In *Organic Syntheses,* Wiley, New York, 1973, Vol. V, pp 658.

(S3) Liu, M.; Dogutan, D. K.; Nocera, D. G. Synthesis of Hangman Chlorins. *J. Org. Chem.* **2010**, *85*, 5065-5072.

(S4) Dogutan, D. K.; Bediako, D. K.; Teets, T. S.; Schwalbe, M.; Nocera, D. G. Efficient Synthesis of Hangman Porphyrins. *Org. Lett.* **2010**, *12*, 1036-1039.

(S5) Nam, W.; Lim, M. H.; Oh, S. Y. Effect of Anionic Axial Ligands on the Formation of Oxoiron (IV) Porphyrin Intermediates. *Inorg. Chem.* **2000**, *39*, 5572-5575.

(S6) rRaman samples were prepared in acetone-d_6: CH_3CN=1:1 to use $H_2{}^{18}O$. ESI-MS spectra confirmed that more than 50% of ^{18}O had been incorporated to the [Fe^{IV}(O)HPX] species under these conditions. We confirmed that the UV-vis spectra and reactivities of [Fe^{IV}(O)HPX] species prepared in acetone were identical to those of [Fe^{IV}(O)HPX] species prepared in CH_3CN.

(S7) Frisch, M. J.; Trucks, G. W.; Schlegel, H. B.; Scuseria, G. E.; Robb, M. A.; Cheeseman, J. R.; Scalmani, G.; Barone, V.; Mennucci, B.; Petersson, G. A.; Nakatsuji, H.; Caricato, M.; Li, X.; Hratchian, H. P.; Izmaylov, A. F.; Bloino, J.; Zheng, G.; Sonnenberg, J. L.; Hada, M.; Ehara, M.; Toyota, K.; Fukuda, R.; Hasegawa, J.; Ishida, M.; Nakajima, T.; Honda, Y.; Kitao, O.; Nakai, H.; Vreven, T.; Montgomery, J. A., Jr.; ; Peralta, J. E.; Ogliaro, F.; Bearpark, M.; Heyd, J. J.; Brothers, E.; Kudin, K. N.; Staroverov, V. N.; Kobayashi, R.; Normand, J.; Raghavachari, K.; Rendell, A.; Burant, J. C.; Iyengar, S. S.; Tomasi, J.; Cossi, M.; Rega, N.; Millam, N. J.; Klene, M.; Knox, J. E.; Cross, J. B.; Bakken, V.; Adamo, C.; Jaramillo, J.; Gomperts, R.; Stratmann, R. E.; Yazyev, O.; Austin, A. J.; Cammi, R.; Pomelli, C.; Ochterski, J. W.; Martin, R. L.; Morokuma, K.; Zakrzewski, V. G.; Voth, G. A.; Salvador, P.; Dannenberg, J. J.; Dapprich, S.; Daniels, A. D.; Farkas, O.; Foresman, J. B.; Ortiz, J. V.; Cioslowski, J.; Fox, D. J. *Gaussian 16, Revision C.01* Gaussian, Inc., Wallingford CT, 2019.

(S8) Grimme, S.; Antony, J.; Ehrlich, S.; Krieg, H. A Consistent and Accurate Ab initio Parametrization of Density Functional Dispersion Correction (DFT-D) for the 94 Elements H-Pu. *J. Chem. Phys.* **2010**, *132*, 154104.

(S9) Grimme, S.; Ehrlich, S.; Goerigk, L. Effect of the Damping Function in Dispersion Corrected Density Functional Fheory. *J. Comput. Chem.* **2011**, *32*, 1456-1465.

(S10) Marenich, A. V.; Cramer, C. J.; Truhlar, D. G. Universal Solvation Model Based on Solute Electron Density and on a Continuum Model of the Solvent Defined by the Bulk Dielectric Constant and Atomic Surface Tensions, *J. Phys. Chem. B.* **2009**, *113*, 6378-639
